# Time-Series Autoregressive Models for Point and Interval Forecasting of Raw and Derived Commercial Near-Infrared Spectroscopy Measures: An Exploratory Cranial Trauma and Healthy Control Analysis †

**DOI:** 10.3390/bioengineering12070682

**Published:** 2025-06-21

**Authors:** Amanjyot Singh Sainbhi, Logan Froese, Kevin Y. Stein, Nuray Vakitbilir, Rakibul Hasan, Alwyn Gomez, Tobias Bergmann, Noah Silvaggio, Mansoor Hayat, Jaewoong Moon, Frederick A. Zeiler

**Affiliations:** 1 Department of Biomedical Engineering, Price Faculty of Engineering, University of Manitoba, Winnipeg, MB R3T 5V6, Canada; steink34@myumanitoba.ca (K.Y.S.); vakitbir@myumanitoba.ca (N.V.); hasanr2@myumanitoba.ca (R.H.); bergmant@myumanitoba.ca (T.B.); frederick.zeiler@umanitoba.ca (F.A.Z.); 2 Department of Clinical Neurosciences, Karolinska Institutet, 171 77 Stockholm, Sweden; logan.froese@ki.se; 3 Undergraduate Medicine, Rady Faculty of Health Sciences, University of Manitoba, Winnipeg, MB R3T 3P5, Canada; 4 Section of Neurosurgery, Department of Surgery, Rady Faculty of Health Sciences, University of Manitoba, Winnipeg, MB R3T 1R9, Canada; gomeza35@myumanitoba.ca (A.G.); mansoor.hayat@umanitoba.ca (M.H.); jaewoong.moon@umanitoba.ca (J.M.); 5 Department of Human Anatomy and Cell Science, Rady Faculty of Health Sciences, University of Manitoba, Winnipeg, MB R3T 0J9, Canada; silvaggn@myumanitoba.ca; 6 Pan Am Clinic Foundation, Winnipeg, MB R3M 3E4, Canada; 7 Division of Anaesthesia, Department of Medicine, Addenbrooke’s Hospital, University of Cambridge, Cambridge CB2 0QQ, UK

**Keywords:** autoregressive integrative moving average structure, cerebral oximetry index, interval forecast, near-infrared spectroscopy, point forecast, regional cerebral oxygen saturation, time series

## Abstract

Cerebral near-infrared spectroscopy (NIRS) systems have been demonstrated to continuously measure aspects of oxygen delivery and cerebrovascular reactivity. However, it remains unknown whether the prediction of these cerebral physiologic signals into the future is feasible. Leveraging existing archived data sources, four point and interval-forecasting methods using autoregressive integrative moving average (ARIMA) models were evaluated to assess their ability to predict NIRS cerebral physiologic signals. NIRS-based regional cerebral oxygen saturation (rSO_2_) and cerebral oximetry index signals were derived in three temporal resolutions (10 s, 1 min, and 5 min). Anchored- and sliding-window forecasting, with varying model memory, using point and interval approaches were used to forecast signals using fitted optimal ARIMA models. The absolute difference in the forecasted and measured data was evaluated with median absolute deviation, along with root mean squared error analysis. Further, Pearson correlation and Bland–Altman statistical analyses were performed. Data from 102 healthy controls, 27 spinal surgery patients, and 101 traumatic brain injury patients were retrospectively analyzed. All ARIMA-based point and interval prediction models demonstrated small residuals, while correlation and agreement varied based on model memory. The ARIMA-based sliding-window approach performed superior to the anchored approach due to data partitioning and model memory. ARIMA-based sliding-window forecasting using point and interval approaches can forecast rSO_2_ and the cerebral oximetry index with reasonably small residuals across all populations. Correlation and agreement between the predicted versus actual values varies substantially based on data-partitioning methods and model memory. Further work is required to assess the ability to forecast high-frequency NIRS signals using ARIMA and ARIMA-variant models in healthy and cranial trauma populations.

## 1. Introduction

Cerebral physiology encapsulates the functional processes of the brain and includes intricate regulatory mechanisms, such as cerebral autoregulation (CA), to ensure optimal brain function. CA is the physiologic process where CBF is kept relatively constant over a wide range of systemic arterial blood pressures (ABP) or cerebral perfusion pressures (CPP) [1,2]. Cerebrovascular reactivity (CVR) is the mechanism that maintains a constant CBF by the constriction and dilation of cerebral blood vessels [1,2] and is a broader term for CA. Impairments in CA have been documented in a range of neuropathological states, such as traumatic brain injury (TBI) [3,4,5,6,7,8], and its exposure in various neuropathological conditions is a major contributor to unfavorable long-term outcomes [5,7,9,10,11]. At the bedside, indirect measurements of CA can be achieved by using CVR metrics (i.e., pressure reactivity index [PRx]) [3], since they evaluate the relationship between slow-wave (i.e., 0.05–0.005 Hz) vasogenic fluctuations in driving pressure (i.e., ABP or CPP) [12,13] and a surrogate for pulsatile CBF or cerebral blood volume (CBv; i.e., intracranial pressure [ICP]) [14,15,16,17,18], and are derived using Pearson correlation coefficients.

Various aspects of cerebral physiology can be measured non-invasively using near-infrared spectroscopy (NIRS) systems. Regional cerebral oxygen saturation (rSO_2_) is a NIRS-based signal that looks at the local cerebral oxygen content and is a surrogate for pulsatile CBv [14,16,17,18]. The moving Pearson correlation coefficients between slow-wave fluctuations of driving pressure (ABP or CPP) and surrogate for pulsatile CBv (rSO_2_) produce the CVR metric known as the cerebral oximetry index (COx and COx-a, respectively) [14,15,17,18,19,20,21]. The most established method to assess CVR in the time domain is the invasive metric known as PRx (correlation between ICP and ABP) [22,23,24]. However, the NIRS-based CVR metric has been demonstrated to assess aspects of the lower limit of autoregulation in the pre-clinical literature [14,25], similar to the ICP-based CVR metric. Additionally, high-frequency NIRS has identified executive dysfunction, referring to the impairment of cognitive neural mechanisms that are essential for humans in executing a goal-directed task, in neurocognitive disorder patients following TBI [26]. Therefore, NIRS-based signals have the potential to assess cerebral physiology in many populations because they can be measured/derived entirely non-invasively and can be applied in a multi-channel capacity to overcome spatial resolution.

At present, there is a lack of information on the predictive accuracy of NIRS-based cerebral signals over time and the influence of a system’s sampling frequency on the predictive capacity of the measured or derived NIRS cerebral physiology. High-resolution monitoring presents challenges related to data overload, necessitating resolution reduction as a common mitigation strategy, but the impact of the predictive capacity of NIRS signals at a reduced temporal resolution remains uncertain. A univariate model known as autoregressive integrative moving average (ARIMA) has been employed in several fields for data prediction, but within the health-related physiologic research, it remains in its early developmental stages [27]. Using fitted optimal ARIMA models, point and interval predictions can be made for the NIRS-based rSO_2_ signal and the derived CVR index of COx/COx-a. The focus of point prediction is to forecast a single value at a specific point in time, while interval prediction aims to forecast a range of values over a period of time. Anchored- and sliding-window methods, with varying model memory, were employed with point and interval forecasting approaches to evaluate the effect of model memory on model performance. Currently, it is unknown whether ARIMA point and interval forecastings of rSO_2_ and COx/COx-a signals are feasible using data from commercially available NIRS systems with low temporal resolution, and how model memory in using the point and interval approaches affects the model’s forecasting performance. This exploratory study evaluates the feasibility of forecasting using an optimal ARIMA model. While ARIMA-variant modeling and forecasting are outside the scope of this exploratory study, it lays the groundwork for future assessments of such models. A preliminary version of this work has been reported at the 16th Symposium of the International Neurotrauma Society (INTS) conference in Cambridge, UK [28]. The goal of this retrospective database study is to explore the role of ARIMA models in point and interval forecasting to assess how well they can predict the rSO_2_ and COx/COx-a signals from healthy and cranial trauma populations, with confirmation through correlation and agreement analysis.

## 2. Materials and Methods

### 2.1. Study Design and Human Populations

High-resolution cerebral physiologic data were retrospectively accessed from the collection of multimodal monitoring physiology at the University of Manitoba Multi-Omic Analytics and Integrative Neuroinformatics in the Human Brain (MAIN-HUB) Lab, similar to previous works from our group [27,29,30]. Three separate populations of archived data were accessed for this study, including A. healthy volunteers [29], B. elective spinal surgery patients [29], and C. TBI patients [27] (herein denoted as HC, SP, and TBI, respectively). These three populations were accessed to provide comparisons between those in normal states (i.e., HC), normal states under anesthesia (i.e., SP), and neuropathological states (i.e., TBI).

The HC group consisted of volunteers who were over the age of 18 and had no personal history of any neurological or cardiovascular conditions. They participated in having bifrontal NIRS and non-invasive ABP data recorded for 30 min in an awake sitting state.

The SP group consisted of adults, age 18 years or older, who underwent elective spinal surgery (no intradural work) with bifrontal NIRS and invasive ABP data collected throughout their procedure.

The TBI cohort included adult patients (age 18 years or older) with moderate/severe TBI admitted to the Health Sciences Centre intensive care unit (ICU; Shared Health Manitoba) between the years of 2017 and 2024, for the purpose of invasive ICP monitoring and care provision in line with the existing standard Brain Trauma Foundation (BTF) guidelines [31]. Such patients had invasive ICP, invasive ABP, and NIRS data collected during their acute phase of care in the ICU. Based on the underlying pathology, either good unilateral or bilateral NIRS data was used in the analysis since the TBI patients included were without bifrontal lobe pathology, without left frontal lobe pathology, or without right frontal lobe pathology. The presence of pathology was defined as underlying extra-axial or intra-axial lesions on admission computed tomography (CT) of the brain.

### 2.2. Ethical Considerations

Data were collected following full approval by the University of Manitoba Health Research Ethics Board (TBI = H2017:181, H2017:188, B2018:103, B2023:001, H2024:266; HC = B2020:019; SP = B2020:104) and the Shared Health/Health Sciences Centre Research Impact Committee.

### 2.3. Data Collection

For all three populations, high-frequency ABP data (100 Hz), along with left and right rSO_2_ data (rSO_2__L and rSO_2__R, respectively; 1 Hz), were collected, but the conditions and signal measurement systems differed among the three populations. For the HC group, ABP was noninvasively collected using the Finapres Nova finger cuff (Finapres Medical Systems, Enschede, The Netherlands). For the SP group, ABP was invasively obtained through arterial lines connected to pressure transducers zeroed at the level of the tragus (Baxter Healthcare Corp. CardioVascular Group, Irvine, CA, USA). For the TBI group, ABP was invasively captured using radial arterial lines, ICP was invasively measured using either intra-parenchymal strain gauge probes (Codman ICP MicroSensor; Codman & Shurtlef Inc., Raynham, MA, USA) placed in the frontal lobe or external ventricular drains (n = 3; Medtronic, Minneapolis, MN, USA), and CPP was derived as the arithmetic difference between the mean ABP and ICP. For all groups, the rSO_2__L and rSO_2__R were determined with NIRS regional oximetry monitoring pads on the left and right forehead, superficial to the frontal lobes (Covidien INVOS 5100C or 7100, Medtronic, Minneapolis, MN, USA).

All physiological data were recorded and digitized in high frequency resolution (100 Hz, where available) using Intensive Care Monitoring “Plus” (ICM+) data acquisition software (Version 8.5, Cambridge Enterprise Ltd., Cambridge, UK, https://icmplus.neurosurg.cam.ac.uk/, accessed on 31 August 2023) and analog-to-digital signal converters (Data Translations, DT9804 or DT9826) where needed. ABP in all populations and CPP in the TBI population were recorded at a 100 Hz sampling rate, while the NIRS signals were recorded at a 1 Hz sampling rate and subsequently up-sampled. In all populations, NIRS was recorded bilaterally, but only the frontal sides without pathology were used in the TBI patients

The patient demographic data were collected for cohort characterization, where available, and included the duration of recording, age, biological sex, hand dominance, procedure type, anesthetic regimen, admission Glasgow Coma Scale (GCS; total and motor sub-score), hypoxia, hypotension, arterial partial pressure of carbon dioxide (pCO_2_), arterial partial pressure of oxygen (pO_2_), procedure type, admission pupillary response, Marshall CT score, and Rotterdam CT score.

### 2.4. Physiologic Data Cleaning and Processing

The recorded high-resolution physiologic data were cleaned and processed afterwards using ICM+ software because the goal of this study was to evaluate the ability to only forecast normal physiologic signals and not signals containing artifactual segments. Signal artifacts are erroneous segments in collected signal data that can occur in monitoring due to equipment malfunction, patient motion, and the degradation of signal quality. Autonomous basic signal thresholding is unable to remove signal artifacts due to the complex morphology and non-stationarity of cerebral signals, and our lab is actively working on a semi-/fully autonomous method of artifact detection and removal [32]. Currently, there is a lack of a universally accepted method or signal-specific methods for artifact detection and management. All signal artifacts were removed from the data streams using manual methods by qualified personnel, similar to past work by our lab [27,29,30]. Each patient recording was graphed using ICM+ software to be manually inspected by qualified personnel, blinded to the study, for the removal of erroneous parts of each signal to eliminate irregular waveforms or implausible values (low or high) mostly due to patient motion, device disconnection, or drain opening in the case of invasive ICP monitoring.

For each patient, the ABP, rSO_2__L, and rSO_2__R (ICP and CPP as well for TBI patients) were decimated using non-overlapping moving average filters of 10 s duration. Decimating the data after manual artifact removal allows for focusing on the slow-wave vasogenic fluctuations associated with CA [12,13] without corrupting the true vasogenic signal. Then, moving Pearson correlation coefficients were calculated using 30 consecutive 10 s mean windows (i.e., five min of data), updated every 10 s, to derive the following rSO_2_-based CVR index for both sides using ABP [25,33]: left cerebral oximetry index with ABP (COx-a_L—correlation between rSO_2__L and ABP) and right cerebral oximetry index with ABP (COx-a_R—correlation between rSO_2__R and ABP). For TBI patients, an additional rSO_2_-based CVR index for both sides using CPP was derived: left cerebral oximetry index with CPP (COx_L—correlation between rSO_2__L and CPP) and right cerebral oximetry index with CPP (COx_R—correlation between rSO_2__R and CPP). These CVR indices have a range from −1 to +1, since they were computed using Pearson correlation coefficients, with higher values (closer to +1) indicating an impaired CVR and values below the range of 0 to +0.4 indicating an intact CVR [25,34].

Although all the data were obtained with the highest resolution of 10 s, the data was also reduced to 1 min and 5 min resolutions (using non-overlapping moving average filters) to reflect commonly available export frequencies from commercial bedside patient monitoring systems. The additional temporal resolution reductions were applied to the signals to mimic the low export frequency of most commercial bedside systems in a clinical environment. Furthermore, if data could be exported at high frequency, two key challenges arise to the clinical end-users: 1. heightened bedside computational requirements lead to infeasibility in most clinical environments that requires lower frequency data streams, and 2. limitations in clinical treatment adjustments arise from the absence of direct closed-loop feedback system for rapidly updating models, as a healthcare team can only react at a limited pace to dynamic data. The analysis, discussed in the next section, was performed using all three data resolutions. The reduction in temporal resolution was achieved with a non-overlapping moving average to each physiologic signal column of the 10 s temporal resolution data for a patient.

### 2.5. Statistical Data Analysis

All statistical analyses were performed with alpha (α) set at 0.05 for statistical significance. General data distributions were summarized using median, interquartile range (IQR), number, and percentage, where appropriate. The overall analysis of point and interval forecasting encompassed:

1. Determining the optimal time-series structures of rSO_2_, COx, and COx-a signals (in 10 s, 1 min, and 5 min temporal resolutions);
2. Cataloging the computational duration for each point and interval forecasting method (using both anchored and sliding-window approaches);
3. Evaluating the absolute forecast residual and root mean squared error between the forecasted and observed data (in 10 s, 1 min, and 5 min temporal resolutions with varying update intervals);
4. Pearson correlation and Bland–Altman agreement analyses between the forecasted and observed data of each point and interval forecasting method

In the sub-sections to follow, the individual methods will be briefly covered.

#### 2.5.1. Optimal Time-Series Structures of rSO_2_, COx, and COx-a Signals—10-s, 1-mim, and 5-min Data

General methods for optimal time-series ARIMA structures can be found described in File S1a,b, and our previous work on the subject [27,30]. In brief, data stationarity and optimal autoregressive order (p-order) and moving average order (q-order) were determined for rSO_2_, COx, and COx-a signal sources in all three temporal resolutions, in all three human populations, using standard Box–Jenkins methodologies while the integrative order (d-order) was held at 1 [35,36,37]. Stationarity analysis was performed for each physiologic signal at an individual level using augmented Dickey–Fuller (ADF) and Kwiatkowski–Phillips–Schmidt–Shin (KPSS) tests [37]. Optimal ARIMA model orders were determined using the lowest Akaike information criterion (AIC) values for the derived ARIMA models. A median optimal ARIMA model order was determined for each signal in all three temporal resolutions, along with the IQR of optimal ARIMA model order across all populations.

#### 2.5.2. Computational Duration of Point and Interval Forecasting Methods—Anchored and Sliding-Window Approaches

The computation time, in minutes, was recorded for each forecasting method per patient in each combination of three temporal resolutions to varying forecasting intervals. Anchored forecasting refers to the usage of a minimum of 80% data to fit a patient–signal-specific optimal ARIMA model, while the rest of the data is used to test against the fitted ARIMA models using point and interval approaches (hereon denoted as anchored point and anchored interval, respectively). Sliding-window forecasting refers to the usage of a window of data points to fit an optimal ARIMA model and by using point (overlapping sliding window) and interval (non-overlapping sliding window) approaches (herein denoted as windowed point and windowed interval, respectively). The data can be tested against the fitted ARIMA models. The interval of 5 min to 1 day was used but the interval range varied between populations based on the amount of the data length..

**Figure 1:**
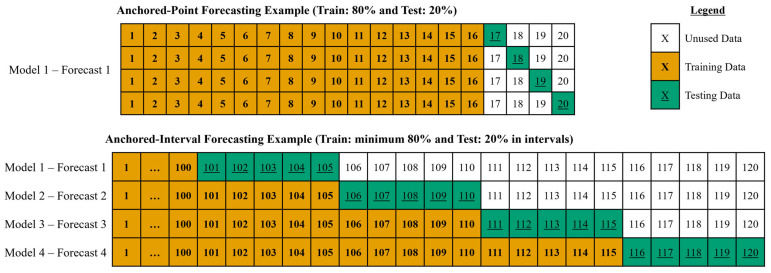
The figure is a visual representation example to compare the training data for model fit versus testing data for ARIMA forecasting using anchored-point and anchored-interval methods. The anchored-point forecasting example is of a signal with a total of 20 data points, and it fits only one optimal ARIMA model using 80% of data (16 data points) to forecast the rest of the 20% of data (4 data points d). The anchored-interval forecasting of a signal with a total of 120 data points using a 5-point forecasting interval. With an optimal ARIMA model, it fits a minimum of 80% of data (100 data points) to forecast the next data interval (5 data points). After each forecasting, it incorporates the 5-point interval of measured data to forecast the next interval of data. ARIMA, autoregressive integrative moving average.

Anchored-point forecasting used only the first 80% of a signal’s data to fit one optimal ARIMA model to forecast the rest of the 20% of the data in all the temporal resolutions. Anchored-interval forecasting used a minimum of 80% of a signal’s data to fit the optimal ARIMA models to forecast the upcoming interval of the data. Moving into the next forecasting, the optimal ARIMA model is again fit using 80% of the signal’s data, along with incorporating the measured interval data for the next data forecast interval. An example of the amount of training data versus testing data for anchored-point and anchored-interval forecasting is depicted visually in Figure 1.

**Figure 2:**
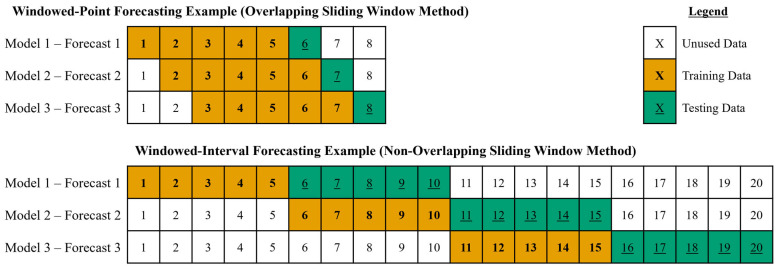
The figure is a visual representation example to compare the training data for model fit versus testing data for ARIMA forecasting using windowed-point and windowed-interval methods. The windowed-point example represents a signal with a total of 8 data points and fits an optimal ARIMA model using a data window (5 data points) to forecast the next data point (1 data point) in an overlapping manner. The windowed-interval example represents a signal with a total of 20 data points, and it uses a fitted optimal ARIMA model using a data window (5 data points) to forecast the next 5 min interval of data (5 data points) in a non-overlapping manner. ARIMA, autoregressive integrative moving average.

Windowed-point forecasting used a window of data points to fit optimal ARIMA models to forecast a single upcoming data point. Then, the optimal ARIMA model was refit by moving the data points window over by one data point to forecast the next data point, and this was performed continuously using the overlapping sliding-window method to forecast data points. Windowed-interval forecasting used a window of data points to fit the optimal ARIMA models to forecast the upcoming interval of points, and the interval length was set to be equal to the sliding-window length. Then, for the next interval of forecasting data points, the window of data points was moved over by the length of the sliding window to refit the optimal ARIMA model and forecast the next interval of data points. This was performed continuously using the non-overlapping sliding-window method to forecast the interval of data points. An example of the amount of training data versus testing data for windowed-point and windowed-interval forecasting is depicted visually in Figure 2.

#### 2.5.3. Absolute Forecast Residual—10-s, 1 min, and 5 min Data

The absolute forecast residual (AFR) was determined for each signal in individual patients, from all three populations, by obtaining the absolute difference of the forecasted and measured data using rSO_2_, COx, and COx-a signals, where available. The data resolutions of 10 s, 1 min, and 5 min were used. The AFRs were calculated for each signal of a patient to determine its median AFR. We were able to derive the median absolute deviation (MAD) of the AFRs for the entire population by finding the median of the absolute deviation of each AFR from the median of AFR.

#### 2.5.4. Root Mean Squared Error Analysis of Forecasted and Observed Data—10 s, 1 min, 5 min Data

A root mean squared error (RMSE) analysis was performed to check the model’s performance by observing the average magnitude of errors between the observed and forecasted data in each population. RMSE was performed on the rSO_2_, COx, and COx-a signals, where available, at 10 s, 1 min, and 5 min temporal resolutions. The RMSE was calculated for each signal of a patient. Then, the median RMSE was found for each signal in all data resolutions and window/interval combinations, along with deriving the median absolute deviation (MAD).

#### 2.5.5. Pearson Correlation Analysis of Forecasted and Observed Data—10 s, 1 min, 5 min Data

Pearson correlation analysis was performed on each observed set of data and its forecasted data to check for their correlation in each signal for a patient using all four forecasting approaches. The Pearson correlation coefficient (*r*) and *p*-value were obtained, along with their median and interquartile range (IQR) values for each population in all forecasting methods and all temporal resolutions. The value of *r* indicates the strength of the correlation, and the *p*-value signifies whether the result was significant, with the alpha (α) set at 0.05 for statistical significance.

#### 2.5.6. Bland-Altman Agreement Analysis of Forecasted and Observed Data—10-s, 1-min, 5-min Data

A Bland–Altman agreement analysis was performed to check the agreement between the observed and forecasted data of each signal for a patient in all four forecasting approaches. The bias, limits of agreement (LoA), spread of LoA, bias as proportion of LoA spread (relative bias), and the regressed slope and intercept were obtained along with their median and IQR values for each population in all forecasting methods and temporal resolutions. The bias is the mean difference, which indicates the magnitude of the systemic difference. LoA is the mean difference with ±1.96 standard deviations of differences. The spread of LoA informs about the agreement. The relative bias helps understand the magnitude of bias relative to the global changeability of the differences, and the regression equation informs about the underestimation or overestimation.

## 3. Results

### 3.1. Population Demographics

A total of 102 HC, 27 SP, and 101 TBI patients were included in this study. The median recording duration was 31.1 min (IQR: 28.7–34.8 min) for HC, 185 min (IQR: 165.8–206.8 min) for SP, and 4644 min (IQR: 2369.3–7942.3 min) for TBI. Table 1 shows the summary of patient admission demographics for all the populations, and File S1c shows additional injury information for SP and TBI populations, as each population was different due to the injury type or lack of it.

### 3.2. Optimal ARIMA Structure Analysis—10 s, 1 min, 5 min Data

Before deriving the optimal ARIMA structure, each signal’s stationarity was assessed for all patient data for each temporal resolution. Across all three populations and temporal resolutions, not all physiologic signals were strictly stationary according to the ADF and KPSS tests. These signals were made strictly stationary by first-order differencing (d-order set as one) and then rechecked for their stationarity by the ADF and KPSS tests. Table 2 provides the general results for the ADF and KPSS testing on first-order differenced COx and COx-a data, while File S2 provides such analyses on the remaining signals. Note in these tables and appendices that the NA represents that the stationarity could not be determined in those patients due to an inadequate number of data points.

Each patient’s optimal ARIMA models for every signal were found separately across all temporal resolutions using the lowest AIC value, and a median optimal ARIMA model was determined for all temporal resolutions of each signal. It can be seen that the median optimal ARIMA model of the HC population tends to stay in the lower ARIMA models range at 10 s and 1 min temporal resolutions but jumps to near the middle of the ARIMA models’ range. The median optimal ARIMA model for the SP population is in the higher ARIMA model range at 10 s temporal resolution but decreases by temporal resolution to the lower ARIMA model range at 5 min temporal resolution. The TBI population’s median optimal ARIMA model has a similar trend to the SP population, but its median optimal ARIMA model decreases to just below the middle ARIMA model range.

There seems to be a clear difference in the median optimal ARIMA models, where the HC population had the lowest median autoregressive structure, while the TBI population had the highest median autoregressive structure in most of the temporal resolutions. However, the autoregressive structure of the signals was fairly similar in each population. Table 3 shows the compiled population results of the median optimal ARIMA model for the combination of each signal and temporal resolution. File S3 shows a TBI patient example AIC recorded while fitting various ARIMA models and a 10 s temporal resolution example of the optimal ARIMA model of each signal per patient in all three populations.

### 3.3. Computational Duration Analysis for Point and Interval Methods Using Anchored and Sliding-Window Approaches

The time it took to forecast all signals for each patient using each forecasting type was recorded from start to finish for each window/interval method. Looking at the temporal resolution of 10 s, the median CSV signals file size was 9.2 kilobytes (KB; IQR: 8.6 KB–10.4 KB) for the HC population, 59.2 KB (IQR: 51.8 KB–66.4 KB) for the SP population, and 1917.4 KB (IQR: 792.3 KB–2951.9 KB) for the TBI population. File S4a lists the median and IQR of the data file sizes using all three temporal resolutions in each population. Anchored-point forecasting was the quickest amongst the forecasting types, since the computational duration was less than one min for each HC and SP patient, and TBI patients had a median computational duration of 2 min (IQR: 1–5 min).

Anchored-interval forecasting has the second-highest computational duration time, with less than one min of computational duration for the HC population using 5 min and 10 min intervals. The intervals of 5 min, 10 min, 15 min, 30 min, and 1 h were used in anchored-interval forecasting for the SP population, with the highest median computational duration of 1 min (IQR: 0.5–2 min) using a 5 min interval. Decreasing computational durations were seen as the interval increased to 1 h, which had a median computational duration of 1 min. The TBI population used all intervals as the SP population, with the addition of 2 h, 6 h, 12 h, and 1-day intervals. Again, the 5 min interval had the highest median computational duration of 535 min (IQR: 87–3101 min), and a decrease in computational durations was seen as intervals increased to 1-day, having a median computational duration of 31.5 min (IQR: 19.5–53.5 min).

Windowed-point forecasting has the highest computational duration time, with the median computational duration of the HC population being 1 min (IQR: 0–1 min) for the 5 min window to the 30-min window having a median computational duration of less than 1 min. For the SP population, a trend of increasing computational duration time was seen as the window length increased, with the 5 min window having a median computational duration of 12 min (IQR: 7–17.5 min) to 6 h window having median computational duration of 67 min (IQR: 56.5–77.5 min). A similar increase of computational duration with an increase in window length was observed in the TBI population, with the 5 min window having a median computational duration of 453 min (IQR: 165–992 min) to the 1-day window having median computational duration of 10,357 min (IQR: 5870–16,993 min).

Windowed-interval forecasting has the second-lowest computational duration time, with the HC population having less than 1 min of computational duration for a 5 min to 15 min window and interval length. In the SP population, a trend of decreasing computational duration time was seen as the window and interval length increased, with a 5 min window and interval length having a median computational duration of 1 min (IQR: 0–1 min) to less than 1 min of computational duration time for a 6 h window and interval length. A similar decrease in computational duration with an increase in window and interval length was seen in the TBI population, with a 5 min window and interval length having a median computational duration of 33 min (IQR: 13–77 min) to 9 min (IQR: 3.5–21 min) for a 1-day window and interval length.

The order of the forecast methods from lowest to highest computational duration was anchored point, windowed interval, anchored interval, and windowed-point. The computational duration for the HC population was unnoticeable due to it having the least amount of data but became prominent in the SP population during windowed-point forecasting. The TBI data took the longest to forecast, particularly when using the windowed-point and anchored-interval methods, due to longer recordings spanning multiple days. As the temporal resolution of the data was decreased to 1 min and 5 min, the CSV signal’s file size also decreased, along with a decrease in the overall medians for each forecasting method, without any change to the computational duration order of the forecasting methods. Also, the trend of computational duration to window/interval length remained the same. Table 4 shows the median and IQR of the computational duration of all forecasting methods in the 10 s temporal resolution for all three populations. File S4 shows similar computational duration results for 1 min and 5 min temporal resolutions for all three populations.

### 3.4. Absolute Forecast Residual Analysis—10 s, 1 min, 5 min Data

The absolute difference between the forecasted and measured data was taken to calculate the AFR of each signal per patient. For anchored-point forecasting, the sequence of groups with the lowest to highest median AFR for both sides of rSO_2_ were HC, SP, and TBI (~0.9, ~2.1, and ~3.1, respectively), but this sequence changes for COx-a to HC, TBI, and SP (~0.2, ~0.22, and ~0.35, respectively). The median AFR for both sides of COx in TBI was similar to COx-a (~0.23). It was seen that the HC group had the lowest MAD of either the left and right side AFR of rSO_2_, the SP group had medium values, and the TBI group had the largest values (~0.7, 1.5, and ~2.3, respectively). For the MAD of either the left and right side AFR of COx-a, again, the HC group had the lowest values, but the TBI group had medium values, while the SP group had the largest values (0.12, 0.19, and 0.26, respectively). The MAD of the left and right sides’ AFR of COx in TBI patients (~0.2) was similar to COx-a sides. HC and TBI groups had the lowest COx-a deviations using anchored-point forecasting, but for the SP group, it is considered a big deviation for the index. Similar results were seen at 1 min and 5 min temporal resolutions. Table 5 shows these AFR results using anchored-point forecasting at 10 s temporal resolution for all three populations, while File S5a shows the results for the MAD of AFR. File S5b shows a similar AFR and MAD for the AFR results at 1 min and 5 min temporal resolutions for all three populations.

With the anchored-interval forecasting using 5 min intervals, the sequence of groups with the lowest to highest median AFR for both sides of rSO_2_ was TBI, SP, and HC (~0.54, ~0.82, and ~0.85, respectively). This sequence changes for COx-a to HC, TBI, and SP (~0.15, ~0.16, and ~0.23, respectively), and the median AFR for both sides of the COx in TBI was the same as COx-a (~0.16). It was seen that the TBI group had the lowest MAD of either the left or right side AFR of rSO_2_. The HC group had medium values, and the SP group had the largest values (~0.57, ~0.7, and ~0.85, respectively). For the MAD of either the left and right side AFR of COx-a, the lowest values were seen in the HC group, while the TBI group had medium values, and the SP group had the largest values (~0.12, ~0.16, and ~0.21, respectively). The MAD of either side of the AFR of COx in TBI patients (~0.16) was the same as the COx-a sides. Most populations had relatively small COx-a deviations at a 5 min interval using anchored-interval forecasting. At higher intervals of up to 1 day, where possible, a marginal increase was seen in the AFR values for the signals, along with their MAD of AFR results, but the mentioned sequence did not change for higher intervals. Similar results were seen for the 1 min and 5 min temporal resolutions. Table 6 shows these AFR results using anchored-interval forecasting at 10 s temporal resolution for all three populations, while File S5c shows the results for the MAD of the AFR. File S5d,e shows similar AFR and MAD for the AFR results at 1 min and 5 min temporal resolutions, respectively, for all three populations.

With the windowed-point forecasting using 5 min intervals, the sequence of groups with the lowest to highest median AFR for both sides of the rSO_2_ were SP, TBI, and HC (~0.28, ~0.31, and ~0.54, respectively). The median AFR of COx-a was very similar amongst the HC, SP, and TBI populations (~0.04, ~0.04, and ~0.05, respectively), and the median AFR of the COx in TBI was the same as COx-a (~0.05). It was seen that the SP group had the lowest MAD of either left or right side AFR of rSO_2_. The TBI group had medium values, and the HC group had the largest values (~0.29, ~0.32, and ~0.49, respectively). For the MAD of either left or right side AFR of the COx-a, the lowest values were seen in the HC group, while the SP and TBI groups had similar values (~0.03, ~0.04, and ~0.04, respectively). The MAD of either side AFR of COx in the TBI patients (~0.04) was the same as the COx-a sides. Deviations in COx/COx-a using windowed-point forecasting were less than 0.05 amongst all populations and are considered to be small deviations for the index. At higher intervals of up to 1 day, where possible, a marginal decrease was seen in the AFR values for the signals, while their MAD results stayed fairly consistent, and the mentioned sequence did not change at higher intervals. Overall, higher AFR and MAD results were seen at 1 min and 5 min temporal resolutions compared to the results at 10 s resolution. Table 7 shows these AFR results using windowed-point forecasting at a 10 s temporal resolution for all three populations, while File S5f shows the results for the MAD of the AFR. File S5g,h shows the AFR and MAD of the AFR results at 1 min and 5 min temporal resolutions, respectively, for all three populations.

With the windowed-interval forecasting using a 5 min window and interval, the sequence of groups with the lowest to highest median AFR for both sides of the rSO_2_ were TBI, SP, and HC (~0.51, ~0.73, and ~1.04, respectively). This sequence changes for COx-a to HC, TBI, and SP (~0.19, ~0.21, and ~0.28, respectively), and the median AFR for both sides of COx in the TBI was the same as COx-a (~0.21). It was seen that the TBI group had the lowest MAD of either the left or right side AFR of rSO_2_. The SP group had medium values, and the HC group had the largest values (~0.54, ~0.74, and ~0.89, respectively). For the MAD of either the left or right side AFR of COx-a, the lowest values were seen in the HC group. The TBI group had medium values, and the SP group had the largest values (~0.18, ~0.21, and ~0.3, respectively). The MAD of either side of the AFR of COx in TBI patients (~0.21) was the same as the COx-a sides. The HC and TBI groups had the lowest COx-a deviations using a 5 min window and interval for windowed-interval forecasting, but for the SP group, it is considered a big deviation for the index. At higher windows and intervals of up to 1 day, where possible, a marginal increase was seen in the AFR values for the signals, along with their MAD results, while the mentioned sequence was similar. Similar results were seen for the 1 min and 5 min temporal resolutions. Table 8 shows these AFR results using windowed-interval forecasting at a 10 s temporal resolution for all three populations, while File S5i shows the results for the MAD of AFR. File S5j,k shows the AFR and MAD of the AFR results at 1 min and 5 min temporal resolutions, respectively, for all three populations.

Overall, the AFR and MAD values for rSO_2_ were relatively small, essentially under 1%, for all four forecasting methods, and this difference between the predicted and measured values is clinically irrelevant. For the anchored-point, anchored-interval, and windowed-interval forecasting methods, the AFR and MAD values for the COx/COx-a signals were a little worse than the windowed-point method, up to 0.2, but generally near 0.1, and it is a clinically relevant difference. The AFR analysis was used to observe the absolute difference between the forecasted data for a signal to its measured data, and other than the most computationally intensive method of windowed point, none of the other point/interval forecasting methods, at any temporal resolution, resulted in as low AFR and MAD results. With an increase in the median AFR and their MAD values in the reduced temporal resolutions, the deviations started to become large for the signals, as seen in File S5. Also, the windowed-point forecasting method had low median AFR values for each signal in all populations at the 5 min training window, but increasing the window size to 10 min and 30 min often produced a slightly closer prediction to the measured signals. This suggests that, to accurately predict single future values using the windowed-point method, we can use a shorter window to forecast the next point compared to using a day’s length of data.

### 3.5. Root Mean Squared Error Analysis—10 s, 1 min, 5 min Data

The RMSE was calculated for the forecasted and measured data of each signal per patient. For the anchored-point forecasting, the sequence of groups with the lowest to highest median RMSE for both sides of rSO_2_ were HC, TBI, and SP (~1.3, ~3.2, and ~3.9 respectively), but it stays the same for COx-a (~0.25, ~0.34, and ~0.45, respectively). The median RMSE for both sides of COx in the TBI was greater than COx-a (~2.7). It was seen that the HC group had the lowest MAD of either the left or right side RMSE of rSO_2_. The TBI group had medium values, and the SP group had the largest values (~0.6, ~2.1, and ~3.1, respectively). For the MAD of either the left or right side RMSE of COx-a, the TBI group had the lowest values, the HC group had medium values, and the SP group had the largest values (~0.05, ~0.14, and ~0.16, respectively). The MAD of the left and right side RMSE of the COx in the TBI patients (~1.6) was greater than the COx-a sides. Similar results were seen at 1 min and 5 min temporal resolutions. File S6a shows these RSME results using anchored-point forecasting at 10 s, 1 min, and 5 min temporal resolutions for all three populations.

With the anchored-interval forecasting using 5 min intervals, the sequence of groups with the lowest to highest median RMSE for both sides of the rSO_2_ were TBI, HC, and SP (~1.1, ~1.2, and ~1.9, respectively). This sequence changes for COx-a to HC, TBI, and SP (~0.23, ~0.28, and ~0.38, respectively), and the median RMSE for both sides of the COx in the TBI was greater than COx-a (~1.1). It was seen that the HC group had the lowest MAD of either the left or right side RMSE of the rSO_2_. The TBI group had medium values, and the SP group had the largest values (~0.51, ~0.69, and ~1.2, respectively). For the MAD of either the left and right side RMSE of COx-a, the lowest values were seen in the TBI group, while the SP group had medium values, and the HC group had the largest values (~0.04, ~0.13, and ~0.14, respectively). The MAD of either side of the RMSE of the COx in the TBI patients (~0.79) was greater than the COx-a sides. At higher intervals of up to 1 day, where possible, a marginal increase was seen in the RMSE values for the signals, along with their MAD for the RMSE results, but the mentioned sequence did not change for higher intervals. Similar results were seen at the 1 min and 5 min temporal resolutions. File S6b–d shows these RMSEE results using anchored-interval forecasting for all three populations at 10 s, 1 min, and 5 min temporal resolutions, respectively.

For the windowed-point forecasting using 5-min intervals, the sequence of groups with the lowest to highest median RMSE for both sides of the rSO_2_ was HC, TBI, and SP (~0.89, ~0.91, and ~1.4, respectively). The median RMSEs of the COx-a were mostly similar amongst HC, SP, and TBI populations (~0.07, ~0.10, and ~0.11, respectively), and the median RMSE of the COx in the TBI was greater than COx-a (~0.84). It was seen that the HC group had the lowest MAD of either the left or right side RMSE of the rSO_2_. The TBI group had medium values, and the SP group had the largest values (~0.32, ~0.71, and ~1.2, respectively). For the MAD of either the left or right side RMSE of the COx-a, the lowest values were seen in the HC group, while the TBI and SP groups had similar values (~0.01, ~0.02, and ~0.02, respectively). The MAD of either side of the RMSE of COx in TBI patients (~0.65) was greater than the COx-a sides. At higher intervals of up to 1 day, where possible, a marginal decrease was seen in the RMSE values for the signals, while their MAD results stayed fairly consistent, but a very high median RMSE for the rSO_2_ and COx in the TBI were seen between 30 min and 2 h using 10 s resolution. Overall, a higher RMSE and its MAD results were seen at 1 min and 5 min temporal resolutions compared to the results at 10 s resolution. File S6e–g shows these RMSE results using windowed-point forecasting at 10 s, 1 min, and 5 min temporal resolutions, respectively.

With the windowed-interval forecasting using a 5 min window and interval, the sequence of groups with the lowest to highest median RMSE for both sides of the rSO_2_ were HC, SP, and TBI (~1.6, ~4.9, and ~924, respectively). This sequence stays the same for COx-a (~0.33, ~0.61, and ~0.66, respectively), and the median RMSE for both sides of COx in the TBI was much larger than COx-a (~516). It was seen that the HC group had the lowest MAD of either the left or right side RMSE of rSO_2_. The SP group had medium values, and the TBI group had the largest values (~0.58, ~4.9, and ~1369, respectively). For the MAD of either the left or right side RMSE of the COx-a, the lowest values were seen in the HC group. The SP group had medium values, and the TBI group had the largest values (~0.12, ~0.24, and ~0.38, respectively). The MAD of either side of the RMSE of COx in the TBI patients (~762) was much larger than the COx-a sides. At higher windows and intervals of up to 1 day, where possible, a marginal decrease was seen in the RMSE values for the signals, along with their MAD results. Similar results were seen at the 1 min and 5 min temporal resolutions. File S6h–j shows these RMSE results using windowed-interval forecasting at 10 s, 1 min, and 5 min temporal resolutions, respectively.

Overall, the RMSE and MAD values for rSO_2_ were relatively small, essentially under 2%, for all four forecasting methods, and this difference between the predicted and measured values is clinically irrelevant. For the anchored-point, anchored-interval, and windowed-interval forecasting methods, the RMSE and their MAD values for the COx/COx-a signals were a little worse than the windowed-point method. These RMSE analysis results are similar to the performed AFR analysis. Since the RMSE analysis puts more weight on larger errors, the RMSE and MAD of RMSE values were quite large in the TBI population for windowed-point and windowed-interval forecasting. Even with one large error, the RMSE and its MAD can become quite large.

### 3.6. Pearson Correlation Analysis—10 s, 1 min, 5 min Data

In all populations and temporal resolutions, the overall Pearson correlation analysis of each observed and its anchored-point forecasted data performed on the rSO_2_ and COx/COx-a signals showed poor correlation strength (median *r* < 0.15), without much of a statistically significant relationship (median *p*-value > 0.05). File S7a,b shows these Pearson correlation analysis results using anchored-point forecasting at 10 s, 1 min, and 5 min temporal resolutions for all three populations.

The Pearson correlation analysis, using the anchored-interval forecasting at a 10 s temporal resolution, showed that all signals in each interval for the HC population had a poor correlation strength (median *r* < 0.26) without a statistically significant relationship (median *p*-value > 0.05). While in the SP population, both sides of the rSO_2_ signal at 5 min and 10 min intervals showed a strong correlation strength (median *r* > 0.71), with a statistically significant relationship (median *p*-value < 0.05), and the COx-a signals only showed a moderate correlation strength in the lower intervals with a statistically significant relationship. The correlation strength was seen to decrease as the interval window increased. The TBI population showed a higher correlation strength for the rSO_2_ and COx/COx-a signals compared to the SP, and the decrease in the correlation strength was also seen with an increased interval window. Similar results were seen for the 1 min and 5 min temporal resolutions. File S7c–e show these Pearson correlation analysis results using anchored-interval forecasting at 10 s, 1 min, and 5 min temporal resolutions, respectively, for all three populations.

The results of the Pearson correlation analysis, using the windowed-point forecasting at a 10 s temporal resolution, mostly showed strong correlation strength for all signals (median *r* > 0.74) in each population with a statistically significant relationship. Similar results were seen for the 1 min and 5 min temporal resolutions, but the correlation strength showed a decrease at lower resolutions. File S7f–h shows these Pearson correlation analysis results using windowed-point forecasting at 10 s, 1 min, and 5 min temporal resolutions, respectively, for all three populations.

With windowed-interval forecasting at a 10 s temporal resolution, the Pearson correlation analysis showed that all signals in each window and interval for the HC population had poor correlation strength (median *r* < 0.26) without a statistically significant relationship (median *p*-value > 0.05). While in the SP population, both sides of the rSO_2_ signal at 5 min, 10 min, and 15 min windows and intervals showed a moderate correlation strength (0.53 > median *r* > 0.69) with a statistically significant relationship (median *p*-value < 0.05), and the COx-a signals showed a low correlation strength in the lower windows and intervals with a statistically significant relationship. The TBI population showed a low correlation strength for rSO_2_ and COx/COx-a signals compared to the SP, and the decrease in the correlation strength was also seen with an increased window and interval for all populations. Similar results were seen for the 1 min and 5 min temporal resolutions, with an overall decrease in the correlation strength. File S7i–k show these Pearson correlation analysis results using windowed-interval forecasting at 10 s, 1 min, and 5 min temporal resolutions, respectively, for all three populations.

These results suggest that the ARIMA model with more memory tended to have a poorer correlation. The windowed-point forecasting showed the strongest correlation, especially at the 10 s temporal resolution, followed by windowed interval, anchored interval, and anchored point. All the other forecasting methods had varying correlation strengths for each signal. From this analysis, we found that the models that included old information, especially in the case of anchored techniques, did not help the correlation of the forecasted data to the observed data. With windowed-point and windowed-interval methods, they were able to forecast the rSO_2_ and COx/COx-a signals with strong correlation.

### 3.7. Bland–Altman Agreement Analysis—10 s, 1 min, 5 min Data

The Bland–Altman agreement analysis on the anchored-point forecasted data at a 10 s temporal resolution showed that, in all populations, the rSO_2_ and COx/COx-a signals had a large relative bias with a large LoA spread along with the median regression slope near two. With the anchored-interval data at a 10 s temporal resolution, the Bland–Altman agreement analysis showed that, in all populations, the rSO_2_ and COx/COx-a signals had a large relative bias with a large LoA spread along with a small median regression slope, but they all increased with incrementing intervals. However, the windowed-point analysis at a 10 s temporal resolution showed that all signals had a low relative bias near zero, with a low LoA spread along with the regression slope of near zero in all populations at most intervals. The agreement for the windowed-interval analysis showed that the relative bias was fairly close to zero, but the LoA spread was high, along with the regression slope often close to zero. Similar results were seen for the 1 min and 5 min temporal resolutions, with generally a higher relative bias. File S8a–k shows the Bland–Altman analysis results using all four forecasting techniques at 10 s, 1-minute, and 5 min temporal resolutions for all three populations.

These results suggest that the ARIMA model with more memory tended to have a poorer agreement. The windowed-point forecasting showed the best agreement, especially at the 10 s temporal resolution, followed by windowed interval, anchored interval, and anchored point. All the other forecasting methods had a poor agreement, as shown by a large relative bias, a large LoA spread, and a non-zero regression slope. From this analysis, we found that the models that included old information, especially in the case of anchored techniques, did not help the agreement of the forecasted data with the observed data. With windowed-point and windowed-interval methods, they were able to forecast the rSO_2_ and COx/COx-a signals with good agreement.

## 4. Discussion

To assess the point and interval forecasting of the NIRS-based rSO_2_ and COx/COx-a signals, we employed various statistical methods, including A. optimal time-series structures of rSO_2_/COx/COx-a, B. the computational duration for each point and interval forecasting method, C. the absolute forecast residual and root mean squared error between the forecasted and observed data, and D. the Pearson correlation and Bland–Altman agreement analyses performed between the forecasted and observed data. These were conducted for each point and interval forecasting method using three different population types (HC, SP, TBI) and three different data resolutions (10 s, 1 min, and 5 min). To our knowledge, this analytic approach represents the most comprehensive point and interval ARIMA-based forecasting analysis using commercial (i.e., readily available in healthcare settings) cerebral NIRS data streams. Through this exhaustive analysis, some important information on the extent of the point and interval forecasting being able to predict the measured and derived signals from the low-resolution cerebral NIRS data streams is highlighted.

First, the median optimal ARIMA model analysis showed that the autoregressive structure in the HC population was lower and higher in the TBI population, while the autoregressive structure was found to be similar in a population. As with the previous work from our lab, a first-order differencing was required since the ADF and KPSS tests showed non-stationarity in the non-differenced signals of all populations [27,30]. A point to note is that the calculated median optimal ARIMA models for a population do not accurately represent all patients in the population, as seen by the IQR of the optimal ARIMA models given in Table 3. This was the reason to use each patient’s optimal ARIMA model for each of the signals to perform the point and interval forecasting.

Second, the computational duration was recorded for comparison of all four types of point and interval methods used in forecasting. The windowed-point forecasting had the highest computational time, followed by anchored interval and windowed interval, and the quickest was Anchored-Point forecasting, as seen in Table 4 and File S4. The computational duration for forecasting using various methods sets a baseline for simple time-series forecasting and will be useful in the future to find a forecasting method that has a short computational time while giving better precision and accuracy. For clinical bedside forecasting, it is very important to consider the computation time of various forecasting approaches because the prediction of future values should not take extended periods of time, and accurate predictions should not rely on high-end hardware to fit forecasting models. Hence, this data will be useful in the future to compare with other complex, multi-variate, or deep-learning methods to find a better forecasting method to be used in the real-time or near real-time forecasting of cerebral physiological signals.

Third, the AFR analysis was used to observe the absolute difference between the forecasted data for a signal to its measured data, and the RMSE analysis was used to assess the average magnitude of errors between the forecasted and measured data. It showed that the windowed-point forecast method had the lowest median AFR for each signal in all populations, with small deviations from the median for all window sizes at the 10 s temporal resolution, as seen in Table 7, although it was the most computationally intensive method. While the lowest median RMSE for each signal in all populations was also found for the windowed-point forecast method, very high RMSEs were seen in some windows since more weights are put on larger errors, even if there was only one large error. The results of the AFR with MAD and the RMSE with MAD seem to suggest a limitation of the ARIMA models to reliably forecast future values for NIRS-based physiological signals using the four examined forecasting techniques with a temporal resolution lower than 10 s. Although it is important to note that the NIRS-based data was measured using a commercial device with a low update frequency, another outlook of these results can be that decimating the raw NIRS signal to focus on slow-wave vasogenic fluctuations at the highest resolution of 10 s may limit the useful information. This can have an effect on the derived optimal ARIMA model for a signal and raises the question about high-resolution research-grade NIRS systems for ARIMA forecasting using the examined methods. Also, a further temporal resolution reduction of the low-resolution signal from the commercial NIRS device may completely eliminate any present useful information and can explain the higher AFR with MAD and RMSE with MAD results seen when used to fit the optimal ARIMA model for the prediction of future values.

Fourth, the computational time to fit the optimal ARIMA model before forecasting a single value using the windowed-point method can be hugely reduced by limiting the window size to a range of 10 min to 30 min, since it often produces a slightly closer prediction to the measured signals. Although windowed point has performed much better than the other tested methods, further research is needed to check the ability of all tested approaches using high-frequency NIRS and for their application in real-time forecasting.

Fifth, the correlation and agreement analysis results suggest that the amount of ARIMA model memory plays a crucial role in point and interval forecasting. The use of the sliding-window forecasting approach eliminates the long-term memory in the system, which is not useful for prediction, and gives a better performance than the anchored forecasting approach. These results help direct future studies to test other linear and non-linear models that use a range of 10 min to 30 min window sizes to fit a model and forecast an interval of data up to the selected window size. Also, the use of lower window sizes to fit models helps in reducing the computational time, which will be useful when exploring alternative forecasting methods.

Finally, the treatment regimens in the SP and TBI populations, related to sedation medications, did not demonstrate an impact on the predictive ability of ARIMA forecasting using point and interval techniques with varying model memory. This aligns with the growing body of the established literature, which highlights that the effects of vasopressor and sedation drugs on continuously measured CVR indices are limited [38,39]. In our case, the prediction of NIRS rSO_2_ metric from a commercial NIRS system, with low sampling frequency, does not seem to be affected by the sedation drugs used in SP and TBI populations.

The ability for such ARIMA or ARIMA-variant techniques to model and detect artifactual segments within continuous time-series cerebral physiologic signals remains unknown. Work on semi-/fully-autonomous artifact detection and removal is ongoing by our lab with a focus on bedside artifact detection and removal in real time. Since the NIRS data lacked pulse waveform information due to the utilization of a commercial NIRS system with a limited temporal resolution of 1 Hz, the future direction would be to use high-frequency NIRS data to assess how well it performs using optimal ARIMA models to forecast data with the used point and interval techniques. Another area to investigate for future direction is to compare the linear model, such as ARIMA, to other ARIMA variants, along with other non-linear or hybrid approaches. The usage of ARIMA variants could better predict the first-order differenced time-series NIRS data by incorporating other variables or signals to create multivariate prediction models. Also, it is possible that the linear models may not be able to fully capture the complexity of physiological signals, especially at such a low temporal resolution. So, accounting for intricate relationships within the data may offer forecasting improvements, specifically with signals that display irregular or non-linear subtleties.

## 5. Limitations

Given the exploratory nature of the analysis, several broad limitations exist. First, it was a retrospective analysis of decent-sized HC and TBI populations but relatively small-sized SP population, although it was considered to be part of the healthy population due to no cranial trauma. As such, our findings should only be considered exploratory and should be taken with caution. Second, further validation in larger multi-center high-frequency physiologic datasets is warranted since these findings may not be generalizable to other HC, SP, or TBI populations due to the injury severity and heterogeneity of treatment protocols. Third, due to the low spatial resolution of the commercial NIRS system, only the recording of the bifrontal lobe was possible, and in the case of some TBI patients, only unilateral recordings were used for forecasting due to the presence of pathology under one side of the NIRS optodes. Fourth, an analysis of recordings from a single commercial NIRS system may introduce bias, with unknown applicability to other available systems in use. Finally, the time needed to train and test using the windowed-point and anchored-interval methods was computationally taxing, and implementing these methodologies on a larger cohort would benefit from more enhanced central computing services that are capable of automatically managing multiple patient analyses in parallel.

## 6. Conclusions

Point and interval forecasting for rSO_2_ and COx/COx-a signals from commercially available cerebral NIRS systems with low update frequency is possible using ARIMA-based methods, yielding reasonably small residuals. The windowed-point forecasting method closely predicted the measured NIRS signals with a high correlation and good agreement, followed by windowed-interval forecasting in the healthy population (HC and SP) and a cranial trauma population (TBI) at the highest 10 s temporal resolution. In contrast, the anchored-point and anchored-interval methods were unsuccessful in consistently predicting physiologic signals with good precision, correlation, and agreement. The correlation and agreement between the predicted and actual values explained that the results using the sliding-window approach were better than the anchored approach due to the data partitioning and model memory related to each approach. The role of high-frequency research-grade NIRS platforms, with increased sampling rates, in point and interval ARIMA forecasting remains unclear. Also, the ability to forecast high-frequency NIRS physiological signals from brain lobes other than the frontal lobe requires evaluation in future studies and requires further evaluation in healthy and pathologic cohorts.

## Figures and Tables

**Table 1:** Demographic data for HC, SP, and TBI populations.

Variable	Median [IQR] or Count (%)
Healthy control (HC) Volunteers	
Duration of Recording (min)	31.1 (28.7–34.8)
Number of Patients	102.0
Age (years)	26.0 (22.2–31.0)
Biological Sex (Male)	42.0 (41.2%)
Hand Dominance (Right)	93.0 (91.2%)
Elective Spinal Surgery (SP) Patients	
Duration of Recording (min)	185.0 (165.8–206.8)
Number of Patients	27.0
Age (years)	57.0 (52.0–65.5)
Biological Sex (Male)	22.0 (81.5%)
Arterial pCO_2_ (mmHg)	42.0 (40.0–44.5)
Arterial pO_2_ (mmHg)	231.0 (211.5–295.0)
Traumatic Brain Injury (TBI) Patients	
Duration of Recordings (min)	4644.0 (2369.3–7942.3)
Number of Patients	101.0
Age (years)	42.0 (28.0–57.0)
Biological Sex (Male)	81.0 (80.2%)
GCS	6.0 (4.0–8.0)
GCS Motor	4.0 (2.0–5.0)
Hypoxia (Yes)	29.0 (28.7%)
Hypotension (Yes)	10.0 (9.9%)
Arterial pCO_2_ (mmHg)	37.0 (35.1–39.0)
Arterial pO_2_ (mmHg)	108.0 (95.5–127.5)
Focal Injury (Contusion, EDH, SDH, or aSDH)	75.0 (74.3%)
Diffuse Injury (DAI or tSAH)	26.0 (25.7%)

The table depicts the summary of admission demographic data for all three populations (HC, SP, and TBI), as the type of injury in each population differed, along with their drug intervention. DAI, diffuse axonal injury; EDH, epidural hematoma; GCS, Glasgow coma score; HC, healthy control, IQR, interquartile range; pCO_2_, partial pressure of carbon dioxide; pO_2_, partial pressure of oxygen; tSAH, traumatic subarachnoid hemorrhage; SDH, subdural hematoma; aSDH, acute subdural hematoma; SP, elective spinal surgery; TBI, traumatic brain injury.

**Table 2:** ADF and KPSS results showing stationary vs. non-stationary vs. NA for calculated CA indices—original and 1st order differenced HC, SP, and TBI Data.

ADF Results for Non-Differenced Data													
Dataset	Time Resolution	COx_L			COx R			COx-a_L			COx-a_R		
		S	NS	NA	S	NS	NA	S	NS	NA	S	NS	NA
HC	10 s	–	–	–	–	–	–	9	93	0	5	97	0
	1 min	–	–	–	–	–	–	27	75	0	28	74	0
	5 min	–	–	–	–	–	–	5	17	80	4	18	80
SP	10 s	–	–	–	–	–	–	25	2	0	22	5	0
	1 min	–	–	–	–	–	–	27	0	0	27	0	0
	5 min	–	–	–	–	–	–	16	11	0	14	13	0
TBI	10 s	96	2	3	93	1	7	97	1	3	94	0	7
	1 min	97	1	3	93	1	7	98	0	3	94	0	7
	5 min	94	2	5	91	2	8	97	0	4	94	0	7
**ADF results for 1st-order differenced data**													
**Dataset**	**Time Resolution**	**COx_L**			**COx_R**			**COx-a_L**			**COx-a_R**		
		**S**	**NS**	**NA**	**S**	**NS**	**NA**	**S**	**NS**	**NA**	**S**	**NS**	**NA**
HC	10 s	–	–	–	–	–	–	102	0	0	102	0	0
	1 min	–	–	–	–	–	–	65	37	0	66	36	0
	5 min	–	–	–	–	–	–	1	6	95	1	6	95
SP	10 s	–	–	–	–	–	–	27	0	0	27	0	0
	1 min	–	–	–	–	–	–	27	0	0	27	0	0
	5 min	–	–	–	–	–	–	25	1	1	27	0	0
TBI	10 s	98	0	3	94	0	7	98	0	3	94	0	7
	1 min	96	2	3	93	1	7	98	0	3	94	0	7
	5 min	96	0	5	93	0	8	97	0	4	94	0	7
**KPSS results for non-differenced data**													
**Dataset**	**Time Resolution**	**COx_L**			**COx_R**			**COx-a_L**			**COx-a_R**		
		**S**	**NS**	**NA**	**S**	**NS**	**NA**	**S**	**NS**	**NA**	**S**	**NS**	**NA**
HC	10 s	–	–	–	–	–	–	39	63	0	47	55	0
	1 min	–	–	–	–	–	–	81	21	0	87	15	0
	5 min	–	–	–	–	–	–	102	0	0	102	0	0
SP	10 s	–	–	–	–	–	–	12	15	0	9	18	0
	1 min	–	–	–	–	–	–	25	2	0	23	4	0
	5 min	–	–	–	–	–	–	26	1	0	25	2	0
TBI	10 s	16	82	3	6	88	7	15	83	3	11	83	7
	1 min	33	65	3	22	72	7	35	63	3	29	65	7
	5 min	43	55	3	35	59	7	48	50	3	42	52	7
**KPSS results for 1st-order differenced data**													
**Dataset**	**Time Resolution**	**COx_L**			**COx_R**			**COx-a_L**			**COx-a_R**		
		**S**	**NS**	**NA**	**S**	**NS**	**NA**	**S**	**NS**	**NA**	**S**	**NS**	**NA**
HC	10 s	–	–	–	–	–	–	101	1	0	102	0	0
	1 min	–	–	–	–	–	–	102	0	0	101	1	0
	5 min	–	–	–	–	–	–	98	4	0	99	3	0
SP	10 s	–	–	–	–	–	–	27	0	0	27	0	0
	1 min	–	–	–	–	–	–	27	0	0	27	0	0
	5 min	–	–	–	–	–	–	27	0	0	27	0	0
TBI	10 s	98	0	3	94	0	7	98	0	3	94	0	7
	1 min	98	0	3	94	0	7	98	0	3	94	0	7
	5 min	98	0	3	94	0	7	98	0	3	94	0	7

The table presents the results of the ADF and KPSS analysis with the count of patient data that was found to be stationary (S), non-stationary (NS), or unable to assess (NA) using non-differenced and 1st-order differenced data for all populations. It was found from these stationarity tests that signals were stationary after 1st order differencing while they were originally non-stationary. ADF, Augmented Dickey–Fuller; COx, cerebral oximetry index with cerebral perfusion pressure; COx-a, cerebral oximetry index with arterial blood pressure; HC, healthy control volunteer group; NA, unable to assess stationarity; NS, non-stationary; S, stationary; SP, elective spinal surgery patient group; TBI, traumatic brain injury patient group.

**Table 3:** Median and IQR of optimal ARIMA models based on AIC using 10 s, 1 min, and 5 min temporal resolutions in all populations.

Population	Optimal ARIMA Models (Median [IQR])							
	ABP	CPP	rSO_2__L	rSO_2__R	COx_L	COx_R	COx-a_L	COx-a_R
10 s Temporal Resolution								
HC	(2,1,3) [(1,1,2)–(4,1,3)]	–	(2,1,4) [(1,1,3)–(5,1,4)]	(2,1,7) [(1,1,3)–(5,1,5)]	–	–	(3,1,5) [(2,1,0)–(5,1,6)]	(3,1,10) [(1,1,1)–(5,1,4)]
SP	(8,1,5) [(3,1,8)–(9,1,10)]	–	(6,1,2) [(3,1,7)–(8,1,10)]	(7,1,9) [(4,1,9)–(9,1,10)]	–	–	(7,1,2) [(4,1,10)–(8,1,10)]	(7,1,4) [(5,1,3)–(9,1,6)]
TBI	(9,1,9) [(7,1,9)–(10,1,7)]	(9,1,7) [(6,1,8)–(10,1,8)]	(8,1,10) [(6,1,4)–(10,1,9)]	(8,1,10) [(6,1,5)–(10,1,8)]	(9,1,6) [(7,1,8)–(10,1,5)]	(8,1,10) [(7,1,7)–(10,1,8)]	(8,1,10) [(7,1,10)–(9,1,10)]	(9,1,5) [(7,1,7)–(10,1,5)]
1 min Temporal Resolution								
HC	(1,1,2) [(1,1,1)–(3,1,0)]	–	(1,1,3) [(1,1,1)–(2,1,6)]	(1,1,4) [(1,1,1)–(3,1,2)]	–	–	(2,1,1) [(1,1,3)–(4,1,1)]	(2,1,1) [(1,1,2)–(4,1,1)]
SP	(4,1,2) [(2,1,3)–(6,1,10)]	–	(3,1,2) [(1,1,9)–(5,1,3)]	(3,1,4) [(2,1,2)–(6,1,10)]	–	–	(3,1,5) [(2,1,6)–(5,1,5)]	(2,1,10) [(1,1,8)–(4,1,5)]
TBI	(6,1,1) [(3,1,1)–(8,1,8)]	(6,1,5) [(2,1,2)–(9,1,4)]	(6,1,10) [(4,1,1)–(9,1,10)]	(6,1,7) [(3,1,10)–(9,1,10)]	(4,1,9) [(2,1,10)–(7,1,1)]	(4,1,9) [(2,1,2)–(8,1,4)]	(4,1,3) [(2,1,8)–(7,1,4)]	(5,1,4) [(3,1,1)–(7,1,10)]
5 min Temporal Resolution								
HC	(4,1,0) [(3,1,1)–(4,1,9)]	–	(4,1,1) [(3,1,2)–(4,1,9)]	(4,1,1) [(3,1,2)–(4,1,7)]	–	–	(4,1,0) [(3,1,0)–(4,1,8)]	(4,1,1) [(3,1,1)–(4,1,9)]
SP	(1,1,5) [(1,1,1)–(3,1,1)]	–	(1,1,5) [(1,1,1)–(5,1,0)]	(2,1,0) [(1,1,0)–(5,1,0)]	–	–	(1,1,1) [(1,1,1)–(2,1,2)]	(2,1,4) [(1,1,1)–(4,1,3)]
TBI	(4,1,5) [(2,1,5)–(7,1,6)]	(4,1,7) [(3,1,1)–(7,1,4)]	(5,1,7) [(2,1,9)–(8,1,9)]	(4,1,5) [(2,1,1)–(7,1,10)]	(3,1,3) [(1,1,3)–(5,1,2)]	(3,1,1) [(1,1,2)–(6,1,5)]	(3,1,3) [(1,1,3)–(6,1,6)]	(3,1,5) [(2,1,4)–(7,1,1)]

The table represents the median and IQR of optimal ARIMA models, selected using lowest AIC criterion, for each signal in all populations. The autoregressive structure in HC population tended to be lower and higher in TBI population, while the autoregressive structure in a population was found to be similar. ABP, arterial blood pressure; AIC, Akaike information criterion; ARIMA, autoregressive integrative moving average; COx, cerebral oximetry index with CPP; COx-a, cerebral oximetry index with ABP; CPP, cerebral perfusion pressure; HC, healthy control volunteer group; IQR, interquartile range; rSO_2_, regional cerebral oxygen saturation; SP, elective spinal surgery patient group; TBI, traumatic brain injury patient group.

**Table 4:** Median and IQR of computational duration of forecast in all populations using 10 s temporal resolution.

Forecast Method	Window/Interval	Computational Duration in Minutes (Median [IQR])		
		HC	SP	TBI
Anchored Point	–	0 [0–0]	0 [0–0]	2 [1–5]
Anchored Interval	5 min	0 [0–0]	1 [0.5–2]	535 [87–3101]
	10 min	0 [0–0]	0 [0–1]	386 [66–1860]
	15 min	–	1 [0–1]	218 [45–1171]
	30 min	–	0 [0–1]	108 [18–495]
	1 h	–	1 [1–1]	69 [12–317]
	2 h	–	–	34 [7–169]
	6 h	–	–	27 [7–66]
	12 h	–	–	26.5 [10.25–45.75]
	1 day	–	–	31.5 [19.5–53.5]
Windowed Point	5 min	1 [0–1]	12 [7–17.5]	453 [165–992]
	10 min	0 [0–1]	15 [7.5–21]	596 [211–1313]
	15 min	0 [0–1]	18 [9.5–24]	748 [258–1649]
	30 min	0 [0–0]	27 [12.5–33]	1161 [372–2538]
	1 h	–	33 [19.5–46]	1973 [652–4307]
	2 h	–	25 [10–54]	3291 [1100–7580]
	6 h	–	67 [56.5–77.5]	2004 [1177–5817]
	12 h	–	–	1771 [1172–7788]
	1 day		–	10,357 [5870–16,993]
Windowed Interval	5 min	0 [0–0]	1 [0–1]	33 [13–77]
	10 min	0 [0–0]	0 [0–1]	21 [8–49]
	15 min	0 [0–0]	0 [0–0.5]	17 [7–40]
	30 min	–	0 [0–0]	13 [5–30]
	1 h	–	0 [0–0]	11 [4–25]
	2 h	–	0 [0–0]	10 [4–22]
	6 h	–	0 [0–0]	8 [3–19.5]
	12 h	–	–	7 [3–17]
	1 day	–	–	9 [3.5–21]

The table shows the median and IQR of computational duration of each method of point and interval forecasting in all populations using 10 s temporal resolution. Overall, the TBI data took the longest to forecast, and windowed point and anchored interval were the most computationally expensive methods. HC, healthy control volunteer group; IQR, interquartile range; SP, elective spinal surgery patient group; TBI, traumatic brain injury patient group.

**Table 5:** Anchored point—absolute forecast residual of rSO_2_ and COx/COx-a in all populations using 10 s temporal resolution.

Population	Median [IQR]					
	AFR of rSO_2__L	AFR of rSO_2__R	AFR of COx_L	AFR of COx_R	AFR of COx-a_L	AFR of COx-a_R
HC	0.88 [0.46–1.52]	0.91 [0.46–1.54]	–	–	0.19 [0.09–0.31]	0.17 [0.08–0.31]
SP	2.36 [0.94–4.26]	1.8 [0.94–3.64]	–	–	0.34 [0.15–0.54]	0.37 [0.17–0.61]
TBI	2.99 [1.37–4.73]	3.18 [1.64–5.25]	0.24 [0.11–0.41]	0.22 [0.11–0.4]	0.22 [0.1–0.39]	0.22 [0.1–0.38]

The table shows the median and IQR of AFR results of rSO_2_ and COx/COx-a signals forecasted using anchored-point method for all populations at 10 s temporal resolution. AFR, absolute forecast residual; COx, cerebral oximetry index with cerebral perfusion pressure; COx-a, cerebral oximetry index with arterial blood pressure; HC, healthy control volunteer group; IQR, interquartile range; rSO_2_, regional cerebral oxygen saturation; SP, elective spinal surgery patient group; TBI, traumatic brain injury patient group.

**Table 6:** Anchored interval—absolute forecast residual of rSO_2_ and COx/COx-a in all populations using 10 s temporal resolution.

Interval	Median [IQR]					
	AFR of rSO_2__L	AFR of rSO_2__R	AFR of COx_L	AFR of COx_R	AFR of COx-a_L	AFR of COx-a_R
HC Population						
5 min	0.82 [0.44–1.35]	0.88 [0.44–1.48]	–	–	0.17 [0.07–0.28]	0.14 [0.07–0.28]
10 min	1.41 [0.95–1.94]	1.55 [0.71–2.49]	–	–	0.08 [0.04–0.26]	0.13 [0.07–0.22]
SP Population						
5 min	0.8 [0.23–1.44]	0.83 [0.27–1.62]	–	–	0.2 [0.09–0.39]	0.25 [0.1–0.47]
10 min	1.03 [0.39–2.09]	1.02 [0.37–2.28]	–	–	0.26 [0.11–0.47]	0.29 [0.13–0.55]
15 min	1.31 [0.56–2.57]	1.24 [0.49–3.06]	–	–	0.25 [0.11–0.49]	0.31 [0.15–0.56]
30 min	1.41 [0.83–4.27]	1.44 [0.65–3.38]	–	–	0.28 [0.14–0.51]	0.32 [0.16–0.53]
1 h	1.8 [0.85–3.01]	1.35 [0.64–2.25]	–	–	0.23 [0.09–0.43]	0.28 [0.12–0.48]
TBI Population						
5 min	0.57 [0.23–1.06]	0.51 [0.19–1.05]	0.16 [0.07–0.3]	0.16 [0.07–0.3]	0.15 [0.06–0.3]	0.15 [0.06–0.29]
10 min	0.73 [0.29–1.43]	0.68 [0.26–1.31]	0.19 [0.08–0.35]	0.19 [0.08–0.34]	0.18 [0.08–0.33]	0.18 [0.08–0.33]
15 min	0.81 [0.35–1.63]	0.77 [0.33–1.6]	0.2 [0.09–0.36]	0.2 [0.09–0.36]	0.2 [0.09–0.35]	0.19 [0.08–0.35]
30 min	1.03 [0.45–2.14]	1.02 [0.42–2.15]	0.21 [0.1–0.38]	0.21 [0.1–0.37]	0.21 [0.09–0.37]	0.2 [0.09–0.36]
1 h	1.33 [0.57–2.66]	1.2 [0.51–2.82]	0.22 [0.1–0.38]	0.22 [0.1–0.38]	0.21 [0.1–0.36]	0.21 [0.1–0.37]
2 h	1.64 [0.72–3.29]	1.84 [0.78–3.71]	0.23 [0.1–0.39]	0.22 [0.1–0.39]	0.21 [0.1–0.37]	0.21 [0.1–0.37]
6 h	2.13 [1–4.19]	2.53 [1.09–4.65]	0.23 [0.11–0.4]	0.23 [0.11–0.4]	0.22 [0.1–0.38]	0.22 [0.1–0.38]
12 h	2.76 [1.36–4.77]	3.05 [1.33–5.24]	0.24 [0.12–0.4]	0.23 [0.11–0.4]	0.23 [0.11–0.39]	0.22 [0.1–0.37]
1 Day	3.23 [1.52–6.09]	3.76 [1.68–5.83]	0.23 [0.11–0.41]	0.25 [0.12–0.44]	0.2 [0.09–0.37]	0.22 [0.1–0.37]

The table shows the median and IQR of AFR results of rSO_2_ and COx/COx-a signals forecasted using anchored-interval method for all populations at 10 s temporal resolution. AFR, absolute forecast residual; COx, cerebral oximetry index with cerebral perfusion pressure; COx-a, cerebral oximetry index with arterial blood pressure; HC, healthy control volunteer group; IQR, interquartile range; rSO_2_, regional cerebral oxygen saturation; SP, elective spinal surgery patient group; TBI, traumatic brain injury patient group.

**Table 7:** Windowed point—absolute forecast residual of rSO_2_ and COx/COx-a in all populations using 10 s temporal resolution.

Window	Median [IQR]					
	AFR of rSO_2__L	AFR of rSO_2__R	AFR of COx_L	AFR of COx_R	AFR of COx-a_L	AFR of COx-a_R
HC Population						
5 min	0.52 [0.24–0.93]	0.55 [0.27–0.97]	–	–	0.04 [0.02–0.06]	0.04 [0.02–0.07]
10 min	0.48 [0.23–0.82]	0.53 [0.25–0.92]	–	–	0.03 [0.01–0.06]	0.03 [0.01–0.06]
15 min	0.46 [0.22–0.8]	0.51 [0.24–0.88]	–	–	0.03 [0.01–0.06]	0.03 [0.01–0.06]
30 min	0.51 [0.29–0.79]	0.5 [0.26–0.79]	–	–	0.03 [0.01–0.05]	0.03 [0.01–0.05]
SP Population						
5 min	0.27 [0.11–0.54]	0.29 [0.13–0.56]	–	–	0.04 [0.02–0.08]	0.04 [0.02–0.08]
10 min	0.24 [0.1–0.5]	0.26 [0.11–0.51]	–	–	0.04 [0.02–0.07]	0.04 [0.02–0.07]
15 min	0.23 [0.09–0.49]	0.23 [0.11–0.51]	–	–	0.03 [0.01–0.06]	0.03 [0.01–0.06]
30 min	0.22 [0.09–0.49]	0.23 [0.1–0.47]	–	–	0.03 [0.01–0.06]	0.03 [0.01–0.06]
1 h	0.21 [0.06–0.45]	0.23 [0.09–0.47]	–	–	0.03 [0.01–0.05]	0.03 [0.01–0.05]
2 h	0.19 [0.06–0.51]	0.21 [0.08–0.48]	–	–	0.03 [0.01–0.05]	0.03 [0.01–0.05]
6 h	0.22 [0.04–0.57]	0.13 [0.03–0.35]	–	–	0.03 [0.01–0.05]	0.03 [0.01–0.05]
TBI Population						
5 min	0.31 [0.13–0.61]	0.3 [0.12–0.58]	0.05 [0.02–0.09]	0.05 [0.02–0.09]	0.05 [0.02–0.09]	0.05 [0.02–0.09]
10 min	0.27 [0.1–0.54]	0.26 [0.1–0.53]	0.04 [0.02–0.07]	0.04 [0.02–0.07]	0.04 [0.02–0.07]	0.04 [0.02–0.07]
15 min	0.25 [0.1–0.52]	0.24 [0.09–0.5]	0.04 [0.02–0.07]	0.04 [0.02–0.07]	0.04 [0.02–0.07]	0.04 [0.02–0.07]
30 min	0.23 [0.08–0.5]	0.22 [0.08–0.48]	0.03 [0.01–0.06]	0.03 [0.01–0.06]	0.03 [0.01–0.06]	0.03 [0.01–0.06]
1 h	0.23 [0.07–0.5]	0.21 [0.07–0.48]	0.03 [0.01–0.06]	0.03 [0.01–0.06]	0.03 [0.01–0.06]	0.03 [0.01–0.06]
2 h	0.22 [0.06–0.5]	0.2 [0.06–0.48]	0.03 [0.01–0.06]	0.03 [0.01–0.06]	0.03 [0.01–0.06]	0.03 [0.01–0.06]
6 h	0.3 [0.1–0.6]	0.27 [0.11–0.51]	0.03 [0.01–0.06]	0.03 [0.01–0.06]	0.03 [0.01–0.06]	0.03 [0.01–0.06]
12 h	0.32 [0.1–0.6]	0.26 [0.09–0.52]	0.03 [0.01–0.06]	0.03 [0.01–0.06]	0.03 [0.01–0.06]	0.03 [0.01–0.06]
1 Day	0.36 [0.13–0.68]	0.22 [0.06–0.51]	0.03 [0.01–0.05]	0.03 [0.01–0.06]	0.04 [0.02–0.07]	0.04 [0.02–0.07]

The table shows the median and IQR of AFR results of rSO_2_ and COx/COx-a signals forecasted using windowed-point method for all populations at 10 s temporal resolution. AFR, absolute forecast residual; COx, cerebral oximetry index with cerebral perfusion pressure; COx-a, cerebral oximetry index with arterial blood pressure; HC, healthy control volunteer group; IQR, interquartile range; rSO_2_, regional cerebral oxygen saturation; SP, elective spinal surgery patient group; TBI, traumatic brain injury patient group.

**Table 8:** Windowed interval—absolute forecast residual of rSO_2_ and COx/COx-a in all populations using 10 s temporal resolution.

Window and Interval	Median [IQR]					
	AFR of rSO_2__L	AFR of rSO_2__R	AFR of COx_L	AFR of COx_R	AFR of COx-a_L	AFR of COx-a_R
HC Population						
5 min	0.99 [0.47–1.69]	1.09 [0.51–1.87]	–	–	0.18 [0.07–0.35]	0.2 [0.08–0.37]
10 min	1.07 [0.5–1.81]	1.12 [0.52–1.97]	–	–	0.21 [0.09–0.38]	0.2 [0.09–0.39]
15 min	1.17 [0.59–1.97]	1.21 [0.58–2]	–	–	0.23 [0.11–0.41]	0.26 [0.12–0.4]
SP Population						
5 min	0.75 [0.27–1.47]	0.7 [0.27–1.39]	–	–	0.27 [0.11–0.51]	0.28 [0.11–0.58]
10 min	0.99 [0.39–2.18]	0.85 [0.34–1.87]	–	–	0.31 [0.12–0.6]	0.3 [0.12–0.56]
15 min	1.02 [0.46–2.43]	1.02 [0.48–2.52]	–	–	0.33 [0.14–0.6]	0.34 [0.14–0.62]
30 min	1.82 [0.71–3.69]	1.4 [0.74–4]	–	–	0.33 [0.14–0.59]	0.33 [0.15–0.57]
1 h	1.96 [0.93–4.12]	1.72 [0.92–3.08]	–	–	0.31 [0.16–0.54]	0.31 [0.15–0.53]
2 h	1.77 [0.92–2.94]	1.58 [0.78–2.72]	–	–	0.28 [0.12–0.42]	0.28 [0.14–0.52]
TBI Population						
5 min	0.54 [0.21–1.09]	0.48 [0.17–1]	0.21 [0.09–0.42]	0.2 [0.09–0.41]	0.2 [0.09–0.4]	0.2 [0.09–0.4]
10 min	0.63 [0.26–1.29]	0.55 [0.2–1.18]	0.24 [0.1–0.45]	0.23 [0.1–0.44]	0.23 [0.1–0.43]	0.23 [0.1–0.44]
15 min	0.71 [0.27–1.44]	0.68 [0.23–1.37]	0.24 [0.11–0.46]	0.24 [0.11–0.46]	0.24 [0.1–0.44]	0.24 [0.1–0.46]
30 min	0.93 [0.36–1.93]	0.91 [0.31–1.9]	0.26 [0.12–0.47]	0.25 [0.11–0.46]	0.24 [0.11–0.45]	0.25 [0.11–0.45]
1 h	1.18 [0.51–2.61]	1.11 [0.46–2.42]	0.26 [0.12–0.46]	0.25 [0.12–0.46]	0.25 [0.11–0.44]	0.25 [0.11–0.44]
2 h	1.7 [0.71–3.27]	1.61 [0.67–3.14]	0.25 [0.12–0.44]	0.25 [0.11–0.44]	0.25 [0.11–0.44]	0.24 [0.11–0.43]
6 h	2.36 [1.04–4.09]	2.27 [1–4.61]	0.24 [0.11–0.43]	0.24 [0.11–0.42]	0.23 [0.11–0.41]	0.23 [0.11–0.4]
12 h	2.92 [1.23–5.2]	3.12 [1.31–5.93]	0.24 [0.11–0.42]	0.24 [0.11–0.42]	0.23 [0.1–0.39]	0.22 [0.1–0.39]
1 Day	3.49 [1.5–6.18]	4.08 [1.72–7.08]	0.24 [0.11–0.42]	0.24 [0.11–0.42]	0.24 [0.1–0.44]	0.24 [0.1–0.46]

The table shows the median and IQR of AFR results of rSO_2_ and COx/COx-a signals forecasted using windowed-interval method for all populations at 10 s temporal resolution. AFR, absolute forecast residual; COx, cerebral oximetry index with cerebral perfusion pressure; COx-a, cerebral oximetry index with arterial blood pressure; HC, healthy control volunteer group; IQR, interquartile range; rSO_2_, regional cerebral oxygen saturation; SP, elective spinal surgery patient group; TBI, traumatic brain injury patient group.

## Data Availability

Research ethics board approval at our institution does not facilitate the free and open sharing of human data, regardless of the data being in a de-identified fashion. All such data are protected under both ethics and privacy acts within the Province of Manitoba, preventing such open sharing of data. All the data analyzed and used are available from the corresponding author upon reasonable request.

## Supplementary Materials

The following supporting information can be downloaded at https://www.mdpi.com/article/10.3390/bioengineering12070682/s1: File S1: Methodology Appendix [27,30,35,36,37]; File S2: Time-Series Stationarity Analysis; File S3: Autoregressive Integrative Moving Average (ARIMA) Analysis; File S4: Computational Duration Analysis; File S5: Absolute Forecast Residual Analysis; File S6: Root Mean Squared Error Analysis; File S7: Pearson Correlation Analysis; File S8: Bland-Altman Agreement Analysis.

## Abbreviations

The following abbreviations are used in this manuscript:

ABP	Arterial blood pressure
ADF	Augmented Dickey–Fuller
AFR	Absolute forecast residual
AIC	Akaike information criterion
Anchored-Interval	Anchored forecasting using interval approach
Anchored-Point	Anchored forecasting using point approach
ARIMA	Autoregressive integrative moving average
BTF	Brain Trauma Foundation
CA	Cerebral autoregulation
CBF	Cerebral blood flow
CBv	Cerebral blood volume
COx	Cerebral oximetry index with CPP
COx_L	Cerebral oximetry index with CPP of left frontal lobe
COx_R	Cerebral oximetry index with CPP of right frontal lobe
COx-a	Cerebral oximetry index with ABP
COx-a_L	Cerebral oximetry index with ABP of left frontal lobe
COx-a_R	Cerebral oximetry index with ABP of right frontal lobe
CPP	Cerebral perfusion pressure
CT	Computed tomography
CVR	Cerebrovascular reactivity
d-order	Moving average order
GCS	Glasgow coma scale
HC	Healthy volunteers
ICM+	Intensive care monitoring “Plus”
ICP	Intracranial pressure
ICU	Intensive care unit
IQR	Interquartile range
KB	Kilobyte
KPSS	Kwiatkowski–Phillips–Schmidt–Shin
LoA	Limit of agreement
MAIN-HUB	Multi-Omic analytics and integrative neuroinformatics in the human brain
MAD	Median absolute deviation
NA	Represents missing value or undetermined value due to inadequate data
NIRS	Near-infrared spectroscopy
pCO_2_	Partial pressure of carbon dioxide
pO_2_	Partial pressure of oxygen
p-order	Autoregressive order
PRx	Pressure reactivity index
q-order	Moving average order
*R*	Pearson correlation coefficient
relative bias	Bias as proportion of LoA spread
RMSE	Root mean squared error
rSO_2_	Regional cerebral oxygen saturation
rSO_2__L	Regional cerebral oxygen saturation of left frontal lobe
rSO_2__R	Regional cerebral oxygen saturation of the right frontal lobe
SP	Elective spinal surgery patients
TBI	Traumatic brain injury
Windowed-Interval	Sliding-window forecasting using interval approach
Windowed-Point	Sliding-window forecasting using point approach
