# Supplementary material for: Time-Series Autoregressive Models for Point and Interval Forecasting of Raw and Derived Commercial Near-Infrared Spectroscopy Measures: An Exploratory Cranial Trauma and Healthy Control Analysis
Source: Bioengineering (Basel). 2025 Jun 21;12(7):682. doi: 10.3390/bioengineering12070682 (PMC12292983; doi:10.3390/bioengineering12070682)
Supplement: Supplementary file 1 [file bioengineering-12-00682-s001.zip › File S1.pdf]

File S1 – Methodology Appendix

File S1 – Table of Contents

File S1a: Evaluation of Data Stationarity 2

File S1b: Evaluation of optimal Autoregressive Integrated Moving Average (ARIMA) orders 3

File S1c: Additional Injury Information for SP and TBI Populations 4

File S1d: References 5

File S1a: Evaluation of Data Stationarity

Stationarity analysis was performed for each physiologic signal at an individual level of each population using Augmented Dickey-Fuller (ADF) and Kwiatkowski-Phillips-Schmidt-Shin (KPSS) tests for the 10-second, 1-minute, and 5-minute temporal resolutions, in keeping with previous work from our group. The ADF test informs if the series is trend-stationary and KPSS test informs if the series is stationary around a linear trend [1]. The ADF and KPSS tests were run on each patient data using the "adf.test" and "kpss.test" functions from the *tseries* package (https://cran.r-project.org/web/packages/tseries/index.html) with an example of patient results shown for each dataset given in Appendices B1-B3.

The data from populations were 1st order differenced, and the above-mentioned stationarity analysis was re-run on each differenced data in the 10-second, 1-minute, and 5-minute temporal resolutions, with an example of patient results for each dataset shown in Appendices B1-B3. It is important to note that for the lower temporal resolutions (1-minute and 5-minute), the differencing was performed after the resolution was reduced because differencing at the native resolution, of 10-second temporal resolution, before resolution reduction does not address the trend in the data as shown previously from our lab [2].

File S1b: Evaluation of optimal Autoregressive Integrated Moving Average (ARIMA) orders

Using R statistical software, Autoregressive Integrated Moving Average (ARIMA) model fit was performed in accordance with standard Box-Jenkin's time-series methodologies [1,3,4]. Various ARIMA models were fit to each univariate physiologic time-series to model every 1st order differenced signal for each patient in 10-second, 1-minute, and 5-minute temporal resolutions. The Akaike Information Criterion (AIC) value was obtained for ARIMA model fit on all physiologic variables (left and right regional oxygen saturation [rSO_2_L and rSO_2_R, respectively], left and right cerebral oximetry index derived with cerebral perfusion pressure [COx_L and COx_R, respectively], and left and right cerebral oximetry index derived with arterial blood pressure [COx-a_L and COx-a_R, respectively) where available since it is neither stringent or lenient as compared to other performance values [2,5]. Based on previous research from our lab [2], the list of ARIMA models were obtained by varying the autoregressive order (p-order), and the moving average order (q-order) from 1 to 10, and 0 to 10, respectively. The integrative order (d-order) was separately varied from 0 to 1 using the previously described differencing method. An example of these outputs is shown in Appendix C1. To find the optimal ARIMA model for each physiologic time-series in various temporal resolutions, the lowest AIC values were extracted for a patient. Finally, by reordering the optimal ARIMA models sequentially, the median optimal ARIMA model for a physiologic signal in each temporal resolution was found using AIC and the 10-second resolution example for all populations is given in Appendix C2.

File S1c: Additional Injury Information for SP and TBI Populations

Variable	Median (IQR) or Number (%)
**Elective Spinal Surgery (SP) Patients**	
**Procedure Type**	
ACDF	60.0 (22.2%)
PCDF	14.0 (51.9%)
ACDF and PCDF	3.0 (11.1%)
Cervical Incision and Drain	1.0 (3.7%)
Corpectomy	1.0 (3.7%)
Laminectomy	1.0 (3.7%)
Thoracic Decompression and Instrumental Fusion	1.0 (3.7%)
**Anesthetic Regimen**	
Propofol + Sufentanil	6.0 (22.2%)
Ketamine + Propofol + Sufentanil	6.0 (22.2%)
Midazolam + Propofol + Remi-Fentanyl	1.0 (3.7%)
Midazolam + Propofol + Sufentanil	4.0 (14.8%)
Propofol + Remi-Fentanyl + Sufentanil	2.0 (7.4%)
Ketamine + Midazolam + Propofol + Sufentanil	7.0 (25.9%)
Ketamine + Midazolam + Propofol + Remi-Fentanyl + Sufentanil	1.0 (3.7%)
**Traumatic Brain Injury Patients (TBI)**	
**Pupils**	
Bilateral Unreactive	15.0 (14.9%)
Unilateral Unreactive	22.0 (21.8%)
Bilateral Reactive	64.0 (63.4%)
**Marshall CT Score**	
V	51.0 (50.5%)
IV	18.0 (17.8%)
III	29.0 (28.7%)
II	3.0 (3.0%)
**Rotterdam CT Score**	
6	24.0 (23.8%)
5	25.0 (24.8%)
4	29.0 (28.7%)
3	16.0 (15.8%)
2	6.0 (5.9%)
1	1.0 (1.0%)
**Anesthetic Regimen**	
None	3.0 (3.0%)
Propofol	24.0 (23.8%)
Fentanyl + Propofol	36.0 (35.6%)
Ketamine + Propofol	2.0 (2.0%)
Midazolam + Propofol	1.0 (1.0%)
Fentanyl + Ketamine + Propofol	6.0 (5.9%)
Fentanyl + Midazolam + Propofol	22.0 (21.8%)
Fentanyl + Ketamine + Midazolam + Propofol	7.0 (6.9%)

ACDF, anterior cervical discectomy and fusion; CT, computed tomography; DAI, diffuse axonal injury; EDH, epidural hematoma; GCS, Glasgow Coma Score; IQR, interquartile range; PCDF, posterior cervical discectomy and fusion.

File S1d: References

1. Chatfield, C.; Xing, H. *The Analysis of Time Series: An Introduction with R*; 7th ed.; Chapman and Hall/CRC, 2019; ISBN 978-1-138-06613-7.
2. Sainbhi, A.S.; Vakitbilir, N.; Gomez, A.; Stein, K.Y.; Froese, L.; Zeiler, F.A. Time-Series Autocorrelative Structure of Cerebrovascular Reactivity Metrics in Severe Neural Injury: An Evaluation of the Impact of Data Resolution. *Biomedical Signal Processing and Control* **2024**, *95*, 106403, doi:10.1016/j.bspc.2024.106403.
3. Chatfield, C. *The Analysis of Time Series: An Introduction*; 6th ed.; Chapman and Hall/CRC: New York, 2003; ISBN 978-0-429-20870-6.
4. Lütkepohl, H. *New Introduction to Multiple Time Series Analysis*; New York : Springer: Berlin, 2005; ISBN 978-3-540-40172-8.
5. Sainbhi, A.S.; Froese, L.; Stein, K.Y.; Vakitbilir, N.; Gomez, A.; Islam, A.; Bergmann, T.; Silvaggio, N.; Hayat, M.; Zeiler, F.A. Commercial NIRS May Not Detect Hemispheric Regional Disparity in Continuously Measured COx/COx-a: An Exploratory Healthy and Cranial Trauma Time-Series Analysis. *Bioengineering* **2025**, *12*, 247, doi:10.3390/bioengineering12030247.
