# Supplementary material for: Time-Series Autoregressive Models for Point and Interval Forecasting of Raw and Derived Commercial Near-Infrared Spectroscopy Measures: An Exploratory Cranial Trauma and Healthy Control Analysis
Source: Bioengineering (Basel). 2025 Jun 21;12(7):682. doi: 10.3390/bioengineering12070682 (PMC12292983; doi:10.3390/bioengineering12070682)
Supplement: Supplementary file 1 [file bioengineering-12-00682-s001.zip › File S2.pdf]

File S2 – Time-Series Stationarity Analysis

File S2 – Table of Contents

File S2a: ADF and KPSS P-Values for Non-Differenced and 1st Order Differenced in 1-Minute Data Resolution – HC Volunteers 2

File S2b: ADF and KPSS P-Values for Non-Differenced and 1st Order Differenced in 1-Minute Data Resolution – SP Patients 7

File S2c: ADF and KPSS P-Values for Non-Differenced and 1st Order Differenced in 1-Minute Data Resolution – TBI Patients 9

File S2d: ADF and KPSS results showing stationary vs non-stationary vs NA for physiologic signals – Original and 1st order differenced HC, SP, and TBI Data 12

File S2a: ADF and KPSS P-Values for Non-Differenced and 1st Order Differenced in 1-Minute Data Resolution – HC Volunteers

ADF p-values for 1-minute data resolution										
**Patient**	***Non-Differenced***					***1st Order Differenced***				
	**ABP**	**rSO_2_L**	**rSO_2_R**	**COx-a_L**	**COx-a_R**	**ABP**	**rSO_2_L**	**rSO_2_R**	**COx-a_L**	**COx-a_R**
1	0.08	0.45	0.37	0.34	0.47	0.01	0.01	0.01	0.09	0.19
2	0.59	0.50	0.24	0.09	0.36	0.02	0.01	0.01	0.03	0.04
3	0.37	0.71	0.70	0.40	0.60	0.01	0.04	0.01	0.01	0.31
4	0.44	0.01	0.09	0.01	0.01	0.02	0.01	0.01	0.01	0.01
5	0.57	0.84	0.35	0.56	0.90	0.07	0.02	0.01	0.02	0.01
6	0.17	0.40	0.03	0.58	0.82	0.09	0.01	0.01	0.21	0.23
7	0.06	0.06	0.25	0.02	0.01	0.02	0.01	0.01	0.01	0.01
8	0.43	0.05	0.10	0.01	0.01	0.05	0.21	0.01	0.01	0.01
9	0.01	0.27	0.37	0.01	0.43	0.01	0.02	0.01	0.01	0.06
10	0.48	0.22	0.13	0.44	0.31	0.19	0.02	0.02	0.22	0.25
11	0.15	0.26	0.39	0.22	0.43	0.01	0.01	0.04	0.02	0.01
12	0.01	0.46	0.52	0.39	0.40	0.01	0.01	0.01	0.04	0.01
13	0.63	0.17	0.02	0.21	0.33	0.01	0.01	0.01	0.02	0.08
14	0.34	0.96	0.54	0.06	0.02	0.01	0.01	0.01	0.09	0.03
15	0.63	0.34	0.37	0.01	0.01	0.01	0.01	0.01	0.01	0.01
16	0.36	0.64	0.40	0.12	0.01	0.13	0.18	0.01	0.06	0.15
17	0.89	0.19	0.46	0.30	0.10	0.02	0.01	0.01	0.02	0.03
18	0.43	0.21	0.01	0.29	0.24	0.01	0.01	0.01	0.01	0.02
19	0.30	0.62	0.89	0.06	0.30	0.01	0.01	0.01	0.06	0.09
20	0.02	0.50	0.47	0.34	0.26	0.01	0.02	0.09	0.01	0.03
21	0.43	0.03	0.03	0.12	0.17	0.01	0.01	0.01	0.01	0.01
22	0.02	0.01	0.05	0.95	0.85	0.01	0.01	0.01	0.01	0.06
23	0.15	0.54	0.24	0.27	0.54	0.01	0.01	0.01	0.03	0.01
24	0.60	0.48	0.01	0.48	0.84	0.10	0.34	0.01	0.26	0.40
25	0.89	0.52	0.49	0.31	0.47	0.01	0.01	0.01	0.01	0.01
26	0.45	0.47	0.17	0.08	0.01	0.03	0.01	0.15	0.01	0.01
27	0.70	0.01	0.02	0.01	0.01	0.01	0.01	0.01	0.01	0.01
28	0.21	0.43	0.13	0.04	0.17	0.01	0.07	0.05	0.07	0.04
29	0.73	0.39	0.03	0.25	0.10	0.05	0.01	0.05	0.07	0.23
30	0.24	0.21	0.07	0.02	0.04	0.01	0.01	0.01	0.02	0.03
31	0.21	0.08	0.06	0.04	0.02	0.01	0.01	0.01	0.01	0.01
32	0.01	0.02	0.19	0.45	0.46	0.01	0.01	0.01	0.24	0.10
33	0.01	0.66	0.25	0.37	0.58	0.01	0.01	0.01	0.01	0.09
34	0.34	0.46	0.52	0.39	0.56	0.09	0.13	0.12	0.43	0.29
35	0.52	0.63	0.46	0.01	0.07	0.01	0.01	0.02	0.01	0.01
36	0.34	0.59	0.12	0.01	0.24	0.02	0.02	0.01	0.01	0.06
37	0.98	0.41	0.48	0.01	0.03	0.05	0.02	0.02	0.32	0.37
38	0.02	0.13	0.32	0.19	0.10	0.01	0.01	0.01	0.04	0.04
39	0.08	0.57	0.01	0.37	0.01	0.01	0.01	0.01	0.09	0.01
40	0.08	0.72	0.07	0.51	0.63	0.01	0.01	0.01	0.04	0.25

41	0.09	0.54	0.04	0.01	0.04	0.01	0.05	0.01	0.01	0.01
42	0.01	0.06	0.60	0.10	0.10	0.01	0.01	0.01	0.08	0.01
43	0.32	0.02	0.08	0.47	0.07	0.01	0.01	0.29	0.58	0.37
44	0.46	0.46	0.01	0.01	0.01	0.01	0.01	0.01	0.07	0.03
45	0.26	0.36	0.47	0.25	0.56	0.01	0.01	0.01	0.03	0.02
46	0.59	0.22	0.01	0.05	0.11	0.21	0.09	0.01	0.04	0.30
47	0.95	0.26	0.29	0.27	0.14	0.01	0.01	0.01	0.01	0.04
48	0.77	0.01	0.20	0.02	0.04	0.01	0.01	0.01	0.02	0.04
49	0.62	0.07	0.48	0.20	0.02	0.02	0.01	0.01	0.02	0.01
50	0.01	0.14	0.62	0.03	0.36	0.01	0.01	0.01	0.04	0.01
51	0.02	0.04	0.26	0.01	0.17	0.01	0.01	0.01	0.01	0.01
52	0.05	0.03	0.04	0.09	0.58	0.01	0.01	0.01	0.19	0.48
53	0.98	0.06	0.95	0.70	0.31	0.02	0.01	0.02	0.03	0.02
54	0.29	0.18	0.17	0.20	0.75	0.01	0.03	0.01	0.27	0.34
55	0.05	0.12	0.80	0.45	0.03	0.01	0.01	0.16	0.09	0.01
56	0.30	0.22	0.17	0.25	0.23	0.01	0.01	0.01	0.01	0.10
57	0.53	0.68	0.22	0.01	0.04	0.05	0.01	0.01	0.02	0.01
58	0.07	0.77	0.01	0.91	0.17	0.01	0.05	0.32	0.06	0.02
59	0.03	0.59	0.11	0.61	0.71	0.01	0.57	0.07	0.31	0.67
60	0.44	0.99	0.82	0.10	0.40	0.01	0.06	0.02	0.05	0.03
61	0.43	0.01	0.01	0.03	0.19	0.01	0.01	0.01	0.02	0.01
62	0.18	0.03	0.55	0.28	0.08	0.01	0.01	0.01	0.34	0.02
63	0.02	0.59	0.66	0.22	0.20	0.01	0.02	0.43	0.05	0.02
64	0.08	0.21	0.13	0.35	0.49	0.01	0.10	0.06	0.25	0.04
65	0.26	0.22	0.46	0.03	0.01	0.01	0.01	0.01	0.01	0.01
66	0.20	0.36	0.55	0.57	0.66	0.02	0.17	0.10	0.04	0.09
67	0.10	0.03	0.30	0.42	0.11	0.01	0.01	0.01	0.02	0.01
68	0.38	0.20	0.49	0.06	0.07	0.03	0.04	0.01	0.01	0.04
69	0.63	0.38	0.44	0.68	0.25	0.02	0.01	0.01	0.01	0.01
70	0.60	0.47	0.12	0.49	0.26	0.01	0.01	0.01	0.03	0.09
71	0.10	0.36	0.72	0.52	0.59	0.01	0.01	0.01	0.10	0.06
72	0.01	0.74	0.05	0.30	0.27	0.01	0.01	0.02	0.06	0.01
73	0.48	0.26	0.28	0.05	0.09	0.01	0.01	0.03	0.02	0.02
74	0.31	0.18	0.16	0.26	0.15	0.01	0.01	0.01	0.01	0.01
75	0.10	0.01	0.04	0.02	0.09	0.01	0.01	0.01	0.01	0.01
76	0.35	0.92	0.92	0.05	0.04	0.01	0.02	0.05	0.04	0.18
77	0.08	0.01	0.04	0.37	0.27	0.02	0.01	0.01	0.31	0.01
78	0.53	0.90	0.44	0.06	0.05	0.01	0.09	0.01	0.04	0.24
79	0.35	0.89	0.82	0.04	0.34	0.03	0.01	0.01	0.01	0.01
80	0.60	0.44	0.43	0.03	0.02	0.02	0.01	0.04	0.05	0.03
81	0.46	0.89	0.62	0.14	0.04	0.05	0.92	0.83	0.03	0.01
82	0.01	0.39	0.47	0.92	0.58	0.01	0.04	0.01	0.02	0.40
83	0.57	0.34	0.17	0.24	0.29	0.02	0.01	0.01	0.05	0.03
84	0.31	0.77	0.46	0.10	0.08	0.01	0.01	0.01	0.02	0.01
85	0.42	0.11	0.04	0.39	0.48	0.04	0.01	0.01	0.22	0.01

86	0.47	0.86	0.99	0.30	0.36	0.01	0.01	0.08	0.50	0.49
87	0.05	0.74	0.54	0.42	0.17	0.01	0.01	0.01	0.21	0.06
88	0.07	0.43	0.04	0.77	0.32	0.10	0.02	0.02	0.02	0.01
89	0.45	0.30	0.15	0.30	0.20	0.01	0.01	0.01	0.10	0.09
90	0.01	0.01	0.04	0.50	0.02	0.01	0.01	0.01	0.30	0.02
91	0.64	0.28	0.31	0.08	0.06	0.01	0.01	0.02	0.01	0.01
92	0.68	0.27	0.11	0.10	0.07	0.02	0.01	0.01	0.01	0.01
93	0.08	0.22	0.19	0.40	0.02	0.01	0.01	0.01	0.01	0.04
94	0.21	0.47	0.08	0.08	0.24	0.01	0.01	0.01	0.06	0.18
95	0.59	0.93	0.95	0.34	0.01	0.01	0.04	0.07	0.08	0.01
96	0.42	0.18	0.08	0.04	0.03	0.03	0.01	0.01	0.01	0.03
97	0.48	0.05	0.02	0.01	0.01	0.01	0.01	0.01	0.01	0.01
98	0.60	0.60	0.79	0.39	0.27	0.02	0.01	0.01	0.38	0.40
99	0.28	0.07	0.01	0.48	0.43	0.01	0.01	0.01	0.01	0.13
100	0.15	0.13	0.46	0.33	0.26	0.01	0.01	0.05	0.07	0.17
101	0.27	0.18	0.42	0.18	0.56	0.01	0.04	0.01	0.01	0.03
102	0.01	0.33	0.04	0.15	0.03	0.01	0.01	0.01	0.01	0.01

KPSS p-values for 1-minute data resolution

**Patient**	***Non-Differenced***					***1st Order Differenced***				
	**ABP**	**rSO$_2$_L**	**rSO$_2$_R**	**COx-a_L**	**COx-a_R**	**ABP**	**rSO$_2$_L**	**rSO$_2$_R**	**COx-a_L**	**COx-a_R**
1	0.10	0.10	0.10	0.02	0.07	0.10	0.10	0.10	0.10	0.10
2	0.02	0.10	0.10	0.02	0.02	0.10	0.10	0.10	0.10	0.10
3	0.01	0.10	0.10	0.10	0.10	0.10	0.10	0.10	0.10	0.10
4	0.02	0.10	0.04	0.10	0.10	0.10	0.10	0.10	0.10	0.10
5	0.01	0.01	0.02	0.10	0.10	0.10	0.10	0.10	0.10	0.05
6	0.10	0.03	0.10	0.07	0.10	0.10	0.10	0.10	0.10	0.04
7	0.07	0.10	0.10	0.10	0.10	0.10	0.10	0.10	0.10	0.10
8	0.10	0.10	0.02	0.10	0.10	0.10	0.10	0.10	0.10	0.10
9	0.01	0.01	0.01	0.01	0.10	0.10	0.10	0.10	0.10	0.10
10	0.10	0.10	0.01	0.10	0.10	0.10	0.10	0.10	0.10	0.10
11	0.01	0.09	0.10	0.10	0.10	0.10	0.10	0.10	0.10	0.10
12	0.10	0.10	0.02	0.10	0.10	0.10	0.10	0.10	0.10	0.10
13	0.10	0.05	0.06	0.10	0.10	0.10	0.10	0.10	0.10	0.10
14	0.10	0.10	0.10	0.10	0.10	0.10	0.09	0.10	0.10	0.10
15	0.02	0.01	0.02	0.04	0.10	0.10	0.10	0.10	0.10	0.10
16	0.10	0.03	0.01	0.10	0.10	0.10	0.10	0.10	0.09	0.10
17	0.02	0.03	0.01	0.10	0.10	0.10	0.10	0.10	0.10	0.10
18	0.10	0.10	0.04	0.10	0.10	0.10	0.10	0.10	0.10	0.10
19	0.01	0.01	0.01	0.10	0.04	0.10	0.10	0.10	0.10	0.10
20	0.01	0.10	0.10	0.01	0.10	0.10	0.10	0.10	0.10	0.10
21	0.10	0.10	0.10	0.10	0.10	0.10	0.10	0.10	0.10	0.10
22	0.01	0.03	0.01	0.07	0.10	0.10	0.10	0.10	0.10	0.10
23	0.10	0.10	0.09	0.10	0.10	0.10	0.10	0.07	0.10	0.10
24	0.04	0.10	0.08	0.10	0.10	0.10	0.10	0.10	0.10	0.10
25	0.09	0.03	0.10	0.10	0.10	0.10	0.10	0.10	0.10	0.10

26	0.10	0.03	0.10	0.10	0.10	0.10	0.10	0.10	0.10	0.10
27	0.01	0.01	0.10	0.01	0.02	0.10	0.10	0.10	0.10	0.10
28	0.02	0.01	0.01	0.10	0.10	0.10	0.10	0.10	0.10	0.10
29	0.01	0.10	0.02	0.10	0.02	0.10	0.10	0.10	0.10	0.10
30	0.10	0.10	0.02	0.10	0.09	0.10	0.10	0.10	0.10	0.10
31	0.10	0.10	0.07	0.06	0.02	0.10	0.10	0.10	0.10	0.10
32	0.01	0.08	0.10	0.05	0.10	0.10	0.10	0.10	0.10	0.10
33	0.10	0.10	0.10	0.10	0.10	0.10	0.10	0.10	0.10	0.10
34	0.01	0.10	0.10	0.04	0.05	0.10	0.10	0.10	0.10	0.10
35	0.10	0.10	0.09	0.10	0.10	0.10	0.10	0.10	0.10	0.10
36	0.10	0.10	0.04	0.10	0.10	0.10	0.10	0.10	0.10	0.10
37	0.01	0.02	0.04	0.10	0.10	0.07	0.10	0.10	0.10	0.10
38	0.10	0.10	0.09	0.10	0.10	0.10	0.10	0.10	0.10	0.10
39	0.01	0.02	0.10	0.10	0.10	0.10	0.10	0.10	0.10	0.10
40	0.01	0.02	0.04	0.10	0.10	0.10	0.10	0.10	0.10	0.10
41	0.02	0.01	0.01	0.10	0.10	0.10	0.10	0.10	0.10	0.10
42	0.01	0.10	0.01	0.07	0.10	0.10	0.10	0.10	0.10	0.10
43	0.02	0.02	0.10	0.10	0.10	0.10	0.10	0.10	0.10	0.10
44	0.10	0.01	0.01	0.10	0.10	0.10	0.10	0.10	0.10	0.10
45	0.05	0.10	0.01	0.10	0.07	0.10	0.10	0.10	0.10	0.10
46	0.10	0.05	0.10	0.03	0.10	0.10	0.10	0.10	0.10	0.10
47	0.01	0.01	0.01	0.10	0.10	0.10	0.10	0.10	0.10	0.10
48	0.10	0.01	0.10	0.10	0.10	0.10	0.10	0.10	0.10	0.10
49	0.08	0.02	0.01	0.10	0.03	0.05	0.10	0.10	0.10	0.10
50	0.10	0.08	0.10	0.06	0.10	0.10	0.10	0.10	0.10	0.10
51	0.10	0.08	0.01	0.10	0.10	0.10	0.10	0.10	0.10	0.10
52	0.10	0.04	0.06	0.04	0.10	0.10	0.10	0.10	0.10	0.10
53	0.01	0.10	0.10	0.10	0.10	0.09	0.10	0.09	0.10	0.10
54	0.04	0.07	0.01	0.10	0.10	0.10	0.10	0.10	0.10	0.10
55	0.10	0.10	0.10	0.10	0.10	0.10	0.10	0.10	0.10	0.10
56	0.10	0.03	0.02	0.09	0.10	0.10	0.10	0.10	0.10	0.10
57	0.10	0.10	0.09	0.02	0.10	0.10	0.10	0.10	0.10	0.10
58	0.10	0.10	0.10	0.02	0.10	0.10	0.10	0.10	0.10	0.10
59	0.04	0.10	0.02	0.10	0.08	0.10	0.10	0.10	0.10	0.10
60	0.10	0.08	0.10	0.10	0.10	0.10	0.07	0.10	0.10	0.10
61	0.10	0.08	0.10	0.10	0.10	0.10	0.10	0.10	0.10	0.10
62	0.10	0.01	0.10	0.10	0.10	0.10	0.10	0.10	0.10	0.10
63	0.10	0.02	0.02	0.08	0.10	0.10	0.10	0.10	0.10	0.10
64	0.10	0.10	0.10	0.03	0.04	0.10	0.10	0.10	0.10	0.10
65	0.10	0.10	0.10	0.03	0.10	0.10	0.03	0.10	0.10	0.10
66	0.10	0.04	0.10	0.10	0.10	0.10	0.10	0.10	0.10	0.10
67	0.01	0.01	0.01	0.10	0.10	0.10	0.10	0.10	0.10	0.10
68	0.02	0.10	0.07	0.10	0.10	0.10	0.10	0.10	0.10	0.10
69	0.01	0.01	0.07	0.10	0.10	0.10	0.10	0.10	0.10	0.10
70	0.04	0.04	0.10	0.10	0.10	0.10	0.10	0.10	0.10	0.10

71	0.03	0.01	0.01	0.09	0.04	0.10	0.10	0.10	0.10	0.10
72	0.10	0.03	0.01	0.10	0.04	0.10	0.10	0.10	0.10	0.10
73	0.10	0.10	0.10	0.10	0.10	0.10	0.10	0.10	0.10	0.10
74	0.10	0.01	0.02	0.10	0.10	0.10	0.10	0.10	0.10	0.10
75	0.01	0.10	0.10	0.03	0.10	0.10	0.10	0.10	0.10	0.10
76	0.01	0.04	0.10	0.10	0.10	0.10	0.04	0.10	0.10	0.10
77	0.10	0.10	0.08	0.04	0.10	0.10	0.10	0.10	0.10	0.10
78	0.08	0.07	0.10	0.02	0.10	0.10	0.10	0.10	0.10	0.10
79	0.10	0.03	0.05	0.10	0.10	0.10	0.10	0.10	0.10	0.10
80	0.10	0.10	0.04	0.10	0.10	0.10	0.10	0.10	0.10	0.10
81	0.01	0.01	0.01	0.02	0.03	0.10	0.10	0.10	0.10	0.10
82	0.10	0.05	0.02	0.03	0.06	0.07	0.10	0.10	0.08	0.10
83	0.10	0.02	0.01	0.10	0.10	0.10	0.10	0.10	0.10	0.10
84	0.03	0.10	0.10	0.10	0.10	0.10	0.10	0.10	0.10	0.10
85	0.10	0.10	0.01	0.10	0.06	0.10	0.10	0.10	0.10	0.10
86	0.10	0.09	0.09	0.10	0.10	0.10	0.10	0.04	0.10	0.10
87	0.10	0.02	0.02	0.10	0.10	0.10	0.10	0.10	0.10	0.10
88	0.01	0.10	0.10	0.10	0.10	0.10	0.10	0.10	0.10	0.10
89	0.10	0.10	0.10	0.10	0.10	0.10	0.10	0.10	0.10	0.10
90	0.01	0.10	0.09	0.04	0.01	0.10	0.10	0.10	0.10	0.10
91	0.03	0.10	0.10	0.01	0.01	0.10	0.10	0.10	0.10	0.10
92	0.10	0.01	0.10	0.05	0.07	0.10	0.10	0.10	0.10	0.10
93	0.10	0.01	0.04	0.10	0.10	0.10	0.10	0.10	0.10	0.10
94	0.07	0.10	0.10	0.10	0.10	0.10	0.10	0.10	0.10	0.10
95	0.01	0.06	0.10	0.10	0.10	0.09	0.10	0.10	0.10	0.10
96	0.01	0.01	0.03	0.09	0.02	0.10	0.10	0.10	0.10	0.10
97	0.01	0.10	0.03	0.10	0.10	0.10	0.10	0.09	0.10	0.10
98	0.01	0.01	0.08	0.10	0.04	0.10	0.10	0.10	0.10	0.10
99	0.10	0.10	0.10	0.10	0.10	0.10	0.10	0.10	0.10	0.10
100	0.03	0.10	0.10	0.10	0.10	0.10	0.10	0.10	0.10	0.10
101	0.10	0.01	0.03	0.10	0.10	0.10	0.10	0.10	0.10	0.10
102	0.03	0.05	0.04	0.10	0.01	0.10	0.10	0.10	0.10	0.10

ABP, arterial blood pressure; ADF, Augmented Dickey-Fuller; COx, cerebral oximetry index with CPP; COx-a, cerebral oximetry index with ABP; CPP, cerebral perfusion pressure; HC, healthy control volunteer group; KPSS, Kwiatkowski-Phillips-Schmidt-Shin; rSO_2, regional cerebral oxygen saturation.

File S2b: ADF and KPSS P-Values for Non-Differenced and 1st Order Differenced in 1-Minute Data Resolution – SP Patients

ADF p-values for 1-minute data resolution										
Patient	*Non-Differenced*					*1st Order Differenced*				
	ABP	rSO_2_L	rSO_2_R	COx-a_L	COx-a_R	ABP	rSO_2_L	rSO_2_R	COx-a_L	COx-a_R
1	0.74	0.59	0.57	0.01	0.01	0.01	0.01	0.01	0.01	0.01
2	0.45	0.01	0.01	0.01	0.01	0.01	0.01	0.01	0.01	0.01
3	0.01	0.01	0.01	0.01	0.01	0.01	0.01	0.01	0.01	0.01
4	0.02	0.99	0.99	0.01	0.01	0.01	0.01	0.01	0.01	0.01
5	0.01	0.66	0.74	0.01	0.01	0.01	0.01	0.01	0.01	0.01
6	0.01	0.01	0.68	0.04	0.04	0.01	0.01	0.01	0.01	0.01
7	0.01	0.09	0.15	0.01	0.01	0.01	0.01	0.01	0.01	0.01
8	0.01	0.94	0.01	0.01	0.01	0.01	0.01	0.01	0.01	0.01
9	0.01	0.01	0.88	0.01	0.01	0.01	0.01	0.01	0.01	0.01
10	0.01	0.76	0.98	0.01	0.01	0.01	0.01	0.01	0.01	0.01
11	0.01	0.01	0.03	0.01	0.01	0.01	0.01	0.01	0.01	0.01
12	0.01	0.09	0.08	0.01	0.01	0.01	0.01	0.01	0.01	0.01
13	0.01	0.66	0.31	0.01	0.01	0.01	0.01	0.01	0.01	0.01
14	0.02	0.94	0.69	0.01	0.01	0.01	0.01	0.01	0.01	0.01
15	0.01	0.77	0.01	0.01	0.01	0.01	0.01	0.01	0.01	0.01
16	0.01	0.45	0.66	0.01	0.01	0.01	0.01	0.01	0.01	0.01
17	0.02	0.10	0.01	0.01	0.01	0.01	0.01	0.01	0.01	0.01
18	0.19	0.03	0.22	0.01	0.01	0.01	0.01	0.01	0.01	0.01
19	0.01	0.02	0.79	0.01	0.01	0.01	0.01	0.01	0.01	0.01
20	0.01	0.09	0.41	0.01	0.01	0.01	0.01	0.01	0.01	0.01
21	0.01	0.08	0.01	0.01	0.01	0.01	0.01	0.01	0.01	0.01
22	0.01	0.27	0.04	0.01	0.01	0.01	0.01	0.01	0.01	0.01
23	0.01	0.19	0.30	0.01	0.01	0.01	0.01	0.01	0.01	0.01
24	0.01	0.24	0.34	0.01	0.01	0.01	0.01	0.01	0.01	0.01
25	0.39	0.95	0.66	0.01	0.01	0.01	0.01	0.01	0.01	0.01
26	0.15	0.62	0.79	0.01	0.01	0.01	0.01	0.01	0.01	0.01
27	0.10	0.89	0.81	0.01	0.01	0.01	0.01	0.01	0.01	0.01

KPSS p-values for 1-minute data resolution										
Patient	*Non-Differenced*					*1st Order Differenced*				
	ABP	rSO_2_L	rSO_2_R	COx-a_L	COx-a_R	ABP	rSO_2_L	rSO_2_R	COx-a_L	COx-a_R
1	0.08	0.01	0.01	0.10	0.10	0.10	0.10	0.10	0.10	0.10
2	0.01	0.01	0.01	0.08	0.10	0.10	0.10	0.10	0.10	0.10
3	0.01	0.01	0.01	0.10	0.10	0.10	0.10	0.10	0.10	0.10
4	0.01	0.01	0.01	0.10	0.10	0.10	0.05	0.01	0.10	0.10
5	0.10	0.01	0.01	0.10	0.10	0.10	0.10	0.10	0.10	0.10
6	0.04	0.01	0.10	0.10	0.10	0.10	0.10	0.10	0.10	0.10
7	0.01	0.01	0.01	0.10	0.10	0.10	0.10	0.10	0.10	0.10
8	0.01	0.01	0.02	0.06	0.03	0.10	0.10	0.10	0.10	0.10
9	0.07	0.01	0.01	0.10	0.01	0.10	0.10	0.10	0.10	0.10
10	0.05	0.07	0.07	0.10	0.10	0.10	0.10	0.10	0.10	0.10

11	0.01	0.05	0.01	0.10	0.10	0.10	0.10	0.10	0.10	0.10
12	0.10	0.09	0.01	0.10	0.10	0.10	0.10	0.10	0.10	0.10
13	0.10	0.01	0.09	0.10	0.10	0.10	0.10	0.10	0.10	0.10
14	0.02	0.09	0.07	0.10	0.10	0.10	0.10	0.10	0.10	0.10
15	0.10	0.03	0.10	0.10	0.10	0.10	0.10	0.10	0.10	0.10
16	0.10	0.01	0.01	0.01	0.07	0.10	0.10	0.10	0.10	0.10
17	0.02	0.07	0.01	0.10	0.07	0.10	0.10	0.10	0.10	0.10
18	0.10	0.01	0.01	0.10	0.10	0.10	0.10	0.10	0.10	0.10
19	0.01	0.05	0.01	0.10	0.10	0.10	0.10	0.10	0.10	0.10
20	0.08	0.01	0.01	0.06	0.10	0.10	0.10	0.10	0.10	0.10
21	0.02	0.01	0.01	0.10	0.10	0.10	0.10	0.10	0.10	0.10
22	0.01	0.10	0.08	0.10	0.02	0.10	0.10	0.10	0.10	0.10
23	0.01	0.10	0.07	0.05	0.01	0.10	0.10	0.10	0.10	0.10
24	0.10	0.03	0.01	0.10	0.10	0.10	0.10	0.10	0.10	0.10
25	0.01	0.01	0.01	0.10	0.10	0.10	0.05	0.01	0.10	0.10
26	0.01	0.03	0.03	0.10	0.10	0.10	0.04	0.08	0.10	0.10
27	0.10	0.01	0.01	0.10	0.09	0.10	0.10	0.10	0.10	0.10

ABP, arterial blood pressure; ADF, Augmented Dickey-Fuller; COx, cerebral oximetry index with CPP; COx-a, cerebral oximetry index with ABP; CPP, cerebral perfusion pressure; KPSS, Kwiatkowski-Phillips-Schmidt-Shin; rSO_2, regional cerebral oxygen saturation; SP, elective spinal surgery patient group.

File S2c: ADF and KPSS P-Values for Non-Differenced and 1st Order Differenced in 1-Minute Data Resolution – TBI Patients

ADF p-values for 1-minute data																
Patient	*Non-Differenced*								*1st Order Differenced*							
	ABP	CPP	rSO2_L	rSO2_R	COx_L	COx_R	COx-a_L	COx-a_R	ABP	CPP	rSO2_L	rSO2_R	COx_L	COx_R	COx-a_L	COx-a_R
1	0.01	0.01	0.01	NA	0.01	NA	0.01	NA	0.01	0.01	0.01	NA	0.01	NA	0.01	NA
2	0.01	0.01	0.01	0.01	0.01	0.01	0.01	0.01	0.01	0.01	0.01	0.01	0.01	0.01	0.01	0.01
3	0.01	0.01	0.01	0.55	0.01	0.01	0.01	0.01	0.01	0.01	0.01	0.01	0.01	0.01	0.01	0.01
4	0.01	0.01	0.24	0.01	0.01	0.01	0.01	0.01	0.01	0.01	0.01	0.01	0.01	0.01	0.01	0.01
5	0.01	0.01	0.01	0.62	0.01	0.01	0.01	0.01	0.01	0.01	0.01	0.01	0.01	0.01	0.01	0.01
6	0.01	0.01	0.23	0.02	NA	0.01	NA	0.01	0.01	0.01	0.01	0.01	NA	0.01	NA	0.01
7	0.01	0.01	0.01	0.01	0.01	0.01	0.01	0.01	0.01	0.01	0.01	0.01	0.01	0.01	0.01	0.01
8	0.01	0.01	0.01	0.02	0.01	0.01	0.01	0.01	0.01	0.01	0.01	0.01	0.01	0.01	0.01	0.01
9	0.01	0.27	0.01	0.01	0.01	0.01	0.01	0.01	0.01	0.01	0.01	0.01	0.01	0.01	0.01	0.01
10	0.01	0.01	0.01	0.01	0.01	0.01	0.01	0.01	0.01	0.01	0.01	0.01	0.01	0.01	0.01	0.01
11	0.01	0.01	0.01	0.01	0.01	0.01	0.01	0.01	0.01	0.01	0.01	0.01	0.01	0.01	0.01	0.01
12	0.01	0.01	0.01	NA	0.01	NA	0.01	NA	0.01	0.01	0.01	NA	0.01	NA	0.01	NA
13	0.01	0.16	0.05	0.43	0.01	0.01	0.01	0.01	0.01	0.01	0.01	0.01	0.01	0.01	0.01	0.01
14	0.01	0.02	0.01	0.01	0.01	0.01	0.01	0.01	0.01	0.01	0.01	0.01	0.01	0.01	0.01	0.01
15	0.01	0.01	0.01	0.01	0.01	0.01	0.01	0.01	0.01	0.01	0.01	0.01	0.01	0.01	0.01	0.01
16	0.01	0.01	0.03	0.01	0.01	0.01	0.01	0.01	0.01	0.01	0.01	0.01	0.01	0.01	0.01	0.01
17	0.01	0.01	0.01	0.01	0.01	0.01	0.01	0.01	0.01	0.01	0.01	0.01	0.01	0.01	0.01	0.01
18	0.01	0.01	0.01	0.01	0.01	0.01	0.01	0.01	0.01	0.01	0.01	0.01	0.01	0.01	0.01	0.01
19	0.01	0.01	0.01	0.01	0.01	0.01	0.01	0.01	0.01	0.01	0.01	0.01	0.01	0.01	0.01	0.01
20	0.01	0.01	0.01	0.01	0.01	0.01	0.01	0.01	0.01	0.01	0.01	0.01	0.01	0.01	0.01	0.01
21	0.01	0.01	0.01	0.01	0.01	0.01	0.01	0.01	0.01	0.01	0.01	0.01	0.01	0.01	0.01	0.01
22	0.01	0.01	0.01	0.01	0.01	0.01	0.01	0.01	0.01	0.01	0.01	0.01	0.01	0.01	0.01	0.01
23	0.01	0.01	0.01	0.01	0.01	0.01	0.01	0.01	0.01	0.01	0.01	0.01	0.01	0.01	0.01	0.01
24	0.01	0.01	0.01	0.95	0.01	0.01	0.01	0.01	0.01	0.01	0.01	0.01	0.01	0.01	0.01	0.01
25	0.01	0.01	0.06	0.06	0.01	0.01	0.01	0.01	0.01	0.01	0.01	0.01	0.01	0.01	0.01	0.01
26	0.01	0.01	0.02	0.24	0.01	0.01	0.01	0.01	0.01	0.01	0.01	0.01	0.01	0.01	0.01	0.01
27	0.01	0.01	0.49	0.39	0.01	0.01	0.01	0.01	0.01	0.01	0.01	0.01	0.01	0.01	0.01	0.01
28	0.01	0.01	0.06	0.01	0.01	0.01	0.01	0.01	0.01	0.01	0.01	0.01	0.01	0.01	0.01	0.01
29	0.01	0.01	0.01	0.01	0.01	0.01	0.01	0.01	0.01	0.01	0.01	0.01	0.01	0.01	0.01	0.01
30	0.01	0.01	0.01	0.01	0.01	0.01	0.01	0.01	0.01	0.01	0.01	0.01	0.01	0.01	0.01	0.01
31	0.01	0.01	0.01	0.01	0.01	0.01	0.01	0.01	0.01	0.01	0.01	0.01	0.01	0.01	0.01	0.01
32	0.01	0.01	0.08	0.01	0.01	0.01	0.01	0.01	0.01	0.01	0.01	0.01	0.01	0.01	0.01	0.01
33	0.01	0.01	0.01	0.01	0.01	0.01	0.01	0.01	0.01	0.01	0.01	0.01	0.01	0.01	0.01	0.01
34	0.01	0.01	0.43	0.01	0.01	0.01	0.01	0.01	0.01	0.01	0.01	0.01	0.01	0.01	0.01	0.01
35	0.01	0.01	0.01	0.01	0.01	0.01	0.01	0.01	0.01	0.01	0.01	0.01	0.01	0.01	0.01	0.01
36	0.01	0.04	0.34	0.75	0.01	0.01	0.01	0.01	0.01	0.01	0.01	0.01	0.01	0.01	0.01	0.01
37	0.01	0.01	0.01	0.01	0.01	0.01	0.01	0.01	0.01	0.01	0.01	0.01	0.01	0.01	0.01	0.01
38	0.01	0.01	0.01	0.01	0.01	0.01	0.01	0.01	0.01	0.01	0.01	0.01	0.01	0.01	0.01	0.01
39	0.01	0.01	0.01	0.01	0.01	0.01	0.01	0.01	0.01	0.01	0.01	0.01	0.01	0.01	0.01	0.01
40	0.01	0.01	0.01	0.01	0.01	0.01	0.01	0.01	0.01	0.01	0.01	0.01	0.01	0.01	0.01	0.01
41	0.01	0.01	0.01	NA	0.01	NA	0.01	NA	0.01	0.01	0.01	NA	0.01	NA	0.01	NA
42	0.01	0.01	0.01	0.01	0.01	0.01	0.01	0.01	0.01	0.01	0.01	0.01	0.01	0.01	0.01	0.01
43	0.01	0.01	0.01	0.01	0.01	0.01	0.01	0.01	0.01	0.01	0.01	0.01	0.01	0.01	0.01	0.01
44	0.01	0.01	0.24	NA	0.01	NA	0.01	NA	0.01	0.01	0.01	NA	0.01	NA	0.01	NA
45	0.01	0.01	0.04	0.01	0.01	0.01	0.01	0.01	0.01	0.01	0.01	0.01	0.01	0.01	0.01	0.01
46	0.01	0.01	0.01	0.01	0.01	0.01	0.01	0.01	0.01	0.01	0.01	0.01	0.01	0.01	0.01	0.01
47	0.01	0.01	0.01	0.01	0.01	0.01	0.01	0.01	0.01	0.01	0.01	0.01	0.01	0.01	0.01	0.01
48	0.01	0.01	0.01	0.01	0.01	0.01	0.01	0.01	0.01	0.01	0.01	0.01	0.01	0.01	0.01	0.01
49	0.01	0.01	0.17	0.03	0.01	0.01	0.01	0.01	0.01	0.01	0.01	0.01	0.01	0.01	0.01	0.01
50	0.01	0.01	0.01	0.01	0.01	0.01	0.01	0.01	0.01	0.01	0.01	0.01	0.01	0.01	0.01	0.01
51	0.01	0.01	0.01	0.01	0.01	0.01	0.01	0.01	0.01	0.01	0.01	0.01	0.01	0.01	0.01	0.01
52	0.01	0.01	0.01	0.01	0.01	0.01	0.01	0.01	0.01	0.01	0.01	0.01	0.01	0.01	0.01	0.01
53	0.01	0.01	0.01	0.01	0.01	0.01	0.01	0.01	0.01	0.01	0.01	0.01	0.01	0.01	0.01	0.01
54	0.01	0.01	0.01	0.01	0.01	0.01	0.01	0.01	0.01	0.01	0.01	0.01	0.01	0.01	0.01	0.01
55	0.01	0.01	0.01	0.01	0.01	0.01	0.01	0.01	0.01	0.01	0.01	0.01	0.01	0.01	0.01	0.01
56	0.01	0.01	NA	0.01	NA	0.01	NA	0.01	0.01	0.01	NA	0.01	NA	0.01	NA	0.01
57	0.01	0.01	0.01	0.01	0.01	0.01	0.01	0.01	0.01	0.01	0.01	0.01	0.01	0.01	0.01	0.01
58	0.01	0.01	0.01	0.01	0.01	0.01	0.01	0.01	0.01	0.01	0.01	0.01	0.01	0.01	0.01	0.01
59	0.01	0.01	0.01	0.01	0.01	0.01	0.01	0.01	0.01	0.01	0.01	0.01	0.01	0.01	0.01	0.01
60	0.01	0.01	0.01	NA	0.01	NA	0.01	NA	0.01	0.01	0.01	NA	0.01	NA	0.01	NA
61	0.01	0.01	0.01	0.01	0.01	0.01	0.01	0.01	0.01	0.01	0.01	0.01	0.01	0.01	0.01	0.01
62	0.01	0.01	0.01	0.01	0.01	0.01	0.01	0.01	0.01	0.01	0.01	0.01	0.01	0.01	0.01	0.01
63	0.01	0.01	0.01	0.01	0.01	0.01	0.01	0.01	0.01	0.01	0.01	0.01	0.01	0.01	0.01	0.01
64	0.01	0.01	0.01	0.01	0.01	0.01	0.01	0.01	0.01	0.01	0.01	0.01	0.01	0.01	0.01	0.01
65	0.01	0.01	0.01	0.01	0.01	0.01	0.01	0.01	0.01	0.01	0.01	0.01	0.01	0.01	0.01	0.01
66	0.01	0.01	0.01	0.01	0.01	0.01	0.01	0.01	0.01	0.01	0.01	0.01	0.01	0.01	0.01	0.01
67	0.01	0.01	0.01	0.53	0.04	0.01	0.04	0.01	0.01	0.01	0.07	0.01	0.05	0.01	0.01	0.01

68	0.01	0.01	0.01	0.01	0.01	0.01	0.01	0.01	0.01	0.01	0.01	0.01	0.01	0.01	0.01	0.01
69	0.01	0.01	0.01	0.01	0.01	0.01	0.01	0.01	0.01	0.01	0.01	0.01	0.01	0.01	0.01	0.01
70	0.01	0.01	0.01	0.01	0.01	0.01	0.01	0.01	0.01	0.01	0.01	0.01	0.01	0.01	0.01	0.01
71	0.01	0.01	0.01	NA	0.01	NA	0.01	NA	0.01	0.01	0.01	NA	0.01	NA	0.01	NA
72	0.01	0.01	0.01	0.02	0.01	0.01	0.01	0.01	0.01	0.01	0.01	0.01	0.01	0.01	0.01	0.01
73	0.01	0.34	0.01	0.01	0.48	0.70	0.01	0.01	0.01	0.05	0.01	0.01	0.37	0.69	0.01	0.01
74	0.01	0.01	0.06	0.01	0.01	0.01	0.01	0.01	0.01	0.01	0.01	0.01	0.01	0.01	0.01	0.01
75	0.01	0.01	0.03	0.01	0.01	0.01	0.01	0.01	0.01	0.01	0.01	0.01	0.01	0.01	0.01	0.01
76	0.01	0.01	0.01	0.01	0.01	0.01	0.01	0.01	0.01	0.01	0.01	0.01	0.01	0.01	0.01	0.01
77	0.01	0.01	0.01	0.01	0.01	0.01	0.01	0.01	0.01	0.01	0.01	0.01	0.01	0.01	0.01	0.01
78	0.01	0.01	0.99	0.99	0.01	0.01	0.01	0.01	0.01	0.01	0.01	0.01	0.01	0.01	0.01	0.01
79	0.01	0.01	0.01	0.01	0.01	0.01	0.01	0.01	0.01	0.01	0.01	0.01	0.01	0.01	0.01	0.01
80	0.01	0.01	0.01	0.01	0.01	0.01	0.01	0.01	0.01	0.01	0.01	0.01	0.01	0.01	0.01	0.01
81	0.01	0.01	0.01	0.01	0.01	0.01	0.01	0.01	0.01	0.01	0.01	0.01	0.01	0.01	0.01	0.01
82	0.01	0.01	0.01	0.01	0.01	0.01	0.01	0.01	0.01	0.01	0.01	0.01	0.01	0.01	0.01	0.01
83	0.01	0.01	0.01	0.01	0.01	0.01	0.01	0.01	0.01	0.01	0.01	0.01	0.01	0.01	0.01	0.01
84	0.01	0.01	0.01	0.01	0.01	0.01	0.01	0.01	0.01	0.01	0.01	0.01	0.01	0.01	0.01	0.01
85	0.01	0.01	0.01	0.01	0.01	0.01	0.01	0.01	0.01	0.01	0.01	0.01	0.01	0.01	0.01	0.01
86	0.01	0.01	0.93	0.02	0.01	0.01	0.01	0.01	0.01	0.01	0.52	0.01	0.01	0.01	0.01	0.01
87	0.01	0.01	0.01	0.01	0.01	0.01	0.01	0.01	0.01	0.01	0.01	0.01	0.01	0.01	0.01	0.01
88	0.01	0.01	0.01	0.01	0.01	0.01	0.01	0.01	0.01	0.01	0.01	0.01	0.01	0.01	0.01	0.01
89	0.01	0.01	0.01	0.01	0.01	0.01	0.01	0.01	0.01	0.01	0.01	0.01	0.01	0.01	0.01	0.01
90	0.01	0.01	0.01	0.01	0.01	0.01	0.01	0.01	0.01	0.01	0.01	0.01	0.01	0.01	0.01	0.01
91	0.01	0.01	0.01	0.01	0.01	0.01	0.01	0.01	0.01	0.01	0.01	0.01	0.01	0.01	0.01	0.01
92	0.01	0.01	NA	0.01	NA	0.01	NA	0.01	0.01	0.01	NA	0.01	NA	0.01	NA	0.01
93	0.01	0.01	0.01	0.02	0.01	0.01	0.01	0.01	0.01	0.01	0.01	0.01	0.01	0.01	0.01	0.01
94	0.01	0.01	0.01	0.01	0.01	0.01	0.01	0.01	0.01	0.01	0.01	0.01	0.01	0.01	0.01	0.01
95	0.01	0.01	0.01	0.01	0.01	0.01	0.01	0.01	0.01	0.01	0.01	0.01	0.01	0.01	0.01	0.01
96	0.01	0.01	0.01	0.01	0.01	0.01	0.01	0.01	0.01	0.01	0.01	0.01	0.01	0.01	0.01	0.01
97	0.01	0.01	0.01	0.01	0.01	0.01	0.01	0.01	0.01	0.01	0.01	0.01	0.01	0.01	0.01	0.01
98	0.01	0.01	0.01	0.01	0.01	0.01	0.01	0.01	0.01	0.01	0.01	0.01	0.01	0.01	0.01	0.01
99	0.01	0.01	0.01	NA	0.01	NA	0.01	NA	0.01	0.01	0.01	NA	0.01	NA	0.01	NA
100	0.01	0.01	0.01	0.01	0.01	0.01	0.01	0.01	0.01	0.01	0.01	0.01	0.01	0.01	0.01	0.01
101	0.01	0.01	0.01	0.01	0.01	0.01	0.01	0.01	0.01	0.01	0.01	0.01	0.01	0.01	0.01	0.01
**KPSS p-values for 1-minute data resolution**																
**Patient**	***Non-Differenced***								***1st Order Differenced***							
	**ABP**	**CPP**	**rSO_2_L**	**rSO_2_R**	**COx_L**	**COx_R**	**COx-a_L**	**COx-a_R**	**ABP**	**CPP**	**rSO_2_L**	**rSO_2_R**	**COx_L**	**COx_R**	**COx-a_L**	**COx-a_R**
1	0.01	0.01	0.01	NA	0.01	NA	0.02	NA	0.10	0.10	0.10	NA	0.10	NA	0.10	NA
2	0.01	0.01	0.01	0.01	0.10	0.10	0.09	0.01	0.10	0.10	0.10	0.02	0.10	0.10	0.10	0.10
3	0.01	0.01	0.01	0.01	0.01	0.01	0.01	0.01	0.10	0.10	0.10	0.10	0.10	0.10	0.10	0.10
4	0.01	0.01	0.01	0.01	0.10	0.05	0.10	0.02	0.10	0.10	0.10	0.10	0.10	0.10	0.10	0.10
5	0.01	0.01	0.01	0.01	0.01	0.01	0.01	0.01	0.10	0.10	0.10	0.10	0.10	0.10	0.10	0.10
6	0.01	0.01	0.01	0.01	NA	0.10	NA	0.10	0.10	0.09	0.10	0.01	NA	0.10	NA	0.10
7	0.01	0.01	0.01	0.01	0.04	0.10	0.06	0.10	0.10	0.10	0.10	0.10	0.10	0.10	0.10	0.10
8	0.01	0.01	0.01	0.01	0.01	0.01	0.01	0.01	0.10	0.10	0.10	0.10	0.10	0.10	0.10	0.10
9	0.01	0.10	0.01	0.01	0.10	0.10	0.01	0.09	0.10	0.10	0.09	0.10	0.10	0.10	0.10	0.10
10	0.07	0.01	0.01	0.01	0.10	0.10	0.10	0.10	0.10	0.10	0.10	0.10	0.10	0.10	0.10	0.10
11	0.01	0.01	0.01	0.01	0.01	0.01	0.01	0.01	0.10	0.10	0.10	0.01	0.10	0.10	0.10	0.10
12	0.01	0.01	0.01	NA	0.10	NA	0.01	NA	0.10	0.10	0.10	NA	0.10	NA	0.10	NA
13	0.05	0.01	0.01	0.01	0.02	0.01	0.04	0.01	0.10	0.10	0.10	0.10	0.10	0.10	0.10	0.10
14	0.01	0.01	0.01	0.01	0.09	0.01	0.02	0.01	0.10	0.10	0.10	0.10	0.10	0.10	0.10	0.10
15	0.01	0.01	0.01	0.01	0.10	0.01	0.10	0.05	0.10	0.10	0.10	0.10	0.10	0.10	0.10	0.10
16	0.01	0.01	0.01	0.01	0.10	0.10	0.05	0.10	0.10	0.10	0.10	0.10	0.10	0.10	0.10	0.10
17	0.01	0.01	0.01	0.01	0.01	0.01	0.01	0.02	0.10	0.10	0.10	0.10	0.10	0.10	0.10	0.10
18	0.01	0.01	0.01	0.01	0.02	0.01	0.10	0.02	0.10	0.10	0.10	0.10	0.10	0.10	0.10	0.10
19	0.01	0.01	0.01	0.01	0.01	0.01	0.01	0.01	0.10	0.10	0.10	0.10	0.10	0.10	0.10	0.10
20	0.01	0.01	0.01	0.01	0.01	0.01	0.01	0.10	0.10	0.10	0.10	0.10	0.10	0.10	0.10	0.10
21	0.01	0.02	0.01	0.01	0.02	0.01	0.09	0.04	0.10	0.10	0.10	0.10	0.10	0.10	0.10	0.10
22	0.01	0.07	0.01	0.01	0.01	0.01	0.01	0.01	0.10	0.10	0.10	0.10	0.10	0.10	0.10	0.10
23	0.01	0.01	0.01	0.01	0.01	0.01	0.04	0.01	0.10	0.10	0.10	0.10	0.10	0.10	0.10	0.10
24	0.01	0.01	0.01	0.05	0.10	0.10	0.08	0.10	0.10	0.10	0.10	0.10	0.10	0.10	0.10	0.10
25	0.01	0.01	0.01	0.01	0.10	0.01	0.10	0.01	0.10	0.10	0.10	0.10	0.10	0.10	0.10	0.10
26	0.01	0.01	0.01	0.01	0.01	0.01	0.01	0.01	0.10	0.10	0.10	0.10	0.10	0.10	0.10	0.10
27	0.01	0.01	0.01	0.01	0.10	0.10	0.10	0.02	0.10	0.10	0.01	0.10	0.10	0.10	0.10	0.10
28	0.01	0.01	0.01	0.01	0.01	0.01	0.01	0.01	0.10	0.10	0.10	0.10	0.10	0.10	0.10	0.10
29	0.01	0.01	0.01	0.01	0.01	0.03	0.10	0.10	0.10	0.08	0.10	0.10	0.10	0.10	0.10	0.10
30	0.01	0.01	0.01	0.01	0.10	0.10	0.10	0.10	0.10	0.10	0.10	0.10	0.10	0.10	0.10	0.10
31	0.01	0.01	0.01	0.01	0.10	0.01	0.10	0.01	0.10	0.10	0.10	0.10	0.10	0.10	0.10	0.10
32	0.01	0.04	0.01	0.01	0.04	0.10	0.10	0.04	0.10	0.10	0.10	0.10	0.10	0.10	0.10	0.10
33	0.01	0.01	0.01	0.01	0.10	0.01	0.10	0.01	0.10	0.10	0.10	0.10	0.10	0.10	0.10	0.10
34	0.01	0.01	0.01	0.01	0.07	0.03	0.10	0.03	0.10	0.10	0.10	0.10	0.10	0.10	0.10	0.10
35	0.01	0.01	0.01	0.01	0.01	0.01	0.01	0.01	0.10	0.10	0.10	0.10	0.10	0.10	0.10	0.10

36	0.01	0.10	0.01	0.01	0.01	0.02	0.01	0.08	0.10	0.10	0.10	0.10	0.10	0.10	0.10	0.10
37	0.01	0.01	0.01	0.01	0.01	0.01	0.01	0.01	0.10	0.04	0.10	0.10	0.10	0.10	0.10	0.10
38	0.01	0.01	0.01	0.01	0.01	0.02	0.01	0.10	0.10	0.10	0.10	0.10	0.10	0.10	0.10	0.10
39	0.01	0.01	0.01	0.01	0.02	0.04	0.07	0.05	0.10	0.10	0.05	0.10	0.10	0.10	0.10	0.10
40	0.01	0.01	0.01	0.01	0.01	0.01	0.04	0.01	0.10	0.10	0.07	0.10	0.10	0.10	0.10	0.10
41	0.01	0.01	0.01	NA	0.10	NA	0.10	NA	0.10	0.10	0.10	NA	0.10	NA	0.10	NA
42	0.03	0.01	0.01	0.01	0.01	0.01	0.01	0.01	0.10	0.10	0.09	0.10	0.10	0.10	0.10	0.10
43	0.10	0.02	0.01	0.01	0.10	0.10	0.10	0.04	0.10	0.10	0.10	0.10	0.10	0.10	0.10	0.10
44	0.01	0.01	0.01	NA	0.01	NA	0.01	NA	0.10	0.10	0.10	NA	0.10	NA	0.10	NA
45	0.01	0.01	0.01	0.01	0.04	0.01	0.02	0.01	0.10	0.10	0.10	0.10	0.10	0.10	0.10	0.10
46	0.01	0.01	0.01	0.02	0.05	0.10	0.02	0.10	0.10	0.10	0.10	0.10	0.10	0.10	0.10	0.10
47	0.01	0.06	0.01	0.01	0.01	0.05	0.04	0.07	0.10	0.10	0.10	0.10	0.10	0.10	0.10	0.10
48	0.01	0.01	0.01	0.01	0.01	0.01	0.10	0.10	0.10	0.10	0.10	0.10	0.10	0.10	0.10	0.10
49	0.01	0.01	0.01	0.04	0.10	0.10	0.02	0.06	0.10	0.10	0.10	0.10	0.10	0.10	0.10	0.10
50	0.01	0.01	0.01	0.01	0.01	0.01	0.01	0.01	0.10	0.10	0.10	0.10	0.10	0.10	0.10	0.10
51	0.01	0.01	0.01	0.01	0.10	0.10	0.10	0.10	0.10	0.10	0.10	0.03	0.10	0.10	0.10	0.10
52	0.01	0.01	0.01	0.01	0.01	0.01	0.01	0.01	0.10	0.10	0.10	0.10	0.10	0.10	0.10	0.10
53	0.01	0.01	0.01	0.01	0.01	0.01	0.01	0.02	0.10	0.10	0.10	0.10	0.10	0.10	0.10	0.10
54	0.01	0.01	0.01	0.01	0.01	0.10	0.01	0.10	0.10	0.10	0.10	0.04	0.10	0.10	0.10	0.10
55	0.01	0.02	0.01	0.01	0.01	0.01	0.01	0.02	0.10	0.10	0.10	0.10	0.10	0.10	0.10	0.10
56	0.04	0.01	NA	0.01	NA	0.10	NA	0.05	0.10	0.10	NA	0.10	NA	0.10	NA	0.10
57	0.01	0.01	0.01	0.01	0.10	0.01	0.01	0.01	0.10	0.10	0.10	0.10	0.10	0.10	0.10	0.10
58	0.01	0.01	0.01	0.01	0.01	0.02	0.01	0.01	0.10	0.10	0.10	0.10	0.10	0.10	0.10	0.10
59	0.01	0.01	0.01	0.01	0.02	0.10	0.02	0.09	0.10	0.10	0.10	0.10	0.10	0.10	0.10	0.10
60	0.01	0.01	0.01	NA	0.06	NA	0.10	NA	0.10	0.10	0.10	NA	0.10	NA	0.10	NA
61	0.10	0.10	0.01	0.01	0.10	0.01	0.10	0.01	0.10	0.10	0.09	0.10	0.10	0.10	0.10	0.10
62	0.01	0.02	0.01	0.01	0.10	0.01	0.10	0.01	0.10	0.10	0.10	0.10	0.10	0.10	0.10	0.10
63	0.01	0.01	0.01	0.01	0.01	0.01	0.10	0.01	0.10	0.10	0.10	0.10	0.10	0.10	0.10	0.10
64	0.01	0.01	0.01	0.01	0.06	0.01	0.10	0.02	0.10	0.10	0.10	0.08	0.10	0.10	0.10	0.10
65	0.01	0.01	0.01	0.01	0.01	0.01	0.01	0.01	0.10	0.10	0.10	0.10	0.10	0.10	0.10	0.10
66	0.01	0.01	0.01	0.01	0.10	0.01	0.07	0.02	0.10	0.10	0.10	0.10	0.10	0.10	0.10	0.10
67	0.01	0.01	0.10	0.01	0.07	0.01	0.06	0.01	0.10	0.10	0.05	0.10	0.10	0.10	0.10	0.10
68	0.01	0.01	0.01	0.01	0.01	0.01	0.01	0.01	0.10	0.10	0.10	0.10	0.10	0.10	0.10	0.10
69	0.01	0.01	0.01	0.01	0.01	0.01	0.01	0.01	0.10	0.10	0.10	0.10	0.10	0.10	0.10	0.10
70	0.01	0.01	0.01	0.01	0.01	0.01	0.01	0.01	0.10	0.10	0.10	0.10	0.10	0.10	0.10	0.10
71	0.10	0.10	0.01	NA	0.01	NA	0.10	NA	0.10	0.10	0.10	NA	0.10	NA	0.10	NA
72	0.01	0.01	0.01	0.01	0.01	0.01	0.01	0.01	0.10	0.10	0.10	0.10	0.10	0.10	0.10	0.10
73	0.01	0.10	0.01	0.01	0.08	0.10	0.10	0.05	0.10	0.10	0.10	0.10	0.10	0.10	0.10	0.10
74	0.01	0.01	0.01	0.01	0.05	0.10	0.01	0.10	0.10	0.10	0.10	0.10	0.10	0.10	0.10	0.10
75	0.01	0.01	0.01	0.01	0.01	0.10	0.01	0.10	0.10	0.10	0.10	0.10	0.10	0.10	0.10	0.10
76	0.01	0.01	0.01	0.01	0.01	0.01	0.01	0.01	0.10	0.10	0.10	0.10	0.10	0.10	0.10	0.10
77	0.01	0.01	0.01	0.01	0.01	0.04	0.01	0.01	0.10	0.10	0.10	0.10	0.10	0.10	0.10	0.10
78	0.01	0.01	0.01	0.01	0.05	0.01	0.10	0.01	0.10	0.10	0.10	0.10	0.10	0.10	0.10	0.10
79	0.01	0.01	0.01	0.01	0.10	0.01	0.10	0.01	0.10	0.10	0.10	0.10	0.10	0.10	0.10	0.10
80	0.01	0.01	0.01	0.01	0.01	0.01	0.01	0.01	0.10	0.10	0.10	0.10	0.10	0.10	0.10	0.10
81	0.04	0.01	0.01	0.01	0.01	0.01	0.01	0.01	0.10	0.10	0.10	0.10	0.10	0.10	0.10	0.10
82	0.01	0.01	0.01	0.01	0.01	0.01	0.03	0.10	0.10	0.10	0.10	0.10	0.10	0.10	0.10	0.10
83	0.01	0.01	0.01	0.01	0.01	0.01	0.01	0.01	0.10	0.10	0.10	0.10	0.10	0.10	0.10	0.10
84	0.01	0.01	0.01	0.01	0.10	0.04	0.05	0.01	0.10	0.10	0.10	0.10	0.10	0.10	0.10	0.10
85	0.01	0.01	0.01	0.01	0.01	0.04	0.01	0.10	0.10	0.10	0.10	0.10	0.10	0.10	0.10	0.10
86	0.01	0.01	0.01	0.01	0.10	0.10	0.10	0.10	0.10	0.10	0.10	0.10	0.10	0.10	0.10	0.10
87	0.01	0.01	0.01	0.01	0.01	0.01	0.01	0.01	0.10	0.10	0.10	0.10	0.10	0.10	0.10	0.10
88	0.01	0.01	0.01	0.01	0.10	0.10	0.01	0.01	0.10	0.10	0.10	0.10	0.10	0.10	0.10	0.10
89	0.03	0.01	0.10	0.01	0.01	0.03	0.01	0.01	0.10	0.10	0.10	0.10	0.10	0.10	0.10	0.10
90	0.01	0.01	0.01	0.01	0.01	0.01	0.01	0.01	0.10	0.10	0.10	0.10	0.10	0.10	0.10	0.10
91	0.01	0.01	0.01	0.01	0.01	0.01	0.01	0.01	0.10	0.10	0.10	0.10	0.10	0.10	0.10	0.10
92	0.01	0.01	0.10	0.01	NA	0.01	NA	0.01	0.10	0.10	NA	0.10	NA	0.10	NA	0.10
93	0.01	0.01	0.01	0.01	0.01	0.01	0.01	0.10	0.10	0.10	0.10	0.10	0.10	0.10	0.10	0.10
94	0.01	0.01	0.01	0.01	0.01	0.01	0.01	0.01	0.10	0.10	0.10	0.10	0.10	0.10	0.10	0.10
95	0.01	0.01	0.01	0.01	0.01	0.01	0.03	0.01	0.10	0.10	0.10	0.10	0.10	0.10	0.10	0.10
96	0.01	0.01	0.01	0.01	0.01	0.01	0.01	0.01	0.10	0.10	0.10	0.10	0.10	0.10	0.10	0.10
97	0.01	0.01	0.01	0.01	0.01	0.03	0.01	0.05	0.10	0.10	0.10	0.10	0.10	0.10	0.10	0.10
98	0.05	0.02	0.01	0.01	0.01	0.01	0.07	0.01	0.10	0.10	0.10	0.10	0.10	0.10	0.10	0.10
99	0.01	0.01	0.01	0.10	0.01	NA	0.01	NA	0.10	0.10	0.10	NA	0.10	NA	0.10	NA
100	0.01	0.01	0.01	0.01	0.01	0.01	0.01	0.01	0.10	0.10	0.10	0.10	0.10	0.10	0.10	0.10
101	0.01	0.01	0.01	0.01	0.01	0.01	0.01	0.01	0.10	0.10	0.10	0.10	0.10	0.10	0.10	0.10

ABP, arterial blood pressure; ADF, Augmented Dickey-Fuller; COx, cerebral oximetry index with CPP; COx-a, cerebral oximetry index with ABP; CPP, cerebral perfusion pressure; KPSS, Kwiatkowski-Phillips-Schmidt-Shin; rSO_2, regional cerebral oxygen saturation; TBI-GLR, traumatic brain injury patient group without bifrontal lobe pathology.

File S2d: ADF and KPSS results showing stationary vs non-stationary vs NA for physiologic signals – Original and 1st order differenced HC, SP, and TBI Data

**ADF results for non-differenced data**													
**Dataset**	**Temporal Resolution**	**ABP**			**CPP**			**rSO_2_L**			**rSO_2_R**		
		**S**	**NS**	**NA**	**S**	**NS**	**NA**	**S**	**NS**	**NA**	**S**	**NS**	**NA**
HC	10-second	86	16	0	–	–	–	82	20	0	91	11	0
	1-minute	18	84	0	–	–	–	17	85	0	22	80	0
	5-minute	10	13	79	–	–	–	9	15	78	8	16	78
SP	10-second	23	4	0	–	–	–	13	14	0	12	15	0
	1-minute	21	6	0	–	–	–	7	20	0	8	19	0
	5-minute	11	16	0	–	–	–	4	23	0	6	21	0
TBI	10-second	101	0	0	100	1	0	93	6	2	86	8	7
	1-minute	101	0	0	98	3	0	86	13	2	84	10	7
	5-minute	99	2	0	93	7	1	78	20	3	73	21	7
**ADF results for 1st order differenced data**													
**Dataset**	**Temporal Resolution**	**ABP**			**CPP**			**rSO_2_L**			**rSO_2_R**		
		**S**	**NS**	**NA**	**S**	**NS**	**NA**	**S**	**NS**	**NA**	**S**	**NS**	**NA**
HC	10-second	102	0	0	–	–	–	102	0	0	102	0	0
	1-minute	93	9	0	–	–	–	90	12	0	87	15	0
	5-minute	3	4	95	–	–	–	2	6	94	1	7	94
SP	10-second	27	0	0	–	–	–	27	0	0	27	0	0
	1-minute	27	0	0	–	–	–	27	0	0	27	0	0
	5-minute	25	2	0	–	–	–	16	11	0	16	11	0
TBI	10-second	101	0	0	101	0	0	99	0	2	94	0	7
	1-minute	101	0	0	101	0	0	97	2	2	94	0	7
	5-minute	101	0	0	99	1	1	98	0	3	94	0	7
**KPSS results for non-differenced data**													
**Dataset**	**Temporal Resolution**	**ABP**			**CPP**			**rSO_2_L**			**rSO_2_R**		
		**S**	**NS**	**NA**	**S**	**NS**	**NA**	**S**	**NS**	**NA**	**S**	**NS**	**NA**
HC	10-second	29	73	0	–	–	–	26	76	0	20	82	0
	1-minute	56	46	0	–	–	–	57	45	0	58	44	0
	5-minute	101	1	0	–	–	–	102	0	0	101	1	0
SP	10-second	1	26	0	–	–	–	0	27	0	1	26	0
	1-minute	12	15	0	–	–	–	6	21	0	7	20	0
	5-minute	17	10	0	–	–	–	15	12	0	13	14	0
TBI	10-second	1	100	0	0	101	0	2	98	1	2	94	5
	1-minute	5	96	0	7	94	0	3	97	1	1	94	6
	5-minute	18	83	0	20	81	0	13	87	1	11	84	6
**KPSS results for 1st order differenced data**													
**Dataset**	**Temporal Resolution**	**ABP**			**CPP**			**rSO_2_L**			**rSO_2_R**		
		**S**	**NS**	**NA**	**S**	**NS**	**NA**	**S**	**NS**	**NA**	**S**	**NS**	**NA**
HC	10-second	102	0	0	–	–	–	102	0	0	102	0	0
	1-minute	102	0	0	–	–	–	100	2	0	101	1	0
	5-minute	99	3	0	–	–	–	98	4	0	97	5	0
SP	10-second	27	0	0	–	–	–	25	2	0	24	3	0
	1-minute	27	0	0	–	–	–	24	3	0	25	2	0
	5-minute	27	0	0	–	–	–	25	2	0	26	1	0
TBI	10-second	101	0	0	98	3	0	97	2	2	92	2	7
	1-minute	101	0	0	100	1	0	97	2	2	89	5	7
	5-minute	101	0	0	101	0	0	96	3	2	91	3	7

ABP, arterial blood pressure; ADF, Augmented Dickey-Fuller; COx, cerebral oximetry index with CPP; COx-a, cerebral oximetry index with ABP; CPP, cerebral perfusion pressure; HC, healthy control volunteer group; rSO_2, regional cerebral oxygen saturation; NA, unable to assess stationarity; NS, non-stationary; S, stationary; SP, elective spinal surgery patient group; TBI, traumatic brain injury patient group.
