# Supplementary material for: Time-Series Autoregressive Models for Point and Interval Forecasting of Raw and Derived Commercial Near-Infrared Spectroscopy Measures: An Exploratory Cranial Trauma and Healthy Control Analysis
Source: Bioengineering (Basel). 2025 Jun 21;12(7):682. doi: 10.3390/bioengineering12070682 (PMC12292983; doi:10.3390/bioengineering12070682)
Supplement: Supplementary file 1 [file bioengineering-12-00682-s001.zip › File S3.pdf]

File S3 – Autoregressive Integrative Moving Average (ARIMA) Analysis

File S3 – Table of Contents

File S3a: Recorded AIC while fitting various ARIMA models – TBI Patient Example 2

File S3b: Optimal ARIMA Models based on AIC for HC, SP, and TBI Populations – 10-Second Data Resolution Example 4

File S3a: Recorded AIC while fitting various ARIMA models – TBI Patient Example

ARIMA Model	ABP	CPP	rSO_2_L	rSO_2_R	COx_L	COx_R	COx_a_L	COx_a_R
(1,1,0)	382288.6321	399490.6892	288185.5568	228020.6482	-228894.6288	-229656.5209	-224013.4010	-225717.8375
(1,1,1)	377414.5956	391217.9382	281928.4853	225684.8593	-228964.4420	-229658.0203	-224082.5431	-225837.6734
(1,1,2)	377041.1506	387829.8958	281772.3930	225683.3395	-228961.7435	-229656.9614	-224089.6173	-225836.3986
(1,1,3)	376689.5425	387547.0412	281741.7098	225591.5110	-228960.5016	-229655.8557	-224068.2507	-225830.5833
(1,1,4)	376682.9491	387422.9029	281736.9485	225571.7339	-228958.5136	-229654.5548	-224078.7352	-225830.1062
(1,1,5)	377357.8174	387319.8575	281734.2162	225291.7913	-228959.8554	-229653.8119	-224097.1076	-225829.8136
(1,1,6)	376646.4856	387134.4209	281597.6666	225212.1732	-228962.5435	-229670.3371	-224099.7028	-225841.7331
(1,1,7)	376643.2916	386630.4106	281602.6681	225214.1532	-228960.9803	-229668.3409	-224097.7971	-225839.7479
(1,1,8)	376644.8384	386626.3518	281689.4292	225049.5057	-230515.1518	-229677.5361	-225675.0414	-225849.3815
(1,1,9)	376640.4427	386627.7184	281668.3566	225050.6869	-228999.6482	-229700.5269	-224141.2385	-225866.0677
(1,1,10)	376641.7256	386614.3896	281668.9502	225049.0312	-229003.7644	-229699.0218	-224141.0545	-225866.1630
(2,1,0)	379498.4084	394512.2389	284660.7039	227031.9787	-228962.9387	-229657.9264	-224074.1640	-225827.8955
(2,1,1)	377296.2139	387317.7816	281730.8350	225683.9697	-228962.5830	-229656.4128	-224089.8336	-225834.2478
(2,1,2)	376690.0986	387259.5552	281727.5355	225194.5653	-228960.5841	-229655.2288	-224083.7607	-225834.6013
(2,1,3)	376693.8006	387258.6732	281726.7094	225148.2391	-228958.6197	-229653.5008	-224087.9119	-225832.6724
(2,1,4)	376689.6056	387097.0907	281698.2016	225149.9588	-228956.7730	-229652.7836	-224086.1502	-225828.9846
(2,1,5)	376686.5738	387052.5599	281728.2716	225133.2094	-228957.7324	-229651.6869	-224095.2338	-225828.1215
(2,1,6)	376675.8094	387126.0081	281606.2872	225109.8215	-228960.0323	-229667.4211	-224096.9359	-225838.8882
(2,1,7)	376563.4555	386628.8694	281598.8295	225092.2442	-228958.6739	-229665.4528	-224095.2115	-225836.9555
(2,1,8)	376647.2703	386633.0594	281603.7026	225050.3630	-230487.9991	-229675.4837	-225702.8145	-225847.6348
(2,1,9)	376355.9921	386628.1382	281605.5726	225050.9769	-230515.9195	-231276.1485	-224140.0233	-227441.9963
(2,1,10)	376356.6403	386629.8295	281564.0951	225052.4551	-230529.3280	-231285.9327	-224139.9640	-227455.6133
(3,1,0)	378538.6430	391221.4036	283194.9356	226846.0560	-228962.6781	-229656.8803	-224081.9486	-225836.4351
(3,1,1)	376978.6235	387260.8832	281726.7299	225602.3049	-228960.5844	-229655.1356	-224078.4446	-225834.3780
(3,1,2)	376682.1096	387260.3789	281731.3982	225148.8084	-228958.5696	-229653.1422	-224086.4370	-225832.1769
(3,1,3)	376685.8680	387257.4841	281731.0938	225149.2485	-228956.6187	-229651.8838	-224085.9368	-225830.7439
(3,1,4)	376704.6694	387003.0081	281641.7832	225151.0662	-228957.0490	-229653.4750	-224085.1740	-225829.9213
(3,1,5)	376568.1026	387064.5599	281649.9762	225135.1561	-228955.7004	-229650.0100	-224092.8345	-225826.6116
(3,1,6)	376471.4946	387054.1050	281641.3996	225110.9154	-228957.7915	-229682.8784	-224112.1642	-225847.4684
(3,1,7)	376511.8376	386590.3183	281641.6382	225073.5453	-228956.4544	-229680.9308	-224117.0750	-225847.7158
(3,1,8)	375437.5256	386576.0289	281468.7645	225042.4432	-230661.4098	-229673.2859	-225810.1275	-225845.4964
(3,1,9)	375288.1944	386628.9254	281471.7751	224999.8364	-230659.4078	-231330.0033	-225663.8277	-227493.0013
(3,1,10)	376352.0298	386592.9513	281567.6043	225055.4506	-230693.9235	-231484.3588	-225672.9868	-227442.3748
(4,1,0)	377833.1621	388665.9673	282309.4898	226169.5716	-228960.8953	-229655.4073	-224089.6164	-225834.4381
(4,1,1)	376826.3940	387247.8859	281728.1701	225578.6155	-228958.7000	-229653.8990	-224085.9488	-225832.4355
(4,1,2)	376774.2797	387225.3937	281724.2006	225150.5510	-228956.7568	-229651.9131	-224083.5851	-225830.3230
(4,1,3)	376685.4304	387131.3401	281722.4226	225150.4609	-228954.4492	-229649.9312	-224084.4753	-225829.8634
(4,1,4)	376645.3903	387137.7330	281645.1530	225151.0246	-228956.1443	-229651.6170	-224087.2676	-225828.1003
(4,1,5)	376555.7785	386979.7443	281642.6507	225100.4802	-228953.7773	-229659.1023	-224106.1872	-225827.5950
(4,1,6)	376528.5498	386695.6010	281640.9034	225084.1309	-229045.4956	-229683.0605	-224187.0795	-225841.7647
(4,1,7)	376513.0785	386528.7916	281565.3249	225078.6825	-229043.9843	-229677.6320	-224198.5166	-225847.2251
(4,1,8)	375511.4299	386545.7042	281516.3619	225046.7302	-230651.3697	-231207.3940	-225769.6683	-227609.4763
(4,1,9)	375417.0275	386631.7816	281446.8423	224996.4733	-230763.1500	-231206.3393	-225728.5054	-227376.5256
(4,1,10)	375041.3901	386401.5622	281464.1163	225046.2932	-230513.6548	-231461.1591	-225667.3665	-227643.1850
(5,1,0)	377638.3628	387889.1561	282046.7306	226086.1001	-228963.8924	-229660.2943	-224097.8440	-225833.7337
(5,1,1)	376625.3824	386947.5246	281669.4854	225464.5816	-228961.3954	-229656.3739	-224095.7651	-225831.3092
(5,1,2)	376619.1937	386655.0328	281605.3594	225141.4716	-228959.2966	-229654.3234	-224093.8936	-225829.3540
(5,1,3)	375701.2175	387119.3933	281610.0048	225110.6904	-228957.1527	-229652.3033	-224241.4568	-225827.3208
(5,1,4)	376550.3913	387117.0914	281613.1743	225103.4591	-228974.9607	-229650.1422	-224183.7859	-225825.4533
(5,1,5)	376518.2562	386651.2348	281640.9172	225056.0359	-228972.4569	-229831.0439	-224200.4916	-225826.3604
(5,1,6)	374843.2344	386754.2694	281566.3529	225088.2416	-228961.7469	-229676.1302	-224196.8609	-225836.8159
(5,1,7)	374865.6059	386581.0081	281472.5906	225070.3590	-228958.5253	-229842.1535	-224195.4998	-225839.1497
(5,1,8)	374890.9930	386564.3199	281394.7639	225046.8169	-230656.5258	-231399.6410	-225847.8243	-227616.8769
(5,1,9)	374852.9565	386610.6357	281396.9330	224982.0078	-230839.7948	-231439.2064	-225962.4906	-227549.1419
(5,1,10)	374914.4016	386437.0902	281392.8244	224984.0100	-230492.1587	-231595.4478	-225647.7842	-227700.9138
(6,1,0)	377633.0357	387733.7779	281924.3557	225927.7095	-228961.9419	-229664.7200	-224095.8824	-225837.2475

(6,1,1)	377642.1739	386721.8369	281636.3771	225463.4019	-228960.2671	-229664.6579	-224093.8440	-225836.0749
(6,1,2)	376533.6289	386672.2730	281606.4722	225136.9515	-228958.1226	-229662.7382	-224092.0664	-225834.5752
(6,1,3)	376475.5285	386646.0377	281608.6061	225097.0072	-228955.8927	-229832.9123	-224108.7243	-225832.7450
(6,1,4)	375679.8639	386607.5744	281604.4910	225090.0882	-228972.8705	-229677.6597	-224201.4953	-225841.9212
(6,1,5)	374919.1692	386601.5234	281565.3844	225089.3133	-228965.5393	-229827.2139	-224195.0137	-225835.9452
(6,1,6)	374860.1378	386588.1340	281566.9731	225048.0365	-228954.8232	-229678.6067	-224197.2552	-227598.8579
(6,1,7)	374682.4284	386589.5139	281504.9969	225041.0572	-230784.6150	-231653.8532	-225808.8932	-227819.4811
(6,1,8)	374659.2191	386552.8934	281373.5456	225012.1040	-230901.0012	-231684.1767	-225988.8202	-227835.0206
(6,1,9)	374643.6541	386157.6876	281502.7541	224982.4347	-230773.5143	-231630.8123	-225942.3432	-227774.8426
(6,1,10)	374636.2456	386455.1003	281498.1926	224975.8341	-230711.1518	-231663.2679	-225978.4171	-227675.4057
(7,1,0)	377484.0844	387434.8425	281860.5228	225926.5420	-228970.9233	-229674.8392	-224102.3009	-225841.8604
(7,1,1)	376455.8818	386713.1106	281623.6601	225451.3321	-228965.0372	-229669.2208	-224097.7183	-225838.1358
(7,1,2)	376455.5487	386720.2911	281627.0363	225104.3836	-228963.1503	-229667.1307	-224095.3748	-225836.1817
(7,1,3)	376447.7900	386652.1839	281522.7833	225086.7153	-228960.9934	-229680.7148	-224117.7865	-225834.2935
(7,1,4)	374939.1414	386605.8838	281477.1902	225080.8290	-228958.9306	-229679.5136	-224200.3883	-225832.2905
(7,1,5)	374888.6547	386597.4051	281520.6350	225075.6523	-228956.9219	-229678.2442	-224198.3378	-225830.2842
(7,1,6)	374863.3407	386595.2267	281402.7382	225057.6941	-230603.2524	-229677.1942	-224335.6381	-227806.3179
(7,1,7)	374858.7971	386617.8268	281196.6956	224972.1416	-230622.3801	-231498.1132	-225948.1067	-227769.2959
(7,1,8)	374652.7135	386552.2532	281240.8301	224936.0653	-230925.4810	-231684.1294	-226092.1961	-227895.1616
(7,1,9)	374721.1324	386178.6583	281382.7352	224942.9762	-230860.6500	-231733.6499	-225766.8802	-227849.5151
(7,1,10)	374686.5158	386462.0868	281525.3648	224953.8883	-230772.1806	-231633.1524	-225932.9581	-227872.7515
(8,1,0)	377252.5879	387361.9134	281761.7431	225480.7772	-228991.9843	-229694.3233	-224135.8416	-225862.2441
(8,1,1)	376453.3224	386619.6742	281717.7369	225419.6179	-228984.3697	-229687.9775	-225734.7145	-225857.2582
(8,1,2)	376459.8175	386605.2705	281625.6363	225078.7159	-230560.3444	-229685.8498	-225723.9021	-225854.6304
(8,1,3)	376457.6712	386651.8868	281501.1088	225069.3273	-230577.4858	-231387.8086	-225757.9752	-227598.6977
(8,1,4)	374897.5709	386573.3733	281498.3459	225064.6348	-230633.1128	-231421.6389	-225774.8428	-227603.2688
(8,1,5)	374861.9774	386607.7056	281541.9560	225067.6515	-230642.5929	-231327.2731	-225777.2270	-227583.5016
(8,1,6)	374779.4420	386459.8367	281395.8615	225064.9679	-230736.5304	-231643.3812	-225884.1166	-227757.1825
(8,1,7)	374642.3408	386512.7922	281255.3836	224930.8051	-230849.5983	-231521.9508	-226060.1329	-227918.5831
(8,1,8)	374714.8412	386517.9281	281205.7709	224961.8676	-230758.0704	-231564.5921	-225984.3436	-227781.9205
(8,1,9)	374628.9714	386514.8961	281398.0225	224947.4668	-230925.2373	-231743.4467	-225996.8018	-227835.5610
(8,1,10)	374611.2102	386408.2311	281283.0372	224943.4315	-230928.6345	-231744.5765	-225901.8669	-227799.9520
(9,1,0)	376940.1625	387237.9961	281663.9675	225482.7214	-229006.6049	-229704.6821	-224149.1779	-225876.4180
(9,1,1)	376412.2608	386616.8581	281630.7632	225190.1047	-229003.3539	-229702.7283	-224146.6130	-225873.5582
(9,1,2)	376326.3074	386623.5668	281619.0895	225038.8901	-229001.5558	-229700.7322	-224144.2949	-225870.8891
(9,1,3)	376461.5015	386639.5613	281497.9884	225000.8567	-230565.0848	-231550.6119	-225849.8821	-227704.5334
(9,1,4)	374865.8065	386653.1625	281482.4711	225003.5673	-230721.9901	-231471.4782	-225847.2163	-227707.6147
(9,1,5)	374868.3666	386582.0956	281455.0141	225045.8155	-230666.5574	-231412.0790	-225805.6605	-227684.8543
(9,1,6)	374745.0714	386452.9166	281227.2172	225030.8224	-230884.0589	-231679.2966	-225955.6972	-227762.9476
(9,1,7)	374650.8351	386474.6018	281291.9666	224942.8495	-230611.9539	-231721.0761	-225879.9408	-227870.3654
(9,1,8)	374650.8065	386081.1795	281188.3722	224948.9707	-230615.8675	-231376.4479	-226021.2252	-227814.7939
(9,1,9)	374615.5319	385966.5058	281265.8955	224940.7614	-230928.6114	-231672.9074	-225982.5789	-227831.4016
(9,1,10)	374634.5446	386046.2280	281253.2246	224973.0487	-230893.9332	-231749.9666	-226036.5716	-227882.0386
(10,1,0)	376729.1032	387159.1648	281619.0223	225257.0634	-229005.7877	-229702.7165	-224147.9046	-225876.5604
(10,1,1)	376730.9122	387161.0602	281620.8270	225230.3204	-229003.1800	-229700.7605	-224145.6550	-225873.8317
(10,1,2)	375351.9981	386618.2572	281507.0639	225006.7644	-229001.4560	-229698.7658	-224143.3480	-225871.1142
(10,1,3)	376442.8406	386602.2550	281455.3201	225008.7267	-230696.9869	-231528.0164	-225727.6270	-227510.8646
(10,1,4)	376249.8512	386522.7531	281460.8728	225000.0861	-230711.2672	-231512.2958	-225891.0871	-227638.2227
(10,1,5)	374849.1981	386590.3134	281461.4088	225046.6374	-230694.1618	-231464.8332	-225847.4079	-227632.7645
(10,1,6)	374679.0358	386405.6988	281364.4153	225031.5407	-230796.4476	-231672.6918	-225889.8312	-227609.3203
(10,1,7)	374676.7466	386425.8618	281375.8059	224933.4929	-230798.1895	-231555.2924	-226002.7232	-227722.5544
(10,1,8)	374699.2168	385985.0041	281376.8179	224915.9895	-230936.2466	-231747.4304	-225911.0650	-227786.9907
(10,1,9)	374733.4363	385994.3556	281352.1852	224978.3831	-230853.1989	-231711.2253	-226035.2218	-227861.4490
(10,1,10)	374711.0814	385928.4496	281316.3807	224862.1747	-230881.9897	-231760.5729	-225972.0967	-227788.8200

ABP, arterial blood pressure; AIC, Akaike Information Criterion; ARIMA, autoregressive integrative moving average; COx, cerebral oximetry index with CPP; COx-a, cerebral oximetry index with ABP; CPP, cerebral perfusion pressure; rSO_2, regional cerebral oxygen saturation; TBI, traumatic brain injury patient group.

File S3b: Optimal ARIMA Models based on AIC for HC, SP, and TBI Populations – 10-Second Data Resolution Example

HC Patient	ABP	rSO_2_L	rSO_2_R	COx-a_L	COx-a_R
1	(1,1,1)	(1,1,1)	(2,1,6)	(3,1,0)	(1,1,0)
2	(1,1,3)	(3,1,4)	(3,1,4)	(6,1,7)	(1,1,0)
3	(2,1,1)	(6,1,5)	(1,1,6)	(4,1,5)	(1,1,1)
4	(1,1,1)	(2,1,2)	(1,1,3)	(5,1,1)	(7,1,3)
5	(3,1,3)	(10,1,2)	(6,1,5)	(3,1,5)	(1,1,0)
6	(2,1,3)	(1,1,5)	(2,1,3)	(1,1,2)	(6,1,4)
7	(2,1,8)	(5,1,5)	(1,1,2)	(1,1,0)	(6,1,4)
8	(2,1,2)	(3,1,5)	(2,1,3)	(10,1,6)	(4,1,8)
9	(1,1,1)	(4,1,5)	(1,1,3)	(10,1,5)	(1,1,0)
10	(2,1,0)	(3,1,1)	(5,1,7)	(4,1,3)	(1,1,7)
11	(3,1,3)	(4,1,3)	(1,1,2)	(4,1,1)	(6,1,3)
12	(10,1,3)	(2,1,7)	(10,1,5)	(3,1,2)	(2,1,0)
13	(4,1,10)	(10,1,10)	(5,1,5)	(3,1,5)	(6,1,5)
14	(1,1,5)	(3,1,5)	(4,1,2)	(4,1,3)	(3,1,2)
15	(4,1,3)	(1,1,0)	(1,1,5)	(4,1,6)	(1,1,0)
16	(5,1,4)	(1,1,1)	(1,1,1)	(2,1,0)	(5,1,1)
17	(2,1,2)	(2,1,6)	(6,1,6)	(4,1,3)	(6,1,1)
18	(1,1,1)	(1,1,1)	(5,1,5)	(3,1,2)	(1,1,1)
19	(6,1,9)	(2,1,4)	(2,1,4)	(3,1,3)	(1,1,0)
20	(4,1,4)	(2,1,1)	(2,1,0)	(4,1,3)	(7,1,4)
21	(1,1,2)	(7,1,7)	(1,1,3)	(5,1,5)	(9,1,10)
22	(2,1,3)	(3,1,2)	(1,1,1)	(1,1,0)	(1,1,0)
23	(7,1,7)	(2,1,1)	(8,1,2)	(1,1,0)	(6,1,3)
24	(1,1,1)	(1,1,1)	(3,1,3)	(8,1,6)	(2,1,2)
25	(1,1,3)	(3,1,0)	(5,1,5)	(4,1,3)	(1,1,0)
26	(2,1,7)	(2,1,1)	(1,1,4)	(8,1,9)	(4,1,5)
27	(1,1,1)	(1,1,4)	(4,1,3)	(10,1,9)	(7,1,3)
28	(2,1,3)	(8,1,1)	(2,1,1)	(4,1,5)	(6,1,7)
29	(1,1,1)	(2,1,2)	(2,1,4)	(1,1,0)	(4,1,7)
30	(1,1,2)	(2,1,0)	(1,1,2)	(2,1,0)	(5,1,9)
31	(3,1,3)	(3,1,7)	(10,1,6)	(1,1,3)	(4,1,4)
32	(2,1,10)	(5,1,5)	(7,1,7)	(1,1,1)	(4,1,2)
33	(4,1,9)	(1,1,1)	(1,1,1)	(5,1,3)	(4,1,6)
34	(3,1,2)	(8,1,8)	(2,1,1)	(1,1,8)	(5,1,2)
35	(2,1,9)	(2,1,1)	(1,1,0)	(8,1,5)	(3,1,5)
36	(8,1,5)	(1,1,2)	(3,1,2)	(6,1,4)	(3,1,7)
37	(1,1,1)	(2,1,2)	(1,1,3)	(2,1,7)	(9,1,7)
38	(4,1,1)	(1,1,0)	(1,1,1)	(1,1,1)	(5,1,4)
39	(1,1,2)	(1,1,1)	(10,1,10)	(6,1,3)	(4,1,5)
40	(5,1,3)	(1,1,3)	(2,1,7)	(7,1,10)	(1,1,0)
41	(2,1,4)	(4,1,3)	(2,1,1)	(4,1,7)	(2,1,0)
42	(1,1,8)	(1,1,1)	(2,1,6)	(5,1,9)	(7,1,4)
43	(1,1,7)	(2,1,1)	(2,1,1)	(2,1,4)	(1,1,0)
44	(6,1,2)	(2,1,1)	(6,1,5)	(8,1,10)	(2,1,0)
45	(2,1,5)	(1,1,4)	(2,1,1)	(1,1,0)	(9,1,7)
46	(1,1,2)	(3,1,1)	(2,1,1)	(3,1,4)	(3,1,10)
47	(1,1,1)	(2,1,1)	(1,1,1)	(10,1,4)	(1,1,0)
48	(1,1,2)	(4,1,3)	(5,1,5)	(7,1,8)	(3,1,2)
49	(2,1,10)	(5,1,4)	(1,1,3)	(4,1,3)	(1,1,0)
50	(1,1,2)	(5,1,6)	(6,1,9)	(2,1,6)	(1,1,0)
51	(4,1,8)	(7,1,5)	(3,1,5)	(5,1,9)	(3,1,2)

SP Patient	ABP	rSO_2_L	rSO_2_R	COx-a_L	COx-a_R
1	(3,1,8)	(6,1,4)	(8,1,8)	(6,1,6)	(7,1,3)
2	(9,1,10)	(8,1,10)	(10,1,8)	(7,1,9)	(7,1,1)
3	(3,1,6)	(8,1,10)	(8,1,10)	(10,1,4)	(8,1,4)
4	(8,1,8)	(10,1,9)	(9,1,10)	(10,1,10)	(8,1,10)
5	(9,1,9)	(1,1,9)	(10,1,5)	(8,1,10)	(7,1,10)
6	(2,1,6)	(5,1,9)	(3,1,3)	(2,1,2)	(3,1,0)
7	(1,1,8)	(6,1,8)	(10,1,9)	(6,1,6)	(5,1,3)
8	(1,1,9)	(2,1,8)	(7,1,9)	(8,1,3)	(5,1,1)
9	(9,1,10)	(6,1,8)	(3,1,3)	(4,1,10)	(5,1,1)
10	(1,1,2)	(3,1,3)	(6,1,1)	(7,1,2)	(10,1,8)
11	(4,1,10)	(10,1,3)	(6,1,3)	(4,1,4)	(10,1,10)
12	(8,1,5)	(6,1,2)	(2,1,10)	(3,1,3)	(5,1,6)
13	(9,1,10)	(3,1,7)	(1,1,4)	(4,1,4)	(9,1,9)
14	(4,1,0)	(8,1,9)	(8,1,5)	(8,1,7)	(6,1,1)
15	(10,1,6)	(5,1,1)	(7,1,4)	(7,1,3)	(4,1,4)
16	(7,1,8)	(4,1,6)	(1,1,7)	(4,1,3)	(3,1,10)
17	(7,1,4)	(10,1,6)	(10,1,10)	(9,1,3)	(7,1,3)
18	(10,1,10)	(3,1,0)	(4,1,8)	(5,1,1)	(8,1,8)
19	(8,1,8)	(5,1,9)	(9,1,6)	(5,1,6)	(10,1,4)
20	(10,1,10)	(10,1,6)	(8,1,10)	(8,1,5)	(7,1,4)
21	(10,1,10)	(8,1,10)	(10,1,10)	(10,1,9)	(7,1,10)
22	(1,1,7)	(1,1,0)	(6,1,5)	(5,1,4)	(10,1,9)
23	(10,1,2)	(4,1,4)	(4,1,10)	(10,1,5)	(8,1,9)
24	(10,1,9)	(4,1,8)	(8,1,5)	(8,1,6)	(5,1,4)
25	(3,1,8)	(3,1,5)	(10,1,2)	(9,1,9)	(3,1,10)
26	(8,1,9)	(6,1,6)	(4,1,9)	(4,1,6)	(9,1,6)
27	(5,1,10)	(7,1,3)	(5,1,6)	(6,1,4)	(10,1,6)

TBI-GLR Patient	ABP	CPP	rSO_2_L	rSO_2_R	COx_L	COx_R	COx-a_L	COx-a_R
1	(10,1,10)	(10,1,9)	(7,1,10)	NA	(7,1,8)	NA	(7,1,10)	NA
2	(6,1,10)	(7,1,4)	(10,1,9)	(10,1,4)	(9,1,8)	(7,1,6)	(8,1,6)	(6,1,6)
3	(7,1,5)	(5,1,8)	(5,1,3)	(10,1,8)	(4,1,7)	(7,1,7)	(3,1,4)	(10,1,9)
4	(10,1,8)	(9,1,7)	(3,1,1)	(8,1,6)	(9,1,8)	(7,1,7)	(9,1,8)	(9,1,10)
5	(10,1,9)	(9,1,10)	(9,1,8)	NA	(8,1,9)	NA	(8,1,10)	NA
6	(7,1,10)	(10,1,7)	(6,1,10)	(1,1,2)	NA	(6,1,8)	NA	(10,1,10)
7	(9,1,10)	(1,1,5)	(6,1,8)	(5,1,5)	(7,1,4)	(8,1,7)	(4,1,7)	(8,1,7)
8	(6,1,10)	(6,1,10)	(5,1,10)	NA	(7,1,10)	NA	(7,1,10)	NA
9	(5,1,1)	(5,1,7)	(9,1,10)	(5,1,10)	(6,1,6)	(1,1,7)	(7,1,6)	(8,1,9)
10	(10,1,10)	(9,1,9)	(10,1,10)	(6,1,10)	(9,1,10)	(8,1,7)	(8,1,9)	(6,1,5)
11	(5,1,9)	(5,1,10)	(10,1,10)	(10,1,10)	(10,1,10)	(7,1,7)	(8,1,9)	(6,1,9)
12	(8,1,9)	(5,1,5)	(9,1,3)	NA	(8,1,9)	NA	(8,1,7)	NA
13	(9,1,10)	(7,1,10)	(1,1,8)	(10,1,5)	(9,1,4)	(6,1,8)	(7,1,6)	(10,1,10)
14	(10,1,5)	(9,1,10)	(10,1,10)	(7,1,8)	(10,1,9)	(8,1,4)	(8,1,10)	(6,1,6)
15	(5,1,5)	(7,1,4)	(10,1,10)	(6,1,5)	(10,1,10)	(4,1,5)	(9,1,4)	(5,1,7)
16	(8,1,8)	(10,1,10)	(5,1,2)	NA	(9,1,7)	NA	(9,1,10)	NA
17	(9,1,10)	(9,1,10)	(10,1,9)	(10,1,9)	(10,1,9)	(9,1,10)	(8,1,7)	(9,1,10)
18	(6,1,8)	(10,1,10)	(10,1,6)	(10,1,10)	(9,1,10)	(7,1,10)	(9,1,10)	(9,1,8)
19	(7,1,8)	(5,1,9)	(7,1,10)	(2,1,10)	(10,1,7)	(8,1,10)	(7,1,10)	(10,1,10)
20	(8,1,8)	(6,1,10)	(10,1,9)	(10,1,8)	(5,1,4)	(8,1,7)	(4,1,4)	(7,1,1)
21	(10,1,10)	(8,1,4)	(8,1,10)	(10,1,8)	(9,1,10)	(8,1,9)	(9,1,10)	(6,1,8)
22	(10,1,9)	(10,1,9)	(10,1,10)	(6,1,10)	(8,1,7)	(10,1,10)	(8,1,7)	(8,1,9)
23	(8,1,9)	(9,1,7)	(8,1,9)	(7,1,1)	(7,1,10)	(4,1,6)	(10,1,10)	(7,1,5)
24	(10,1,10)	(10,1,9)	(8,1,0)	(7,1,9)	(9,1,6)	(10,1,8)	(8,1,10)	(8,1,10)
25	(6,1,9)	(8,1,8)	(6,1,3)	(8,1,7)	(10,1,10)	(8,1,9)	(10,1,10)	(9,1,10)
26	(10,1,10)	(9,1,10)	(8,1,10)	(9,1,10)	(10,1,6)	(9,1,8)	(9,1,9)	(9,1,8)
27	(9,1,9)	(10,1,6)	(5,1,7)	(10,1,5)	(10,1,8)	(6,1,9)	(8,1,8)	(10,1,3)
28	(8,1,7)	(10,1,7)	(9,1,7)	(10,1,10)	(10,1,10)	(10,1,3)	(9,1,9)	(9,1,10)
29	(8,1,8)	(9,1,8)	(7,1,8)	(10,1,8)	(7,1,8)	(8,1,6)	(7,1,9)	(6,1,8)
30	(5,1,4)	(8,1,9)	(8,1,3)	(2,1,2)	(10,1,9)	(9,1,10)	(10,1,9)	(10,1,9)
31	(2,1,7)	(2,1,7)	(1,1,4)	(4,1,7)	(6,1,10)	(9,1,8)	(6,1,8)	(8,1,9)
32	(7,1,7)	(4,1,9)	(4,1,8)	NA	(6,1,7)	NA	(6,1,8)	NA
33	(7,1,0)	(6,1,0)	(5,1,1)	NA	(8,1,3)	NA	(8,1,3)	NA
34	(9,1,1)	(6,1,3)	NA	(3,1,1)	NA	(9,1,9)	NA	(8,1,7)
35	(8,1,5)	(8,1,8)	(8,1,10)	(10,1,8)	(9,1,6)	(10,1,10)	(9,1,6)	(10,1,9)
36	(10,1,10)	(7,1,8)	(8,1,6)	(2,1,4)	(7,1,4)	(3,1,9)	(4,1,4)	(6,1,6)
37	(10,1,10)	(9,1,7)	(7,1,10)	(10,1,10)	(10,1,10)	(10,1,10)	(10,1,9)	(10,1,10)
38	(6,1,10)	(3,1,10)	(6,1,9)	(8,1,7)	(10,1,10)	(10,1,10)	(10,1,10)	(9,1,9)
39	(9,1,10)	(9,1,10)	(6,1,2)	(5,1,7)	(10,1,10)	(8,1,6)	(7,1,10)	(10,1,5)
40	(10,1,10)	(10,1,10)	NA	(9,1,10)	NA	(10,1,7)	NA	(9,1,7)
41	(10,1,10)	(10,1,10)	(10,1,7)	NA	(8,1,1)	NA	(4,1,7)	NA
42	(8,1,10)	(10,1,5)	(8,1,3)	(7,1,10)	(9,1,6)	(9,1,5)	(10,1,4)	(9,1,6)
43	(1,1,4)	(4,1,10)	(6,1,10)	NA	(4,1,2)	NA	(4,1,2)	NA
44	(10,1,10)	(10,1,10)	(10,1,10)	NA	(9,1,9)	NA	(9,1,9)	NA
45	(7,1,10)	(9,1,10)	NA	(5,1,10)	NA	(7,1,9)	NA	(8,1,10)
46	(7,1,10)	(3,1,10)	(10,1,10)	NA	(10,1,9)	NA	(10,1,6)	NA
47	(10,1,8)	(10,1,8)	(5,1,7)	(10,1,6)	(7,1,9)	(6,1,7)	(8,1,10)	(6,1,8)
48	(10,1,10)	(10,1,10)	(9,1,8)	(9,1,9)	(8,1,10)	(8,1,10)	(9,1,8)	(8,1,5)
49	(2,1,9)	(2,1,2)	(4,1,10)	(5,1,5)	(10,1,4)	(9,1,9)	(10,1,9)	(5,1,5)
50	(10,1,2)	(10,1,1)	(10,1,9)	(10,1,10)	(7,1,10)	(8,1,9)	(9,1,8)	(9,1,7)
51	(10,1,9)	(10,1,8)	(10,1,10)	(7,1,7)	(4,1,9)	(5,1,8)	(10,1,9)	(9,1,5)

52	(1,1,2)	(4,1,3)	(5,1,7)	(6,1,4)	(4,1,5)
53	(1,1,1)	(4,1,3)	(6,1,2)	(1,1,3)	(1,1,0)
54	(2,1,1)	(1,1,1)	(2,1,4)	(5,1,4)	(1,1,0)
55	(5,1,5)	(1,1,1)	(1,1,1)	(3,1,0)	(4,1,1)
56	(1,1,1)	(1,1,1)	(10,1,7)	(4,1,6)	(2,1,3)
57	(2,1,1)	(5,1,6)	(5,1,8)	(5,1,6)	(8,1,3)
58	(1,1,10)	(9,1,2)	(5,1,5)	(1,1,2)	(5,1,4)
59	(1,1,1)	(5,1,9)	(3,1,3)	(3,1,4)	(8,1,8)
60	(1,1,2)	(1,1,1)	(2,1,5)	(1,1,0)	(3,1,2)
61	(3,1,0)	(6,1,10)	(5,1,6)	(1,1,0)	(2,1,2)
62	(2,1,3)	(6,1,8)	(2,1,9)	(1,1,1)	(3,1,2)
63	(3,1,4)	(1,1,3)	(5,1,2)	(1,1,1)	(3,1,2)
64	(4,1,3)	(1,1,1)	(3,1,8)	(2,1,3)	(3,1,2)
65	(1,1,1)	(8,1,3)	(1,1,2)	(3,1,5)	(4,1,7)
66	(2,1,5)	(7,1,0)	(5,1,4)	(2,1,0)	(1,1,8)
67	(1,1,1)	(2,1,2)	(1,1,1)	(3,1,2)	(9,1,7)
68	(1,1,2)	(3,1,3)	(1,1,2)	(2,1,3)	(7,1,5)
69	(3,1,3)	(1,1,1)	(5,1,6)	(8,1,0)	(1,1,2)
70	(2,1,1)	(1,1,1)	(4,1,5)	(1,1,2)	(4,1,5)
71	(5,1,6)	(2,1,1)	(4,1,4)	(2,1,3)	(4,1,5)
72	(7,1,4)	(4,1,6)	(1,1,3)	(4,1,5)	(4,1,3)
73	(1,1,1)	(2,1,6)	(3,1,2)	(1,1,0)	(5,1,4)
74	(4,1,7)	(1,1,0)	(8,1,8)	(3,1,2)	(1,1,0)
75	(1,1,1)	(2,1,4)	(5,1,3)	(6,1,5)	(4,1,4)
76	(7,1,5)	(2,1,2)	(1,1,1)	(6,1,6)	(9,1,9)
77	(1,1,1)	(1,1,1)	(5,1,4)	(8,1,2)	(1,1,0)
78	(1,1,0)	(8,1,5)	(3,1,0)	(2,1,1)	(7,1,3)
79	(5,1,5)	(4,1,7)	(1,1,4)	(4,1,3)	(1,1,0)
80	(1,1,1)	(2,1,2)	(7,1,2)	(3,1,2)	(4,1,4)
81	(3,1,9)	(8,1,4)	(7,1,7)	(1,1,7)	(4,1,5)
82	(2,1,3)	(1,1,1)	(1,1,1)	(3,1,2)	(1,1,0)
83	(2,1,1)	(10,1,8)	(9,1,8)	(1,1,0)	(1,1,0)
84	(3,1,4)	(3,1,5)	(2,1,2)	(4,1,1)	(4,1,4)
85	(3,1,4)	(9,1,8)	(2,1,6)	(7,1,3)	(3,1,2)
86	(8,1,5)	(1,1,0)	(2,1,5)	(3,1,0)	(2,1,1)
87	(1,1,2)	(1,1,2)	(1,1,1)	(4,1,5)	(3,1,2)
88	(3,1,2)	(5,1,3)	(3,1,3)	(1,1,1)	(1,1,0)
89	(6,1,2)	(2,1,1)	(9,1,3)	(2,1,6)	(4,1,0)
90	(4,1,3)	(6,1,3)	(8,1,8)	(1,1,1)	(3,1,4)
91	(2,1,6)	(2,1,7)	(2,1,3)	(7,1,10)	(6,1,2)
92	(6,1,5)	(2,1,9)	(10,1,5)	(6,1,5)	(5,1,4)
93	(1,1,1)	(2,1,2)	(1,1,1)	(2,1,6)	(4,1,5)
94	(5,1,5)	(1,1,1)	(1,1,1)	(5,1,6)	(5,1,4)
95	(4,1,7)	(2,1,4)	(6,1,5)	(6,1,2)	(1,1,4)
96	(2,1,5)	(2,1,4)	(2,1,8)	(3,1,7)	(9,1,2)
97	(7,1,5)	(2,1,5)	(1,1,1)	(1,1,1)	(4,1,4)
98	(4,1,10)	(8,1,6)	(2,1,7)	(1,1,0)	(3,1,2)
99	(2,1,1)	(8,1,7)	(4,1,4)	(2,1,3)	(1,1,0)
100	(1,1,2)	(1,1,2)	(2,1,4)	(7,1,7)	(7,1,7)
101	(1,1,1)	(1,1,1)	(1,1,1)	(3,1,8)	(1,1,0)
102	(1,1,1)	(8,1,8)	(5,1,5)	(4,1,3)	(1,1,1)

52	(10,1,6)	(7,1,10)	(6,1,9)	(9,1,10)	(8,1,7)	(9,1,9)	(7,1,8)	(7,1,10)
53	(9,1,10)	(6,1,9)	(5,1,9)	(9,1,6)	(10,1,10)	(9,1,10)	(9,1,8)	(10,1,10)
54	(5,1,10)	(9,1,7)	(10,1,10)	(10,1,9)	(10,1,8)	(10,1,10)	(8,1,9)	(8,1,9)
55	(10,1,10)	(7,1,7)	(9,1,2)	(2,1,7)	(8,1,9)	(10,1,8)	(8,1,8)	(7,1,7)
56	(6,1,8)	(9,1,6)	NA	(6,1,10)	NA	(9,1,6)	NA	(10,1,6)
57	(10,1,10)	(10,1,10)	(10,1,10)	(10,1,9)	(10,1,10)	(10,1,10)	(10,1,10)	(9,1,8)
58	(9,1,9)	(5,1,10)	(6,1,4)	(7,1,7)	(8,1,9)	(7,1,8)	(8,1,10)	(6,1,7)
59	(3,1,4)	(3,1,3)	NA	(2,1,2)	NA	(9,1,5)	NA	(5,1,6)
60	(10,1,6)	(10,1,1)	(9,1,7)	NA	(7,1,8)	NA	(5,1,4)	NA
61	(1,1,9)	(1,1,9)	(10,1,6)	(9,1,9)	(9,1,9)	(6,1,9)	(7,1,10)	(6,1,9)
62	(10,1,10)	(10,1,10)	NA	(1,1,9)	NA	(8,1,9)	NA	(8,1,10)
63	(10,1,7)	(10,1,10)	(9,1,10)	(4,1,10)	(9,1,10)	(10,1,9)	(9,1,10)	(10,1,9)
64	(9,1,10)	(8,1,10)	(8,1,10)	NA	(7,1,8)	NA	(7,1,8)	NA
65	(8,1,10)	(6,1,10)	(10,1,10)	(10,1,9)	(9,1,10)	(10,1,9)	(10,1,9)	(9,1,10)
66	(9,1,6)	(10,1,10)	NA	(10,1,10)	NA	(9,1,9)	NA	(10,1,8)
67	(9,1,9)	(9,1,9)	NA	(7,1,0)	NA	(10,1,8)	NA	(10,1,9)
68	(9,1,10)	(6,1,8)	(4,1,10)	(9,1,8)	(9,1,10)	(10,1,9)	(9,1,10)	(8,1,10)
69	(10,1,8)	(9,1,10)	(5,1,10)	(8,1,9)	(9,1,10)	(10,1,9)	(10,1,10)	(8,1,9)
70	(5,1,9)	(7,1,7)	(3,1,7)	NA	(9,1,10)	NA	(10,1,9)	NA
71	(7,1,6)	(10,1,3)	(2,1,6)	NA	(10,1,1)	NA	(10,1,6)	NA
72	(9,1,10)	(10,1,7)	(10,1,10)	(10,1,10)	(9,1,10)	(6,1,9)	(10,1,10)	(8,1,9)
73	(8,1,10)	(1,1,1)	(7,1,7)	(1,1,6)	(1,1,0)	(1,1,0)	(7,1,6)	(7,1,10)
74	(9,1,7)	(5,1,5)	NA	(10,1,10)	NA	(10,1,8)	NA	(10,1,8)
75	(10,1,10)	(10,1,10)	(8,1,10)	NA	(8,1,8)	NA	(8,1,8)	NA
76	(7,1,3)	(7,1,8)	(8,1,8)	NA	(7,1,8)	NA	(7,1,10)	NA
77	(10,1,5)	(9,1,10)	(9,1,9)	NA	(7,1,10)	NA	(10,1,10)	NA
78	(10,1,2)	(10,1,1)	NA	(3,1,2)	NA	(10,1,5)	NA	(8,1,5)
79	(9,1,10)	(8,1,6)	NA	(10,1,6)	NA	(8,1,9)	NA	(7,1,9)
80	(10,1,6)	(10,1,10)	(10,1,9)	(10,1,3)	(3,1,6)	(6,1,10)	(10,1,9)	(10,1,8)
81	(10,1,6)	(9,1,4)	(9,1,7)	(6,1,9)	(9,1,10)	(9,1,10)	(9,1,10)	(9,1,10)
82	(10,1,3)	(10,1,2)	(10,1,10)	(10,1,9)	(10,1,10)	(10,1,10)	(10,1,9)	(9,1,10)
83	(9,1,10)	(10,1,9)	(1,1,8)	(9,1,10)	(7,1,8)	(7,1,9)	(9,1,7)	(10,1,8)
84	(7,1,9)	(2,1,4)	(4,1,9)	(7,1,8)	(6,1,10)	(7,1,9)	(9,1,6)	(7,1,10)
85	(9,1,9)	(10,1,8)	NA	(9,1,10)	NA	(5,1,5)	NA	(7,1,5)
86	(10,1,9)	(9,1,9)	(2,1,9)	(5,1,8)	(6,1,9)	(5,1,5)	(4,1,4)	(10,1,5)
87	(3,1,10)	(10,1,6)	(9,1,4)	(5,1,9)	(7,1,8)	(9,1,10)	(7,1,9)	(9,1,8)
88	(10,1,7)	(5,1,7)	(10,1,10)	(10,1,8)	(10,1,5)	(7,1,4)	(10,1,1)	(10,1,9)
89	(7,1,9)	(7,1,10)	(9,1,9)	(10,1,10)	(6,1,9)	(8,1,10)	(10,1,10)	(6,1,6)
90	(10,1,7)	(10,1,8)	NA	(10,1,9)	NA	(10,1,9)	NA	(9,1,9)
91	(10,1,7)	(10,1,8)	(10,1,10)	(10,1,10)	(9,1,8)	(10,1,10)	(9,1,8)	(10,1,10)
92	(9,1,8)	(6,1,8)	NA	(8,1,8)	NA	(10,1,8)	NA	(8,1,9)
93	(10,1,10)	(10,1,10)	(7,1,10)	(7,1,7)	(7,1,9)	(10,1,10)	(9,1,8)	(10,1,10)
94	(8,1,10)	(10,1,10)	(9,1,8)	NA	(10,1,8)	NA	(7,1,8)	NA
95	(10,1,10)	(10,1,9)	(8,1,9)	(9,1,10)	(10,1,9)	(8,1,10)	(7,1,9)	(10,1,8)
96	(9,1,9)	(5,1,10)	(10,1,8)	(8,1,10)	(9,1,8)	(10,1,10)	(10,1,10)	(7,1,7)
97	(8,1,8)	(6,1,4)	(10,1,7)	NA	(8,1,8)	NA	(7,1,8)	NA
98	(10,1,7)	(5,1,9)	(10,1,10)	(10,1,10)	(6,1,4)	(7,1,8)	(4,1,4)	(5,1,9)
99	(9,1,10)	(10,1,10)	(8,1,10)	NA	(8,1,10)	NA	(8,1,7)	NA
100	(6,1,9)	(9,1,10)	(10,1,10)	(7,1,10)	(10,1,10)	(9,1,10)	(9,1,10)	(9,1,10)
101	(10,1,10)	(10,1,10)	(10,1,9)	NA	(9,1,10)	NA	(9,1,10)	NA

ABP, arterial blood pressure; AIC, Akaike Information Criterion; ARIMA, autoregressive integrative moving average; COx, cerebral oximetry index with CPP; COx-a, cerebral oximetry index with ABP; CPP, cerebral perfusion pressure; HC, healthy control volunteer group; rSO_2, regional cerebral oxygen saturation; SP, elective spinal surgery patient group; TBI, traumatic brain injury patient group.
