# Supplementary material for: Time-Series Autoregressive Models for Point and Interval Forecasting of Raw and Derived Commercial Near-Infrared Spectroscopy Measures: An Exploratory Cranial Trauma and Healthy Control Analysis
Source: Bioengineering (Basel). 2025 Jun 21;12(7):682. doi: 10.3390/bioengineering12070682 (PMC12292983; doi:10.3390/bioengineering12070682)
Supplement: Supplementary file 1 [file bioengineering-12-00682-s001.zip › File S4.pdf]

File S4 – Computational Duration Analysis

File S4 – Table of Contents

File S4a: Median and IQR of Data File Size using 10-Second, 1-Minute, and 5-Minute Temporal Resolutions in All Populations 2

File S4b: Median and IQR of Computational Duration of Forecast in All Populations Using 1-Minute Temporal Resolution 3

File S4c: Median and IQR of Computational Duration of Forecast in All Populations Using 5-Minute Temporal Resolution 4

File S4a: Median and IQR of Data File Size using 10-Second, 1-Minute, and 5-Minute Temporal Resolutions in All Populations

Temporal Resolution	File Size in Kilobytes (Median [IQR])		
	HC	SP	TBI
**10-Second**	9.2 [8.6 – 10.4]	59.2 [51.8 – 66.4]	1917.4 [792.3 – 2951.9]
**1-Minute**	2.1 [2.0 – 2.4]	13.1 [11.8 – 15.6]	485.4 [197.6 – 797.3]
**5-Minute**	0.5 [0.4 – 0.5]	2.9 [2.6 – 3.3]	102.7 [41.8 – 162.7]

KB, kilobytes; HC, healthy control volunteer group; IQR, interquartile range; SP, elective spinal surgery patient group; TBI, traumatic brain injury patient group.

File S4b: Median and IQR of Computational Duration of Forecast in All Populations Using 1-Minute Temporal Resolution

Forecast Method	Window / Interval	Computational Duration in Minutes (Median [IQR])		
		HC	SP	TBI
Anchored-Point	–	0 [0 – 0]	0 [0 – 0]	0 [0 – 0]
Anchored-Interval	5-Minute	0 [0 – 0]	0 [0 – 0]	79 [10 – 294]
	10-Minute	–	0 [0 – 0]	37 [6 – 135]
	15-Minute	–	0 [0 – 0]	21 [4 – 82]
	30-Minute	–	0 [0 – 0]	13 [2 – 47]
	1-Hour	–	0 [0 – 0]	5 [1 – 20]
	2-Hour	–	–	4 [1 – 12]
	6-Hour	–	–	2 [1 – 6]
	12-Hour	–	–	2 [1 – 4]
	1-Day	–	–	2.5 [1 – 4]
Windowed-Point	5-Minute	0 [0 – 0]	1 [0.5 – 1]	26 [9 – 52]
	10-Minute	0 [0 – 0]	2 [1 – 2]	53 [21 – 111]
	15-Minute	0 [0 – 0]	1 [1 – 2]	68 [23 – 154]
	30-Minute	0 [0 – 0]	1 [1 – 2]	77 [26 – 162]
	1-Hour	–	1 [0 – 2]	95 [31 – 198]
	2-Hour	–	1 [0 – 1]	127 [41 – 287]
	6-Hour	–	3 [2 – 4]	252 [69.5 – 591.5]
	12-Hour	–	–	400 [73 – 968]
	1-Day	–	–	711 [309.5 – 1570]
Windowed-Interval	5-Minute	0 [0 – 0]	0 [0 – 0]	8 [3 – 15]
	10-Minute	0 [0 – 0]	0 [0 – 0]	8 [3 – 17]
	15-Minute	0 [0 – 0]	0 [0 – 0]	7 [2 – 14]
	30-Minute	–	0 [0 – 0]	4 [1 – 7]
	1-Hour	–	0 [0 – 0]	2 [1 – 5]
	2-Hour	–	0 [0 – 0]	2 [1 – 3]
	6-Hour	–	0 [0 – 0]	1 [0 – 2]
	12-Hour	–	–	1 [0 – 2]
	1-Day	–	–	1 [0 – 2]

KB, kilobytes; HC, healthy control volunteer group; IQR, interquartile range; SP, elective spinal surgery patient group; TBI, traumatic brain injury patient group.

File S4c: Median and IQR of Computational Duration of Forecast in All Populations Using 5-Minute Temporal Resolution

Forecast Method	Window / Interval	Computational Duration in Minutes (Median [IQR])		
		HC	SP	TBI
Anchored-Point	–	0 [0 – 0]	0 [0 – 0]	0 [0 – 0]
Anchored-Interval	5-Minute	0 [0 – 0]	0 [0 – 0]	16 [2 – 53]
	10-Minute	–	0 [0 – 0]	8 [1 – 26]
	15-Minute	–	0 [0 – 0]	5 [1 – 17]
	30-Minute	–	0 [0 – 0]	2 [0 – 8]
	1-Hour	–	0 [0 – 0]	1 [0 – 4]
	2-Hour	–	–	1 [0 – 2]
	6-Hour	–	–	1 [0 – 1]
	12-Hour	–	–	0 [0 – 1]
	1-Day	–	–	0.5 [0 – 1]
Windowed-Point	5-Minute	0 [0 – 0]	0 [0 – 0]	0 [0 – 0]
	10-Minute	0 [0 – 0]	0 [0 – 0]	4 [1 – 7]
	15-Minute	0 [0 – 0]	0 [0 – 0]	4 [1 – 7]
	30-Minute	0 [0 – 0]	0 [0 – 0]	5 [2 – 12]
	1-Hour	–	0 [0 – 0]	9 [3 – 22]
	2-Hour	–	0 [0 – 0]	9 [2 – 22]
	6-Hour	–	0 [0 – 0]	11 [3 – 29.5]
	12-Hour	–	–	14 [3 – 38]
	1-Day	–	–	30 [7 – 67.5]
Windowed-Interval	5-Minute	0 [0 – 0]	0 [0 – 0]	0 [0 – 0]
	10-Minute	0 [0 – 0]	0 [0 – 0]	2 [1 – 6]
	15-Minute	0 [0 – 0]	0 [0 – 0]	2 [0 – 3]
	30-Minute	–	0 [0 – 0]	1 [0 – 3]
	1-Hour	–	0 [0 – 0]	1 [0 – 3]
	2-Hour	–	0 [0 – 0]	1 [0 – 1]
	6-Hour	–	0 [0 – 0]	0 [0 – 1]
	12-Hour	–	–	0 [0 – 0]
	1-Day	–	–	0 [0 – 0]

KB, kilobytes; HC, healthy control volunteer group; IQR, interquartile range; SP, elective spinal surgery patient group; TBI, traumatic brain injury patient group.
