# Supplementary material for: Time-Series Autoregressive Models for Point and Interval Forecasting of Raw and Derived Commercial Near-Infrared Spectroscopy Measures: An Exploratory Cranial Trauma and Healthy Control Analysis
Source: Bioengineering (Basel). 2025 Jun 21;12(7):682. doi: 10.3390/bioengineering12070682 (PMC12292983; doi:10.3390/bioengineering12070682)
Supplement: Supplementary file 1 [file bioengineering-12-00682-s001.zip › File S5.pdf]

File S5 – Absolute Forecast Residual Analysis

File S5 – Table of Contents

File S5a: Anchored-Point – Median Absolute Deviation of Absolute Forecast Residual of rSO_2 and COx/COx-a in all Populations using 10-Second Temporal Resolution 2

File S5b: Anchored-Point – Absolute Forecast Residual of rSO_2 and COx/COx-a in All Populations using 1-Minute and 5-Minute Temporal Resolutions 3

File S5c: Anchored-Interval – Median Absolute Deviation of Absolute Forecast Residual of rSO_2 and COx/COx-a in all Populations using 10-Second Temporal Resolution 4

File S5d: Anchored-Interval – Absolute Forecast Residual of rSO_2 and COx/COx-a in All Populations using 1-Minute Temporal Resolution 5

File S5e: Anchored-Interval – Absolute Forecast Residual of rSO_2 and COx/COx-a in All Populations using 5-Minute Temporal Resolution 6

File S5f: Windowed-Point – Median Absolute Deviation of Absolute Forecast Residual of rSO_2 and COx/COx-a in all Populations using 10-Second Temporal Resolution 7

File S5g: Windowed-Point – Absolute Forecast Residual of rSO_2 and COx/COx-a in All Populations using 1-Minute Temporal Resolution 8

File S5h: Windowed-Point – Absolute Forecast Residual of rSO_2 and COx/COx-a in All Populations using 5-Minute Temporal Resolution 9

File S5i: Windowed-Interval – Median Absolute Deviation of Absolute Forecast Residual of rSO_2 and COx/COx-a in all Populations using 10-Second Temporal Resolution 10

File S5j: Windowed-Interval – Absolute Forecast Residual of rSO_2 and COx/COx-a in All Populations using 1-Minute Temporal Resolution 11

File S5k: Windowed-Interval – Absolute Forecast Residual of rSO_2 and COx/COx-a in All Populations using 5-Minute Temporal Resolution 12

File S5a: Anchored-Point – Median Absolute Deviation of Absolute Forecast Residual of rSO_2 and COx/COx-a in all Populations using 10-Second Temporal Resolution

Physiologic Variable	Median [IQR]		
	HC	SP	TBI
MAD of AFR rSO_2_L	0.67 [0.5 – 0.91]	1.5 [1.25 – 3.27]	2.33 [1.48 – 2.98]
MAD of AFR rSO_2_R	0.73 [0.5 – 0.98]	1.5 [0.69 – 3.11]	2.24 [1.48 – 3.34]
MAD of AFR COx_L	–	–	0.21 [0.18 – 0.23]
MAD of AFR COx_R	–	–	0.2 [0.18 – 0.24]
MAD of AFR COx-a_L	0.12 [0.08 – 0.19]	0.26 [0.21 – 0.34]	0.19 [0.17 – 0.22]
MAD of AFR COx-a_R	0.12 [0.08 – 0.17]	0.26 [0.22 – 0.33]	0.19 [0.17 – 0.22]

AFR, absolute forecast residual; COx, cerebral oximetry index with cerebral perfusion pressure; COx-a, cerebral oximetry index with arterial blood pressure; HC, healthy control volunteer group; IQR, interquartile range; MAD, median absolute deviation; rSO2, regional cerebral oxygen saturation; SP, elective spinal surgery patient group; TBI, traumatic brain injury patient group.

File S5b: Anchored-Point – Absolute Forecast Residual of rSO_2 and COx/COx-a in All Populations using 1-Minute and 5-Minute Temporal Resolutions

Physiologic Variable	Median [IQR]		
	HC	SP	TBI
**1-Minute Temporal Resolution**			
ARF of rSO_2_L	0.77 [0.46 – 1.16]	2.24 [0.87 – 3.97]	2.83 [1.31 – 4.7]
ARF of rSO_2_R	0.82 [0.44 – 1.22]	1.6 [0.73 – 3.56]	3.28 [1.58 – 5.25]
ARF of COx_L	–	–	0.23 [0.11 – 0.39]
ARF of COx_R	–	–	0.22 [0.11 – 0.38]
ARF of COx-a_L	0.18 [0.11 – 0.28]	0.28 [0.14 – 0.46]	0.21 [0.1 – 0.36]
ARF of COx-a_R	0.18 [0.12 – 0.26]	0.35 [0.17 – 0.51]	0.21 [0.1 – 0.36]
MAD of ARF rSO_2_L	0.47 [0.29 – 0.76]	1.65 [1.17 – 3.31]	2.17 [1.5 – 3.33]
MAD of ARF rSO_2_R	0.49 [0.25 – 0.72]	1.47 [0.8 – 2.96]	2.21 [1.48 – 3.71]
MAD of ARF COx_L	–	–	0.2 [0.17 – 0.24]
MAD of ARF COx_R	–	–	0.2 [0.16 – 0.23]
MAD of ARF COx-a_L	0.11 [0.06 – 0.18]	0.24 [0.19 – 0.33]	0.19 [0.16 – 0.21]
MAD of ARF COx-a_R	0.1 [0.06 – 0.16]	0.26 [0.21 – 0.31]	0.19 [0.16 – 0.2]
**5-Minute Temporal Resolution**			
ARF of rSO_2_L	1.22 [0.92 – 1.5]	3.48 [1.45 – 4.42]	2.88 [1.45 – 4.86]
ARF of rSO_2_R	1.47 [1.24 – 1.59]	2.12 [0.82 – 3.3]	3.01 [1.49 – 4.78]
ARF of COx_L	–	–	0.19 [0.09 – 0.31]
ARF of COx_R	–	–	0.2 [0.09 – 0.32]
ARF of COx-a_L	0.28 [0.24 – 0.33]	0.24 [0.1 – 0.4]	0.17 [0.08 – 0.29]
ARF of COx-a_R	0.28 [0.23 – 0.35]	0.25 [0.16 – 0.43]	0.18 [0.08 – 0.3]
MAD of ARF rSO_2_L	0.44 [0 – 1.05]	1.76 [1.28 – 4.01]	2.4 [1.36 – 3.65]
MAD of ARF rSO_2_R	0.34 [0 – 0.9]	1.31 [0.73 – 3.42]	2 [1.32 – 3.24]
MAD of ARF COx_L	–	–	0.16 [0.14 – 0.19]
MAD of ARF COx_R	–	–	0.16 [0.14 – 0.19]
MAD of ARF COx-a_L	0.07 [0 – 0.2]	0.16 [0.12 – 0.24]	0.15 [0.13 – 0.17]
MAD of ARF COx-a_R	0.07 [0 – 0.18]	0.17 [0.13 – 0.27]	0.15 [0.14 – 0.17]

ARF, absolute forecast residual; COx, cerebral oximetry index with cerebral perfusion pressure; COx-a, cerebral oximetry index with arterial blood pressure; HC, healthy control volunteer group; IQR, interquartile range; MAD, median absolute deviation; rSO_2, regional cerebral oxygen saturation; SP, elective spinal surgery patient group; TBI, traumatic brain injury patient group.

File S5c: Anchored-Interval – Median Absolute Deviation of Absolute Forecast Residual of rSO_2 and COx/COx-a in all Populations using 10-Second Temporal Resolution

Physiologic Variable	Median [IQR]								
	5-Minute Interval	10-Minute Interval	15-Minute Interval	30-Minute Interval	1-Hour Interval	2-Hour Interval	6-Hour Interval	12-Hour Interval	1-Day Interval
**HC Population**									
MAD of AFR rSO_2_L	0.69 [0.49 – 0.87]	0.77 [0.77 – 0.77]	–	–	–	–	–	–	–
MAD of AFR rSO_2_R	0.7 [0.51 – 1.01]	1.31 [1.31 – 1.31]	–	–	–	–	–	–	–
MAD of AFR COx-a_L	0.12 [0.07 – 0.18]	0.08 [0.08 – 0.08]	–	–	–	–	–	–	–
MAD of AFR COx-a_R	0.11 [0.07 – 0.18]	0.1 [0.1 – 0.1]	–	–	–	–	–	–	–
**SP Population**									
MAD of AFR rSO_2_L	0.81 [0.56 – 1.21]	1.12 [0.94 – 1.52]	1.41 [1.05 – 1.75]	1.61 [0.96 – 3.01]	1.53 [1.5 – 1.68]	–	–	–	–
MAD of AFR rSO_2_R	0.88 [0.55 – 1.27]	1.23 [0.63 – 1.52]	1.32 [0.78 – 2.2]	1.53 [0.7 – 2.97]	0.8 [0.66 – 1.15]	–	–	–	–
MAD of AFR COx-a_L	0.21 [0.15 – 0.29]	0.26 [0.18 – 0.33]	0.22 [0.21 – 0.32]	0.26 [0.21 – 0.33]	0.22 [0.21 – 0.23]	–	–	–	–
MAD of AFR COx-a_R	0.21 [0.18 – 0.29]	0.25 [0.21 – 0.31]	0.25 [0.21 – 0.31]	0.25 [0.22 – 0.31]	0.25 [0.24 – 0.25]	–	–	–	–
**TBI Population**									
MAD of AFR rSO_2_L	0.57 [0.42 – 0.82]	0.74 [0.5 – 1.06]	0.83 [0.58 – 1.23]	1.1 [0.75 – 1.52]	1.43 [0.92 – 1.85]	1.57 [1.1 – 2.25]	2.15 [1.47 – 3.02]	2.36 [1.49 – 3.42]	2.94 [2.29 – 3.53]
MAD of AFR rSO_2_R	0.57 [0.38 – 0.77]	0.69 [0.46 – 1.03]	0.82 [0.53 – 1.21]	1.11 [0.72 – 1.49]	1.29 [0.85 – 1.95]	1.8 [1.18 – 2.44]	2.14 [1.53 – 3.22]	2.83 [1.76 – 3.41]	2.83 [1.84 – 5.9]
MAD of AFR COx_L	0.16 [0.15 – 0.18]	0.18 [0.16 – 0.21]	0.19 [0.17 – 0.21]	0.19 [0.17 – 0.22]	0.2 [0.18 – 0.22]	0.2 [0.18 – 0.22]	0.2 [0.18 – 0.23]	0.21 [0.18 – 0.23]	0.21 [0.19 – 0.24]
MAD of AFR COx_R	0.16 [0.14 – 0.18]	0.18 [0.15 – 0.2]	0.19 [0.16 – 0.21]	0.19 [0.16 – 0.22]	0.2 [0.17 – 0.22]	0.2 [0.17 – 0.23]	0.2 [0.17 – 0.23]	0.21 [0.18 – 0.23]	0.22 [0.19 – 0.25]
MAD of AFR COx-a_L	0.15 [0.06 – 0.3]	0.18 [0.08 – 0.33]	0.2 [0.09 – 0.35]	0.21 [0.09 – 0.37]	0.21 [0.1 – 0.36]	0.21 [0.1 – 0.37]	0.22 [0.1 – 0.38]	0.23 [0.11 – 0.39]	0.2 [0.09 – 0.37]
MAD of AFR COx-a_R	0.15 [0.13 – 0.17]	0.18 [0.15 – 0.2]	0.18 [0.16 – 0.2]	0.19 [0.16 – 0.21]	0.19 [0.16 – 0.21]	0.19 [0.17 – 0.21]	0.19 [0.17 – 0.22]	0.19 [0.17 – 0.21]	0.19 [0.18 – 0.22]

AFR, absolute forecast residual; COx, cerebral oximetry index with cerebral perfusion pressure; COx-a, cerebral oximetry index with arterial blood pressure; HC, healthy control volunteer group; IQR, interquartile range; MAD, median absolute deviation; rSO_2, regional cerebral oxygen saturation; SP, elective spinal surgery patient group; TBI, traumatic brain injury patient group.

File S5d: Anchored-Interval – Absolute Forecast Residual of rSO_2 and COx/COx-a in All Populations using 1-Minute Temporal Resolution

Physiologic Variable	Median [IQR]								
	5-Minute Interval	10-Minute Interval	15-Minute Interval	30-Minute Interval	1-Hour Interval	2-Hour Interval	6-Hour Interval	12-Hour Interval	1-Day Interval
**HC Population**									
ARF of rSO_2_L	0.66 [0.41 – 0.98]	–	–	–	–	–	–	–	–
ARF of rSO_2_R	0.79 [0.42 – 1.2]	–	–	–	–	–	–	–	–
ARF of COx-a_L	0.15 [0.1 – 0.25]	–	–	–	–	–	–	–	–
ARF of COx-a_R	0.17 [0.1 – 0.24]	–	–	–	–	–	–	–	–
MAD of ARF rSO_2_L	0.46 [0.28 – 0.64]	–	–	–	–	–	–	–	–
MAD of ARF rSO_2_R	0.52 [0.32 – 0.72]	–	–	–	–	–	–	–	–
MAD of ARF COx-a_L	0.1 [0.06 – 0.17]	–	–	–	–	–	–	–	–
MAD of ARF COx-a_R	0.1 [0.05 – 0.16]	–	–	–	–	–	–	–	–
**SP Population**									
ARF of rSO_2_L	0.83 [0.35 – 1.56]	1.03 [0.5 – 2.68]	1.45 [0.61 – 2.82]	2.22 [0.84 – 5.36]	2.63 [0.99 – 3.63]	–	–	–	–
ARF of rSO_2_R	0.77 [0.27 – 1.78]	1.15 [0.43 – 2.83]	1.19 [0.54 – 2.79]	1.6 [0.53 – 3.56]	0.82 [0.39 – 1.49]	–	–	–	–
ARF of COx-a_L	0.24 [0.11 – 0.41]	0.27 [0.12 – 0.44]	0.27 [0.11 – 0.49]	0.29 [0.13 – 0.49]	0.24 [0.13 – 0.44]	–	–	–	–
ARF of COx-a_R	0.23 [0.11 – 0.46]	0.28 [0.14 – 0.49]	0.26 [0.14 – 0.53]	0.33 [0.15 – 0.58]	0.28 [0.13 – 0.44]	–	–	–	–
MAD of ARF rSO_2_L	0.74 [0.61 – 1.03]	0.97 [0.78 – 1.36]	1.3 [0.73 – 2.29]	2.33 [0.91 – 3.86]	1.48 [1.32 – 2.69]	–	–	–	–
MAD of ARF rSO_2_R	0.75 [0.49 – 1.22]	0.95 [0.58 – 1.79]	1.33 [0.62 – 1.64]	1.76 [0.73 – 2.88]	0.86 [0.58 – 2.29]	–	–	–	–
MAD of ARF COx-a_L	0.23 [0.16 – 0.31]	0.25 [0.18 – 0.35]	0.24 [0.19 – 0.31]	0.27 [0.22 – 0.37]	0.26 [0.22 – 0.32]	–	–	–	–
MAD of ARF COx-a_R	0.2 [0.19 – 0.26]	0.23 [0.21 – 0.28]	0.23 [0.21 – 0.28]	0.26 [0.2 – 0.28]	0.25 [0.24 – 0.27]	–	–	–	–
**TBI Population**									
ARF of rSO_2_L	0.5 [0.22 – 1.05]	0.68 [0.28 – 1.4]	0.77 [0.32 – 1.65]	1.05 [0.43 – 2.14]	1.3 [0.55 – 2.77]	1.81 [0.76 – 3.46]	2.24 [1 – 4.19]	2.77 [1.27 – 5.03]	2.9 [1.39 – 5.87]
ARF of rSO_2_R	0.46 [0.2 – 0.97]	0.58 [0.23 – 1.24]	0.69 [0.29 – 1.55]	0.9 [0.38 – 2.1]	1.3 [0.55 – 2.79]	1.75 [0.74 – 3.82]	2.35 [1.1 – 4.18]	3.02 [1.33 – 4.89]	3.35 [1.62 – 6]
ARF of COx_L	0.16 [0.08 – 0.31]	0.19 [0.09 – 0.34]	0.2 [0.09 – 0.35]	0.21 [0.1 – 0.36]	0.21 [0.1 – 0.37]	0.21 [0.1 – 0.38]	0.23 [0.11 – 0.38]	0.23 [0.11 – 0.38]	0.23 [0.11 – 0.39]
ARF of COx_R	0.17 [0.07 – 0.3]	0.19 [0.09 – 0.34]	0.2 [0.09 – 0.35]	0.21 [0.1 – 0.36]	0.21 [0.1 – 0.37]	0.22 [0.1 – 0.37]	0.22 [0.1 – 0.38]	0.22 [0.1 – 0.38]	0.25 [0.12 – 0.42]
ARF of COx-a_L	0.16 [0.07 – 0.29]	0.18 [0.08 – 0.32]	0.19 [0.09 – 0.34]	0.19 [0.09 – 0.35]	0.2 [0.09 – 0.35]	0.21 [0.1 – 0.36]	0.21 [0.1 – 0.37]	0.21 [0.1 – 0.36]	0.19 [0.09 – 0.36]
ARF of COx-a_R	0.16 [0.07 – 0.28]	0.18 [0.08 – 0.32]	0.18 [0.08 – 0.33]	0.19 [0.09 – 0.34]	0.2 [0.09 – 0.35]	0.2 [0.09 – 0.35]	0.21 [0.09 – 0.36]	0.21 [0.1 – 0.36]	0.21 [0.1 – 0.36]
MAD of ARF rSO_2_L	0.52 [0.34 – 0.77]	0.71 [0.44 – 1.06]	0.82 [0.53 – 1.25]	1.06 [0.74 – 1.68]	1.39 [0.89 – 2.12]	1.72 [1.13 – 2.42]	1.99 [1.38 – 3.26]	2.41 [1.61 – 3.46]	2.7 [1.92 – 3.35]
MAD of ARF rSO_2_R	0.46 [0.29 – 0.71]	0.62 [0.38 – 0.96]	0.74 [0.48 – 1.08]	1.05 [0.65 – 1.48]	1.34 [0.84 – 1.94]	1.82 [1.16 – 2.59]	2.12 [1.58 – 3.06]	2.54 [1.85 – 3.42]	2.83 [1.73 – 5.8]
MAD of ARF COx_L	0.15 [0.14 – 0.18]	0.18 [0.15 – 0.2]	0.18 [0.16 – 0.2]	0.19 [0.16 – 0.21]	0.19 [0.17 – 0.22]	0.19 [0.17 – 0.22]	0.2 [0.17 – 0.22]	0.19 [0.18 – 0.23]	0.2 [0.18 – 0.23]
MAD of ARF COx_R	0.15 [0.14 – 0.17]	0.17 [0.15 – 0.2]	0.18 [0.15 – 0.2]	0.19 [0.16 – 0.21]	0.19 [0.16 – 0.21]	0.19 [0.16 – 0.22]	0.19 [0.16 – 0.22]	0.2 [0.17 – 0.22]	0.21 [0.19 – 0.23]
MAD of ARF COx-a_L	0.15 [0.13 – 0.17]	0.16 [0.15 – 0.19]	0.17 [0.15 – 0.19]	0.17 [0.15 – 0.2]	0.18 [0.16 – 0.2]	0.18 [0.16 – 0.21]	0.19 [0.17 – 0.21]	0.19 [0.17 – 0.21]	0.18 [0.16 – 0.21]
MAD of ARF COx-a_R	0.15 [0.13 – 0.17]	0.16 [0.15 – 0.19]	0.17 [0.15 – 0.19]	0.18 [0.16 – 0.2]	0.18 [0.16 – 0.2]	0.18 [0.16 – 0.2]	0.18 [0.16 – 0.21]	0.18 [0.17 – 0.21]	0.19 [0.18 – 0.21]

ARF, absolute forecast residual; COx, cerebral oximetry index with cerebral perfusion pressure; COx-a, cerebral oximetry index with arterial blood pressure; HC, healthy control volunteer group; IQR, interquartile range; MAD, median absolute deviation; rSO_2, regional cerebral oxygen saturation; SP, elective spinal surgery patient group; TBI, traumatic brain injury patient group.

File S5e: Anchored-Interval – Absolute Forecast Residual of rSO_2 and COx/COx-a in All Populations using 5-Minute Temporal Resolution

Physiologic Variable	Median [IQR]								
	5-Minute Interval	10-Minute Interval	15-Minute Interval	30-Minute Interval	1-Hour Interval	2-Hour Interval	6-Hour Interval	12-Hour Interval	1-Day Interval
**HC Population**									
ARF of rSO_2_L	1.24 [0.98 – 1.46]	–	–	–	–	–	–	–	–
ARF of rSO_2_R	1.29 [1.08 – 1.54]	–	–	–	–	–	–	–	–
ARF of COx-a_L	0.27 [0.24 – 0.3]	–	–	–	–	–	–	–	–
ARF of COx-a_R	0.29 [0.23 – 0.34]	–	–	–	–	–	–	–	–
MAD of ARF rSO_2_L	0.24 [0 – 0.97]	–	–	–	–	–	–	–	–
MAD of ARF rSO_2_R	0.32 [0 – 0.84]	–	–	–	–	–	–	–	–
MAD of ARF COx-a_L	0.04 [0 – 0.2]	–	–	–	–	–	–	–	–
MAD of ARF COx-a_R	0.03 [0 – 0.2]	–	–	–	–	–	–	–	–
**SP Population**									
ARF of rSO_2_L	1.28 [0.74 – 2.21]	1.62 [0.89 – 2.81]	1.62 [1.15 – 4.01]	1.76 [1.15 – 3.26]	1.32 [0.71 – 4.07]	–	–	–	–
ARF of rSO_2_R	1.06 [0.56 – 2.11]	1.13 [0.49 – 2.14]	1.45 [0.61 – 2.95]	1.13 [0.61 – 2.52]	1.07 [0.51 – 2.04]	–	–	–	–
ARF of COx-a_L	0.22 [0.16 – 0.35]	0.22 [0.12 – 0.37]	0.22 [0.16 – 0.36]	0.21 [0.09 – 0.36]	0.22 [0.11 – 0.36]	–	–	–	–
ARF of COx-a_R	0.23 [0.14 – 0.35]	0.22 [0.13 – 0.36]	0.25 [0.15 – 0.43]	0.23 [0.13 – 0.42]	0.17 [0.07 – 0.39]	–	–	–	–
MAD of ARF rSO_2_L	0.95 [0.66 – 1.31]	1.3 [0.74 – 2.21]	1.73 [0.82 – 2.95]	1.51 [0.7 – 2.56]	1.52 [1.25 – 1.55]	–	–	–	–
MAD of ARF rSO_2_R	0.83 [0.44 – 1.48]	0.97 [0.57 – 1.6]	1.06 [0.72 – 1.93]	0.99 [0.56 – 2.25]	0.91 [0.68 – 0.97]	–	–	–	–
MAD of ARF COx-a_L	0.15 [0.12 – 0.21]	0.16 [0.12 – 0.26]	0.15 [0.12 – 0.19]	0.17 [0.14 – 0.24]	0.19 [0.15 – 0.21]	–	–	–	–
MAD of ARF COx-a_R	0.16 [0.11 – 0.19]	0.13 [0.09 – 0.2]	0.16 [0.1 – 0.27]	0.15 [0.12 – 0.25]	0.21 [0.16 – 0.22]	–	–	–	–
**TBI Population**									
ARF of rSO_2_L	0.62 [0.28 – 1.26]	0.78 [0.35 – 1.55]	0.91 [0.4 – 1.74]	1.04 [0.52 – 2.22]	1.32 [0.6 – 2.74]	1.8 [0.71 – 3.4]	2.25 [1.08 – 4.14]	2.79 [1.26 – 4.82]	2.9 [1.4 – 6.42]
ARF of rSO_2_R	0.62 [0.26 – 1.12]	0.72 [0.34 – 1.51]	0.87 [0.38 – 1.82]	1.08 [0.47 – 2.3]	1.26 [0.61 – 2.9]	1.75 [0.76 – 3.51]	2.47 [1.21 – 4.56]	2.99 [1.36 – 5.02]	3.33 [1.73 – 5.51]
ARF of COx_L	0.17 [0.08 – 0.29]	0.17 [0.08 – 0.29]	0.17 [0.09 – 0.31]	0.18 [0.09 – 0.3]	0.18 [0.09 – 0.3]	0.19 [0.09 – 0.3]	0.19 [0.09 – 0.32]	0.19 [0.1 – 0.32]	0.2 [0.09 – 0.34]
ARF of COx_R	0.17 [0.08 – 0.29]	0.17 [0.08 – 0.3]	0.18 [0.08 – 0.3]	0.18 [0.08 – 0.31]	0.18 [0.09 – 0.31]	0.19 [0.09 – 0.31]	0.19 [0.09 – 0.32]	0.19 [0.09 – 0.31]	0.2 [0.1 – 0.35]
ARF of COx-a_L	0.16 [0.08 – 0.27]	0.16 [0.08 – 0.28]	0.16 [0.08 – 0.29]	0.17 [0.08 – 0.28]	0.17 [0.08 – 0.29]	0.17 [0.08 – 0.29]	0.17 [0.08 – 0.3]	0.18 [0.08 – 0.3]	0.17 [0.08 – 0.3]
ARF of COx-a_R	0.15 [0.07 – 0.27]	0.16 [0.07 – 0.28]	0.16 [0.07 – 0.29]	0.16 [0.07 – 0.29]	0.16 [0.08 – 0.29]	0.17 [0.08 – 0.29]	0.17 [0.08 – 0.29]	0.17 [0.08 – 0.3]	0.17 [0.08 – 0.3]
MAD of ARF rSO_2_L	0.62 [0.38 – 0.9]	0.79 [0.49 – 1.13]	0.86 [0.55 – 1.37]	1.13 [0.7 – 1.67]	1.31 [0.86 – 2.06]	1.66 [1.09 – 2.62]	1.9 [1.32 – 3.05]	2.28 [1.53 – 3.37]	2.7 [1.95 – 3.75]
MAD of ARF rSO_2_R	0.57 [0.33 – 0.9]	0.71 [0.42 – 1.11]	0.85 [0.5 – 1.34]	1.12 [0.68 – 1.52]	1.29 [0.77 – 1.99]	1.73 [1.07 – 2.44]	2.07 [1.47 – 2.98]	2.44 [1.75 – 3.73]	2.6 [1.89 – 5.59]
MAD of ARF COx_L	0.14 [0.12 – 0.16]	0.15 [0.13 – 0.17]	0.15 [0.13 – 0.18]	0.16 [0.13 – 0.18]	0.15 [0.14 – 0.19]	0.16 [0.14 – 0.19]	0.16 [0.15 – 0.19]	0.16 [0.14 – 0.19]	0.17 [0.15 – 0.19]
MAD of ARF COx_R	0.15 [0.13 – 0.16]	0.15 [0.13 – 0.17]	0.16 [0.13 – 0.18]	0.16 [0.13 – 0.18]	0.16 [0.13 – 0.18]	0.16 [0.14 – 0.19]	0.16 [0.14 – 0.19]	0.16 [0.14 – 0.19]	0.17 [0.16 – 0.2]
MAD of ARF COx-a_L	0.14 [0.12 – 0.16]	0.14 [0.13 – 0.16]	0.14 [0.13 – 0.16]	0.15 [0.13 – 0.16]	0.15 [0.13 – 0.16]	0.15 [0.13 – 0.16]	0.15 [0.14 – 0.17]	0.15 [0.13 – 0.17]	0.15 [0.13 – 0.17]
MAD of ARF COx-a_R	0.14 [0.12 – 0.16]	0.14 [0.12 – 0.16]	0.15 [0.13 – 0.16]	0.15 [0.13 – 0.17]	0.15 [0.13 – 0.17]	0.15 [0.13 – 0.17]	0.15 [0.13 – 0.17]	0.15 [0.14 – 0.17]	0.15 [0.14 – 0.17]

ARF, absolute forecast residual; COx, cerebral oximetry index with cerebral perfusion pressure; COx-a, cerebral oximetry index with arterial blood pressure; HC, healthy control volunteer group; IQR, interquartile range; MAD, median absolute deviation; rSO_2, regional cerebral oxygen saturation; SP, elective spinal surgery patient group; TBI, traumatic brain injury patient group.

File S5f: Windowed-Point – Median Absolute Deviation of Absolute Forecast Residual of rSO_2 and COx/COx-a in all Populations using 10-Second Temporal Resolution

Physiologic Variable	Median [IQR]								
	5-Minute Window	10-Minute Window	15-Minute Window	30-Minute Window	1-Hour Window	2-Hour Window	6-Hour Window	12-Hour Window	1-Day Window
**HC Population**									
MAD of AFR rSO_2_L	0.48 [0.35 – 0.56]	0.42 [0.33 – 0.52]	0.41 [0.32 – 0.52]	0.37 [0.25 – 0.56]	–	–	–	–	–
MAD of AFR rSO_2_R	0.5 [0.4 – 0.65]	0.46 [0.37 – 0.57]	0.46 [0.38 – 0.57]	0.39 [0.3 – 0.52]	–	–	–	–	–
MAD of AFR COx-a_L	0.03 [0.03 – 0.04]	0.03 [0.03 – 0.03]	0.03 [0.02 – 0.03]	0.03 [0.02 – 0.03]	–	–	–	–	–
MAD of AFR COx-a_R	0.03 [0.03 – 0.04]	0.03 [0.03 – 0.03]	0.03 [0.02 – 0.03]	0.02 [0.02 – 0.03]	–	–	–	–	–
**SP Population**									
MAD of AFR rSO_2_L	0.29 [0.24 – 0.36]	0.27 [0.2 – 0.33]	0.25 [0.19 – 0.32]	0.24 [0.14 – 0.32]	0.25 [0.12 – 0.3]	0.22 [0.11 – 0.3]	0.3 [0.29 – 0.31]	–	–
MAD of AFR rSO_2_R	0.29 [0.24 – 0.37]	0.27 [0.2 – 0.34]	0.26 [0.18 – 0.32]	0.23 [0.17 – 0.31]	0.23 [0.15 – 0.27]	0.24 [0.19 – 0.31]	0.15 [0.1 – 0.2]	–	–
MAD of AFR COx-a_L	0.04 [0.04 – 0.04]	0.03 [0.03 – 0.04]	0.03 [0.03 – 0.03]	0.03 [0.03 – 0.03]	0.03 [0.03 – 0.03]	0.03 [0.02 – 0.03]	0.03 [0.03 – 0.03]	–	–
MAD of AFR COx-a_R	0.04 [0.04 – 0.04]	0.03 [0.03 – 0.04]	0.03 [0.03 – 0.03]	0.03 [0.03 – 0.03]	0.03 [0.03 – 0.03]	0.03 [0.02 – 0.03]	0.03 [0.03 – 0.03]	–	–
**TBI Population**									
MAD of AFR rSO_2_L	0.33 [0.28 – 0.39]	0.29 [0.23 – 0.35]	0.28 [0.22 – 0.33]	0.27 [0.19 – 0.31]	0.27 [0.17 – 0.31]	0.27 [0.15 – 0.32]	0.34 [0.27 – 0.4]	0.35 [0.31 – 0.42]	0.36 [0.32 – 0.43]
MAD of AFR rSO_2_R	0.31 [0.26 – 0.39]	0.27 [0.22 – 0.34]	0.26 [0.19 – 0.33]	0.25 [0.17 – 0.32]	0.24 [0.16 – 0.32]	0.24 [0.15 – 0.32]	0.27 [0.24 – 0.35]	0.31 [0.2 – 0.41]	0.27 [0.17 – 0.38]
MAD of AFR COx_L	0.04 [0.04 – 0.05]	0.04 [0.04 – 0.04]	0.03 [0.03 – 0.04]	0.03 [0.03 – 0.03]	0.03 [0.03 – 0.03]	0.03 [0.03 – 0.03]	0.03 [0.03 – 0.03]	0.03 [0.03 – 0.03]	0.03 [0.03 – 0.03]
MAD of AFR COx_R	0.04 [0.04 – 0.05]	0.04 [0.04 – 0.04]	0.03 [0.03 – 0.04]	0.03 [0.03 – 0.03]	0.03 [0.03 – 0.03]	0.03 [0.03 – 0.03]	0.03 [0.03 – 0.03]	0.03 [0.03 – 0.03]	0.03 [0.03 – 0.03]
MAD of AFR COx-a_L	0.04 [0.04 – 0.05]	0.04 [0.04 – 0.04]	0.03 [0.03 – 0.04]	0.03 [0.03 – 0.03]	0.03 [0.03 – 0.03]	0.03 [0.03 – 0.03]	0.03 [0.03 – 0.03]	0.03 [0.03 – 0.03]	0.03 [0.02 – 0.03]
MAD of AFR COx-a_R	0.04 [0.04 – 0.05]	0.04 [0.04 – 0.04]	0.03 [0.03 – 0.04]	0.03 [0.03 – 0.03]	0.03 [0.03 – 0.03]	0.03 [0.03 – 0.03]	0.03 [0.03 – 0.03]	0.03 [0.02 – 0.03]	0.03 [0.03 – 0.03]

AFR, absolute forecast residual; COx, cerebral oximetry index with cerebral perfusion pressure; COx-a, cerebral oximetry index with arterial blood pressure; HC, healthy control volunteer group; IQR, interquartile range; MAD, median absolute deviation; rSO_2, regional cerebral oxygen saturation; SP, elective spinal surgery patient group; TBI, traumatic brain injury patient group.

File S5g: Windowed-Point – Absolute Forecast Residual of rSO_2 and COx/COx-a in All Populations using 1-Minute Temporal Resolution

Physiologic Variable	Median [IQR]								
	5-Minute Window	10-Minute Window	15-Minute Window	30-Minute Window	1-Hour Window	2-Hour Window	6-Hour Window	12-Hour Window	1-Day Window
**HC Population**									
ARF of rSO_2_L	0.76 [0.35 – 1.3]	0.74 [0.35 – 1.15]	0.63 [0.34 – 1.03]	0.73 [0.39 – 1]	–	–	–	–	–
ARF of rSO_2_R	0.79 [0.4 – 1.46]	0.79 [0.37 – 1.3]	0.69 [0.36 – 1.16]	0.63 [0.4 – 0.94]	–	–	–	–	–
ARF of COx-a_L	0.16 [0.08 – 0.28]	0.13 [0.07 – 0.22]	0.12 [0.06 – 0.21]	0.11 [0.07 – 0.17]	–	–	–	–	–
ARF of COx-a_R	0.15 [0.07 – 0.25]	0.14 [0.07 – 0.22]	0.12 [0.06 – 0.2]	0.12 [0.09 – 0.17]	–	–	–	–	–
MAD of ARF rSO_2_L	0.65 [0.52 – 0.85]	0.6 [0.45 – 0.8]	0.54 [0.35 – 0.71]	0.39 [0.13 – 0.63]	–	–	–	–	–
MAD of ARF rSO_2_R	0.7 [0.56 – 0.89]	0.7 [0.49 – 0.87]	0.6 [0.41 – 0.75]	0.27 [0.1 – 0.54]	–	–	–	–	–
MAD of ARF COx-a_L	0.12 [0.1 – 0.17]	0.11 [0.08 – 0.16]	0.09 [0.07 – 0.13]	0.05 [0.02 – 0.13]	–	–	–	–	–
MAD of ARF COx-a_R	0.13 [0.1 – 0.16]	0.11 [0.09 – 0.14]	0.09 [0.07 – 0.12]	0.07 [0.01 – 0.1]	–	–	–	–	–
**SP Population**									
ARF of rSO_2_L	0.43 [0.2 – 0.9]	0.42 [0.18 – 0.84]	0.38 [0.17 – 0.78]	0.35 [0.16 – 0.67]	0.33 [0.15 – 0.61]	0.4 [0.16 – 0.79]	1.21 [0.43 – 2.68]	–	–
ARF of rSO_2_R	0.47 [0.21 – 0.94]	0.45 [0.21 – 0.91]	0.4 [0.17 – 0.83]	0.37 [0.16 – 0.73]	0.34 [0.15 – 0.69]	0.36 [0.15 – 0.7]	0.67 [0.3 – 2.47]	–	–
ARF of COx-a_L	0.19 [0.09 – 0.35]	0.19 [0.08 – 0.33]	0.15 [0.07 – 0.31]	0.14 [0.07 – 0.26]	0.13 [0.06 – 0.23]	0.14 [0.07 – 0.23]	0.14 [0.08 – 0.26]	–	–
ARF of COx-a_R	0.21 [0.1 – 0.36]	0.19 [0.09 – 0.33]	0.17 [0.08 – 0.31]	0.15 [0.07 – 0.27]	0.13 [0.06 – 0.24]	0.13 [0.06 – 0.22]	0.15 [0.1 – 0.22]	–	–
MAD of ARF rSO_2_L	0.47 [0.38 – 0.63]	0.44 [0.37 – 0.62]	0.41 [0.35 – 0.58]	0.34 [0.29 – 0.48]	0.31 [0.25 – 0.35]	0.34 [0.28 – 0.48]	1.43 [0.91 – 1.95]	–	–
MAD of ARF rSO_2_R	0.44 [0.36 – 0.6]	0.44 [0.39 – 0.56]	0.41 [0.36 – 0.59]	0.36 [0.3 – 0.45]	0.32 [0.25 – 0.4]	0.34 [0.27 – 0.55]	0.66 [0.47 – 0.86]	–	–
MAD of ARF COx-a_L	0.18 [0.16 – 0.22]	0.16 [0.13 – 0.19]	0.15 [0.12 – 0.18]	0.14 [0.11 – 0.15]	0.11 [0.1 – 0.15]	0.11 [0.1 – 0.15]	0.12 [0.12 – 0.12]	–	–
MAD of ARF COx-a_R	0.19 [0.17 – 0.22]	0.17 [0.15 – 0.21]	0.16 [0.14 – 0.19]	0.14 [0.11 – 0.17]	0.12 [0.1 – 0.14]	0.11 [0.09 – 0.14]	0.08 [0.07 – 0.09]	–	–
**TBI Population**									
ARF of rSO_2_L	0.32 [0.13 – 0.67]	0.43 [0.18 – 0.89]	0.4 [0.17 – 0.83]	0.33 [0.14 – 0.69]	0.31 [0.13 – 0.63]	0.3 [0.13 – 0.62]	0.29 [0.13 – 0.59]	0.3 [0.12 – 0.59]	0.29 [0.11 – 0.59]
ARF of rSO_2_R	0.3 [0.11 – 0.6]	0.4 [0.17 – 0.82]	0.38 [0.16 – 0.78]	0.31 [0.13 – 0.65]	0.29 [0.12 – 0.58]	0.27 [0.12 – 0.54]	0.26 [0.11 – 0.52]	0.26 [0.1 – 0.54]	0.27 [0.11 – 0.55]
ARF of COx_L	0.16 [0.07 – 0.28]	0.16 [0.07 – 0.29]	0.14 [0.06 – 0.25]	0.12 [0.05 – 0.21]	0.11 [0.05 – 0.2]	0.1 [0.05 – 0.18]	0.09 [0.04 – 0.17]	0.09 [0.04 – 0.17]	0.09 [0.04 – 0.17]
ARF of COx_R	0.16 [0.07 – 0.29]	0.15 [0.07 – 0.27]	0.14 [0.06 – 0.26]	0.12 [0.06 – 0.22]	0.11 [0.05 – 0.19]	0.1 [0.05 – 0.18]	0.09 [0.04 – 0.17]	0.09 [0.04 – 0.17]	0.09 [0.04 – 0.16]
ARF of COx-a_L	0.15 [0.07 – 0.27]	0.15 [0.07 – 0.28]	0.13 [0.06 – 0.24]	0.11 [0.05 – 0.21]	0.1 [0.05 – 0.19]	0.1 [0.04 – 0.17]	0.09 [0.04 – 0.17]	0.09 [0.04 – 0.16]	0.09 [0.04 – 0.16]
ARF of COx-a_R	0.15 [0.07 – 0.28]	0.16 [0.07 – 0.29]	0.14 [0.06 – 0.26]	0.12 [0.05 – 0.21]	0.1 [0.05 – 0.19]	0.1 [0.04 – 0.18]	0.09 [0.04 – 0.17]	0.09 [0.04 – 0.16]	0.09 [0.04 – 0.16]
MAD of ARF rSO_2_L	0.34 [0.26 – 0.42]	0.43 [0.32 – 0.56]	0.42 [0.29 – 0.58]	0.33 [0.26 – 0.47]	0.32 [0.23 – 0.41]	0.32 [0.22 – 0.4]	0.29 [0.21 – 0.4]	0.3 [0.22 – 0.39]	0.3 [0.22 – 0.39]
MAD of ARF rSO_2_R	0.31 [0.24 – 0.42]	0.41 [0.31 – 0.51]	0.39 [0.28 – 0.51]	0.31 [0.25 – 0.42]	0.29 [0.23 – 0.4]	0.27 [0.21 – 0.38]	0.26 [0.2 – 0.37]	0.26 [0.21 – 0.37]	0.28 [0.21 – 0.38]
MAD of ARF COx_L	0.14 [0.13 – 0.16]	0.15 [0.12 – 0.17]	0.13 [0.11 – 0.15]	0.11 [0.1 – 0.12]	0.1 [0.09 – 0.11]	0.09 [0.08 – 0.1]	0.09 [0.08 – 0.1]	0.08 [0.08 – 0.09]	0.08 [0.08 – 0.09]
MAD of ARF COx_R	0.15 [0.13 – 0.16]	0.14 [0.12 – 0.17]	0.13 [0.11 – 0.15]	0.11 [0.1 – 0.12]	0.1 [0.09 – 0.11]	0.09 [0.08 – 0.1]	0.09 [0.08 – 0.09]	0.08 [0.08 – 0.09]	0.08 [0.08 – 0.09]
MAD of ARF COx-a_L	0.14 [0.13 – 0.16]	0.14 [0.12 – 0.16]	0.12 [0.11 – 0.14]	0.11 [0.1 – 0.12]	0.1 [0.09 – 0.11]	0.09 [0.08 – 0.1]	0.08 [0.08 – 0.09]	0.08 [0.08 – 0.09]	0.08 [0.08 – 0.09]
MAD of ARF COx-a_R	0.14 [0.12 – 0.15]	0.15 [0.12 – 0.17]	0.13 [0.11 – 0.15]	0.11 [0.1 – 0.12]	0.1 [0.09 – 0.11]	0.09 [0.08 – 0.1]	0.08 [0.08 – 0.09]	0.08 [0.08 – 0.09]	0.08 [0.08 – 0.09]

ARF, absolute forecast residual; COx, cerebral oximetry index with cerebral perfusion pressure; COx-a, cerebral oximetry index with arterial blood pressure; HC, healthy control volunteer group; IQR, interquartile range; MAD, median absolute deviation; rSO_2, regional cerebral oxygen saturation; SP, elective spinal surgery patient group; TBI, traumatic brain injury patient group.

File S5h: Windowed-Point – Absolute Forecast Residual of rSO_2 and COx/COx-a in All Populations using 5-Minute Temporal Resolution

Physiologic Variable	Median [IQR]							
	10-Minute Window	15-Minute Window	30-Minute Window	1-Hour Window	2-Hour Window	6-Hour Window	12-Hour Window	1-Day Window
**HC Population**								
ARF of rSO_2_L	0.69 [0.47 – 1.08]	0.89 [0.64 – 1.03]	1.89 [1.72 – 2.03]	–	–	–	–	–
ARF of rSO_2_R	0.78 [0.47 – 1.15]	1.05 [0.77 – 1.31]	1.68 [1.55 – 1.91]	–	–	–	–	–
ARF of COx-a_L	0.21 [0.16 – 0.27]	0.23 [0.17 – 0.29]	0.28 [0.28 – 0.3]	–	–	–	–	–
ARF of COx-a_R	0.21 [0.15 – 0.29]	0.23 [0.17 – 0.32]	0.3 [0.27 – 0.32]	–	–	–	–	–
MAD of ARF rSO_2_L	0.39 [0.21 – 0.55]	0.28 [0.08 – 0.71]	0 [0 – 0.95]	–	–	–	–	–
MAD of ARF rSO_2_R	0.37 [0.19 – 0.78]	0.37 [0.13 – 0.85]	0 [0 – 0.29]	–	–	–	–	–
MAD of ARF COx-a_L	0.09 [0.02 – 0.15]	0.08 [0.03 – 0.16]	0 [0 – 0.05]	–	–	–	–	–
MAD of ARF COx-a_R	0.09 [0.05 – 0.16]	0.08 [0.03 – 0.16]	0 [0 – 0.01]	–	–	–	–	–
**SP Population**								
ARF of rSO_2_L	0.92 [0.44 – 1.78]	0.97 [0.39 – 2.2]	0.96 [0.37 – 2.07]	0.79 [0.37 – 1.57]	0.84 [0.37 – 1.77]	3.05 [0.43 – 12.56]	–	–
ARF of rSO_2_R	0.8 [0.36 – 1.5]	0.88 [0.38 – 1.96]	0.86 [0.38 – 1.69]	0.7 [0.33 – 1.39]	0.75 [0.36 – 1.63]	2.37 [0.47 – 24.41]	–	–
ARF of COx-a_L	0.29 [0.13 – 0.46]	0.29 [0.15 – 0.52]	0.27 [0.13 – 0.47]	0.26 [0.13 – 0.44]	0.24 [0.13 – 0.33]	0.2 [0.15 – 0.32]	–	–
ARF of COx-a_R	0.28 [0.12 – 0.42]	0.31 [0.14 – 0.5]	0.3 [0.13 – 0.47]	0.27 [0.13 – 0.43]	0.25 [0.15 – 0.38]	0.16 [0.08 – 0.25]	–	–
MAD of ARF rSO_2_L	0.83 [0.57 – 1.22]	1.03 [0.71 – 1.35]	0.86 [0.58 – 1.16]	0.8 [0.46 – 1.36]	0.77 [0.51 – 1.14]	4.38 [2.64 – 6.11]	–	–
MAD of ARF rSO_2_R	0.75 [0.63 – 1.01]	0.87 [0.65 – 1.35]	0.68 [0.52 – 1.19]	0.67 [0.48 – 1.15]	0.6 [0.43 – 1.12]	3.15 [1.98 – 4.33]	–	–
MAD of ARF COx-a_L	0.22 [0.18 – 0.28]	0.25 [0.22 – 0.37]	0.23 [0.18 – 0.28]	0.22 [0.18 – 0.29]	0.18 [0.12 – 0.2]	0.08 [0.07 – 0.09]	–	–
MAD of ARF COx-a_R	0.2 [0.18 – 0.24]	0.25 [0.21 – 0.29]	0.24 [0.19 – 0.32]	0.23 [0.16 – 0.28]	0.18 [0.14 – 0.25]	0.15 [0.14 – 0.16]	–	–
**TBI Population**								
ARF of rSO_2_L	0.47 [0.19 – 1.11]	0.49 [0.19 – 1.14]	0.74 [0.29 – 1.67]	0.81 [0.34 – 1.78]	0.73 [0.33 – 1.49]	0.61 [0.27 – 1.26]	0.64 [0.27 – 1.26]	0.61 [0.27 – 1.2]
ARF of rSO_2_R	0.42 [0.17 – 0.91]	0.45 [0.17 – 1.03]	0.64 [0.25 – 1.51]	0.68 [0.27 – 1.49]	0.54 [0.24 – 1.31]	0.48 [0.21 – 1.07]	0.49 [0.21 – 1.1]	0.53 [0.24 – 1.14]
ARF of COx_L	0.19 [0.09 – 0.33]	0.23 [0.11 – 0.41]	0.25 [0.12 – 0.45]	0.22 [0.1 – 0.39]	0.2 [0.09 – 0.34]	0.17 [0.08 – 0.3]	0.17 [0.08 – 0.28]	0.17 [0.08 – 0.29]
ARF of COx_R	0.19 [0.09 – 0.33]	0.23 [0.11 – 0.41]	0.24 [0.12 – 0.42]	0.21 [0.1 – 0.38]	0.19 [0.09 – 0.34]	0.17 [0.08 – 0.29]	0.17 [0.08 – 0.29]	0.16 [0.08 – 0.28]
ARF of COx-a_L	0.19 [0.09 – 0.33]	0.23 [0.1 – 0.4]	0.25 [0.11 – 0.43]	0.21 [0.1 – 0.37]	0.19 [0.09 – 0.33]	0.17 [0.08 – 0.29]	0.16 [0.08 – 0.28]	0.16 [0.08 – 0.28]
ARF of COx-a_R	0.18 [0.08 – 0.32]	0.22 [0.1 – 0.39]	0.24 [0.12 – 0.43]	0.23 [0.11 – 0.4]	0.19 [0.09 – 0.34]	0.17 [0.08 – 0.3]	0.16 [0.08 – 0.28]	0.16 [0.07 – 0.27]
MAD of ARF rSO_2_L	0.52 [0.37 – 0.69]	0.54 [0.35 – 0.73]	0.81 [0.53 – 1.11]	0.85 [0.6 – 1.23]	0.73 [0.51 – 1.1]	0.6 [0.38 – 0.9]	0.62 [0.38 – 0.82]	0.6 [0.35 – 0.8]
MAD of ARF rSO_2_R	0.45 [0.34 – 0.68]	0.5 [0.35 – 0.67]	0.68 [0.44 – 1.06]	0.71 [0.48 – 1.09]	0.59 [0.43 – 0.88]	0.52 [0.37 – 0.8]	0.54 [0.36 – 0.76]	0.55 [0.33 – 0.86]
MAD of ARF COx_L	0.17 [0.16 – 0.19]	0.21 [0.19 – 0.23]	0.22 [0.19 – 0.26]	0.2 [0.18 – 0.23]	0.18 [0.16 – 0.2]	0.15 [0.14 – 0.17]	0.15 [0.14 – 0.16]	0.15 [0.14 – 0.16]
MAD of ARF COx_R	0.17 [0.15 – 0.19]	0.2 [0.19 – 0.23]	0.21 [0.19 – 0.25]	0.19 [0.17 – 0.24]	0.17 [0.15 – 0.19]	0.15 [0.14 – 0.17]	0.15 [0.13 – 0.16]	0.15 [0.13 – 0.16]
MAD of ARF COx-a_L	0.17 [0.16 – 0.19]	0.21 [0.19 – 0.22]	0.22 [0.19 – 0.25]	0.19 [0.17 – 0.24]	0.17 [0.15 – 0.19]	0.15 [0.14 – 0.17]	0.14 [0.13 – 0.16]	0.14 [0.13 – 0.15]
MAD of ARF COx-a_R	0.16 [0.15 – 0.18]	0.2 [0.18 – 0.23]	0.22 [0.2 – 0.25]	0.2 [0.18 – 0.25]	0.18 [0.16 – 0.2]	0.15 [0.14 – 0.16]	0.14 [0.13 – 0.16]	0.14 [0.13 – 0.15]

ARF, absolute forecast residual; COx, cerebral oximetry index with cerebral perfusion pressure; COx-a, cerebral oximetry index with arterial blood pressure; HC, healthy control volunteer group; IQR, interquartile range; MAD, median absolute deviation; rSO_2, regional cerebral oxygen saturation; SP, elective spinal surgery patient group; TBI, traumatic brain injury patient group.

File S5i: Windowed-Interval – Median Absolute Deviation of Absolute Forecast Residual of rSO_2 and COx/COx-a in all Populations using 10-Second Temporal Resolution

Physiologic Variable	Median [IQR]								
	5-Minute W&I	10-Minute W&I	15-Minute W&I	30-Minute W&I	1-Hour W&I	2-Hour W&I	6-Hour W&I	12-Hour W&I	1-Day W&I
**HC Population**									
MAD of AFR rSO_2_L	0.87 [0.7 – 1.16]	0.9 [0.7 – 1.21]	0.97 [0.71 – 1.22]	–	–	–	–	–	–
MAD of AFR rSO_2_R	0.91 [0.74 – 1.19]	1.01 [0.76 – 1.27]	0.93 [0.72 – 1.32]	–	–	–	–	–	–
MAD of AFR COx-a_L	0.18 [0.13 – 0.21]	0.19 [0.14 – 0.25]	0.19 [0.13 – 0.23]	–	–	–	–	–	–
MAD of AFR COx-a_R	0.18 [0.13 – 0.25]	0.19 [0.14 – 0.25]	0.18 [0.14 – 0.23]	–	–	–	–	–	–
**SP Population**									
MAD of AFR rSO_2_L	0.76 [0.64 – 1.07]	1.08 [0.74 – 1.56]	1.3 [0.84 – 1.83]	1.8 [1.25 – 2.15]	1.75 [1.28 – 2.34]	1.47 [1.33 – 2.3]	–	–	–
MAD of AFR rSO_2_R	0.72 [0.62 – 1.16]	0.94 [0.74 – 1.52]	1.3 [0.91 – 1.58]	1.58 [1.1 – 2.15]	1.49 [1.15 – 2.79]	1.19 [0.97 – 2.47]	–	–	–
MAD of AFR COx-a_L	0.29 [0.24 – 0.31]	0.3 [0.24 – 0.37]	0.31 [0.26 – 0.39]	0.28 [0.24 – 0.35]	0.26 [0.22 – 0.33]	0.21 [0.2 – 0.23]	–	–	–
MAD of AFR COx-a_R	0.3 [0.24 – 0.34]	0.29 [0.25 – 0.36]	0.34 [0.28 – 0.38]	0.29 [0.25 – 0.34]	0.26 [0.21 – 0.31]	0.27 [0.24 – 0.28]	–	–	–
**TBI Population**									
MAD of AFR rSO_2_L	0.57 [0.44 – 0.71]	0.68 [0.54 – 0.86]	0.75 [0.59 – 1.03]	1.03 [0.78 – 1.33]	1.29 [1.02 – 1.8]	1.71 [1.22 – 2.23]	2.12 [1.48 – 3.27]	2.64 [1.82 – 3.81]	2.97 [2.17 – 4.54]
MAD of AFR rSO_2_R	0.52 [0.42 – 0.67]	0.64 [0.51 – 0.85]	0.72 [0.58 – 0.97]	0.97 [0.71 – 1.17]	1.25 [1.03 – 1.58]	1.68 [1.3 – 2.08]	2.24 [1.63 – 3.19]	2.95 [2.19 – 4.14]	3.68 [2.48 – 4.46]
MAD of AFR COx_L	0.21 [0.19 – 0.23]	0.23 [0.21 – 0.25]	0.24 [0.21 – 0.27]	0.24 [0.22 – 0.26]	0.24 [0.22 – 0.27]	0.23 [0.21 – 0.25]	0.22 [0.2 – 0.24]	0.22 [0.19 – 0.24]	0.21 [0.19 – 0.24]
MAD of AFR COx_R	0.21 [0.19 – 0.23]	0.23 [0.21 – 0.25]	0.24 [0.22 – 0.26]	0.24 [0.22 – 0.26]	0.23 [0.22 – 0.26]	0.22 [0.21 – 0.25]	0.21 [0.19 – 0.24]	0.22 [0.19 – 0.24]	0.21 [0.19 – 0.23]
MAD of AFR COx-a_L	0.2 [0.19 – 0.23]	0.22 [0.21 – 0.25]	0.23 [0.21 – 0.25]	0.23 [0.21 – 0.25]	0.23 [0.21 – 0.25]	0.23 [0.2 – 0.25]	0.21 [0.18 – 0.23]	0.2 [0.18 – 0.22]	0.2 [0.18 – 0.22]
MAD of AFR COx-a_R	0.21 [0.19 – 0.22]	0.22 [0.2 – 0.25]	0.23 [0.21 – 0.26]	0.23 [0.21 – 0.26]	0.23 [0.21 – 0.25]	0.22 [0.19 – 0.24]	0.2 [0.18 – 0.23]	0.2 [0.18 – 0.22]	0.2 [0.17 – 0.22]

AFR, absolute forecast residual; COx, cerebral oximetry index with cerebral perfusion pressure; COx-a, cerebral oximetry index with arterial blood pressure; HC, healthy control volunteer group; IQR, interquartile range; MAD, median absolute deviation; rSO_2, regional cerebral oxygen saturation; SP, elective spinal surgery patient group; TBI, traumatic brain injury patient group; W&I, window and interval.

File S5j: Windowed-Interval – Absolute Forecast Residual of rSO_2 and COx/COx-a in All Populations using 1-Minute Temporal Resolution

Physiologic Variable	Median [IQR]								
	5-Minute W&I	10-Minute W&I	15-Minute W&I	30-Minute W&I	1-Hour W&I	2-Hour W&I	6-Hour W&I	12-Hour W&I	1-Day W&I
**HC Population**									
ARF of rSO_2_L	0.95 [0.49 – 1.68]	1.05 [0.53 – 1.71]	1.21 [0.61 – 1.84]	–	–	–	–	–	–
ARF of rSO_2_R	1.1 [0.53 – 1.91]	1.19 [0.57 – 1.91]	1.21 [0.77 – 2.02]	–	–	–	–	–	–
ARF of COx-a_L	0.27 [0.13 – 0.48]	0.25 [0.12 – 0.41]	0.25 [0.13 – 0.38]	–	–	–	–	–	–
ARF of COx-a_R	0.23 [0.12 – 0.43]	0.24 [0.13 – 0.41]	0.23 [0.11 – 0.36]	–	–	–	–	–	–
MAD of ARF rSO2_L	0.83 [0.62 – 1.08]	0.85 [0.59 – 1.14]	0.78 [0.55 – 1.14]	–	–	–	–	–	–
MAD of ARF rSO_2_R	0.93 [0.67 – 1.29]	0.92 [0.65 – 1.32]	0.77 [0.55 – 1.24]	–	–	–	–	–	–
MAD of ARF COx-a_L	0.22 [0.18 – 0.29]	0.19 [0.14 – 0.26]	0.17 [0.13 – 0.22]	–	–	–	–	–	–
MAD of ARF COx-a_R	0.21 [0.17 – 0.27]	0.19 [0.13 – 0.26]	0.15 [0.11 – 0.21]	–	–	–	–	–	–
**SP Population**									
ARF of rSO_2_L	0.84 [0.33 – 1.73]	1.18 [0.44 – 2.52]	1.24 [0.58 – 3.11]	1.8 [0.82 – 4.11]	1.97 [0.81 – 4.16]	1.82 [0.96 – 2.94]	–	–	–
ARF of rSO_2_R	0.81 [0.32 – 1.73]	0.88 [0.4 – 2.35]	1.17 [0.47 – 2.67]	1.45 [0.67 – 4.34]	2.08 [0.92 – 3.68]	1.34 [0.62 – 2.46]	–	–	–
ARF of COx-a_L	0.33 [0.14 – 0.65]	0.38 [0.17 – 0.68]	0.36 [0.17 – 0.62]	0.31 [0.15 – 0.55]	0.28 [0.14 – 0.47]	0.28 [0.12 – 0.47]	–	–	–
ARF of COx-a_R	0.35 [0.15 – 0.67]	0.37 [0.17 – 0.65]	0.36 [0.16 – 0.64]	0.33 [0.15 – 0.55]	0.29 [0.14 – 0.49]	0.3 [0.13 – 0.54]	–	–	–
MAD of ARF rSO_2_L	0.87 [0.7 – 1.13]	1.34 [0.88 – 1.69]	1.4 [0.96 – 2.22]	1.89 [1.33 – 2.54]	1.63 [1.14 – 3.35]	1.41 [1.32 – 2.37]	–	–	–
MAD of ARF rSO_2_R	0.81 [0.67 – 1.02]	0.94 [0.79 – 1.59]	1.27 [1.02 – 1.83]	1.67 [1.28 – 2.21]	1.99 [1.06 – 2.92]	1.11 [0.96 – 2.55]	–	–	–
MAD of ARF COx-a_L	0.33 [0.31 – 0.42]	0.35 [0.28 – 0.41]	0.32 [0.29 – 0.37]	0.29 [0.19 – 0.31]	0.25 [0.21 – 0.28]	0.26 [0.21 – 0.26]	–	–	–
MAD of ARF COx-a_R	0.34 [0.28 – 0.42]	0.34 [0.28 – 0.41]	0.32 [0.24 – 0.41]	0.28 [0.23 – 0.31]	0.24 [0.2 – 0.29]	0.26 [0.23 – 0.28]	–	–	–
**TBI Population**									
ARF of rSO_2_L	0.48 [0.18 – 1.03]	0.77 [0.3 – 1.75]	0.82 [0.32 – 1.88]	0.98 [0.4 – 2.19]	1.33 [0.56 – 2.68]	1.72 [0.75 – 3.4]	2.29 [1.07 – 4.53]	2.87 [1.26 – 5.32]	3.37 [1.55 – 5.81]
ARF of rSO_2_R	0.46 [0.17 – 0.99]	0.72 [0.28 – 1.56]	0.76 [0.29 – 1.63]	0.89 [0.35 – 2]	1.17 [0.48 – 2.59]	1.66 [0.65 – 3.48]	2.48 [1.04 – 4.59]	3.21 [1.37 – 5.37]	3.99 [1.75 – 6.99]
ARF of COx_L	0.26 [0.12 – 0.5]	0.29 [0.13 – 0.54]	0.27 [0.12 – 0.5]	0.25 [0.12 – 0.46]	0.24 [0.11 – 0.42]	0.22 [0.11 – 0.39]	0.22 [0.11 – 0.39]	0.23 [0.11 – 0.39]	0.23 [0.1 – 0.4]
ARF of COx_R	0.26 [0.12 – 0.5]	0.28 [0.12 – 0.54]	0.27 [0.12 – 0.5]	0.25 [0.11 – 0.45]	0.24 [0.11 – 0.42]	0.23 [0.11 – 0.4]	0.22 [0.1 – 0.38]	0.22 [0.1 – 0.39]	0.23 [0.11 – 0.4]
ARF of COx-a_L	0.26 [0.12 – 0.5]	0.29 [0.13 – 0.55]	0.27 [0.12 – 0.49]	0.24 [0.11 – 0.43]	0.22 [0.1 – 0.4]	0.22 [0.1 – 0.39]	0.21 [0.1 – 0.37]	0.21 [0.1 – 0.37]	0.21 [0.1 – 0.37]
ARF of COx-a_R	0.26 [0.11 – 0.49]	0.29 [0.13 – 0.54]	0.27 [0.12 – 0.5]	0.24 [0.11 – 0.44]	0.23 [0.1 – 0.41]	0.21 [0.1 – 0.38]	0.21 [0.1 – 0.37]	0.21 [0.1 – 0.36]	0.22 [0.1 – 0.38]
MAD of ARF rSO_2_L	0.53 [0.39 – 0.68]	0.85 [0.57 – 1.15]	0.9 [0.6 – 1.34]	1.08 [0.78 – 1.49]	1.35 [1.06 – 1.94]	1.75 [1.28 – 2.46]	2.27 [1.53 – 3.21]	2.54 [1.85 – 3.74]	2.97 [2.2 – 4.69]
MAD of ARF rSO_2_R	0.51 [0.4 – 0.65]	0.77 [0.55 – 1.06]	0.82 [0.62 – 1.1]	0.96 [0.76 – 1.37]	1.27 [0.99 – 1.77]	1.73 [1.31 – 2.11]	2.35 [1.73 – 3.54]	2.95 [2.15 – 3.73]	3.42 [2.41 – 4.49]
MAD of ARF COx_L	0.25 [0.22 – 0.29]	0.28 [0.24 – 0.34]	0.26 [0.23 – 0.3]	0.23 [0.21 – 0.26]	0.22 [0.2 – 0.24]	0.2 [0.19 – 0.23]	0.2 [0.18 – 0.22]	0.2 [0.18 – 0.22]	0.2 [0.18 – 0.23]
MAD of ARF COx_R	0.25 [0.22 – 0.29]	0.27 [0.24 – 0.33]	0.25 [0.23 – 0.29]	0.23 [0.21 – 0.25]	0.22 [0.19 – 0.23]	0.2 [0.19 – 0.22]	0.2 [0.18 – 0.22]	0.2 [0.18 – 0.22]	0.21 [0.18 – 0.23]
MAD of ARF COx-a_L	0.25 [0.22 – 0.28]	0.28 [0.23 – 0.34]	0.25 [0.22 – 0.28]	0.22 [0.2 – 0.25]	0.21 [0.19 – 0.23]	0.2 [0.18 – 0.22]	0.19 [0.17 – 0.21]	0.19 [0.17 – 0.21]	0.19 [0.17 – 0.21]
MAD of ARF COx-a_R	0.25 [0.22 – 0.28]	0.27 [0.23 – 0.33]	0.25 [0.23 – 0.29]	0.22 [0.2 – 0.25]	0.21 [0.19 – 0.23]	0.2 [0.18 – 0.22]	0.18 [0.17 – 0.21]	0.19 [0.17 – 0.21]	0.19 [0.17 – 0.21]

ARF, absolute forecast residual; COx, cerebral oximetry index with cerebral perfusion pressure; COx-a, cerebral oximetry index with arterial blood pressure; HC, healthy control volunteer group; IQR, interquartile range; MAD, median absolute deviation; rSO_2, regional cerebral oxygen saturation; SP, elective spinal surgery patient group; TBI, traumatic brain injury patient group; W&I, window and interval.

File S5k: Windowed-Interval – Absolute Forecast Residual of rSO_2 and COx/COx-a in All Populations using 5-Minute Temporal Resolution

Physiologic Variable	Median [IQR]							
	10-Minute W&I	15-Minute W&I	30-Minute W&I	1-Hour W&I	2-Hour W&I	6-Hour W&I	12-Hour W&I	1-Day W&I
**HC Population**								
ARF of rSO_2_L	0.87 [0.57 – 1.23]	1.11 [0.78 – 1.4]	–	–	–	–	–	–
ARF of rSO_2_R	0.93 [0.6 – 1.32]	1.16 [0.89 – 1.53]	–	–	–	–	–	–
ARF of COx-a_L	0.25 [0.17 – 0.33]	0.25 [0.2 – 0.37]	–	–	–	–	–	–
ARF of COx-a_R	0.23 [0.16 – 0.29]	0.22 [0.14 – 0.3]	–	–	–	–	–	–
MAD of ARF rSO_2_L	0.4 [0.19 – 0.66]	0.33 [0.15 – 0.72]	–	–	–	–	–	–
MAD of ARF rSO_2_R	0.51 [0.23 – 0.78]	0.44 [0.24 – 0.89]	–	–	–	–	–	–
MAD of ARF COx-a_L	0.1 [0.04 – 0.17]	0.09 [0.04 – 0.19]	–	–	–	–	–	–
MAD of ARF COx-a_R	0.09 [0.04 – 0.17]	0.1 [0.05 – 0.17]	–	–	–	–	–	–
**SP Population**								
ARF of rSO_2_L	1.06 [0.51 – 2.34]	1.24 [0.57 – 3]	2.06 [0.9 – 4.03]	2.56 [1.33 – 4.09]	1.25 [0.64 – 2.37]	–	–	–
ARF of rSO_2_R	1.02 [0.38 – 2.25]	1.45 [0.51 – 3.58]	1.93 [0.8 – 4.11]	1.87 [0.93 – 3.79]	1.28 [0.62 – 2.41]	–	–	–
ARF of COx-a_L	0.29 [0.15 – 0.48]	0.31 [0.15 – 0.61]	0.29 [0.14 – 0.51]	0.26 [0.14 – 0.41]	0.21 [0.09 – 0.4]	–	–	–
ARF of COx-a_R	0.27 [0.14 – 0.47]	0.31 [0.17 – 0.57]	0.3 [0.17 – 0.49]	0.31 [0.16 – 0.54]	0.2 [0.1 – 0.36]	–	–	–
MAD of ARF rSO_2_L	0.98 [0.64 – 1.71]	1.39 [0.86 – 1.88]	2.08 [1.26 – 3.01]	1.98 [1.15 – 2.91]	1.31 [1.07 – 2.12]	–	–	–
MAD of ARF rSO_2_R	1.04 [0.7 – 1.77]	1.54 [1.02 – 2.29]	1.71 [1.18 – 3.09]	1.89 [0.97 – 3.39]	1.13 [1.09 – 2.51]	–	–	–
MAD of ARF COx-a_L	0.22 [0.18 – 0.28]	0.31 [0.23 – 0.36]	0.26 [0.2 – 0.31]	0.22 [0.16 – 0.34]	0.2 [0.17 – 0.24]	–	–	–
MAD of ARF COx-a_R	0.22 [0.19 – 0.26]	0.28 [0.22 – 0.37]	0.24 [0.2 – 0.29]	0.23 [0.18 – 0.31]	0.19 [0.18 – 0.23]	–	–	–
**TBI Population**								
ARF of rSO_2_L	0.58 [0.24 – 1.35]	0.77 [0.31 – 1.75]	1.43 [0.55 – 3]	1.73 [0.76 – 3.9]	1.83 [0.79 – 3.77]	2.65 [1.09 – 4.69]	2.98 [1.39 – 5.72]	3.66 [1.62 – 5.87]
ARF of rSO_2_R	0.54 [0.2 – 1.15]	0.64 [0.24 – 1.62]	1.25 [0.5 – 2.64]	1.49 [0.58 – 3.12]	1.76 [0.76 – 3.61]	2.52 [1.04 – 4.77]	3.3 [1.53 – 6.01]	3.94 [1.9 – 7.11]
ARF of COx_L	0.21 [0.1 – 0.37]	0.25 [0.12 – 0.45]	0.28 [0.12 – 0.5]	0.23 [0.11 – 0.41]	0.2 [0.1 – 0.36]	0.19 [0.09 – 0.34]	0.19 [0.09 – 0.33]	0.19 [0.09 – 0.34]
ARF of COx_R	0.2 [0.09 – 0.37]	0.25 [0.11 – 0.45]	0.26 [0.12 – 0.46]	0.23 [0.11 – 0.41]	0.21 [0.1 – 0.36]	0.19 [0.09 – 0.34]	0.19 [0.09 – 0.32]	0.19 [0.09 – 0.33]
ARF of COx-a_L	0.2 [0.09 – 0.36]	0.24 [0.11 – 0.44]	0.27 [0.12 – 0.49]	0.22 [0.11 – 0.4]	0.2 [0.09 – 0.35]	0.18 [0.08 – 0.31]	0.18 [0.08 – 0.31]	0.18 [0.08 – 0.3]
ARF of COx-a_R	0.2 [0.09 – 0.35]	0.24 [0.11 – 0.43]	0.27 [0.13 – 0.51]	0.23 [0.11 – 0.41]	0.2 [0.09 – 0.35]	0.18 [0.09 – 0.31]	0.17 [0.08 – 0.31]	0.18 [0.09 – 0.31]
MAD of ARF rSO_2_L	0.64 [0.47 – 0.89]	0.86 [0.56 – 1.14]	1.44 [1.03 – 1.97]	1.87 [1.33 – 2.6]	1.87 [1.38 – 2.63]	2.45 [1.59 – 3.44]	2.67 [2 – 3.79]	2.99 [2.12 – 4.42]
MAD of ARF rSO_2_R	0.56 [0.43 – 0.86]	0.74 [0.54 – 1]	1.3 [0.84 – 1.8]	1.6 [1.18 – 2.5]	1.79 [1.47 – 2.55]	2.4 [1.77 – 3.4]	2.85 [2.04 – 3.97]	3.48 [2.39 – 4.54]
MAD of ARF COx_L	0.19 [0.17 – 0.21]	0.23 [0.21 – 0.26]	0.26 [0.21 – 0.3]	0.2 [0.18 – 0.26]	0.19 [0.17 – 0.21]	0.17 [0.15 – 0.19]	0.17 [0.15 – 0.19]	0.17 [0.15 – 0.19]
MAD of ARF COx_R	0.18 [0.17 – 0.2]	0.23 [0.21 – 0.26]	0.24 [0.2 – 0.29]	0.21 [0.19 – 0.26]	0.18 [0.17 – 0.2]	0.17 [0.16 – 0.19]	0.17 [0.15 – 0.18]	0.17 [0.16 – 0.18]
MAD of ARF COx-a_L	0.18 [0.17 – 0.2]	0.22 [0.21 – 0.25]	0.25 [0.21 – 0.29]	0.2 [0.18 – 0.25]	0.17 [0.16 – 0.2]	0.16 [0.15 – 0.18]	0.16 [0.14 – 0.18]	0.16 [0.14 – 0.18]
MAD of ARF COx-a_R	0.18 [0.17 – 0.2]	0.22 [0.21 – 0.24]	0.25 [0.22 – 0.3]	0.21 [0.18 – 0.25]	0.18 [0.16 – 0.2]	0.16 [0.15 – 0.18]	0.16 [0.14 – 0.17]	0.16 [0.14 – 0.18]

ARF, absolute forecast residual; COx, cerebral oximetry index with cerebral perfusion pressure; COx-a, cerebral oximetry index with arterial blood pressure; HC, healthy control volunteer group; IQR, interquartile range; MAD, median absolute deviation; rSO_2, regional cerebral oxygen saturation; SP, elective spinal surgery patient group; TBI, traumatic brain injury patient group; W&I, window and interval.
