# Supplementary material for: Time-Series Autoregressive Models for Point and Interval Forecasting of Raw and Derived Commercial Near-Infrared Spectroscopy Measures: An Exploratory Cranial Trauma and Healthy Control Analysis
Source: Bioengineering (Basel). 2025 Jun 21;12(7):682. doi: 10.3390/bioengineering12070682 (PMC12292983; doi:10.3390/bioengineering12070682)
Supplement: Supplementary file 1 [file bioengineering-12-00682-s001.zip › File S6.pdf]

File S6 – Root Mean Squared Error Analysis

File S6 – Table of Contents

File S6a: Anchored-Point – Root Mean Squared Error of rSO_2 and COx/COx-a in All Populations using 10-Second, 1-Minute, and 5-Minute Temporal Resolutions 2

File S6b: Anchored-Interval – Root Mean Squared Error of rSO_2 and COx/COx-a in All Populations using 10-Second Temporal Resolution 3

File S6c: Anchored-Interval – Root Mean Squared Error of rSO_2 and COx/COx-a in All Populations using 1-Minute Temporal Resolution 4

File S6d: Anchored-Interval – Root Mean Squared Error of rSO_2 and COx/COx-a in All Populations using 5-Minute Temporal Resolution 5

File S6e: Windowed-Point – Root Mean Squared Error of rSO_2 and COx/COx-a in All Populations using 10-Second Temporal Resolution 6

File S6f: Windowed-Point – Root Mean Squared Error of rSO_2 and COx/COx-a in All Populations using 1-Minute Temporal Resolution 7

File S6g: Windowed-Point – Root Mean Squared Error of rSO_2 and COx/COx-a in All Populations using 5-Minute Temporal Resolution 8

File S6h: Windowed-Interval – Root Mean Squared Error of rSO_2 and COx/COx-a in All Populations using 10-Second Temporal Resolution 9

File S6i: Windowed-Interval – Root Mean Squared Error of rSO_2 and COx/COx-a in All Populations using 1-Minute Temporal Resolution 10

File S6j: Windowed-Interval – Root Mean Squared Error of rSO_2 and COx/COx-a in All Populations using 5-Minute Temporal Resolution 11

File S6a: Anchored-Point – Root Mean Squared Error of rSO_2 and COx/COx-a in All Populations using 10-Second, 1-Minute, and 5-Minute Temporal Resolutions

RMSE of Physiologic Variable	Median [IQR; MAD]		
	**HC**	**SP**	**TBI**
**10-Second Temporal Resolution**			
rSO_2_L	1.29 [0.97 – 1.77; 0.56]	4.05 [2.2 – 7.29; 3.51]	6.08 [3.55 – 9.35; 4.14]
rSO_2_R	1.35 [1.01 – 1.72; 0.54]	3.83 [2.94 – 7.49; 2.69]	0.35 [0.31 – 0.39; 0.06]
COx_L	–	–	0.33 [0.3 – 0.35; 0.04]
COx_R	–	–	4.96 [3.15 – 8.33; 3.26]
COx-a_L	0.25 [0.17 – 0.36; 0.14]	0.4 [0.36 – 0.59; 0.15]	0.35 [0.32 – 0.4; 0.06]
COx-a_R	0.26 [0.17 – 0.35; 0.13]	0.49 [0.38 – 0.6; 0.17]	0.33 [0.3 – 0.37; 0.05]
**1-Minute Temporal Resolution**			
rSO_2_L	1.05 [0.75 – 1.53; 0.53]	3.76 [2.55 – 7.4; 3.09]	5.64 [3.13 – 9.19; 4.23]
rSO_2_R	1.09 [0.8 – 1.56; 0.52]	3.68 [2.97 – 6.77; 2.32]	0.33 [0.29 – 0.38; 0.06]
COx_L	–	–	0.32 [0.28 – 0.34; 0.04]
COx_R	–	–	5.22 [3.44 – 8.57; 3.21]
COx-a_L	0.24 [0.17 – 0.4; 0.13]	0.41 [0.34 – 0.54; 0.13]	0.34 [0.31 – 0.39; 0.06]
COx-a_R	0.23 [0.17 – 0.33; 0.13]	0.45 [0.37 – 0.54; 0.12]	0.32 [0.29 – 0.35; 0.05]
**5-Minute Temporal Resolution**			
rSO_2_L	1.36 [0.77 – 2.14; 0.94]	5.15 [2.25 – 7.42; 4.28]	5.04 [2.75 – 8.97; 3.73]
rSO_2_R	1.51 [0.95 – 2.67; 1.15]	3.18 [2.42 – 5.73; 2.47]	0.28 [0.26 – 0.32; 0.04]
COx_L	–	–	0.27 [0.24 – 0.29; 0.04]
COx_R	–	–	5.06 [3.24 – 8.32; 3.24]
COx-a_L	0.3 [0.19 – 0.47; 0.2]	0.35 [0.24 – 0.48; 0.18]	0.28 [0.25 – 0.33; 0.05]
COx-a_R	0.32 [0.16 – 0.46; 0.22]	0.42 [0.33 – 0.47; 0.12]	0.26 [0.24 – 0.3; 0.04]

COx, cerebral oximetry index with cerebral perfusion pressure; COx-a, cerebral oximetry index with arterial blood pressure; HC, healthy control volunteer group; IQR, interquartile range; MAD, median absolute deviation; RMSE, root mean squared error; rSO_2, regional cerebral oxygen saturation; SP, elective spinal surgery patient group; TBI, traumatic brain injury patient group.

File S6b: Anchored-Interval – Root Mean Squared Error of rSO_2 and COx/COx-a in All Populations using 10-Second Temporal Resolution

RMSE of Physiologic Variable	Median [IQR; MAD]								
	5-Minute Interval	10-Minute Interval	15-Minute Interval	30-Minute Interval	1-Hour Interval	2-Hour Interval	6-Hour Interval	12-Hour Interval	1-Day Interval
**HC Population**									
rSO_2_L	1.21 [0.91 – 1.63; 0.49]	1.66 [1.66 – 1.66; 0]	–	–	–	–	–	–	–
rSO_2_R	1.27 [0.95 – 1.63; 0.52]	1.93 [1.93 – 1.93; 0]	–	–	–	–	–	–	–
COx-a_L	0.23 [0.17 – 0.34; 0.13]	0.32 [0.32 – 0.32; 0]	–	–	–	–	–	–	–
COx-a_R	0.23 [0.14 – 0.33; 0.14]	0.24 [0.24 – 0.24; 0]	–	–	–	–	–	–	–
**SP Population**									
rSO_2_L	1.83 [1.41 – 3.21; 1.32]	2.44 [1.87 – 4.04; 1.86]	3.14 [1.91 – 4.86; 1.96]	4.12 [2.37 – 6.73; 3.46]	4.2 [3.49 – 14.35; 2.08]	–	–	–	–
rSO_2_R	2.06 [1.31 – 2.55; 1.04]	2.5 [1.83 – 3.96; 1.55]	3.76 [2.01 – 4.87; 2.16]	3.44 [2.27 – 7.12; 2.97]	5.47 [3.92 – 15.66; 4.58]	–	–	–	–
COx-a_L	0.35 [0.28 – 0.46; 0.14]	0.36 [0.32 – 0.48; 0.1]	0.38 [0.34 – 0.51; 0.14]	0.45 [0.35 – 0.58; 0.19]	0.36 [0.34 – 0.38; 0.06]	–	–	–	–
COx-a_R	0.4 [0.33 – 0.48; 0.11]	0.44 [0.34 – 0.53; 0.15]	0.46 [0.36 – 0.54; 0.14]	0.45 [0.36 – 0.56; 0.16]	0.36 [0.34 – 0.4; 0.07]	–	–	–	–
**TBI Population**									
rSO_2_L	1.93 [1.27 – 3.17; 1.34]	2.25 [1.45 – 3.78; 1.56]	2.53 [1.47 – 4.2; 1.75]	2.83 [1.77 – 4.43; 1.86]	3.56 [2.08 – 5.3; 2.35]	3.81 [2.3 – 6.21; 2.52]	4.58 [2.87 – 7.44; 2.83]	4.8 [3.22 – 8.04; 2.72]	5.67 [4.24 – 10.38; 2.98]
rSO_2_R	0.28 [0.26 – 0.32; 0.04]	0.31 [0.28 – 0.35; 0.05]	0.32 [0.29 – 0.36; 0.05]	0.33 [0.3 – 0.37; 0.05]	0.33 [0.31 – 0.38; 0.05]	0.34 [0.31 – 0.38; 0.05]	0.35 [0.32 – 0.39; 0.05]	0.35 [0.32 – 0.39; 0.05]	0.37 [0.32 – 0.4; 0.06]
COx_L	0.28 [0.25 – 0.31; 0.04]	0.3 [0.27 – 0.33; 0.05]	0.31 [0.28 – 0.34; 0.04]	0.32 [0.29 – 0.35; 0.05]	0.33 [0.3 – 0.36; 0.04]	0.33 [0.3 – 0.36; 0.04]	0.33 [0.3 – 0.36; 0.05]	0.34 [0.31 – 0.36; 0.04]	0.33 [0.3 – 0.36; 0.04]
COx_R	1.97 [1.15 – 3.17; 1.53]	2.46 [1.31 – 4.16; 1.77]	2.66 [1.49 – 4.49; 1.96]	3.19 [1.86 – 4.93; 2.22]	3.63 [1.97 – 5.93; 2.61]	4.12 [2.29 – 6.5; 3.09]	5.54 [2.97 – 7.32; 3.43]	5.8 [3.83 – 9.77; 3.58]	6.36 [4.58 – 10.73; 3.92]
COx-a_L	0.28 [0.25 – 0.32; 0.04]	0.31 [0.28 – 0.35; 0.05]	0.32 [0.29 – 0.36; 0.05]	0.33 [0.29 – 0.36; 0.05]	0.34 [0.29 – 0.36; 0.06]	0.34 [0.3 – 0.38; 0.06]	0.35 [0.31 – 0.38; 0.05]	0.35 [0.31 – 0.38; 0.06]	0.37 [0.32 – 0.4; 0.05]
COx-a_R	0.27 [0.24 – 0.3; 0.04]	0.3 [0.27 – 0.33; 0.04]	0.31 [0.28 – 0.33; 0.04]	0.32 [0.29 – 0.34; 0.04]	0.33 [0.29 – 0.35; 0.04]	0.33 [0.3 – 0.35; 0.04]	0.33 [0.3 – 0.36; 0.04]	0.33 [0.3 – 0.35; 0.04]	0.34 [0.31 – 0.36; 0.04]

COx, cerebral oximetry index with cerebral perfusion pressure; COx-a, cerebral oximetry index with arterial blood pressure; HC, healthy control volunteer group; IQR, interquartile range; MAD, median absolute deviation; RMSE, root mean squared error; rSO_2, regional cerebral oxygen saturation; SP, elective spinal surgery patient group; TBI, traumatic brain injury patient group.

File S6c: Anchored-Interval – Root Mean Squared Error of rSO_2 and COx/COx-a in All Populations using 1-Minute Temporal Resolution

RMSE of Physiologic Variable	Median [IQR; MAD]								
	5-Minute Interval	10-Minute Interval	15-Minute Interval	30-Minute Interval	1-Hour Interval	2-Hour Interval	6-Hour Interval	12-Hour Interval	1-Day Interval
**HC Population**									
rSO_2_L	0.91 [0.72 – 1.45; 0.45]	–	–	–	–	–	–	–	–
rSO_2_R	1.09 [0.79 – 1.43; 0.48]	–	–	–	–	–	–	–	–
COx-a_L	0.23 [0.15 – 0.35; 0.14]	–	–	–	–	–	–	–	–
COx-a_R	0.21 [0.14 – 0.31; 0.12]	–	–	–	–	–	–	–	–
**SP Population**									
rSO_2_L	2.27 [1.32 – 3.24; 1.58]	3.2 [1.9 – 5.1; 1.98]	3 [1.86 – 5.73; 2.4]	4.17 [2.56 – 6.32; 3.18]	3.81 [2.95 – 14.15; 2.56]	–	–	–	–
rSO_2_R	2.57 [1.46 – 2.86; 0.98]	3.18 [1.87 – 4.31; 1.69]	3.42 [2.34 – 4.4; 1.64]	3.67 [2.23 – 6.32; 2.64]	4.97 [3.37 – 15.29; 4.76]	–	–	–	–
COx-a_L	0.37 [0.29 – 0.46; 0.13]	0.39 [0.31 – 0.49; 0.13]	0.41 [0.33 – 0.51; 0.14]	0.45 [0.33 – 0.54; 0.16]	0.34 [0.33 – 0.44; 0.01]	–	–	–	–
COx-a_R	0.37 [0.3 – 0.44; 0.11]	0.41 [0.36 – 0.45; 0.06]	0.43 [0.36 – 0.49; 0.1]	0.44 [0.36 – 0.53; 0.13]	0.35 [0.34 – 0.4; 0.03]	–	–	–	–
**TBI Population**									
rSO_2_L	1.81 [1.16 – 3.36; 1.38]	2.3 [1.31 – 3.75; 1.68]	2.52 [1.42 – 4.27; 1.84]	2.91 [1.8 – 4.6; 2.05]	3.65 [2.02 – 5.96; 2.58]	3.73 [2.37 – 6.33; 2.56]	4.32 [2.89 – 7.37; 2.53]	4.86 [3.41 – 7.77; 2.85]	5.66 [4.18 – 11.39; 3.51]
rSO_2_R	0.28 [0.26 – 0.31; 0.04]	0.31 [0.28 – 0.34; 0.04]	0.31 [0.28 – 0.35; 0.05]	0.32 [0.29 – 0.36; 0.05]	0.32 [0.3 – 0.37; 0.05]	0.32 [0.3 – 0.37; 0.05]	0.33 [0.31 – 0.37; 0.05]	0.34 [0.31 – 0.37; 0.05]	0.35 [0.31 – 0.38; 0.05]
COx_L	0.28 [0.25 – 0.3; 0.04]	0.3 [0.27 – 0.32; 0.04]	0.3 [0.27 – 0.33; 0.04]	0.31 [0.28 – 0.34; 0.05]	0.32 [0.28 – 0.35; 0.05]	0.32 [0.29 – 0.35; 0.05]	0.32 [0.29 – 0.35; 0.05]	0.32 [0.3 – 0.35; 0.04]	0.32 [0.29 – 0.35; 0.04]
COx_R	1.83 [1.02 – 3.55; 1.52]	2.18 [1.2 – 4.15; 1.75]	2.5 [1.36 – 4.67; 2.04]	2.86 [1.72 – 4.95; 2.37]	3.33 [2.06 – 5.89; 2.7]	3.86 [2.27 – 6.44; 3.25]	5.41 [3.03 – 7.72; 3.6]	5.64 [3.68 – 9.61; 3.49]	6.36 [4.56 – 11.24; 4.03]
COx-a_L	0.28 [0.25 – 0.31; 0.04]	0.31 [0.27 – 0.34; 0.05]	0.3 [0.28 – 0.34; 0.04]	0.32 [0.28 – 0.35; 0.05]	0.32 [0.28 – 0.35; 0.06]	0.33 [0.29 – 0.36; 0.05]	0.33 [0.3 – 0.36; 0.05]	0.34 [0.29 – 0.37; 0.05]	0.35 [0.32 – 0.38; 0.05]
COx-a_R	0.27 [0.25 – 0.3; 0.04]	0.29 [0.27 – 0.31; 0.03]	0.3 [0.27 – 0.32; 0.04]	0.31 [0.27 – 0.33; 0.04]	0.31 [0.28 – 0.34; 0.04]	0.32 [0.28 – 0.34; 0.04]	0.32 [0.29 – 0.34; 0.04]	0.32 [0.28 – 0.34; 0.04]	0.33 [0.3 – 0.35; 0.04]

COx, cerebral oximetry index with cerebral perfusion pressure; COx-a, cerebral oximetry index with arterial blood pressure; HC, healthy control volunteer group; IQR, interquartile range; MAD, median absolute deviation; RMSE, root mean squared error; rSO_2, regional cerebral oxygen saturation; SP, elective spinal surgery patient group; TBI, traumatic brain injury patient group.

File S6d: Anchored-Interval – Root Mean Squared Error of rSO_2 and COx/COx-a in All Populations using 5-Minute Temporal Resolution

RMSE of Physiologic Variable	Median [IQR; MAD]								
	5-Minute Interval	10-Minute Interval	15-Minute Interval	30-Minute Interval	1-Hour Interval	2-Hour Interval	6-Hour Interval	12-Hour Interval	1-Day Interval
**HC Population**									
rSO_2_L	1.34 [0.82 – 2.08; 0.91]	–	–	–	–	–	–	–	–
rSO_2_R	1.42 [0.92 – 2.19; 0.93]	–	–	–	–	–	–	–	–
COx-a_L	0.28 [0.18 – 0.48; 0.18]	–	–	–	–	–	–	–	–
COx-a_R	0.33 [0.16 – 0.5; 0.25]	–	–	–	–	–	–	–	–
**SP Population**									
rSO_2_L	2.09 [1.37 – 3.57; 1.25]	2.91 [1.89 – 4.46; 1.76]	3.26 [2.04 – 5.51; 2.72]	2.97 [1.69 – 6.23; 2.23]	3.83 [2.69 – 49.32; 3.38]	–	–	–	–
rSO_2_R	2.27 [1.23 – 2.88; 1.38]	2.53 [1.3 – 3.91; 1.97]	2.87 [1.63 – 4.16; 1.9]	3.18 [1.81 – 5.58; 2.85]	2.34 [2.11 – 38.03; 0.68]	–	–	–	–
COx-a_L	0.34 [0.26 – 0.42; 0.13]	0.33 [0.23 – 0.43; 0.15]	0.36 [0.26 – 0.49; 0.14]	0.33 [0.24 – 0.45; 0.14]	0.29 [0.28 – 0.33; 0.03]	–	–	–	–
COx-a_R	0.36 [0.28 – 0.42; 0.1]	0.35 [0.29 – 0.41; 0.09]	0.38 [0.31 – 0.44; 0.1]	0.38 [0.3 – 0.47; 0.13]	0.29 [0.27 – 0.34; 0.06]	–	–	–	–
**TBI Population**									
rSO_2_L	1.81 [1.08 – 3.53; 1.32]	2.03 [1.26 – 4.29; 1.47]	2.19 [1.36 – 4.83; 1.47]	2.7 [1.74 – 5.27; 2.04]	3.07 [1.85 – 5.49; 2.2]	3.63 [2.26 – 6.64; 2.67]	4.08 [3.01 – 7.21; 2.92]	5.06 [3.34 – 8.61; 3.43]	6.21 [4.11 – 9.73; 3.58]
rSO_2_R	0.26 [0.24 – 0.29; 0.04]	0.27 [0.25 – 0.3; 0.04]	0.27 [0.25 – 0.31; 0.04]	0.27 [0.25 – 0.32; 0.04]	0.27 [0.25 – 0.32; 0.04]	0.27 [0.25 – 0.32; 0.04]	0.28 [0.26 – 0.32; 0.05]	0.28 [0.26 – 0.32; 0.05]	0.29 [0.26 – 0.32; 0.05]
COx_L	0.25 [0.23 – 0.27; 0.03]	0.26 [0.23 – 0.28; 0.04]	0.26 [0.23 – 0.28; 0.03]	0.26 [0.24 – 0.28; 0.04]	0.26 [0.23 – 0.28; 0.04]	0.26 [0.24 – 0.29; 0.04]	0.27 [0.25 – 0.3; 0.04]	0.27 [0.25 – 0.3; 0.03]	0.26 [0.25 – 0.31; 0.04]
COx_R	1.88 [0.89 – 3.44; 1.7]	2.3 [1.09 – 4.03; 1.93]	2.4 [1.22 – 4.66; 1.92]	2.66 [1.61 – 5.11; 2.12]	3.3 [1.76 – 5.3; 2.41]	3.79 [1.96 – 6.26; 2.96]	5.17 [2.97 – 7.56; 3.45]	5.51 [3.39 – 9.52; 4.04]	6.1 [3.89 – 9.41; 3.9]
COx-a_L	0.26 [0.23 – 0.28; 0.04]	0.27 [0.24 – 0.29; 0.04]	0.27 [0.24 – 0.3; 0.04]	0.28 [0.25 – 0.3; 0.04]	0.28 [0.25 – 0.31; 0.04]	0.28 [0.25 – 0.31; 0.04]	0.28 [0.26 – 0.31; 0.04]	0.28 [0.25 – 0.31; 0.04]	0.31 [0.28 – 0.33; 0.04]
COx-a_R	0.25 [0.22 – 0.27; 0.04]	0.25 [0.23 – 0.28; 0.04]	0.25 [0.23 – 0.28; 0.04]	0.26 [0.23 – 0.28; 0.04]	0.26 [0.23 – 0.28; 0.04]	0.26 [0.24 – 0.29; 0.04]	0.27 [0.24 – 0.29; 0.04]	0.26 [0.24 – 0.29; 0.03]	0.27 [0.25 – 0.29; 0.02]

COx, cerebral oximetry index with cerebral perfusion pressure; COx-a, cerebral oximetry index with arterial blood pressure; HC, healthy control volunteer group; IQR, interquartile range; MAD, median absolute deviation; RMSE, root mean squared error; rSO_2, regional cerebral oxygen saturation; SP, elective spinal surgery patient group; TBI, traumatic brain injury patient group.

File S6e: Windowed-Point – Root Mean Squared Error of rSO_2 and COx/COx-a in All Populations using 10-Second Temporal Resolution

RMSE of Physiologic Variable	Median [IQR; MAD]								
	5-Minute Window	10-Minute Window	15-Minute Window	30-Minute Window	1-Hour Window	2-Hour Window	6-Hour Window	12-Hour Window	1-Day Window
**HC Population**									
rSO_2_L	0.85 [0.63 – 1.05; 0.32]	0.78 [0.6 – 0.94; 0.27]	0.74 [0.57 – 0.93; 0.27]	0.7 [0.52 – 0.97; 0.31]	–	–	–	–	–
rSO_2_R	0.93 [0.76 – 1.21; 0.31]	0.85 [0.68 – 1.08; 0.28]	0.8 [0.68 – 1.06; 0.26]	0.78 [0.59 – 1.06; 0.36]	–	–	–	–	–
COx-a_L	0.07 [0.06 – 0.08; 0.01]	0.06 [0.05 – 0.07; 0.01]	0.06 [0.05 – 0.07; 0.01]	0.05 [0.04 – 0.06; 0.02]	–	–	–	–	–
COx-a_R	0.07 [0.06 – 0.08; 0.01]	0.06 [0.05 – 0.07; 0.01]	0.06 [0.05 – 0.07; 0.01]	0.05 [0.04 – 0.06; 0.02]	–	–	–	–	–
**SP Population**									
rSO_2_L	1.2 [0.81 – 2.3; 0.92]	1.43 [0.63 – 2.82; 1.3]	0.96 [0.59 – 2.47; 0.89]	1.13 [0.5 – 2.26; 1.07]	0.56 [0.45 – 1.2; 0.33]	0.6 [0.46 – 1.08; 0.33]	2781.91 [1393.77 – 4170.06; 4116.13]	–	–
rSO_2_R	1.65 [0.7 – 3.14; 1.5]	1.14 [0.54 – 3.85; 0.98]	0.81 [0.52 – 3.67; 0.63]	0.59 [0.48 – 1.49; 0.35]	0.49 [0.42 – 1.05; 0.19]	0.62 [0.46 – 0.82; 0.27]	8.91 [7.74 – 10.09; 3.48]	–	–
COx-a_L	0.1 [0.09 – 0.12; 0.03]	0.08 [0.08 – 0.1; 0.02]	0.08 [0.07 – 0.09; 0.01]	0.07 [0.07 – 0.08; 0.01]	0.07 [0.06 – 0.08; 0.01]	0.07 [0.06 – 0.08; 0.01]	0.07 [0.07 – 0.07; 0]	–	–
COx-a_R	0.1 [0.09 – 0.12; 0.02]	0.08 [0.08 – 0.1; 0.01]	0.08 [0.07 – 0.1; 0.02]	0.07 [0.07 – 0.09; 0.01]	0.07 [0.06 – 0.08; 0.01]	0.07 [0.06 – 0.08; 0.02]	0.08 [0.08 – 0.08; 0.01]	–	–
**TBI Population**									
rSO_2_L	1.71 [0.95 – 3.38; 1.41]	2.47 [0.97 – 14.86; 2.64]	63.46 [0.93 – 4.13E+06; 93.59]	3.65E+04 [0.89 – 3.54E+20; 5.42E+04]	1.21E+05 [0.7 – 9.40E+35; 1.80E+05]	5.12 [0.68 – 1.05E+33; 7.19]	0.66 [0.57 – 2497.55; 0.52]	0.75 [0.57 – 1.86; 0.54]	1.31 [1 – 1.53; 0.45]
rSO_2_R	0.1 [0.1 – 0.11; 0.01]	0.08 [0.08 – 0.09; 0.01]	0.08 [0.07 – 0.09; 0.01]	0.07 [0.07 – 0.08; 0.01]	0.07 [0.07 – 0.08; 0.01]	0.07 [0.06 – 0.07; 0.01]	0.07 [0.07 – 0.07; 0.01]	0.07 [0.06 – 0.07; 0.01]	0.06 [0.06 – 0.07; 0]
COx_L	0.1 [0.09 – 0.11; 0.01]	0.08 [0.08 – 0.09; 0.01]	0.08 [0.07 – 0.09; 0.01]	0.07 [0.07 – 0.08; 0.01]	0.07 [0.07 – 0.08; 0.01]	0.07 [0.06 – 0.07; 0.01]	0.07 [0.07 – 0.07; 0.01]	0.07 [0.06 – 0.07; 0.01]	0.07 [0.07 – 0.07; 0]
COx_R	1.58 [0.93 – 2.86; 1.29]	3.21 [1.28 – 14.97; 3.67]	28.46 [1.06 – 3.12E+16; 41.52]	1.32E+06 [1.31 – 3.12E+16; 1.96E+06]	1.24E+08 [0.76 – 4.65E+26; 1.83E+08]	1.33E+11 [0.69 – 2.67E+40; 1.97E+11]	0.57 [0.42 – 0.76; 0.23]	0.52 [0.41 – 1.12; 0.28]	0.53 [0.47 – 3.36; 0.13]
COx-a_L	0.11 [0.1 – 0.12; 0.02]	0.09 [0.08 – 0.09; 0.01]	0.08 [0.07 – 0.09; 0.01]	0.08 [0.07 – 0.08; 0.01]	0.07 [0.07 – 0.08; 0.01]	0.07 [0.06 – 0.07; 0.01]	0.07 [0.07 – 0.08; 0.01]	0.08 [0.07 – 0.08; 0.01]	0.07 [0.07 – 0.07; 0]
COx-a_R	0.1 [0.09 – 0.12; 0.02]	0.08 [0.08 – 0.09; 0.01]	0.08 [0.07 – 0.09; 0.01]	0.07 [0.07 – 0.08; 0.01]	0.07 [0.07 – 0.08; 0.01]	0.07 [0.07 – 0.07; 0.01]	0.07 [0.07 – 0.08; 0.01]	0.07 [0.07 – 0.08; 0.01]	0.07 [0.07 – 0.07; 0]

COx, cerebral oximetry index with cerebral perfusion pressure; COx-a, cerebral oximetry index with arterial blood pressure; HC, healthy control volunteer group; IQR, interquartile range; MAD, median absolute deviation; RMSE, root mean squared error; rSO_2, regional cerebral oxygen saturation; SP, elective spinal surgery patient group; TBI, traumatic brain injury patient group.

File S6f: Windowed-Point – Root Mean Squared Error of rSO_2 and COx/COx-a in All Populations using 1-Minute Temporal Resolution

RMSE of Physiologic Variable	Median [IQR; MAD]								
	5-Minute Window	10-Minute Window	15-Minute Window	30-Minute Window	1-Hour Window	2-Hour Window	6-Hour Window	12-Hour Window	1-Day Window
**HC Population**									
rSO_2_L	1.26 [0.91 – 1.71; 0.55]	1.12 [0.82 – 1.48; 0.5]	0.96 [0.73 – 1.29; 0.38]	0.84 [0.66 – 1.2; 0.34]	–	–	–	–	–
rSO_2_R	1.44 [1.08 – 1.84; 0.55]	1.23 [0.97 – 1.6; 0.45]	1.11 [0.9 – 1.45; 0.41]	0.83 [0.54 – 1.2; 0.49]	–	–	–	–	–
COx-a_L	0.26 [0.2 – 0.32; 0.09]	0.21 [0.16 – 0.27; 0.07]	0.18 [0.14 – 0.22; 0.06]	0.14 [0.12 – 0.19; 0.06]	–	–	–	–	–
COx-a_R	0.25 [0.2 – 0.33; 0.09]	0.21 [0.17 – 0.26; 0.07]	0.19 [0.14 – 0.23; 0.06]	0.17 [0.12 – 0.2; 0.06]	–	–	–	–	–
**SP Population**									
rSO_2_L	1.75 [1.17 – 3.74; 1.01]	1.6 [1.21 – 3.88; 0.79]	1.36 [1.13 – 3.02; 0.73]	1.1 [0.89 – 2.01; 0.6]	0.91 [0.58 – 1.79; 0.63]	1.06 [0.69 – 1.95; 0.83]	8.08 [5.87 – 10.29; 6.56]	–	–
rSO_2_R	1.45 [1.14 – 2.55; 0.72]	1.44 [1.12 – 2.22; 0.89]	1.35 [0.96 – 3.57; 0.74]	1.21 [0.87 – 2.21; 0.56]	0.84 [0.59 – 1.2; 0.46]	1.07 [0.57 – 1.4; 0.7]	11.1 [9.73 – 12.48; 4.07]	–	–
COx-a_L	0.34 [0.3 – 0.43; 0.08]	0.31 [0.28 – 0.4; 0.07]	0.3 [0.25 – 0.36; 0.09]	0.24 [0.21 – 0.29; 0.07]	0.23 [0.19 – 0.27; 0.06]	0.22 [0.19 – 0.28; 0.09]	0.21 [0.21 – 0.21; 0]	–	–
COx-a_R	0.35 [0.32 – 0.42; 0.07]	0.33 [0.29 – 0.37; 0.05]	0.3 [0.27 – 0.32; 0.05]	0.26 [0.23 – 0.3; 0.06]	0.22 [0.2 – 0.25; 0.04]	0.21 [0.18 – 0.26; 0.06]	0.2 [0.19 – 0.21; 0.04]	–	–
**TBI Population**									
rSO_2_L	1.71 [1.18 – 3.42; 1.06]	2.14 [1.38 – 3.96; 1.41]	1.94 [1.24 – 4.16; 1.46]	1.88 [1.21 – 4.58; 1.4]	2.06 [1.14 – 6.83; 1.7]	1.97 [1.04 – 2.28E+04; 1.93]	1.45 [0.92 – 1.28E+08; 1.28]	1.35 [0.88 – 10.08; 1.1]	1.36 [0.9 – 2.72; 1.15]
rSO_2_R	0.29 [0.25 – 0.32; 0.05]	0.29 [0.24 – 0.33; 0.07]	0.25 [0.22 – 0.3; 0.05]	0.21 [0.2 – 0.24; 0.03]	0.19 [0.18 – 0.21; 0.02]	0.18 [0.17 – 0.19; 0.02]	0.17 [0.16 – 0.18; 0.02]	0.17 [0.16 – 0.18; 0.02]	0.17 [0.15 – 0.18; 0.02]
COx_L	0.27 [0.25 – 0.31; 0.04]	0.28 [0.23 – 0.31; 0.06]	0.25 [0.22 – 0.28; 0.04]	0.21 [0.2 – 0.23; 0.02]	0.19 [0.18 – 0.2; 0.02]	0.18 [0.17 – 0.2; 0.02]	0.17 [0.16 – 0.18; 0.02]	0.17 [0.15 – 0.18; 0.02]	0.16 [0.15 – 0.18; 0.02]
COx_R	1.7 [1.05 – 3.35; 1.52]	2.33 [1.24 – 4.01; 1.83]	2.36 [1.27 – 4.87; 1.86]	2.2 [1.16 – 4.31; 2.02]	2.43 [1.1 – 11.72; 2.51]	2.25 [0.95 – 676.42; 2.46]	1.83 [0.83 – 6.13E+08; 2.01]	1.98 [0.91 – 4.74; 2.06]	1.95 [0.84 – 3.84; 1.81]
COx-a_L	0.28 [0.25 – 0.31; 0.05]	0.28 [0.25 – 0.32; 0.06]	0.25 [0.22 – 0.3; 0.05]	0.21 [0.2 – 0.25; 0.03]	0.19 [0.18 – 0.22; 0.03]	0.18 [0.17 – 0.19; 0.02]	0.17 [0.16 – 0.19; 0.02]	0.17 [0.15 – 0.19; 0.02]	0.16 [0.15 – 0.18; 0.02]
COx-a_R	0.28 [0.25 – 0.3; 0.04]	0.29 [0.24 – 0.33; 0.06]	0.26 [0.23 – 0.29; 0.04]	0.22 [0.2 – 0.23; 0.03]	0.19 [0.18 – 0.21; 0.02]	0.18 [0.17 – 0.19; 0.02]	0.17 [0.16 – 0.18; 0.02]	0.17 [0.15 – 0.18; 0.02]	0.16 [0.15 – 0.18; 0.02]

COx, cerebral oximetry index with cerebral perfusion pressure; COx-a, cerebral oximetry index with arterial blood pressure; HC, healthy control volunteer group; IQR, interquartile range; MAD, median absolute deviation; RMSE, root mean squared error; rSO_2, regional cerebral oxygen saturation; SP, elective spinal surgery patient group; TBI, traumatic brain injury patient group.

File S6g: Windowed-Point – Root Mean Squared Error of rSO_2 and COx/COx-a in All Populations using 5-Minute Temporal Resolution

RMSE of Physiologic Variable	Median [IQR; MAD]							
	**10-Minute Window**	**15-Minute Window**	**30-Minute Window**	**1-Hour Window**	**2-Hour Window**	**6-Hour Window**	**12-Hour Window**	**1-Day Window**
**HC Population**								
rSO_2_L	0.98 [0.63 – 1.25; 0.49]	1.01 [0.7 – 1.56; 0.58]	1.89 [1.37 – 2.52; 0.93]	–	–	–	–	–
rSO_2_R	1.06 [0.69 – 1.48; 0.58]	1.16 [0.78 – 1.81; 0.68]	1.73 [1.12 – 3.53; 2.02]	–	–	–	–	–
COx-a_L	0.25 [0.18 – 0.33; 0.11]	0.27 [0.18 – 0.35; 0.13]	0.29 [0.24 – 0.57; 0.16]	–	–	–	–	–
COx-a_R	0.25 [0.16 – 0.33; 0.13]	0.29 [0.18 – 0.42; 0.18]	0.31 [0.19 – 0.64; 0.27]	–	–	–	–	–
**SP Population**								
rSO_2_L	2.06 [1.56 – 3.41; 1.4]	2.61 [1.51 – 4.63; 1.87]	2.83 [1.77 – 3.72; 1.51]	1.99 [1.28 – 3.89; 1.53]	1.68 [1.25 – 3.46; 0.95]	14.76 [8.76 – 20.77; 17.8]	–	–
rSO_2_R	2.05 [1.41 – 3.02; 1.08]	3.04 [1.82 – 4.8; 2.11]	2.72 [1.89 – 3.75; 1.27]	2.19 [1.33 – 3.09; 1.33]	1.94 [1.28 – 2.89; 1.32]	3422.82 [1713.18 – 5132.45; 5069.41]	–	–
COx-a_L	0.38 [0.34 – 0.49; 0.11]	0.5 [0.4 – 0.59; 0.14]	0.44 [0.41 – 0.56; 0.13]	0.38 [0.31 – 0.48; 0.12]	0.35 [0.27 – 0.41; 0.11]	0.3 [0.29 – 0.3; 0.02]	–	–
COx-a_R	0.4 [0.36 – 0.48; 0.08]	0.48 [0.43 – 0.57; 0.12]	0.45 [0.37 – 0.56; 0.14]	0.43 [0.35 – 0.5; 0.12]	0.36 [0.3 – 0.42; 0.1]	0.21 [0.19 – 0.24; 0.06]	–	–
**TBI Population**								
rSO_2_L	1.95 [1.31 – 3.71; 1.39]	2.31 [1.56 – 4.38; 1.45]	3.19 [1.99 – 5.82; 2.37]	3.48 [2.15 – 6.7; 2.45]	3.18 [1.74 – 6.41; 2.4]	2.27 [1.33 – 5.72; 1.89]	1.89 [1.32 – 5.14; 1.36]	1.92 [1.38 – 4.55; 1.39]
rSO_2_R	0.31 [0.29 – 0.33; 0.03]	0.38 [0.35 – 0.41; 0.04]	0.42 [0.37 – 0.49; 0.09]	0.36 [0.33 – 0.43; 0.06]	0.31 [0.28 – 0.35; 0.04]	0.27 [0.25 – 0.3; 0.03]	0.26 [0.25 – 0.29; 0.03]	0.26 [0.24 – 0.28; 0.03]
COx_L	0.31 [0.28 – 0.33; 0.04]	0.37 [0.34 – 0.4; 0.05]	0.42 [0.37 – 0.46; 0.07]	0.36 [0.31 – 0.41; 0.07]	0.31 [0.28 – 0.33; 0.05]	0.27 [0.25 – 0.29; 0.04]	0.26 [0.24 – 0.28; 0.03]	0.25 [0.23 – 0.27; 0.03]
COx_R	2.07 [1.14 – 3.67; 1.58]	2.43 [1.29 – 4.56; 1.96]	3.29 [1.93 – 5.37; 2.22]	3.46 [1.88 – 6.48; 2.93]	2.88 [1.6 – 6.35; 2.48]	2.3 [1.24 – 4.94; 1.98]	2.28 [1.36 – 5.81; 2.21]	2.47 [1.17 – 5.07; 2.23]
COx-a_L	0.31 [0.28 – 0.34; 0.04]	0.37 [0.35 – 0.41; 0.04]	0.41 [0.36 – 0.49; 0.08]	0.35 [0.33 – 0.43; 0.06]	0.3 [0.28 – 0.35; 0.05]	0.27 [0.26 – 0.29; 0.03]	0.26 [0.25 – 0.28; 0.03]	0.25 [0.24 – 0.28; 0.03]
COx-a_R	0.3 [0.28 – 0.34; 0.05]	0.36 [0.34 – 0.4; 0.04]	0.41 [0.38 – 0.46; 0.06]	0.37 [0.32 – 0.43; 0.08]	0.31 [0.28 – 0.35; 0.05]	0.27 [0.24 – 0.29; 0.03]	0.26 [0.23 – 0.28; 0.04]	0.25 [0.23 – 0.28; 0.04]

COx, cerebral oximetry index with cerebral perfusion pressure; COx-a, cerebral oximetry index with arterial blood pressure; HC, healthy control volunteer group; IQR, interquartile range; MAD, median absolute deviation; RMSE, root mean squared error; rSO_2, regional cerebral oxygen saturation; SP, elective spinal surgery patient group; TBI, traumatic brain injury patient group.

File S6h: Windowed-Interval – Root Mean Squared Error of rSO_2 and COx/COx-a in All Populations using 10-Second Temporal Resolution

RMSE of Physiologic Variable	Median [IQR; MAD]								
	5-Minute W&I	10-Minute W&I	15-Minute W&I	30-Minute W&I	1-Hour W&I	2-Hour W&I	6-Hour W&I	12-Hour W&I	1-Day W&I
**HC Population**									
rSO_2_L	1.57 [1.24 – 2.13; 0.58]	1.55 [1.22 – 2.12; 0.61]	1.67 [1.22 – 2.05; 0.59]	–	–	–	–	–	–
rSO_2_R	1.72 [1.39 – 2.18; 0.57]	1.67 [1.32 – 2.13; 0.65]	1.75 [1.36 – 2.23; 0.64]	–	–	–	–	–	–
COx-a_L	0.32 [0.24 – 0.4; 0.11]	0.32 [0.25 – 0.4; 0.11]	0.35 [0.23 – 0.44; 0.16]	–	–	–	–	–	–
COx-a_R	0.33 [0.26 – 0.43; 0.13]	0.32 [0.25 – 0.4; 0.12]	0.33 [0.27 – 0.39; 0.09]	–	–	–	–	–	–
**SP Population**									
rSO_2_L	5.94 [2.46 – 85.78; 6.38]	3.73 [2.27 – 12.41; 3.32]	3.78 [2.38 – 5.66; 2.16]	4.33 [3 – 5.29; 1.94]	3.91 [3.22 – 5.39; 2.09]	5.99 [4.43 – 9.43; 4.64]	–	–	–
rSO_2_R	3.86 [2.09 – 19.69; 3.4]	2.85 [2.33 – 5.6; 1.31]	3.54 [2.58 – 5; 1.53]	4.03 [3.3 – 5.72; 1.45]	3.97 [2.45 – 5.31; 2.22]	9.79 [5.93 – 11.74; 5.79]	–	–	–
COx-a_L	0.56 [0.48 – 0.73; 0.17]	0.58 [0.47 – 0.71; 0.16]	0.55 [0.45 – 0.67; 0.18]	0.5 [0.41 – 0.62; 0.15]	0.45 [0.39 – 0.56; 0.12]	0.37 [0.34 – 0.37; 0]	–	–	–
COx-a_R	0.65 [0.5 – 1.38; 0.3]	0.59 [0.48 – 0.81; 0.22]	0.58 [0.48 – 0.7; 0.17]	0.49 [0.43 – 0.57; 0.12]	0.44 [0.4 – 0.5; 0.09]	0.43 [0.39 – 0.45; 0.05]	–	–	–
**TBI Population**									
rSO_2_L	1848.16 [41.5 – 9.47E+04; 2737.15]	127.47 [3.39 – 2.74E+05; 186.67]	13.03 [2.94 – 2.10E+04; 16.63]	5.67 [3.32 – 63.15; 5.3]	3.97 [2.88 – 6.35; 2.11]	4.17 [3.09 – 7; 2.3]	4.98 [3.64 – 7.88; 2.69]	5.73 [4.11 – 9.49; 3.14]	5.83 [4.24 – 10.83; 3.13]
rSO_2_R	0.66 [0.46 – 3.31; 0.35]	0.55 [0.43 – 1.06; 0.21]	0.53 [0.43 – 0.91; 0.2]	0.45 [0.4 – 0.5; 0.08]	0.42 [0.38 – 0.45; 0.06]	0.39 [0.36 – 0.43; 0.05]	0.38 [0.34 – 0.41; 0.06]	0.36 [0.33 – 0.4; 0.06]	0.36 [0.33 – 0.4; 0.05]
COx_L	0.95 [0.5 – 6.91; 0.78]	0.52 [0.44 – 1.11; 0.19]	0.47 [0.42 – 0.69; 0.12]	0.43 [0.39 – 0.5; 0.07]	0.4 [0.37 – 0.44; 0.05]	0.39 [0.35 – 0.43; 0.05]	0.36 [0.32 – 0.4; 0.06]	0.34 [0.32 – 0.39; 0.05]	0.33 [0.32 – 0.36; 0.03]
COx_R	1030.93 [52.37 – 1.74E+04; 1523.41]	80.05 [7.41 – 4.56E+04; 115.18]	73 [3.81 – 1.35E+04; 105.61]	5.72 [2.49 – 40.54; 5.8]	3.83 [2.6 – 9.08; 2.28]	4.24 [3.22 – 8.03; 2.45]	5.2 [3.89 – 8.59; 2.74]	6.14 [4.42 – 10.57; 3.65]	6.83 [4.92 – 11.46; 3.54]
COx-a_L	0.63 [0.48 – 2.77; 0.36]	0.57 [0.44 – 1.29; 0.23]	0.51 [0.43 – 0.8; 0.15]	0.44 [0.41 – 0.49; 0.06]	0.41 [0.39 – 0.45; 0.05]	0.38 [0.36 – 0.42; 0.05]	0.36 [0.34 – 0.39; 0.04]	0.36 [0.33 – 0.4; 0.06]	0.36 [0.33 – 0.39; 0.04]
COx-a_R	0.68 [0.51 – 2.92; 0.4]	0.52 [0.43 – 1.11; 0.21]	0.5 [0.45 – 0.89; 0.15]	0.44 [0.4 – 0.48; 0.07]	0.4 [0.36 – 0.44; 0.05]	0.38 [0.34 – 0.42; 0.06]	0.35 [0.31 – 0.39; 0.06]	0.34 [0.31 – 0.38; 0.05]	0.34 [0.3 – 0.38; 0.06]

COx, cerebral oximetry index with cerebral perfusion pressure; COx-a, cerebral oximetry index with arterial blood pressure; HC, healthy control volunteer group; IQR, interquartile range; MAD, median absolute deviation; RMSE, root mean squared error; rSO_2, regional cerebral oxygen saturation; SP, elective spinal surgery patient group; TBI, traumatic brain injury patient group; W&I, window and interval.

File S6i: Windowed-Interval – Root Mean Squared Error of rSO_2 and COx/COx-a in All Populations using 1-Minute Temporal Resolution

RMSE of Physiologic Variable	Median [IQR; MAD]								
	5-Minute W&I	10-Minute W&I	15-Minute W&I	30-Minute W&I	1-Hour W&I	2-Hour W&I	6-Hour W&I	12-Hour W&I	1-Day W&I
**HC Population**									
rSO_2_L	1.64 [1.2 – 2.31; 0.71]	1.56 [1.11 – 2.19; 0.76]	1.54 [1.12 – 1.97; 0.63]	–	–	–	–	–	–
rSO_2_R	1.79 [1.28 – 2.49; 0.94]	1.69 [1.15 – 2.19; 0.78]	1.63 [1.17 – 2.27; 0.87]	–	–	–	–	–	–
COx-a_L	0.46 [0.33 – 0.7; 0.26]	0.35 [0.26 – 0.48; 0.18]	0.31 [0.25 – 0.42; 0.14]	–	–	–	–	–	–
COx-a_R	0.39 [0.33 – 0.63; 0.14]	0.34 [0.26 – 0.48; 0.17]	0.31 [0.23 – 0.37; 0.11]	–	–	–	–	–	–
**SP Population**									
rSO_2_L	3.7 [2.16 – 9.08; 2.92]	3.72 [2.05 – 7.44; 2.89]	4.07 [2.69 – 7.86; 2.88]	4.78 [3.37 – 6.87; 2.54]	4.88 [3.51 – 6.84; 2.18]	7.95 [5.13 – 10.41; 7.3]	–	–	–
rSO_2_R	3.27 [2.5 – 5.81; 1.82]	4.68 [3.3 – 6.41; 2.47]	3.56 [2.64 – 6.8; 2.17]	4.7 [3.14 – 6.28; 2.45]	4.37 [2.43 – 5.57; 2.81]	9.07 [5.52 – 11.26; 6.5]	–	–	–
COx-a_L	0.71 [0.54 – 1.09; 0.3]	0.68 [0.52 – 0.99; 0.3]	0.58 [0.47 – 0.69; 0.16]	0.49 [0.4 – 0.55; 0.1]	0.42 [0.33 – 0.49; 0.13]	0.38 [0.33 – 0.4; 0.04]	–	–	–
COx-a_R	0.77 [0.61 – 0.92; 0.24]	0.62 [0.53 – 0.79; 0.2]	0.58 [0.46 – 0.71; 0.19]	0.48 [0.4 – 0.55; 0.13]	0.42 [0.34 – 0.47; 0.11]	0.44 [0.39 – 0.45; 0.03]	–	–	–
**TBI Population**									
rSO_2_L	2.8 [2 – 5.2; 1.52]	6.51 [3.09 – 16.57; 5.95]	6.93 [3.79 – 16.04; 5.88]	7.38 [3.44 – 26.46; 6.81]	5.08 [3.12 – 19.71; 3.61]	5.15 [3.51 – 13.06; 3.31]	5.12 [3.72 – 7.93; 3]	5.68 [3.95 – 9.58; 3.43]	5.89 [4.3 – 10.46; 2.84]
rSO_2_R	0.62 [0.52 – 0.73; 0.16]	0.91 [0.52 – 2.56; 0.67]	0.69 [0.48 – 2.23; 0.38]	0.47 [0.4 – 0.61; 0.11]	0.39 [0.35 – 0.44; 0.07]	0.35 [0.33 – 0.39; 0.04]	0.34 [0.32 – 0.38; 0.05]	0.34 [0.32 – 0.37; 0.04]	0.35 [0.32 – 0.37; 0.04]
COx_L	0.58 [0.49 – 0.7; 0.15]	0.94 [0.56 – 2.46; 0.66]	0.66 [0.52 – 2.73; 0.33]	0.47 [0.4 – 0.61; 0.15]	0.38 [0.34 – 0.42; 0.06]	0.35 [0.32 – 0.39; 0.05]	0.32 [0.3 – 0.35; 0.03]	0.32 [0.3 – 0.35; 0.04]	0.32 [0.3 – 0.35; 0.03]
COx_R	2.93 [1.54 – 6.3; 2.4]	5.05 [3.13 – 14.08; 4.19]	7.36 [3.41 – 42.77; 7.23]	16.91 [4.78 – 119.44; 22.6]	6.15 [3.1 – 19.3; 5.91]	5.37 [3.36 – 14.22; 4]	5.37 [3.99 – 9.9; 3.53]	5.97 [4.3 – 10; 3.29]	6.61 [4.82 – 9.93; 3.51]
COx-a_L	0.58 [0.49 – 0.69; 0.15]	0.98 [0.54 – 2.61; 0.78]	0.67 [0.51 – 2.46; 0.33]	0.45 [0.41 – 0.66; 0.12]	0.38 [0.36 – 0.42; 0.04]	0.36 [0.33 – 0.38; 0.04]	0.34 [0.32 – 0.37; 0.04]	0.34 [0.31 – 0.38; 0.04]	0.35 [0.32 – 0.38; 0.04]
COx-a_R	0.57 [0.48 – 0.67; 0.15]	0.87 [0.56 – 1.62; 0.53]	0.65 [0.5 – 1.92; 0.31]	0.44 [0.39 – 0.63; 0.11]	0.38 [0.34 – 0.41; 0.05]	0.35 [0.31 – 0.38; 0.05]	0.32 [0.3 – 0.36; 0.04]	0.33 [0.29 – 0.36; 0.05]	0.33 [0.3 – 0.36; 0.05]

COx, cerebral oximetry index with cerebral perfusion pressure; COx-a, cerebral oximetry index with arterial blood pressure; HC, healthy control volunteer group; IQR, interquartile range; MAD, median absolute deviation; RMSE, root mean squared error; rSO_2, regional cerebral oxygen saturation; SP, elective spinal surgery patient group; TBI, traumatic brain injury patient group; W&I, window and interval.

File S6j: Windowed-Interval – Root Mean Squared Error of rSO_2 and COx/COx-a in All Populations using 5-Minute Temporal Resolution

RMSE of Physiologic Variable	Median [IQR; MAD]							
	10-Minute W&I	15-Minute W&I	30-Minute W&I	1-Hour W&I	2-Hour W&I	6-Hour W&I	12-Hour W&I	1-Day W&I
**HC Population**								
rSO_2_L	1.13 [0.73 – 1.52; 0.59]	1.27 [0.86 – 1.85; 0.82]	–	–	–	–	–	–
rSO_2_R	1.21 [0.72 – 1.84; 0.84]	1.39 [0.83 – 2.14; 0.93]	–	–	–	–	–	–
COx-a_L	0.29 [0.19 – 0.36; 0.11]	0.31 [0.2 – 0.46; 0.19]	–	–	–	–	–	–
COx-a_R	0.26 [0.17 – 0.37; 0.14]	0.26 [0.2 – 0.37; 0.1]	–	–	–	–	–	–
**SP Population**								
rSO_2_L	2.88 [1.69 – 4.16; 1.81]	4.23 [2.36 – 8.02; 3.53]	4.52 [3.23 – 7.08; 2.87]	4.14 [2.64 – 9.41; 3.26]	5.2 [3.52 – 8.18; 5]	–	–	–
rSO_2_R	3.07 [1.95 – 3.88; 1.43]	4.77 [3.39 – 10.61; 3.18]	4.81 [3.84 – 11.31; 3.65]	4.3 [2.62 – 7.94; 3.72]	7.53 [4.91 – 9.69; 6.41]	–	–	–
COx-a_L	0.42 [0.37 – 0.5; 0.09]	0.52 [0.45 – 0.65; 0.16]	0.48 [0.37 – 0.86; 0.17]	0.39 [0.33 – 0.61; 0.16]	0.33 [0.28 – 0.41; 0.15]	–	–	–
COx-a_R	0.43 [0.37 – 0.48; 0.08]	0.55 [0.48 – 0.6; 0.11]	0.44 [0.38 – 0.58; 0.13]	0.47 [0.33 – 0.66; 0.21]	0.31 [0.29 – 0.35; 0.06]	–	–	–
**TBI Population**								
rSO_2_L	2.25 [1.63 – 3.9; 1.39]	2.93 [2.04 – 5.11; 1.73]	6.41 [3.17 – 11.36; 5.15]	8.04 [5.07 – 39.39; 7.21]	6.24 [4.15 – 18.11; 4.48]	5.45 [3.59 – 11.74; 3.57]	5.82 [4.17 – 9.6; 3.25]	6.04 [4.42 – 10.32; 2.77]
rSO_2_R	0.33 [0.32 – 0.37; 0.03]	0.44 [0.4 – 0.49; 0.06]	0.63 [0.45 – 0.87; 0.27]	0.41 [0.34 – 0.8; 0.15]	0.34 [0.3 – 0.39; 0.06]	0.3 [0.26 – 0.32; 0.04]	0.3 [0.27 – 0.32; 0.04]	0.3 [0.26 – 0.32; 0.04]
COx_L	0.33 [0.31 – 0.36; 0.04]	0.43 [0.4 – 0.47; 0.05]	0.61 [0.45 – 0.84; 0.25]	0.4 [0.35 – 0.57; 0.11]	0.32 [0.29 – 0.35; 0.05]	0.28 [0.26 – 0.3; 0.04]	0.28 [0.26 – 0.3; 0.03]	0.27 [0.25 – 0.3; 0.03]
COx_R	2.43 [1.48 – 4; 1.79]	3 [1.86 – 5.5; 2.16]	5.27 [3.34 – 12.31; 3.85]	6.75 [3.47 – 23.61; 6.58]	6.44 [3.66 – 18.99; 5.58]	5.3 [3.8 – 9.38; 3.34]	6.44 [4.32 – 10.34; 3.6]	7.07 [4.85 – 9.47; 3.33]
COx-a_L	0.34 [0.31 – 0.37; 0.04]	0.44 [0.41 – 0.48; 0.04]	0.63 [0.42 – 1.29; 0.35]	0.44 [0.37 – 0.72; 0.17]	0.33 [0.3 – 0.38; 0.06]	0.3 [0.27 – 0.33; 0.04]	0.29 [0.27 – 0.31; 0.03]	0.3 [0.27 – 0.31; 0.03]
COx-a_R	0.33 [0.3 – 0.36; 0.05]	0.43 [0.39 – 0.47; 0.06]	0.58 [0.47 – 0.78; 0.19]	0.41 [0.34 – 0.59; 0.14]	0.32 [0.29 – 0.36; 0.05]	0.28 [0.26 – 0.31; 0.03]	0.27 [0.25 – 0.3; 0.04]	0.27 [0.25 – 0.3; 0.04]

COx, cerebral oximetry index with cerebral perfusion pressure; COx-a, cerebral oximetry index with arterial blood pressure; HC, healthy control volunteer group; IQR, interquartile range; MAD, median absolute deviation; RMSE, root mean squared error; rSO_2, regional cerebral oxygen saturation; SP, elective spinal surgery patient group; TBI, traumatic brain injury patient group; W&I, window and interval.
