# Supplementary material for: Time-Series Autoregressive Models for Point and Interval Forecasting of Raw and Derived Commercial Near-Infrared Spectroscopy Measures: An Exploratory Cranial Trauma and Healthy Control Analysis
Source: Bioengineering (Basel). 2025 Jun 21;12(7):682. doi: 10.3390/bioengineering12070682 (PMC12292983; doi:10.3390/bioengineering12070682)
Supplement: Supplementary file 1 [file bioengineering-12-00682-s001.zip › File S7.pdf]

File S7 – Pearson Correlation Analysis

File S7 – Table of Contents

File S7a: Anchored-Point – Pearson Correlation Analysis of rSO_2 and COx/COx-a in All Populations using 10-Second Temporal Resolution 2

File S7b: Anchored-Point – Pearson Correlation Analysis of rSO_2 and COx/COx-a in All Populations using 1-Minute and 5-Minute Temporal Resolutions.............. 3

File S7c: Anchored-Interval – Pearson Correlation Analysis of rSO_2 and COx/COx-a in All Populations using 10-Second Temporal Resolution................................ 4

File S7d: Anchored-Interval – Pearson Correlation Analysis of rSO_2 and COx/COx-a in All Populations using 1-Minute Temporal Resolution................................. 5

File S7e: Anchored-Interval – Pearson Correlation Analysis of rSO_2 and COx/COx-a in All Populations using 5-Minute Temporal Resolution................................. 6

File S7f: Windowed-Point – Pearson Correlation Analysis of rSO_2 and COx/COx-a in All Populations using 10-Second Temporal Resolution 7

File S7g: Windowed-Point – Pearson Correlation Analysis of rSO_2 and COx/COx-a in All Populations using 1-Minute Temporal Resolution................................... 8

File S7h: Windowed-Point – Pearson Correlation Analysis of rSO_2 and COx/COx-a in All Populations using 5-Minute Temporal Resolution................................... 9

File S7i: Windowed-Interval – Pearson Correlation Analysis of rSO_2 and COx/COx-a in All Populations using 10-Second Temporal Resolution............................ 10

File S7j: Windowed-Interval – Pearson Correlation Analysis of rSO_2 and COx/COx-a in All Populations using 1-Minute Temporal Resolution.............................. 11

File S7k: Windowed-Interval – Pearson Correlation Analysis of rSO_2 and COx/COx-a in All Populations using 5-Minute Temporal Resolution.............................. 12

File S7a: Anchored-Point – Pearson Correlation Analysis of rSO_2 and COx/COx-a in All Populations using 10-Second Temporal Resolution

Physiologic Variable	Value	Median [IQR]		
		HC	SP	TBI
rSO_2_L	*r*	0.07 [-0.11 – 0.24]	-0.02 [-0.07 – 0.05]	0 [-0.02 – 0.03]
	p	0.37 [0.03 – 0.58]	0.37 [0.05 – 0.68]	0.13 [0 – 0.76]
rSO_2_R	*r*	0.06 [-0.07 – 0.21]	0.01 [-0.05 – 0.08]	0 [-0.03 – 0.03]
	p	0.32 [0.12 – 0.7]	0.23 [0.03 – 0.72]	0.12 [0 – 0.56]
COx_L	*r*	–	–	0.02 [0 – 0.07]
	p	–	–	0.01 [0 – 0.45]
COx_R	*r*	–	–	0.02 [-0.01 – 0.06]
	p	–	–	0.04 [0 – 0.29]
COx-a_L	*r*	0.11 [-0.14 – 0.38]	0.1 [-0.02 – 0.23]	0.03 [0 – 0.06]
	p	0.12 [0 – 0.49]	0.1 [0 – 0.51]	0.02 [0 – 0.27]
COx-a_R	*r*	0.04 [-0.15 – 0.36]	0.15 [-0.03 – 0.24]	0.02 [0 – 0.07]
	p	0.22 [0 – 0.57]	0 [0 – 0.18]	0.05 [0 – 0.47]

COx, cerebral oximetry index with cerebral perfusion pressure; COx-a, cerebral oximetry index with arterial blood pressure; HC, healthy control volunteer group; IQR, interquartile range; r-value, Pearson correlation coefficient; rSO_2, regional cerebral oxygen saturation; SP, elective spinal surgery patient group; TBI, traumatic brain injury patient group.

File S7b: Anchored-Point – Pearson Correlation Analysis of rSO_2 and COx/COx-a in All Populations using 1-Minute and 5-Minute Temporal Resolutions

Physiologic Variable	Value	Median [IQR]		
		HC	SP	TBI
**1-Minute Temporal Resolution**				
rSO_2_L	*r*	0.18 [-0.15 – 0.54]	-0.04 [-0.22 – 0.12]	0 [-0.05 – 0.04]
	p	0.42 [0.16 – 0.75]	0.37 [0.07 – 0.64]	0.16 [0.01 – 0.6]
rSO_2_R	*r*	0.09 [-0.23 – 0.47]	-0.02 [-0.18 – 0.13]	0 [-0.06 – 0.06]
	p	0.46 [0.22 – 0.73]	0.4 [0.1 – 0.73]	0.19 [0 – 0.62]
COx_L	*r*	–	–	0.04 [0 – 0.09]
	p	–	–	0.23 [0.05 – 0.59]
COx_R	*r*	–	–	0.04 [0 – 0.07]
	p	–	–	0.3 [0.05 – 0.63]
COx-a_L	*r*	0.17 [-0.27 – 0.64]	0.1 [-0.08 – 0.22]	0.05 [0.02 – 0.1]
	p	0.22 [0.07 – 0.68]	0.29 [0.05 – 0.61]	0.19 [0.05 – 0.53]
COx-a_R	*r*	0.28 [-0.26 – 0.7]	0.3 [0.07 – 0.37]	0.02 [-0.02 – 0.07]
	p	0.22 [0.07 – 0.6]	0.15 [0.03 – 0.55]	0.3 [0.02 – 0.67]
**5-Minute Temporal Resolution**				
rSO_2_L	*r*	NA	-0.07 [-0.48 – 0.25]	0 [-0.06 – 0.09]
	p	NA	0.32 [0.1 – 0.73]	0.39 [0.02 – 0.76]
rSO_2_R	*r*	NA	0.16 [-0.12 – 0.57]	0 [-0.06 – 0.13]
	p	NA	0.4 [0.15 – 0.68]	0.22 [0 – 0.79]
COx_L	*r*	–	–	0.03 [-0.04 – 0.11]
	p	–	–	0.44 [0.14 – 0.71]
COx_R	*r*	–	–	0.02 [-0.06 – 0.09]
	p	–	–	0.4 [0.14 – 0.68]
COx-a_L	*r*	NA	0.15 [-0.29 – 0.4]	0.03 [-0.05 – 0.08]
	p	NA	0.42 [0.21 – 0.64]	0.39 [0.17 – 0.68]
COx-a_R	*r*	NA	-0.16 [-0.41 – 0.18]	0.02 [-0.04 – 0.07]
	p	NA	0.49 [0.22 – 0.72]	0.49 [0.19 – 0.78]

AFR, absolute forecast residual; COx, cerebral oximetry index with cerebral perfusion pressure; COx-a, cerebral oximetry index with arterial blood pressure; HC, healthy control volunteer group; IQR, interquartile range; MAD, median absolute deviation; r-value, Pearson correlation coefficient; rSO_2, regional cerebral oxygen saturation; SP, elective spinal surgery patient group; TBI, traumatic brain injury patient group.

File S7c: Anchored-Interval – Pearson Correlation Analysis of rSO_2 and COx/COx-a in All Populations using 10-Second Temporal Resolution

Physiologic Variable	Value	Median [IQR]								
		5-Minute Interval	10-Minute Interval	15-Minute Interval	30-Minute Interval	1-Hour Interval	2-Hour Interval	6-Hour Interval	12-Hour Interval	1-Day Interval
**HC Population**										
rSO_2_L	*r*	0.13 [-0.04 – 0.38]	0.4 [0.4 – 0.4]	–	–	–	–	–	–	–
	p	0.16 [0.01 – 0.59]	**0 [0 – 0]**	–	–	–	–	–	–	–
rSO_2_R	*r*	0.16 [-0.06 – 0.33]	0.18 [0.18 – 0.18]	–	–	–	–	–	–	–
	p	0.15 [0.02 – 0.42]	0.17 [0.17 – 0.17]	–	–	–	–	–	–	–
COx_L	*r*	–	–	–	–	–	–	–	–	–
	p	–	–	–	–	–	–	–	–	–
COx_R	*r*	–	–	–	–	–	–	–	–	–
	p	–	–	–	–	–	–	–	–	–
COx-a_L	*r*	0.3 [0 – 0.54]	-0.07 [-0.07 – -0.07]	–	–	–	–	–	–	–
	p	0.04 [0 – 0.28]	0.61 [0.61 – 0.61]	–	–	–	–	–	–	–
COx-a_R	*r*	0.26 [-0.01 – 0.51]	-0.07 [-0.07 – -0.07]	–	–	–	–	–	–	–
	p	0.03 [0 – 0.36]	0.62 [0.62 – 0.62]	–	–	–	–	–	–	–
**SP Population**										
rSO_2_L	*r*	**0.78 [0.59 – 0.9]**	**0.71 [0.13 – 0.84]**	0.46 [0.13 – 0.83]	0.3 [-0.07 – 0.64]	0.24 [0.04 – 0.51]	–	–	–	–
	p	**0 [0 – 0]**	**0 [0 – 0]**	**0 [0 – 0]**	**0 [0 – 0.01]**	**0 [0 – 0]**	–	–	–	–
rSO_2_R	*r*	**0.81 [0.62 – 0.92]**	**0.77 [0.34 – 0.86]**	**0.56 [0.27 – 0.71]**	0.36 [0.18 – 0.58]	0.25 [0.18 – 0.5]	–	–	–	–
	p	**0 [0 – 0]**	**0 [0 – 0]**	**0 [0 – 0]**	**0 [0 – 0.01]**	**0 [0 – 0.01]**	–	–	–	–
COx_L	*r*	–	–	–	–	–	–	–	–	–
	p	–	–	–	–	–	–	–	–	–
COx_R	*r*	–	–	–	–	–	–	–	–	–
	p	–	–	–	–	–	–	–	–	–
COx-a_L	*r*	0.52 [0.44 – 0.62]	0.32 [0.2 – 0.56]	0.34 [0.23 – 0.5]	0.28 [-0.02 – 0.37]	0.29 [0.26 – 0.33]	–	–	–	–
	p	**0 [0 – 0]**	**0 [0 – 0]**	**0 [0 – 0]**	0 [0 – 0.45]	**0 [0 – 0]**	–	–	–	–
COx-a_R	*r*	0.44 [0.25 – 0.53]	0.24 [0.16 – 0.4]	0.28 [0.03 – 0.4]	0.14 [-0.05 – 0.39]	0.35 [0.06 – 0.37]	–	–	–	–
	p	**0 [0 – 0]**	**0 [0 – 0.04]**	**0 [0 – 0]**	**0 [0 – 0.05]**	**0 [0 – 0]**	–	–	–	–
**TBI Population**										
rSO_2_L	*r*	**0.85 [0.73 – 0.92]**	**0.77 [0.63 – 0.88]**	**0.72 [0.55 – 0.85]**	**0.64 [0.42 – 0.81]**	0.48 [0.26 – 0.72]	0.34 [0.13 – 0.58]	0.23 [-0.02 – 0.5]	0.15 [-0.04 – 0.36]	0.02 [-0.29 – 0.37]
	p	**0 [0 – 0]**	**0 [0 – 0]**	**0 [0 – 0]**	**0 [0 – 0]**	**0 [0 – 0]**	**0 [0 – 0]**	**0 [0 – 0]**	**0 [0 – 0]**	**0 [0 – 0]**
rSO_2_R	*r*	**0.84 [0.74 – 0.92]**	**0.78 [0.65 – 0.88]**	**0.71 [0.61 – 0.86]**	**0.63 [0.5 – 0.79]**	**0.5 [0.36 – 0.73]**	0.39 [0.27 – 0.65]	0.2 [0.02 – 0.49]	0.11 [-0.02 – 0.42]	0.13 [-0.04 – 0.44]
	p	**0 [0 – 0]**	**0 [0 – 0]**	**0 [0 – 0]**	**0 [0 – 0]**	**0 [0 – 0]**	**0 [0 – 0]**	**0 [0 – 0]**	**0 [0 – 0]**	**0 [0 – 0]**
COx_L	*r*	**0.53 [0.47 – 0.58]**	0.38 [0.33 – 0.43]	0.28 [0.23 – 0.35]	0.21 [0.13 – 0.26]	0.15 [0.08 – 0.21]	0.08 [0.01 – 0.16]	0.04 [-0.02 – 0.11]	0.02 [-0.02 – 0.08]	0.02 [-0.02 – 0.11]
	p	**0 [0 – 0]**	**0 [0 – 0]**	**0 [0 – 0]**	**0 [0 – 0]**	**0 [0 – 0]**	**0 [0 – 0.02]**	**0 [0 – 0.03]**	0 [0 – 0.18]	**0 [0 – 0.03]**
COx_R	*r*	**0.52 [0.49 – 0.58]**	0.37 [0.3 – 0.44]	0.3 [0.25 – 0.34]	0.19 [0.13 – 0.24]	0.11 [0.04 – 0.18]	0.06 [0 – 0.1]	0.03 [-0.01 – 0.09]	0.03 [-0.02 – 0.08]	0.03 [-0.03 – 0.07]
	p	**0 [0 – 0]**	**0 [0 – 0]**	**0 [0 – 0]**	**0 [0 – 0]**	**0 [0 – 0]**	0 [0 – 0.07]	0 [0 – 0.24]	0 [0 – 0.06]	**0 [0 – 0.02]**
COx-a_L	*r*	**0.53 [0.48 – 0.57]**	0.38 [0.33 – 0.42]	0.29 [0.24 – 0.34]	0.2 [0.15 – 0.24]	0.13 [0.05 – 0.18]	0.08 [0.01 – 0.13]	0.04 [-0.02 – 0.09]	0.02 [-0.01 – 0.08]	0.02 [0 – 0.07]
	p	**0 [0 – 0]**	**0 [0 – 0]**	**0 [0 – 0]**	**0 [0 – 0]**	**0 [0 – 0]**	**0 [0 – 0]**	**0 [0 – 0.01]**	0 [0 – 0.09]	0 [0 – 0.2]
COx-a_R	*r*	**0.52 [0.47 – 0.57]**	0.37 [0.31 – 0.41]	0.29 [0.22 – 0.33]	0.18 [0.13 – 0.22]	0.1 [0.04 – 0.17]	0.06 [-0.02 – 0.13]	0.02 [-0.03 – 0.09]	0.04 [0 – 0.1]	0.03 [0 – 0.07]
	p	**0 [0 – 0]**	**0 [0 – 0]**	**0 [0 – 0]**	**0 [0 – 0]**	**0 [0 – 0]**	**0 [0 – 0]**	**0 [0 – 0.04]**	**0 [0 – 0]**	**0 [0 – 0.04]**

COx, cerebral oximetry index with cerebral perfusion pressure; COx-a, cerebral oximetry index with arterial blood pressure; HC, healthy control volunteer group; IQR, interquartile range; r-value, Pearson correlation coefficient; rSO_2, regional cerebral oxygen saturation; SP, elective spinal surgery patient group; TBI, traumatic brain injury patient group.

File S7d: Anchored-Interval – Pearson Correlation Analysis of rSO_2 and COx/COx-a in All Populations using 1-Minute Temporal Resolution

Physiologic Variable	Value	Median [IQR]								
		5-Minute Interval	10-Minute Interval	15-Minute Interval	30-Minute Interval	1-Hour Interval	2-Hour Interval	6-Hour Interval	12-Hour Interval	1-Day Interval
**HC Population**										
rSO_2_L	*r*	0.27 [-0.16 – 0.62]	–	–	–	–	–	–	–	–
	p	0.33 [0.12 – 0.65]	–	–	–	–	–	–	–	–
rSO_2_R	*r*	0.15 [-0.16 – 0.49]	–	–	–	–	–	–	–	–
	p	0.48 [0.15 – 0.75]	–	–	–	–	–	–	–	–
COx_L	*r*	–	–	–	–	–	–	–	–	–
	p	–	–	–	–	–	–	–	–	–
COx_R	*r*	–	–	–	–	–	–	–	–	–
	p	–	–	–	–	–	–	–	–	–
COx-a_L	*r*	0.45 [-0.01 – 0.67]	–	–	–	–	–	–	–	–
	p	0.25 [0.09 – 0.54]	–	–	–	–	–	–	–	–
COx-a_R	*r*	0.42 [-0.13 – 0.72]	–	–	–	–	–	–	–	–
	p	0.22 [0.06 – 0.53]	–	–	–	–	–	–	–	–
**SP Population**										
rSO_2_L	*r*	**0.74 [0.62 – 0.9]**	0.54 [0.08 – 0.78]	0.45 [0.04 – 0.79]	0.31 [0.04 – 0.65]	-0.25 [-0.27 – 0.25]	–	–	–	–
	p	**0 [0 – 0]**	0.01 [0 – 0.37]	0 [0 – 0.09]	0 [0 – 0.22]	**0.01 [0.01 – 0.02]**	–	–	–	–
rSO_2_R	*r*	**0.73 [0.52 – 0.85]**	**0.63 [0.4 – 0.82]**	**0.53 [0.05 – 0.74]**	0.45 [0.2 – 0.65]	0.01 [-0.11 – 0.32]	–	–	–	–
	p	**0 [0 – 0]**	**0 [0 – 0.01]**	**0 [0 – 0.03]**	0.01 [0 – 0.14]	0.04 [0.02 – 0.49]	–	–	–	–
COx_L	*r*	–	–	–	–	–	–	–	–	–
	p	–	–	–	–	–	–	–	–	–
COx_R	*r*	–	–	–	–	–	–	–	–	–
	p	–	–	–	–	–	–	–	–	–
COx-a_L	*r*	0.51 [0.24 – 0.59]	0.3 [0.09 – 0.48]	0.19 [-0.03 – 0.37]	0.19 [-0.02 – 0.29]	0.32 [0.03 – 0.35]	–	–	–	–
	p	0 [0 – 0.2]	0.07 [0.01 – 0.51]	0.09 [0 – 0.53]	0.14 [0.01 – 0.48]	**0.01 [0 – 0.01]**	–	–	–	–
COx-a_R	*r*	0.45 [0.35 – 0.59]	0.36 [0.24 – 0.49]	0.25 [0.1 – 0.43]	0.19 [-0.01 – 0.37]	0.29 [0.07 – 0.35]	–	–	–	–
	p	**0.01 [0 – 0.03]**	0.03 [0 – 0.17]	0.05 [0.01 – 0.4]	0.15 [0.02 – 0.66]	0.01 [0.01 – 0.09]	–	–	–	–
**TBI Population**										
rSO_2_L	*r*	**0.86 [0.76 – 0.93]**	**0.78 [0.66 – 0.89]**	**0.74 [0.53 – 0.86]**	**0.64 [0.45 – 0.8]**	0.03 [-0.25 – 0.42]	0.32 [0.11 – 0.59]	0.24 [0.05 – 0.5]	0.16 [-0.02 – 0.36]	0.03 [-0.25 – 0.42]
	p	**0 [0 – 0]**	**0 [0 – 0]**	**0 [0 – 0]**	**0 [0 – 0]**	**0 [0 – 0.01]**	**0 [0 – 0]**	**0 [0 – 0]**	**0 [0 – 0.02]**	**0 [0 – 0.01]**
rSO_2_R	*r*	**0.87 [0.76 – 0.93]**	**0.8 [0.71 – 0.9]**	**0.75 [0.62 – 0.87]**	**0.67 [0.53 – 0.8]**	0.14 [-0.11 – 0.37]	0.42 [0.2 – 0.63]	0.17 [-0.05 – 0.5]	0.13 [-0.07 – 0.49]	0.14 [-0.11 – 0.37]
	p	**0 [0 – 0]**	**0 [0 – 0]**	**0 [0 – 0]**	**0 [0 – 0]**	**0 [0 – 0]**	**0 [0 – 0]**	**0 [0 – 0]**	**0 [0 – 0]**	**0 [0 – 0]**
COx_L	*r*	**0.5 [0.45 – 0.55]**	0.37 [0.29 – 0.41]	0.29 [0.22 – 0.34]	0.21 [0.15 – 0.25]	0.04 [-0.01 – 0.12]	0.08 [0.01 – 0.16]	0.04 [-0.05 – 0.11]	0.03 [-0.04 – 0.07]	0.04 [-0.01 – 0.12]
	p	**0 [0 – 0]**	**0 [0 – 0]**	**0 [0 – 0]**	**0 [0 – 0]**	0.02 [0 – 0.16]	0.03 [0 – 0.25]	0.02 [0 – 0.2]	0.04 [0 – 0.32]	0.02 [0 – 0.16]
COx_R	*r*	**0.5 [0.44 – 0.54]**	0.35 [0.29 – 0.4]	0.28 [0.22 – 0.33]	0.17 [0.09 – 0.23]	0.03 [0 – 0.07]	0.05 [-0.02 – 0.12]	0.05 [0 – 0.1]	0.02 [-0.02 – 0.08]	0.03 [0 – 0.07]
	p	**0 [0 – 0]**	**0 [0 – 0]**	**0 [0 – 0]**	**0 [0 – 0.08]**	0.01 [0 – 0.16]	0.04 [0 – 0.4]	0.1 [0 – 0.43]	0.06 [0 – 0.47]	0.01 [0 – 0.16]
COx-a_L	*r*	0.49 [0.44 – 0.54]	0.34 [0.3 – 0.4]	0.29 [0.25 – 0.33]	0.2 [0.17 – 0.24]	0.02 [-0.03 – 0.09]	0.07 [0 – 0.15]	0.03 [-0.03 – 0.09]	0.02 [-0.03 – 0.08]	0.02 [-0.03 – 0.09]
	p	**0 [0 – 0]**	**0 [0 – 0]**	**0 [0 – 0]**	**0 [0 – 0]**	0.03 [0 – 0.38]	0.03 [0 – 0.27]	0.07 [0 – 0.29]	0.1 [0.01 – 0.41]	0.03 [0 – 0.38]
COx-a_R	*r*	0.49 [0.44 – 0.52]	0.34 [0.28 – 0.4]	0.27 [0.21 – 0.31]	0.17 [0.11 – 0.22]	0.02 [0 – 0.06]	0.05 [-0.01 – 0.12]	0.03 [-0.03 – 0.09]	0.02 [-0.05 – 0.07]	0.02 [0 – 0.06]
	p	**0 [0 – 0]**	**0 [0 – 0]**	**0 [0 – 0]**	**0 [0 – 0.02]**	0.06 [0 – 0.39]	0.06 [0 – 0.31]	0.1 [0 – 0.4]	0.02 [0 – 0.23]	0.06 [0 – 0.39]

COx, cerebral oximetry index with cerebral perfusion pressure; COx-a, cerebral oximetry index with arterial blood pressure; HC, healthy control volunteer group; IQR, interquartile range; r-value, Pearson correlation coefficient; rSO_2, regional cerebral oxygen saturation; SP, elective spinal surgery patient group; TBI, traumatic brain injury patient group.

File S7e: Anchored-Interval – Pearson Correlation Analysis of rSO_2 and COx/COx-a in All Populations using 5-Minute Temporal Resolution

Physiologic Variable	Value	Median [IQR]								
		5-Minute Interval	10-Minute Interval	15-Minute Interval	30-Minute Interval	1-Hour Interval	2-Hour Interval	6-Hour Interval	12-Hour Interval	1-Day Interval
**HC Population**										
rSO_2_L	*r*	NA	–	–	–	–	–	–	–	–
	p	NA	–	–	–	–	–	–	–	–
rSO_2_R	*r*	NA	–	–	–	–	–	–	–	–
	p	NA	–	–	–	–	–	–	–	–
COx_L	*r*	–	–	–	–	–	–	–	–	–
	p	–	–	–	–	–	–	–	–	–
COx_R	*r*	–	–	–	–	–	–	–	–	–
	p	–	–	–	–	–	–	–	–	–
COx-a_L	*r*	NA	–	–	–	–	–	–	–	–
	p	NA	–	–	–	–	–	–	–	–
COx-a_R	*r*	NA	–	–	–	–	–	–	–	–
	p	NA	–	–	–	–	–	–	–	–
**SP Population**										
rSO_2_L	*r*	0.76 [0.52 – 0.9]	0.65 [0.39 – 0.85]	0.47 [0.21 – 0.85]	0.6 [0.12 – 0.81]	-0.26 [-0.35 – 0.16]	–	–	–	–
	p	0.03 [0.01 – 0.16]	0.11 [0.02 – 0.33]	0.08 [0.02 – 0.41]	0.08 [0.01 – 0.36]	0.1 [0.06 – 0.2]	–	–	–	–
rSO_2_R	*r*	0.74 [0.43 – 0.89]	0.69 [0.39 – 0.84]	0.68 [0.1 – 0.85]	0.57 [-0.18 – 0.74]	0.17 [0.04 – 0.41]	–	–	–	–
	p	0.06 [0 – 0.2]	0.06 [0.01 – 0.21]	0.08 [0.01 – 0.33]	0.09 [0.01 – 0.24]	0.52 [0.26 – 0.61]	–	–	–	–
COx_L	*r*	–	–	–	–	–	–	–	–	–
	p	–	–	–	–	–	–	–	–	–
COx_R	*r*	–	–	–	–	–	–	–	–	–
	p	–	–	–	–	–	–	–	–	–
COx-a_L	*r*	0.17 [-0.18 – 0.38]	0.29 [-0.16 – 0.52]	0.17 [-0.54 – 0.35]	0.12 [-0.28 – 0.31]	0.14 [0.13 – 0.37]	–	–	–	–
	p	0.53 [0.14 – 0.7]	0.35 [0.1 – 0.65]	0.31 [0.07 – 0.61]	0.46 [0.13 – 0.75]	0.6 [0.31 – 0.63]	–	–	–	–
COx-a_R	*r*	0.1 [-0.1 – 0.36]	0.24 [-0.09 – 0.47]	0.14 [-0.29 – 0.31]	-0.02 [-0.22 – 0.28]	0 [-0.09 – 0.26]	–	–	–	–
	p	0.69 [0.32 – 0.84]	0.46 [0.2 – 0.74]	0.43 [0.17 – 0.62]	0.59 [0.28 – 0.75]	0.51 [0.29 – 0.75]	–	–	–	–
**TBI Population**										
rSO_2_L	*r*	**0.86 [0.71 – 0.93]**	**0.79 [0.54 – 0.9]**	**0.76 [0.52 – 0.87]**	**0.67 [0.47 – 0.84]**	**0.55 [0.29 – 0.73]**	0.38 [0.08 – 0.66]	0.23 [0.02 – 0.52]	0.1 [-0.03 – 0.36]	0.11 [-0.05 – 0.43]
	p	**0 [0 – 0]**	**0 [0 – 0]**	**0 [0 – 0]**	**0 [0 – 0]**	**0 [0 – 0.01]**	0 [0 – 0.23]	0 [0 – 0.07]	0 [0 – 0.22]	0 [0 – 0.05]
rSO_2_R	*r*	**0.87 [0.75 – 0.93]**	**0.82 [0.66 – 0.9]**	**0.79 [0.59 – 0.89]**	**0.71 [0.49 – 0.83]**	**0.55 [0.34 – 0.74]**	0.48 [0.1 – 0.64]	0.2 [-0.06 – 0.5]	0.11 [-0.05 – 0.48]	0.21 [-0.02 – 0.43]
	p	**0 [0 – 0]**	**0 [0 – 0]**	**0 [0 – 0]**	**0 [0 – 0]**	**0 [0 – 0.01]**	**0 [0 – 0.05]**	0 [0 – 0.09]	0 [0 – 0.24]	0 [0 – 0.02]
COx_L	*r*	0.31 [0.18 – 0.39]	0.2 [0.08 – 0.3]	0.16 [0.05 – 0.27]	0.11 [0.02 – 0.21]	0.08 [-0.03 – 0.17]	0.04 [-0.04 – 0.12]	0.03 [-0.06 – 0.11]	0.01 [-0.06 – 0.09]	0 [-0.05 – 0.11]
	p	0 [0 – 0.15]	0.02 [0 – 0.4]	0.04 [0 – 0.42]	0.21 [0.01 – 0.57]	0.25 [0.03 – 0.61]	0.36 [0.07 – 0.68]	0.27 [0.05 – 0.56]	0.3 [0.08 – 0.68]	0.2 [0.02 – 0.65]
COx_R	*r*	0.29 [0.18 – 0.37]	0.2 [0.09 – 0.28]	0.14 [0.04 – 0.21]	0.1 [-0.01 – 0.18]	0.05 [-0.05 – 0.11]	0.01 [-0.08 – 0.11]	0.03 [-0.07 – 0.11]	-0.01 [-0.06 – 0.12]	0.07 [0 – 0.1]
	p	0 [0 – 0.27]	0.06 [0 – 0.35]	0.07 [0 – 0.74]	0.25 [0.03 – 0.55]	0.26 [0.11 – 0.59]	0.39 [0.11 – 0.66]	0.28 [0.08 – 0.59]	0.24 [0.03 – 0.63]	0.16 [0.06 – 0.33]
COx-a_L	*r*	0.26 [0.19 – 0.36]	0.19 [0.07 – 0.29]	0.16 [0.08 – 0.24]	0.1 [0.01 – 0.19]	0.06 [-0.01 – 0.16]	0.02 [-0.04 – 0.12]	0.01 [-0.04 – 0.07]	0.01 [-0.06 – 0.07]	0.03 [-0.04 – 0.1]
	p	0 [0 – 0.1]	0.02 [0 – 0.37]	0.08 [0 – 0.28]	0.18 [0.01 – 0.6]	0.28 [0.06 – 0.56]	0.34 [0.13 – 0.63]	0.37 [0.17 – 0.69]	0.4 [0.13 – 0.68]	0.2 [0.04 – 0.56]
COx-a_R	*r*	0.28 [0.21 – 0.36]	0.21 [0.14 – 0.27]	0.14 [0.08 – 0.22]	0.09 [0.04 – 0.17]	0.04 [-0.01 – 0.12]	0.02 [-0.04 – 0.09]	-0.01 [-0.07 – 0.07]	0 [-0.06 – 0.06]	0.06 [0.03 – 0.08]
	p	0 [0 – 0.06]	0.02 [0 – 0.26]	0.08 [0 – 0.58]	0.33 [0.04 – 0.68]	0.42 [0.17 – 0.72]	0.47 [0.11 – 0.8]	0.36 [0.06 – 0.59]	0.42 [0.11 – 0.69]	0.27 [0.04 – 0.51]

COx, cerebral oximetry index with cerebral perfusion pressure; COx-a, cerebral oximetry index with arterial blood pressure; HC, healthy control volunteer group; IQR, interquartile range; r-value, Pearson correlation coefficient; rSO_2, regional cerebral oxygen saturation; SP, elective spinal surgery patient group; TBI, traumatic brain injury patient group.

File S7f: Windowed-Point – Pearson Correlation Analysis of rSO_2 and COx/COx-a in All Populations using 10-Second Temporal Resolution

Physiologic Variable	Value	Median [IQR]								
		**5-Minute Interval**	**10-Minute Interval**	**15-Minute Interval**	**30-Minute Interval**	**1-Hour Interval**	**2-Hour Interval**	**6-Hour Interval**	**12-Hour Interval**	**1-Day Interval**
**HC Population**										
rSO_2_L	*r*	**0.78 [0.66 – 0.87]**	**0.77 [0.66 – 0.86]**	**0.75 [0.65 – 0.85]**	0.64 [0.49 – 0.79]	–	–	–	–	–
	p	**0 [0 – 0]**	**0 [0 – 0]**	**0 [0 – 0]**	0 [0 – 0.08]	–	–	–	–	–
rSO_2_R	*r*	**0.79 [0.6 – 0.86]**	**0.77 [0.63 – 0.85]**	**0.74 [0.61 – 0.85]**	0.57 [0.4 – 0.68]	–	–	–	–	–
	p	**0 [0 – 0]**	**0 [0 – 0]**	**0 [0 – 0]**	0 [0 – 0.23]	–	–	–	–	–
COx_L	*r*	–	–	–	–	–	–	–	–	–
	p	–	–	–	–	–	–	–	–	–
COx_R	*r*	–	–	–	–	–	–	–	–	–
	p	–	–	–	–	–	–	–	–	–
COx-a_L	*r*	**0.97 [0.95 – 0.98]**	**0.97 [0.95 – 0.98]**	**0.96 [0.94 – 0.98]**	**0.94 [0.84 – 0.96]**	–	–	–	–	–
	p	**0 [0 – 0]**	**0 [0 – 0]**	**0 [0 – 0]**	**0 [0 – 0]**	–	–	–	–	–
COx-a_R	*r*	**0.96 [0.95 – 0.97]**	**0.97 [0.95 – 0.98]**	**0.96 [0.94 – 0.98]**	**0.93 [0.84 – 0.96]**	–	–	–	–	–
	p	**0 [0 – 0]**	**0 [0 – 0]**	**0 [0 – 0]**	**0 [0 – 0.01]**	–	–	–	–	–
**SP Population**										
rSO_2_L	*r*	**0.96 [0.9 – 0.98]**	**0.96 [0.85 – 0.99]**	**0.95 [0.88 – 0.99]**	**0.94 [0.63 – 0.99]**	**0.96 [0.91 – 0.99]**	**0.96 [0.89 – 0.99]**	0.33 [0.23 – 0.43]	–	–
	p	**0 [0 – 0]**	**0 [0 – 0]**	**0 [0 – 0]**	**0 [0 – 0]**	**0 [0 – 0]**	**0 [0 – 0]**	0.01 [0 – 0.01]	–	–
rSO_2_R	*r*	**0.95 [0.74 – 0.99]**	**0.95 [0.74 – 0.99]**	**0.96 [0.72 – 0.99]**	**0.96 [0.89 – 0.99]**	**0.97 [0.87 – 0.99]**	**0.97 [0.92 – 0.98]**	0.51 [0.27 – 0.74]	–	–
	p	**0 [0 – 0]**	**0 [0 – 0]**	**0 [0 – 0]**	**0 [0 – 0]**	**0 [0 – 0]**	**0 [0 – 0]**	0.32 [0.16 – 0.47]	–	–
COx_L	*r*	–	–	–	–	–	–	–	–	–
	p	–	–	–	–	–	–	–	–	–
COx_R	*r*	–	–	–	–	–	–	–	–	–
	p	–	–	–	–	–	–	–	–	–
COx-a_L	*r*	**0.97 [0.95 – 0.98]**	**0.97 [0.97 – 0.99]**	**0.98 [0.97 – 0.99]**	**0.98 [0.97 – 0.99]**	**0.98 [0.97 – 0.99]**	**0.98 [0.97 – 0.99]**	**0.98 [0.98 – 0.98]**	–	–
	p	**0 [0 – 0]**	**0 [0 – 0]**	**0 [0 – 0]**	**0 [0 – 0]**	**0 [0 – 0]**	**0 [0 – 0]**	**0 [0 – 0]**	–	–
COx-a_R	*r*	**0.97 [0.96 – 0.98]**	**0.98 [0.97 – 0.98]**	**0.98 [0.97 – 0.98]**	**0.98 [0.97 – 0.99]**	**0.98 [0.98 – 0.99]**	**0.98 [0.97 – 0.99]**	**0.96 [0.95 – 0.97]**	–	–
	p	**0 [0 – 0]**	**0 [0 – 0]**	**0 [0 – 0]**	**0 [0 – 0]**	**0 [0 – 0]**	**0 [0 – 0]**	**0 [0 – 0]**	–	–
**TBI Population**										
rSO_2_L	*r*	**0.96 [0.87 – 0.98]**	**0.93 [0.32 – 0.98]**	0.26 [0 – 0.98]	0.02 [0 – 0.98]	0.04 [0 – 0.99]	**0.73 [0 – 0.99]**	**0.97 [0.02 – 0.99]**	**0.92 [0.86 – 0.99]**	**0.92 [0.87 – 0.94]**
	p	**0 [0 – 0]**	**0 [0 – 0]**	0 [0 – 0.24]	0 [0 – 0.19]	0 [0 – 0.34]	**0 [0 – 0.31]**	**0 [0 – 0.02]**	**0 [0 – 0]**	**0 [0 – 0]**
rSO_2_R	*r*	**0.97 [0.92 – 0.98]**	**0.89 [0.32 – 0.97]**	0.29 [0.01 – 0.98]	0.02 [0 – 0.96]	0.03 [0 – 0.98]	0.02 [0 – 0.99]	**0.99 [0.96 – 0.99]**	**0.98 [0.96 – 0.99]**	**0.99 [0.98 – 0.99]**
	p	**0 [0 – 0]**	**0 [0 – 0]**	**0 [0 – 0.03]**	0 [0 – 0.51]	0 [0 – 0.37]	0 [0 – 0.4]	**0 [0 – 0]**	**0 [0 – 0]**	**0 [0 – 0]**
COx_L	*r*	**0.96 [0.94 – 0.96]**	**0.97 [0.96 – 0.97]**	**0.97 [0.97 – 0.98]**	**0.98 [0.97 – 0.98]**	**0.98 [0.97 – 0.98]**	**0.98 [0.97 – 0.98]**	**0.98 [0.97 – 0.98]**	**0.98 [0.97 – 0.98]**	**0.98 [0.98 – 0.98]**
	p	**0 [0 – 0]**	**0 [0 – 0]**	**0 [0 – 0]**	**0 [0 – 0]**	**0 [0 – 0]**	**0 [0 – 0]**	**0 [0 – 0]**	**0 [0 – 0]**	**0 [0 – 0]**
COx_R	*r*	**0.95 [0.94 – 0.96]**	**0.97 [0.96 – 0.98]**	**0.97 [0.97 – 0.98]**	**0.98 [0.97 – 0.98]**	**0.98 [0.97 – 0.98]**	**0.98 [0.98 – 0.98]**	**0.98 [0.97 – 0.98]**	**0.98 [0.97 – 0.98]**	**0.98 [0.97 – 0.98]**
	p	**0 [0 – 0]**	**0 [0 – 0]**	**0 [0 – 0]**	**0 [0 – 0]**	**0 [0 – 0]**	**0 [0 – 0]**	**0 [0 – 0]**	**0 [0 – 0]**	**0 [0 – 0]**
COx-a_L	*r*	**0.96 [0.94 – 0.96]**	**0.97 [0.96 – 0.97]**	**0.97 [0.97 – 0.98]**	**0.97 [0.97 – 0.98]**	**0.98 [0.97 – 0.98]**	**0.98 [0.97 – 0.98]**	**0.98 [0.97 – 0.98]**	**0.98 [0.97 – 0.98]**	**0.98 [0.97 – 0.98]**
	p	**0 [0 – 0]**	**0 [0 – 0]**	**0 [0 – 0]**	**0 [0 – 0]**	**0 [0 – 0]**	**0 [0 – 0]**	**0 [0 – 0]**	**0 [0 – 0]**	**0 [0 – 0]**
COx-a_R	*r*	**0.95 [0.94 – 0.96]**	**0.97 [0.96 – 0.97]**	**0.97 [0.96 – 0.97]**	**0.97 [0.97 – 0.98]**	**0.98 [0.97 – 0.98]**	**0.98 [0.97 – 0.98]**	**0.98 [0.97 – 0.98]**	**0.98 [0.97 – 0.98]**	**0.97 [0.97 – 0.98]**
	p	**0 [0 – 0]**	**0 [0 – 0]**	**0 [0 – 0]**	**0 [0 – 0]**	**0 [0 – 0]**	**0 [0 – 0]**	**0 [0 – 0]**	**0 [0 – 0]**	**0 [0 – 0]**

COx, cerebral oximetry index with cerebral perfusion pressure; COx-a, cerebral oximetry index with arterial blood pressure; HC, healthy control volunteer group; IQR, interquartile range; r-value, Pearson correlation coefficient; rSO_2, regional cerebral oxygen saturation; SP, elective spinal surgery patient group; TBI, traumatic brain injury patient group.

File S7g: Windowed-Point – Pearson Correlation Analysis of rSO_2 and COx/COx-a in All Populations using 1-Minute Temporal Resolution

Physiologic Variable	Value	Median [IQR]								
		5-Minute Interval	10-Minute Interval	15-Minute Interval	30-Minute Interval	1-Hour Interval	2-Hour Interval	6-Hour Interval	12-Hour Interval	1-Day Interval
**HC Population**										
rSO_2_L	*r*	0.46 [0.3 – 0.62]	0.47 [0.22 – 0.59]	0.42 [0.13 – 0.6]	0.28 [-0.21 – 0.67]	–	–	–	–	–
	p	0.01 [0 – 0.15]	0.04 [0 – 0.27]	0.09 [0.02 – 0.48]	0.34 [0.18 – 0.59]	–	–	–	–	–
rSO_2_R	*r*	0.46 [0.2 – 0.65]	0.46 [0.19 – 0.65]	0.39 [0.18 – 0.6]	0.27 [-0.34 – 0.58]	–	–	–	–	–
	p	0.02 [0 – 0.3]	0.04 [0 – 0.34]	0.14 [0.02 – 0.51]	0.43 [0.2 – 0.66]	–	–	–	–	–
COx_L	*r*	–	–	–	–	–	–	–	–	–
	p	–	–	–	–	–	–	–	–	–
COx_R	*r*	–	–	–	–	–	–	–	–	–
	p	–	–	–	–	–	–	–	–	–
COx-a_L	*r*	**0.61 [0.45 – 0.76]**	**0.65 [0.48 – 0.78]**	**0.69 [0.49 – 0.79]**	0.55 [0.26 – 0.68]	–	–	–	–	–
	p	**0 [0 – 0.03]**	**0 [0 – 0.06]**	**0.01 [0 – 0.08]**	0.31 [0.07 – 0.7]	–	–	–	–	–
COx-a_R	*r*	**0.62 [0.51 – 0.75]**	**0.65 [0.52 – 0.73]**	**0.67 [0.49 – 0.78]**	0.65 [0.07 – 0.75]	–	–	–	–	–
	p	**0 [0 – 0.02]**	**0 [0 – 0.03]**	**0.01 [0 – 0.11]**	0.21 [0.05 – 0.53]	–	–	–	–	–
**SP Population**										
rSO_2_L	*r*	**0.91 [0.72 – 0.96]**	**0.91 [0.62 – 0.95]**	**0.92 [0.69 – 0.95]**	**0.94 [0.77 – 0.97]**	**0.92 [0.82 – 0.97]**	**0.93 [0.79 – 0.97]**	**0.78 [0.73 – 0.84]**	–	–
	p	**0 [0 – 0]**	**0 [0 – 0]**	**0 [0 – 0]**	**0 [0 – 0]**	**0 [0 – 0]**	**0 [0 – 0]**	**0 [0 – 0]**	–	–
rSO_2_R	*r*	**0.92 [0.83 – 0.97]**	**0.9 [0.85 – 0.97]**	**0.91 [0.81 – 0.97]**	**0.92 [0.83 – 0.97]**	**0.92 [0.89 – 0.97]**	**0.91 [0.85 – 0.97]**	**0.66 [0.55 – 0.77]**	–	–
	p	**0 [0 – 0]**	**0 [0 – 0]**	**0 [0 – 0]**	**0 [0 – 0]**	**0 [0 – 0]**	**0 [0 – 0]**	**0.01 [0 – 0.01]**	–	–
COx_L	*r*	–	–	–	–	–	–	–	–	–
	p	–	–	–	–	–	–	–	–	–
COx_R	*r*	–	–	–	–	–	–	–	–	–
	p	–	–	–	–	–	–	–	–	–
COx-a_L	*r*	**0.71 [0.63 – 0.76]**	**0.73 [0.65 – 0.77]**	**0.75 [0.72 – 0.8]**	**0.8 [0.77 – 0.81]**	**0.8 [0.77 – 0.83]**	**0.83 [0.75 – 0.86]**	**0.84 [0.84 – 0.84]**	–	–
	p	**0 [0 – 0]**	**0 [0 – 0]**	**0 [0 – 0]**	**0 [0 – 0]**	**0 [0 – 0]**	**0 [0 – 0]**	**0 [0 – 0]**	–	–
COx-a_R	*r*	**0.68 [0.6 – 0.74]**	**0.73 [0.65 – 0.78]**	**0.75 [0.67 – 0.79]**	**0.78 [0.73 – 0.82]**	**0.81 [0.78 – 0.83]**	**0.83 [0.77 – 0.86]**	**0.77 [0.74 – 0.81]**	–	–
	p	**0 [0 – 0]**	**0 [0 – 0]**	**0 [0 – 0]**	**0 [0 – 0]**	**0 [0 – 0]**	**0 [0 – 0]**	**0.01 [0 – 0.01]**	–	–
**TBI Population**										
rSO_2_L	*r*	**0.95 [0.87 – 0.98]**	**0.94 [0.83 – 0.97]**	**0.93 [0.84 – 0.97]**	**0.94 [0.87 – 0.97]**	**0.94 [0.61 – 0.98]**	**0.95 [0.02 – 0.98]**	**0.95 [0.03 – 0.98]**	**0.94 [0.26 – 0.98]**	**0.96 [0.9 – 0.98]**
	p	**0 [0 – 0]**	**0 [0 – 0]**	**0 [0 – 0]**	**0 [0 – 0]**	**0 [0 – 0]**	**0 [0 – 0]**	**0 [0 – 0.01]**	**0 [0 – 0]**	**0 [0 – 0]**
rSO_2_R	*r*	**0.95 [0.89 – 0.98]**	**0.93 [0.85 – 0.96]**	**0.93 [0.85 – 0.96]**	**0.94 [0.87 – 0.97]**	**0.94 [0.25 – 0.98]**	**0.95 [0.05 – 0.97]**	**0.96 [0.04 – 0.98]**	**0.96 [0.67 – 0.98]**	**0.95 [0.8 – 0.98]**
	p	**0 [0 – 0]**	**0 [0 – 0]**	**0 [0 – 0]**	**0 [0 – 0]**	**0 [0 – 0]**	**0 [0 – 0]**	**0 [0 – 0]**	**0 [0 – 0]**	**0 [0 – 0]**
COx_L	*r*	**0.7 [0.68 – 0.74]**	**0.71 [0.66 – 0.76]**	**0.74 [0.7 – 0.78]**	**0.79 [0.76 – 0.82]**	**0.82 [0.8 – 0.84]**	**0.84 [0.82 – 0.86]**	**0.85 [0.84 – 0.87]**	**0.85 [0.84 – 0.87]**	**0.86 [0.85 – 0.87]**
	p	**0 [0 – 0]**	**0 [0 – 0]**	**0 [0 – 0]**	**0 [0 – 0]**	**0 [0 – 0]**	**0 [0 – 0]**	**0 [0 – 0]**	**0 [0 – 0]**	**0 [0 – 0]**
COx_R	*r*	**0.7 [0.68 – 0.77]**	**0.69 [0.65 – 0.76]**	**0.74 [0.68 – 0.79]**	**0.78 [0.76 – 0.81]**	**0.82 [0.8 – 0.83]**	**0.84 [0.82 – 0.85]**	**0.85 [0.84 – 0.86]**	**0.86 [0.84 – 0.87]**	**0.86 [0.84 – 0.88]**
	p	**0 [0 – 0]**	**0 [0 – 0]**	**0 [0 – 0]**	**0 [0 – 0]**	**0 [0 – 0]**	**0 [0 – 0]**	**0 [0 – 0]**	**0 [0 – 0]**	**0 [0 – 0]**
COx-a_L	*r*	**0.7 [0.67 – 0.74]**	**0.7 [0.65 – 0.75]**	**0.74 [0.69 – 0.78]**	**0.78 [0.76 – 0.81]**	**0.81 [0.8 – 0.84]**	**0.83 [0.81 – 0.85]**	**0.85 [0.84 – 0.86]**	**0.85 [0.84 – 0.86]**	**0.86 [0.84 – 0.87]**
	p	**0 [0 – 0]**	**0 [0 – 0]**	**0 [0 – 0]**	**0 [0 – 0]**	**0 [0 – 0]**	**0 [0 – 0]**	**0 [0 – 0]**	**0 [0 – 0]**	**0 [0 – 0]**
COx-a_R	*r*	**0.7 [0.67 – 0.73]**	**0.69 [0.63 – 0.74]**	**0.72 [0.68 – 0.76]**	**0.79 [0.76 – 0.8]**	**0.82 [0.8 – 0.83]**	**0.83 [0.82 – 0.85]**	**0.85 [0.83 – 0.86]**	**0.85 [0.84 – 0.86]**	**0.85 [0.84 – 0.86]**
	p	**0 [0 – 0]**	**0 [0 – 0]**	**0 [0 – 0]**	**0 [0 – 0]**	**0 [0 – 0]**	**0 [0 – 0]**	**0 [0 – 0]**	**0 [0 – 0]**	**0 [0 – 0]**

COx, cerebral oximetry index with cerebral perfusion pressure; COx-a, cerebral oximetry index with arterial blood pressure; HC, healthy control volunteer group; IQR, interquartile range; r-value, Pearson correlation coefficient; rSO_2, regional cerebral oxygen saturation; SP, elective spinal surgery patient group; TBI, traumatic brain injury patient group.

File S7h: Windowed-Point – Pearson Correlation Analysis of rSO$_2$ and COx/COx-a in All Populations using 5-Minute Temporal Resolution

Physiologic Variable	Value	Median [IQR]								
		**5-Minute Interval**	**10-Minute Interval**	**15-Minute Interval**	**30-Minute Interval**	**1-Hour Interval**	**2-Hour Interval**	**6-Hour Interval**	**12-Hour Interval**	**1-Day Interval**
**HC Population**										
rSO$_2$_L	*r*	NA	0.09 [-0.27 – 0.59]	-0.16 [-0.55 – 0.61]	-0.26 [-0.26 – -0.26]	–	–	–	–	–
	p	NA	0.56 [0.28 – 0.81]	0.55 [0.14 – 0.73]	0.74 [0.74 – 0.74]	–	–	–	–	–
rSO$_2$_R	*r*	NA	0.31 [-0.31 – 0.68]	-0.04 [-0.55 – 0.44]	-0.76 [-0.76 – -0.76]	–	–	–	–	–
	p	NA	0.53 [0.27 – 0.73]	0.6 [0.34 – 0.79]	0.24 [0.24 – 0.24]	–	–	–	–	–
COx_L	*r*	–	–	–	–	–	–	–	–	–
	p	–	–	–	–	–	–	–	–	–
COx_R	*r*	–	–	–	–	–	–	–	–	–
	p	–	–	–	–	–	–	–	–	–
COx-a_L	*r*	NA	-0.17 [-0.55 – 0.31]	0.23 [-0.44 – 0.7]	-0.23 [-0.23 – -0.23]	–	–	–	–	–
	p	NA	0.59 [0.32 – 0.79]	0.51 [0.3 – 0.68]	0.77 [0.77 – 0.77]	–	–	–	–	–
COx-a_R	*r*	NA	-0.06 [-0.57 – 0.44]	-0.24 [-0.61 – 0.26]	0.66 [0.66 – 0.66]	–	–	–	–	–
	p	NA	0.54 [0.35 – 0.81]	0.62 [0.38 – 0.79]	0.34 [0.34 – 0.34]	–	–	–	–	–
**SP Population**										
rSO$_2$_L	*r*	NA	**0.77 [0.69 – 0.89]**	**0.76 [0.6 – 0.86]**	**0.72 [0.5 – 0.87]**	**0.74 [0.57 – 0.89]**	**0.72 [0.56 – 0.86]**	0.57 [0.49 – 0.65]	–	–
	p	NA	**0 [0 – 0]**	**0 [0 – 0]**	**0 [0 – 0]**	**0 [0 – 0]**	**0 [0 – 0.04]**	0.11 [0.09 – 0.13]	–	–
rSO$_2$_R	*r*	NA	**0.82 [0.75 – 0.91]**	**0.75 [0.5 – 0.9]**	**0.66 [0.56 – 0.87]**	**0.7 [0.5 – 0.86]**	**0.77 [0.61 – 0.88]**	-0.1 [-0.25 – 0.05]	–	–
	p	NA	**0 [0 – 0]**	**0 [0 – 0]**	**0 [0 – 0]**	**0 [0 – 0.01]**	**0.03 [0 – 0.06]**	0.46 [0.42 – 0.5]	–	–
COx_L	*r*	–	–	–	–	–	–	–	–	–
	p	–	–	–	–	–	–	–	–	–
COx_R	*r*	–	–	–	–	–	–	–	–	–
	p	–	–	–	–	–	–	–	–	–
COx-a_L	*r*	NA	0.23 [0.09 – 0.38]	0.11 [0 – 0.25]	0.04 [-0.08 – 0.24]	-0.05 [-0.17 – 0.1]	0.03 [-0.09 – 0.25]	0.14 [-0.09 – 0.36]	–	–
	p	NA	0.21 [0.06 – 0.56]	0.43 [0.16 – 0.69]	0.54 [0.15 – 0.76]	0.45 [0.33 – 0.8]	0.57 [0.18 – 0.8]	0.26 [0.15 – 0.38]	–	–
COx-a_R	*r*	NA	0.18 [0.05 – 0.32]	0.11 [-0.02 – 0.23]	0.06 [-0.03 – 0.18]	0.04 [-0.14 – 0.09]	0.03 [-0.22 – 0.18]	0.11 [-0.17 – 0.38]	–	–
	p	NA	0.42 [0.12 – 0.68]	0.53 [0.27 – 0.68]	0.37 [0.2 – 0.74]	0.55 [0.38 – 0.84]	0.54 [0.29 – 0.72]	0.17 [0.1 – 0.24]	–	–
**TBI Population**										
rSO$_2$_L	*r*	NA	**0.94 [0.86 – 0.97]**	**0.92 [0.82 – 0.96]**	**0.85 [0.77 – 0.93]**	**0.85 [0.72 – 0.91]**	**0.88 [0.72 – 0.93]**	**0.91 [0.75 – 0.95]**	**0.89 [0.7 – 0.95]**	**0.92 [0.76 – 0.96]**
	p	NA	**0 [0 – 0]**	**0 [0 – 0]**	**0 [0 – 0]**	**0 [0 – 0]**	**0 [0 – 0]**	**0 [0 – 0]**	**0 [0 – 0]**	**0 [0 – 0]**
rSO$_2$_R	*r*	NA	**0.94 [0.84 – 0.97]**	**0.92 [0.81 – 0.96]**	**0.86 [0.72 – 0.94]**	**0.85 [0.69 – 0.93]**	**0.88 [0.78 – 0.93]**	**0.91 [0.81 – 0.96]**	**0.92 [0.77 – 0.95]**	**0.92 [0.78 – 0.96]**
	p	NA	**0 [0 – 0]**	**0 [0 – 0]**	**0 [0 – 0]**	**0 [0 – 0]**	**0 [0 – 0]**	**0 [0 – 0]**	**0 [0 – 0]**	**0 [0 – 0]**
COx_L	*r*	NA	0.37 [0.31 – 0.44]	0.27 [0.21 – 0.32]	0.2 [0.13 – 0.26]	0.21 [0.15 – 0.27]	0.24 [0.17 – 0.33]	0.3 [0.21 – 0.37]	0.33 [0.24 – 0.39]	0.34 [0.29 – 0.44]
	p	NA	**0 [0 – 0]**	**0 [0 – 0]**	**0 [0 – 0.01]**	**0 [0 – 0.01]**	**0 [0 – 0]**	**0 [0 – 0]**	**0 [0 – 0]**	**0 [0 – 0]**
COx_R	*r*	NA	0.37 [0.32 – 0.47]	0.28 [0.21 – 0.35]	0.2 [0.13 – 0.28]	0.21 [0.16 – 0.32]	0.26 [0.17 – 0.37]	0.31 [0.23 – 0.42]	0.33 [0.24 – 0.44]	0.36 [0.28 – 0.49]
	p	NA	**0 [0 – 0]**	**0 [0 – 0]**	**0 [0 – 0.02]**	**0 [0 – 0]**	**0 [0 – 0]**	**0 [0 – 0]**	**0 [0 – 0]**	**0 [0 – 0]**
COx-a_L	*r*	NA	0.35 [0.3 – 0.41]	0.25 [0.2 – 0.31]	0.19 [0.13 – 0.23]	0.21 [0.15 – 0.25]	0.22 [0.15 – 0.3]	0.27 [0.19 – 0.34]	0.3 [0.21 – 0.39]	0.33 [0.26 – 0.42]
	p	NA	**0 [0 – 0]**	**0 [0 – 0]**	**0 [0 – 0.05]**	**0 [0 – 0.01]**	**0 [0 – 0.01]**	**0 [0 – 0]**	**0 [0 – 0]**	**0 [0 – 0]**
COx-a_R	*r*	NA	0.36 [0.31 – 0.42]	0.26 [0.21 – 0.31]	0.19 [0.14 – 0.25]	0.2 [0.14 – 0.27]	0.24 [0.15 – 0.3]	0.27 [0.2 – 0.34]	0.31 [0.22 – 0.38]	0.32 [0.27 – 0.39]
	p	NA	**0 [0 – 0]**	**0 [0 – 0]**	**0 [0 – 0.02]**	**0 [0 – 0]**	**0 [0 – 0]**	**0 [0 – 0]**	**0 [0 – 0]**	**0 [0 – 0]**

COx, cerebral oximetry index with cerebral perfusion pressure; COx-a, cerebral oximetry index with arterial blood pressure; HC, healthy control volunteer group; IQR, interquartile range; r-value, Pearson correlation coefficient; rSO$_2$, regional cerebral oxygen saturation; SP, elective spinal surgery patient group; TBI, traumatic brain injury patient group.

File S7i: Windowed-Interval – Pearson Correlation Analysis of rSO_2 and COx/COx-a in All Populations using 10-Second Temporal Resolution

Physiologic Variable	Value	Median [IQR]								
		**5-Minute Interval**	**10-Minute Interval**	**15-Minute Interval**	**30-Minute Interval**	**1-Hour Interval**	**2-Hour Interval**	**6-Hour Interval**	**12-Hour Interval**	**1-Day Interval**
**HC Population**										
rSO_2_L	*r*	0.21 [0.01 – 0.46]	0.06 [-0.08 – 0.31]	0.08 [-0.07 – 0.23]	–	–	–	–	–	–
	p	0 [0 – 0.18]	0.01 [0 – 0.44]	0.08 [0 – 0.41]	–	–	–	–	–	–
rSO_2_R	*r*	0.3 [0.06 – 0.48]	0.23 [-0.05 – 0.38]	0.07 [-0.06 – 0.32]	–	–	–	–	–	–
	p	0 [0 – 0.14]	0 [0 – 0.09]	0.04 [0 – 0.48]	–	–	–	–	–	–
COx_L	*r*	–	–	–	–	–	–	–	–	–
	p	–	–	–	–	–	–	–	–	–
COx_R	*r*	–	–	–	–	–	–	–	–	–
	p	–	–	–	–	–	–	–	–	–
COx-a_L	*r*	0.28 [0.05 – 0.55]	0.16 [-0.25 – 0.49]	0.06 [-0.1 – 0.27]	–	–	–	–	–	–
	p	0 [0 – 0.1]	**0 [0 – 0.04]**	0.03 [0 – 0.41]	–	–	–	–	–	–
COx-a_R	*r*	0.26 [0.02 – 0.47]	0.19 [-0.11 – 0.46]	0.11 [-0.03 – 0.28]	–	–	–	–	–	–
	p	0 [0 – 0.09]	0 [0 – 0.13]	0.13 [0 – 0.4]	–	–	–	–	–	–
**SP Population**										
rSO_2_L	*r*	**0.69 [0.13 – 0.85]**	**0.55 [0.26 – 0.77]**	**0.53 [0.32 – 0.77]**	0.29 [0.09 – 0.7]	0.01 [-0.26 – 0.33]	0.1 [0.08 – 0.3]	–	–	–
	p	**0 [0 – 0]**	**0 [0 – 0]**	**0 [0 – 0]**	**0 [0 – 0]**	**0 [0 – 0.01]**	**0 [0 – 0.01]**	–	–	–
rSO_2_R	*r*	**0.65 [0.22 – 0.89]**	**0.66 [0.4 – 0.85]**	**0.6 [0.31 – 0.84]**	0.26 [0 – 0.56]	-0.03 [-0.23 – 0.2]	-0.14 [-0.14 – 0.26]	–	–	–
	p	**0 [0 – 0]**	**0 [0 – 0]**	**0 [0 – 0]**	**0 [0 – 0]**	0 [0 – 0.1]	**0 [0 – 0]**	–	–	–
COx_L	*r*	–	–	–	–	–	–	–	–	–
	p	–	–	–	–	–	–	–	–	–
COx_R	*r*	–	–	–	–	–	–	–	–	–
	p	–	–	–	–	–	–	–	–	–
COx-a_L	*r*	0.33 [0.18 – 0.42]	0.18 [0 – 0.26]	0.1 [-0.04 – 0.19]	0.07 [-0.02 – 0.19]	0.03 [-0.11 – 0.1]	-0.05 [-0.11 – 0.16]	–	–	–
	p	**0 [0 – 0]**	**0 [0 – 0.01]**	**0 [0 – 0.01]**	0 [0 – 0.27]	0.01 [0 – 0.35]	**0 [0 – 0.04]**	–	–	–
COx-a_R	*r*	0.28 [0.15 – 0.34]	0.19 [0.1 – 0.25]	0.15 [0 – 0.19]	0 [-0.1 – 0.16]	0.08 [-0.05 – 0.21]	0.07 [-0.03 – 0.19]	–	–	–
	p	**0 [0 – 0]**	**0 [0 – 0]**	**0 [0 – 0.01]**	**0 [0 – 0.04]**	0 [0 – 0.07]	**0 [0 – 0.01]**	–	–	–
**TBI Population**										
rSO_2_L	*r*	0.01 [0 – 0.13]	0.04 [0 – 0.78]	0.37 [0 – 0.82]	**0.62 [0.12 – 0.83]**	**0.74 [0.54 – 0.84]**	**0.65 [0.45 – 0.82]**	0.48 [0.31 – 0.7]	0.39 [0.09 – 0.63]	0.21 [-0.02 – 0.5]
	p	0.01 [0 – 0.54]	0 [0 – 0.26]	0 [0 – 0.12]	**0 [0 – 0]**	**0 [0 – 0]**	**0 [0 – 0]**	**0 [0 – 0]**	**0 [0 – 0]**	**0 [0 – 0]**
rSO_2_R	*r*	0.01 [0 – 0.08]	0.1 [0.01 – 0.57]	0.08 [0 – 0.71]	**0.65 [0.18 – 0.87]**	**0.71 [0.39 – 0.86]**	**0.67 [0.43 – 0.79]**	**0.54 [0.34 – 0.67]**	0.46 [0.18 – 0.68]	0.28 [0.11 – 0.5]
	p	0.1 [0 – 0.62]	0 [0 – 0.15]	0 [0 – 0.12]	**0 [0 – 0]**	**0 [0 – 0]**	**0 [0 – 0]**	**0 [0 – 0]**	**0 [0 – 0]**	**0 [0 – 0]**
COx_L	*r*	0.24 [0.06 – 0.34]	0.2 [0.1 – 0.25]	0.15 [0.08 – 0.21]	0.12 [0.08 – 0.2]	0.11 [0.06 – 0.16]	0.08 [0.04 – 0.14]	0.06 [0 – 0.13]	0.08 [0.03 – 0.13]	0.05 [-0.02 – 0.14]
	p	**0 [0 – 0]**	**0 [0 – 0]**	**0 [0 – 0]**	**0 [0 – 0]**	**0 [0 – 0]**	**0 [0 – 0]**	**0 [0 – 0]**	**0 [0 – 0]**	**0 [0 – 0]**
COx_R	*r*	0.25 [0.07 – 0.35]	0.19 [0.08 – 0.26]	0.14 [0.08 – 0.21]	0.13 [0.1 – 0.19]	0.12 [0.06 – 0.18]	0.1 [0.06 – 0.16]	0.07 [0.03 – 0.18]	0.09 [0.02 – 0.17]	0.03 [-0.04 – 0.11]
	p	**0 [0 – 0]**	**0 [0 – 0]**	**0 [0 – 0]**	**0 [0 – 0]**	**0 [0 – 0]**	**0 [0 – 0]**	**0 [0 – 0]**	**0 [0 – 0]**	**0 [0 – 0]**
COx-a_L	*r*	0.19 [0.03 – 0.36]	0.19 [0.09 – 0.24]	0.15 [0.09 – 0.22]	0.13 [0.07 – 0.18]	0.12 [0.06 – 0.17]	0.08 [0.02 – 0.13]	0.07 [0 – 0.14]	0.06 [0 – 0.15]	0.06 [0.01 – 0.11]
	p	**0 [0 – 0]**	**0 [0 – 0]**	**0 [0 – 0]**	**0 [0 – 0]**	**0 [0 – 0]**	**0 [0 – 0]**	**0 [0 – 0]**	**0 [0 – 0]**	**0 [0 – 0]**
COx-a_R	*r*	0.24 [0.07 – 0.33]	0.19 [0.1 – 0.24]	0.13 [0.06 – 0.19]	0.12 [0.08 – 0.17]	0.09 [0.04 – 0.14]	0.09 [0.03 – 0.14]	0.08 [0.02 – 0.15]	0.07 [-0.01 – 0.15]	0.03 [-0.02 – 0.08]
	p	**0 [0 – 0]**	**0 [0 – 0]**	**0 [0 – 0]**	**0 [0 – 0]**	**0 [0 – 0]**	**0 [0 – 0]**	**0 [0 – 0]**	**0 [0 – 0]**	**0 [0 – 0.02]**

COx, cerebral oximetry index with cerebral perfusion pressure; COx-a, cerebral oximetry index with arterial blood pressure; HC, healthy control volunteer group; IQR, interquartile range; r-value, Pearson correlation coefficient; rSO_2, regional cerebral oxygen saturation; SP, elective spinal surgery patient group; TBI, traumatic brain injury patient group.

File S7j: Windowed-Interval – Pearson Correlation Analysis of rSO_2 and COx/COx-a in All Populations using 1-Minute Temporal Resolution

Physiologic Variable	Value	Median [IQR]								
		5-Minute Interval	10-Minute Interval	15-Minute Interval	30-Minute Interval	1-Hour Interval	2-Hour Interval	6-Hour Interval	12-Hour Interval	1-Day Interval
**HC Population**										
rSO_2_L	*r*	0.21 [0.03 – 0.47]	0.07 [-0.18 – 0.35]	0.16 [-0.13 – 0.4]	–	–	–	–	–	–
	p	0.24 [0.02 – 0.55]	0.21 [0.02 – 0.62]	0.35 [0.06 – 0.59]	–	–	–	–	–	–
rSO_2_R	*r*	0.3 [0.04 – 0.45]	0.13 [-0.22 – 0.39]	0.07 [-0.15 – 0.32]	–	–	–	–	–	–
	p	0.11 [0.02 – 0.45]	0.14 [0.03 – 0.43]	0.33 [0.07 – 0.72]	–	–	–	–	–	–
COx_L	*r*	–	–	–	–	–	–	–	–	–
	p	–	–	–	–	–	–	–	–	–
COx_R	*r*	–	–	–	–	–	–	–	–	–
	p	–	–	–	–	–	–	–	–	–
COx-a_L	*r*	0.25 [-0.05 – 0.42]	0.04 [-0.22 – 0.37]	0.18 [-0.14 – 0.44]	–	–	–	–	–	–
	p	0.15 [0.03 – 0.5]	0.17 [0.03 – 0.61]	0.2 [0.05 – 0.56]	–	–	–	–	–	–
COx-a_R	*r*	0.22 [0.02 – 0.42]	0.15 [-0.23 – 0.42]	0.15 [-0.08 – 0.49]	–	–	–	–	–	–
	p	0.21 [0.03 – 0.46]	0.21 [0.02 – 0.51]	0.31 [0.06 – 0.67]	–	–	–	–	–	–
**SP Population**										
rSO_2_L	*r*	**0.66 [0.54 – 0.84]**	**0.54 [0.33 – 0.76]**	**0.58 [0.25 – 0.75]**	0.27 [0.01 – 0.58]	0.07 [-0.32 – 0.32]	0.1 [0.07 – 0.2]	–	–	–
	p	**0 [0 – 0]**	**0 [0 – 0]**	**0 [0 – 0]**	0 [0 – 0.12]	0 [0 – 0.17]	0.14 [0.07 – 0.33]	–	–	–
rSO_2_R	*r*	**0.7 [0.56 – 0.81]**	**0.54 [0.4 – 0.81]**	**0.59 [0.31 – 0.76]**	0.31 [0.02 – 0.49]	-0.02 [-0.23 – 0.29]	0.16 [0.04 – 0.44]	–	–	–
	p	**0 [0 – 0]**	**0 [0 – 0]**	**0 [0 – 0]**	0 [0 – 0.12]	0 [0 – 0.61]	0.02 [0.01 – 0.08]	–	–	–
COx_L	*r*	–	–	–	–	–	–	–	–	–
	p	–	–	–	–	–	–	–	–	–
COx_R	*r*	–	–	–	–	–	–	–	–	–
	p	–	–	–	–	–	–	–	–	–
COx-a_L	*r*	0.19 [0.07 – 0.32]	0.08 [-0.03 – 0.24]	0.07 [0.01 – 0.17]	0.02 [-0.14 – 0.06]	-0.01 [-0.2 – 0.09]	-0.1 [-0.14 – -0.04]	–	–	–
	p	0.01 [0 – 0.42]	0.25 [0 – 0.47]	0.3 [0.01 – 0.6]	0.24 [0.06 – 0.46]	0.18 [0.03 – 0.79]	0.08 [0.04 – 0.43]	–	–	–
COx-a_R	*r*	0.24 [0.14 – 0.34]	0.09 [-0.01 – 0.27]	0.11 [0.03 – 0.19]	0.08 [-0.04 – 0.14]	0.08 [-0.11 – 0.19]	0.12 [0 – 0.18]	–	–	–
	p	0.01 [0 – 0.07]	0.02 [0 – 0.31]	0.17 [0.02 – 0.46]	0.18 [0.06 – 0.48]	0.13 [0.03 – 0.3]	0.04 [0.02 – 0.06]	–	–	–
**TBI Population**										
rSO_2_L	*r*	**0.88 [0.73 – 0.93]**	**0.61 [0.36 – 0.82]**	**0.61 [0.34 – 0.83]**	**0.58 [0.19 – 0.8]**	**0.6 [0.19 – 0.82]**	**0.59 [0.22 – 0.74]**	0.49 [0.28 – 0.72]	0.34 [0.09 – 0.63]	0.24 [0 – 0.49]
	p	**0 [0 – 0]**	**0 [0 – 0]**	**0 [0 – 0]**	**0 [0 – 0]**	**0 [0 – 0]**	**0 [0 – 0]**	**0 [0 – 0]**	**0 [0 – 0]**	**0 [0 – 0]**
rSO_2_R	*r*	**0.88 [0.72 – 0.96]**	**0.71 [0.39 – 0.86]**	**0.65 [0.13 – 0.82]**	0.25 [0.04 – 0.7]	**0.57 [0.25 – 0.8]**	**0.57 [0.23 – 0.74]**	**0.53 [0.27 – 0.67]**	0.49 [0.17 – 0.65]	0.29 [0.05 – 0.5]
	p	**0 [0 – 0]**	**0 [0 – 0]**	**0 [0 – 0]**	**0 [0 – 0]**	**0 [0 – 0]**	**0 [0 – 0]**	**0 [0 – 0]**	**0 [0 – 0]**	**0 [0 – 0]**
COx_L	*r*	0.27 [0.22 – 0.32]	0.1 [0.03 – 0.17]	0.1 [0.02 – 0.15]	0.1 [0.04 – 0.15]	0.11 [0.07 – 0.16]	0.13 [0.06 – 0.17]	0.08 [-0.01 – 0.15]	0.09 [0.04 – 0.14]	0.06 [-0.03 – 0.13]
	p	**0 [0 – 0]**	**0 [0 – 0.04]**	0 [0 – 0.15]	**0 [0 – 0.02]**	**0 [0 – 0]**	**0 [0 – 0]**	0 [0 – 0.14]	0 [0 – 0.09]	0 [0 – 0.08]
COx_R	*r*	0.25 [0.21 – 0.38]	0.09 [0.03 – 0.17]	0.1 [0.02 – 0.15]	0.11 [0.05 – 0.17]	0.13 [0.08 – 0.19]	0.12 [0.06 – 0.2]	0.08 [0.02 – 0.19]	0.1 [0.02 – 0.16]	0.05 [-0.04 – 0.12]
	p	**0 [0 – 0]**	0 [0 – 0.13]	0 [0 – 0.07]	**0 [0 – 0]**	**0 [0 – 0]**	0 [0 – 0.01]	**0 [0 – 0.03]**	**0 [0 – 0.02]**	0 [0 – 0.07]
COx-a_L	*r*	0.26 [0.21 – 0.31]	0.08 [0.02 – 0.16]	0.09 [0.02 – 0.15]	0.1 [0.04 – 0.14]	0.1 [0.05 – 0.16]	0.1 [0.04 – 0.15]	0.09 [0.01 – 0.14]	0.06 [0.01 – 0.15]	0.06 [0 – 0.12]
	p	**0 [0 – 0]**	0 [0 – 0.15]	0 [0 – 0.15]	**0 [0 – 0.04]**	**0 [0 – 0.01]**	**0 [0 – 0.02]**	0 [0 – 0.08]	0 [0 – 0.1]	0 [0 – 0.24]
COx-a_R	*r*	0.26 [0.21 – 0.33]	0.1 [0.03 – 0.16]	0.09 [0.04 – 0.14]	0.1 [0.04 – 0.14]	0.11 [0.05 – 0.15]	0.09 [0.03 – 0.19]	0.08 [0.02 – 0.15]	0.06 [0 – 0.13]	0.04 [0 – 0.08]
	p	**0 [0 – 0]**	0 [0 – 0.14]	**0 [0 – 0.04]**	**0 [0 – 0.03]**	**0 [0 – 0.01]**	**0 [0 – 0.01]**	**0 [0 – 0.03]**	0 [0 – 0.1]	0 [0 – 0.31]

COx, cerebral oximetry index with cerebral perfusion pressure; COx-a, cerebral oximetry index with arterial blood pressure; HC, healthy control volunteer group; IQR, interquartile range; r-value, Pearson correlation coefficient; rSO_2, regional cerebral oxygen saturation; SP, elective spinal surgery patient group; TBI, traumatic brain injury patient group.

File S7k: Windowed-Interval – Pearson Correlation Analysis of rSO_2 and COx/COx-a in All Populations using 5-Minute Temporal Resolution

Physiologic Variable	Value	Median [IQR]								
		**5-Minute Interval**	**10-Minute Interval**	**15-Minute Interval**	**30-Minute Interval**	**1-Hour Interval**	**2-Hour Interval**	**6-Hour Interval**	**12-Hour Interval**	**1-Day Interval**
**HC Population**										
rSO_2_L	*r*	NA	0.09 [-0.6 – 0.58]	0.08 [-0.66 – 0.83]	–	–	–	–	–	–
	p	NA	0.43 [0.15 – 0.73]	0.42 [0.19 – 0.61]	–	–	–	–	–	–
rSO_2_R	*r*	NA	0.24 [-0.6 – 0.76]	0 [-0.74 – 0.59]	–	–	–	–	–	–
	p	NA	0.32 [0.14 – 0.66]	0.47 [0.26 – 0.72]	–	–	–	–	–	–
COx_L	*r*	–	–	–	–	–	–	–	–	–
	p	–	–	–	–	–	–	–	–	–
COx_R	*r*	–	–	–	–	–	–	–	–	–
	p	–	–	–	–	–	–	–	–	–
COx-a_L	*r*	NA	-0.31 [-0.77 – 0.38]	-0.33 [-0.87 – 0.69]	–	–	–	–	–	–
	p	NA	0.47 [0.1 – 0.73]	0.29 [0.14 – 0.56]	–	–	–	–	–	–
COx-a_R	*r*	NA	-0.33 [-0.64 – 0.21]	-0.2 [-0.76 – 0.39]	–	–	–	–	–	–
	p	NA	0.42 [0.23 – 0.77]	0.49 [0.23 – 0.73]	–	–	–	–	–	–
**SP Population**										
rSO_2_L	*r*	NA	**0.75 [0.59 – 0.89]**	**0.57 [0.12 – 0.79]**	0.32 [0.14 – 0.53]	0.02 [-0.31 – 0.33]	0.11 [0.03 – 0.31]	–	–	–
	p	NA	**0 [0 – 0]**	**0 [0 – 0.04]**	0.08 [0 – 0.34]	0.18 [0.02 – 0.56]	0.47 [0.23 – 0.59]	–	–	–
rSO_2_R	*r*	NA	**0.75 [0.47 – 0.85]**	0.34 [0.12 – 0.8]	0.21 [0.02 – 0.46]	0.13 [-0.06 – 0.38]	-0.21 [-0.27 – 0.28]	–	–	–
	p	NA	**0 [0 – 0.01]**	0.08 [0 – 0.33]	0.14 [0.01 – 0.71]	0.39 [0.04 – 0.58]	0.02 [0.01 – 0.07]	–	–	–
COx_L	*r*	–	–	–	–	–	–	–	–	–
	p	–	–	–	–	–	–	–	–	–
COx_R	*r*	–	–	–	–	–	–	–	–	–
	p	–	–	–	–	–	–	–	–	–
COx-a_L	*r*	NA	0.13 [-0.05 – 0.28]	0.07 [-0.08 – 0.18]	-0.05 [-0.28 – 0.1]	-0.16 [-0.33 – 0.01]	-0.06 [-0.24 – -0.04]	–	–	–
	p	NA	0.28 [0.08 – 0.51]	0.4 [0.11 – 0.67]	0.38 [0.14 – 0.68]	0.24 [0.1 – 0.69]	0.66 [0.33 – 0.77]	–	–	–
COx-a_R	*r*	NA	0.17 [0.06 – 0.3]	0.03 [-0.11 – 0.15]	-0.07 [-0.16 – 0.05]	-0.19 [-0.34 – 0.04]	0.15 [0.01 – 0.17]	–	–	–
	p	NA	0.49 [0.2 – 0.66]	0.48 [0.19 – 0.73]	0.4 [0.16 – 0.71]	0.27 [0.12 – 0.46]	0.3 [0.23 – 0.32]	–	–	–
**TBI Population**										
rSO_2_L	*r*	NA	**0.92 [0.82 – 0.96]**	**0.86 [0.72 – 0.93]**	**0.68 [0.45 – 0.83]**	**0.5 [0.17 – 0.71]**	**0.55 [0.22 – 0.74]**	0.44 [0.2 – 0.66]	0.36 [0.1 – 0.62]	0.24 [0.02 – 0.51]
	p	NA	**0 [0 – 0]**	**0 [0 – 0]**	**0 [0 – 0]**	**0 [0 – 0.02]**	**0 [0 – 0]**	**0 [0 – 0]**	**0 [0 – 0]**	**0 [0 – 0.01]**
rSO_2_R	*r*	NA	**0.91 [0.79 – 0.96]**	**0.87 [0.71 – 0.92]**	**0.61 [0.25 – 0.83]**	**0.52 [0.16 – 0.76]**	0.47 [0.16 – 0.73]	0.47 [0.27 – 0.68]	0.4 [0.11 – 0.65]	0.32 [0.1 – 0.5]
	p	NA	**0 [0 – 0]**	**0 [0 – 0]**	**0 [0 – 0]**	**0 [0 – 0]**	**0 [0 – 0]**	**0 [0 – 0]**	**0 [0 – 0]**	**0 [0 – 0]**
COx_L	*r*	NA	0.27 [0.2 – 0.33]	0.14 [0.09 – 0.2]	0.07 [0.02 – 0.13]	0.07 [0 – 0.12]	0.1 [0.04 – 0.18]	0.09 [-0.01 – 0.17]	0.09 [0.01 – 0.16]	0.05 [-0.02 – 0.14]
	p	NA	**0 [0 – 0]**	0 [0 – 0.08]	0.06 [0 – 0.4]	0.07 [0 – 0.49]	0.01 [0 – 0.15]	0.03 [0 – 0.39]	0.02 [0 – 0.28]	0.02 [0 – 0.35]
COx_R	*r*	NA	0.26 [0.19 – 0.36]	0.15 [0.09 – 0.23]	0.06 [0.02 – 0.12]	0.09 [0.03 – 0.15]	0.09 [0.03 – 0.17]	0.09 [0.01 – 0.2]	0.08 [0.01 – 0.2]	0.06 [-0.02 – 0.14]
	p	NA	**0 [0 – 0]**	0 [0 – 0.15]	0.15 [0.01 – 0.47]	0.06 [0 – 0.34]	0.02 [0 – 0.14]	0.01 [0 – 0.4]	0.01 [0 – 0.22]	0.02 [0 – 0.45]
COx-a_L	*r*	NA	0.26 [0.2 – 0.31]	0.14 [0.08 – 0.19]	0.05 [0.01 – 0.11]	0.05 [0.01 – 0.11]	0.09 [0.02 – 0.18]	0.07 [0.01 – 0.17]	0.08 [0.02 – 0.17]	0.07 [-0.02 – 0.14]
	p	NA	**0 [0 – 0]**	0 [0 – 0.07]	0.12 [0 – 0.53]	0.2 [0.01 – 0.47]	0.01 [0 – 0.27]	0.05 [0 – 0.38]	0.02 [0 – 0.24]	0.08 [0 – 0.42]
COx-a_R	*r*	NA	0.26 [0.2 – 0.33]	0.13 [0.1 – 0.2]	0.06 [0.02 – 0.11]	0.06 [0 – 0.11]	0.08 [0.01 – 0.16]	0.09 [0.04 – 0.15]	0.07 [0 – 0.15]	0.04 [0 – 0.1]
	p	NA	**0 [0 – 0]**	0 [0 – 0.08]	0.1 [0.01 – 0.38]	0.2 [0.01 – 0.53]	0.04 [0 – 0.38]	0.03 [0 – 0.23]	0.05 [0 – 0.57]	0.16 [0 – 0.58]

COx, cerebral oximetry index with cerebral perfusion pressure; COx-a, cerebral oximetry index with arterial blood pressure; HC, healthy control volunteer group; IQR, interquartile range; r-value, Pearson correlation coefficient; rSO_2, regional cerebral oxygen saturation; SP, elective spinal surgery patient group; TBI, traumatic brain injury patient group.
