# Supplementary material for: Time-Series Autoregressive Models for Point and Interval Forecasting of Raw and Derived Commercial Near-Infrared Spectroscopy Measures: An Exploratory Cranial Trauma and Healthy Control Analysis
Source: Bioengineering (Basel). 2025 Jun 21;12(7):682. doi: 10.3390/bioengineering12070682 (PMC12292983; doi:10.3390/bioengineering12070682)
Supplement: Supplementary file 1 [file bioengineering-12-00682-s001.zip › File S8.pdf]

File S8 – Bland-Altman Agreement Analysis

File S8 – Table of Contents

File S8a: Anchored-Point – Bland-Altman Analysis of rSO_2 and COx/COx-a in All Populations using 10-Second Temporal Resolution 2

File S8b: Anchored-Interval – Bland-Altman Analysis of rSO_2 and COx/COx-a in All Populations using 10-Second Temporal Resolution 3

File S8c: Windowed-Point – Bland-Altman Analysis of rSO_2 and COx/COx-a in All Populations using 10-Second Temporal Resolution 6

File S8d: Windowed-Interval – Bland-Altman Analysis of rSO_2 and COx/COx-a in All Populations using 10-Second Temporal Resolution 9

File S8e: Anchored-Point – Bland-Altman Analysis of rSO_2 and COx/COx-a in All Populations using 1-Minute and 5-Minute Temporal Resolutions 12

File S8f: Anchored-Interval – Bland-Altman Analysis of rSO_2 and COx/COx-a in All Populations using 1-Minute Temporal Resolution 14

File S8g: Anchored-Interval – Bland-Altman Analysis of rSO_2 and COx/COx-a in All Populations using 5-Minute Temporal Resolution 17

File S8h: Windowed-Point – Bland-Altman Analysis of rSO_2 and COx/COx-a in All Populations using 1-Minute Temporal Resolution 20

File S8i: Windowed-Point – Bland-Altman Analysis of rSO_2 and COx/COx-a in All Populations using 5-Minute Temporal Resolution 23

File S8j: Windowed-Interval – Bland-Altman Analysis of rSO_2 and COx/COx-a in All Populations using 1-Minute Temporal Resolution 26

File S8k: Windowed-Interval – Bland-Altman Analysis of rSO_2 and COx/COx-a in All Populations using 5-Minute Temporal Resolution 29

File S8a: Anchored-Point – Bland-Altman Analysis of rSO_2 and COx/COx-a in All Populations using 10-Second Temporal Resolution

Physiologic Variable	Value	Median [IQR]		
		HC	SP	TBI
rSO_2_L	Bias	0.18 [-0.35 – 0.87]	1.49 [0.23 – 4.57]	-0.61 [-3.32 – 1.22]
	Lower LoA	-1.84 [-2.79 – -0.95]	-3.1 [-4.78 – -1.32]	-7.73 [-13.42 – -3.97]
	Upper LoA	2.07 [1.44 – 3.02]	7.86 [3.99 – 12.08]	6.55 [3.52 – 13.14]
	LoA Spread	3.9 [3.12 – 5.4]	12.42 [7.68 – 17.31]	15.6 [9.93 – 20.54]
	Relative Bias	4.15 [-9.19 – 21.81]	15.55 [2.75 – 33.97]	-3.42 [-19.64 – 9.97]
	Regression Slope	1.95 [1.8 – 2]	2 [2 – 2]	2 [2 – 2]
	Regression Intercept	-140 [-150 – -120]	-130 [-150 – -110]	-130 [-150 – -120]
rSO_2_R	Bias	0.26 [-0.33 – 0.94]	2.1 [0 – 3.54]	-1.4 [-4.48 – 1.64]
	Lower LoA	-1.74 [-2.55 – -0.99]	-4.02 [-5.96 – -1.95]	-8.94 [-15.47 – -3.72]
	Upper LoA	2.29 [1.51 – 3.38]	7.58 [5.35 – 12.94]	7.12 [2.82 – 12.2]
	LoA Spread	4.09 [3.16 – 5.43]	12.41 [9.17 – 17.14]	16.3 [8.8 – 25]
	Relative Bias	5.96 [-8.51 – 19.86]	18.13 [-0.01 – 30.31]	-8.29 [-24.51 – 10.67]
	Regression Slope	1.9 [1.7 – 2]	2 [2 – 2]	2 [2 – 2]
	Regression Intercept	-130 [-150 – -112.5]	-130 [-145 – -115]	-140 [-150 – -120]
COx_L	Bias	–	–	0.01 [-0.08 – 0.08]
	Lower LoA	–	–	-0.63 [-0.77 – -0.54]
	Upper LoA	–	–	0.67 [0.56 – 0.75]
	LoA Spread	–	–	1.3 [1.2 – 1.46]
	Relative Bias	–	–	0.51 [-5.39 – 6.63]
	Regression Slope	–	–	2 [2 – 2]
	Regression Intercept	–	–	-0.08 [-0.28 – 0.06]
COx_R	Bias	–	–	-0.01 [-0.09 – 0.07]
	Lower LoA	–	–	-0.63 [-0.77 – -0.53]
	Upper LoA	–	–	0.63 [0.51 – 0.72]
	LoA Spread	–	–	1.28 [1.15 – 1.42]
	Relative Bias	–	–	-0.64 [-6.58 – 5.35]
	Regression Slope	–	–	2 [2 – 2]
	Regression Intercept	–	–	-0.09 [-0.32 – 0.11]
COx-a_L	Bias	0 [-0.19 – 0.17]	-0.01 [-0.18 – 0.08]	0.01 [-0.06 – 0.06]
	Lower LoA	-0.28 [-0.56 – -0.13]	-0.75 [-1.15 – -0.55]	-0.62 [-0.7 – -0.55]
	Upper LoA	0.26 [0.13 – 0.49]	0.68 [0.51 – 0.86]	0.65 [0.57 – 0.73]
	LoA Spread	0.65 [0.46 – 0.84]	1.51 [1.16 – 1.87]	1.27 [1.12 – 1.39]
	Relative Bias	0.39 [-33.24 – 27.67]	-0.62 [-11.42 – 7.04]	0.53 [-4.78 – 4.76]
	Regression Slope	2 [1.8 – 2]	2 [1.8 – 2]	2 [2 – 2]
	Regression Intercept	-0.22 [-0.65 – 0.1]	-0.39 [-0.57 – -0.24]	-0.2 [-0.34 – 0.01]
COx-a_R	Bias	-0.03 [-0.19 – 0.15]	-0.06 [-0.17 – 0.2]	-0.02 [-0.07 – 0.05]
	Lower LoA	-0.31 [-0.53 – -0.14]	-0.76 [-1.01 – -0.43]	-0.65 [-0.73 – -0.55]
	Upper LoA	0.27 [0.13 – 0.49]	0.77 [0.59 – 1.06]	0.6 [0.53 – 0.69]
	LoA Spread	0.66 [0.48 – 0.81]	1.52 [1.3 – 1.77]	1.26 [1.11 – 1.36]
	Relative Bias	-6.59 [-26.74 – 28.61]	-2.76 [-10.97 – 17.53]	-1.56 [-5.66 – 3.61]
	Regression Slope	2 [1.8 – 2]	1.9 [1.55 – 2]	2 [2 – 2]
	Regression Intercept	-0.21 [-0.61 – 0.23]	-0.36 [-0.54 – -0.11]	-0.18 [-0.38 – -0.01]

COx, cerebral oximetry index with cerebral perfusion pressure; COx-a, cerebral oximetry index with arterial blood pressure; HC, healthy control volunteer group; IQR, interquartile range; LoA, limit of agreement; rSO_2, regional cerebral oxygen saturation; SP, elective spinal surgery patient group; TBI, traumatic brain injury patient group.

File S8b: Anchored-Interval – Bland-Altman Analysis of rSO_2 and COx/COx-a in All Populations using 10-Second Temporal Resolution

Physiologic Variable	Value	Median [IQR]								
		5-Minute Interval	10-Minute Interval	15-Minute Interval	30-Minute Interval	1-Hour Interval	2-Hour Interval	6-Hour Interval	12-Hour Interval	1-Day Interval
**HC Population**										
rSO_2_L	Bias	0.09 [-0.31 – 0.68]	1.48 [1.48 – 1.48]	–	–	–	–	–	–	–
	Lower LoA	-1.96 [-2.72 – -1.2]	0 [0 – 0]	–	–	–	–	–	–	–
	Upper LoA	2.08 [1.57 – 3.17]	2.96 [2.96 – 2.96]	–	–	–	–	–	–	–
	LoA Spread	3.92 [3.12 – 5.3]	2.96 [2.96 – 2.96]	–	–	–	–	–	–	–
	Relative Bias	2.32 [-8.52 – 16.31]	49.88 [49.88 – 49.88]	–	–	–	–	–	–	–
	Regression Slope	1.3 [0.71 – 1.9]	0.94 [0.94 – 0.94]	–	–	–	–	–	–	–
	Regression Intercept	-97 [-130 – -49.75]	-73 [-73 – -73]	–	–	–	–	–	–	–
rSO_2_R	Bias	0.17 [-0.35 – 0.68]	1.6 [1.6 – 1.6]	–	–	–	–	–	–	–
	Lower LoA	-1.93 [-2.67 – -1.26]	-0.54 [-0.54 – -0.54]	–	–	–	–	–	–	–
	Upper LoA	2.29 [1.46 – 3.33]	3.74 [3.74 – 3.74]	–	–	–	–	–	–	–
	LoA Spread	4.14 [3.23 – 5.71]	4.28 [4.28 – 4.28]	–	–	–	–	–	–	–
	Relative Bias	3.64 [-9.65 – 16.45]	37.27 [37.27 – 37.27]	–	–	–	–	–	–	–
	Regression Slope	1.5 [0.73 – 1.8]	1.5 [1.5 – 1.5]	–	–	–	–	–	–	–
	Regression Intercept	-100 [-130 – -52]	-96 [-96 – -96]	–	–	–	–	–	–	–
COx_L	Bias	–	–	–	–	–	–	–	–	–
	Lower LoA	–	–	–	–	–	–	–	–	–
	Upper LoA	–	–	–	–	–	–	–	–	–
	LoA Spread	–	–	–	–	–	–	–	–	–
	Relative Bias	–	–	–	–	–	–	–	–	–
	Regression Slope	–	–	–	–	–	–	–	–	–
	Regression Intercept	–	–	–	–	–	–	–	–	–
COx_R	Bias	–	–	–	–	–	–	–	–	–
	Lower LoA	–	–	–	–	–	–	–	–	–
	Upper LoA	–	–	–	–	–	–	–	–	–
	LoA Spread	–	–	–	–	–	–	–	–	–
	Relative Bias	–	–	–	–	–	–	–	–	–
	Regression Slope	–	–	–	–	–	–	–	–	–
	Regression Intercept	–	–	–	–	–	–	–	–	–
COx-a_L	Bias	0.01 [-0.16 – 0.13]	0.19 [0.19 – 0.19]	–	–	–	–	–	–	–
	Lower LoA	-0.31 [-0.48 – -0.2]	-0.33 [-0.33 – -0.33]	–	–	–	–	–	–	–
	Upper LoA	2.35 [-25.22 – 18.41]	18.09 [18.09 – 18.09]	–	–	–	–	–	–	–
	LoA Spread	0.69 [0.48 – 0.92]	1.03 [1.03 – 1.03]	–	–	–	–	–	–	–
	Relative Bias	0.3 [0.17 – 0.49]	0.7 [0.7 – 0.7]	–	–	–	–	–	–	–
	Regression Slope	-0.11 [-0.32 – 0.11]	-0.29 [-0.29 – -0.29]	–	–	–	–	–	–	–
	Regression Intercept	0.85 [0.4 – 1.6]	2 [2 – 2]	–	–	–	–	–	–	–
COx-a_R	Bias	-0.02 [-0.14 – 0.13]	0.14 [0.14 – 0.14]	–	–	–	–	–	–	–
	Lower LoA	-0.32 [-0.5 – -0.18]	-0.23 [-0.23 – -0.23]	–	–	–	–	–	–	–
	Upper LoA	-2.87 [-22.11 – 19.47]	18.96 [18.96 – 18.96]	–	–	–	–	–	–	–
	LoA Spread	0.66 [0.47 – 0.84]	0.75 [0.75 – 0.75]	–	–	–	–	–	–	–
	Relative Bias	0.28 [0.16 – 0.52]	0.52 [0.52 – 0.52]	–	–	–	–	–	–	–
	Regression Slope	-0.08 [-0.36 – 0.15]	-0.33 [-0.33 – -0.33]	–	–	–	–	–	–	–
	Regression Intercept	0.94 [0.32 – 1.6]	2 [2 – 2]	–	–	–	–	–	–	–
**SP Population**										
rSO_2_L	Bias	0.47 [-0.06 – 0.73]	0.76 [0.13 – 1.35]	1.03 [-0.02 – 2.58]	0.76 [0.02 – 3.79]	0.89 [-3.79 – 1.63]	–	–	–	–
	Lower LoA	-2.78 [-4.43 – -1.75]	-3.15 [-5.92 – -1.82]	-3.41 [-4.88 – -1.78]	-3.3 [-6.53 – -1.59]	-7.15 [-30.37 – -3.83]	–	–	–	–
	Upper LoA	3.61 [2.93 – 6.45]	5.02 [3.76 – 7.98]	6.07 [3.15 – 9.19]	8.12 [4.51 – 12.02]	8.94 [7.09 – 22.78]	–	–	–	–
	LoA Spread	6.95 [4.47 – 10.65]	7.7 [6.06 – 14.8]	9.21 [6.47 – 14.88]	9.72 [8.5 – 19.49]	16.09 [10.92 – 53.14]	–	–	–	–
	Relative Bias	5.34 [-1.08 – 11.44]	8.68 [1.77 – 19.04]	7.81 [-0.45 – 20.96]	7.88 [0.29 – 29.78]	5.55 [-1.93 – 23.4]	–	–	–	–

	Regression Slope	0.12 [0.02 – 0.35]	0.27 [0 – 0.96]	0.62 [0.1 – 1.5]	0.79 [0.41 – 1.9]	1.8 [1.36 – 1.85]	–	–	–	–
	Regression Intercept	-5.5 [-22.5 – -0.55]	-13 [-58.5 – 1.45]	-34 [-110 – -3.8]	-51 [-110 – -24]	-130 [-140 – -94]	–	–	–	–
rSO_2_R	Bias	0.45 [-0.14 – 0.89]	0.7 [-0.22 – 1.53]	1.04 [0.02 – 2.77]	1.18 [-0.29 – 3.65]	-0.76 [-5.06 – 0.58]	–	–	–	–
	Lower LoA	-2.85 [-4.4 – -1.92]	-3.39 [-6.54 – -2.03]	-3.95 [-6.57 – -2.44]	-3.85 [-6.64 – -1.94]	-11.39 [-34.02 – -6.12]	–	–	–	–
	Upper LoA	4.01 [2.46 – 5.42]	5.3 [2.71 – 8.35]	7.08 [3.36 – 9.34]	7.58 [4.24 – 12.56]	9.86 [7.27 – 23.9]	–	–	–	–
	LoA Spread	7.23 [4.87 – 9.27]	8.38 [5.88 – 12.48]	11.01 [6.81 – 14.31]	10.12 [6.15 – 17.72]	21.25 [13.39 – 57.91]	–	–	–	–
	Relative Bias	4.95 [-1.54 – 11.22]	6.73 [-1.47 – 14.08]	6.97 [0.73 – 17.4]	11.74 [-2.66 – 24.01]	-3.59 [-6.74 – 15.55]	–	–	–	–
	Regression Slope	0.32 [0.04 – 0.44]	0.52 [0.16 – 1.15]	0.65 [0.12 – 1.1]	1.2 [0.66 – 1.9]	1.9 [1.45 – 1.95]	–	–	–	–
	Regression Intercept	-21 [-29.5 – -2.5]	-32 [-66.5 – -10.8]	-42 [-69.5 – -9.8]	-66 [-110 – -34]	-130 [-135 – -101]	–	–	–	–
COx_L	Bias	–	–	–	–	–	–	–	–	–
	Lower LoA	–	–	–	–	–	–	–	–	–
	Upper LoA	–	–	–	–	–	–	–	–	–
	LoA Spread	–	–	–	–	–	–	–	–	–
	Relative Bias	–	–	–	–	–	–	–	–	–
	Regression Slope	–	–	–	–	–	–	–	–	–
	Regression Intercept	–	–	–	–	–	–	–	–	–
COx_R	Bias	–	–	–	–	–	–	–	–	–
	Lower LoA	–	–	–	–	–	–	–	–	–
	Upper LoA	–	–	–	–	–	–	–	–	–
	LoA Spread	–	–	–	–	–	–	–	–	–
	Relative Bias	–	–	–	–	–	–	–	–	–
	Regression Slope	–	–	–	–	–	–	–	–	–
	Regression Intercept	–	–	–	–	–	–	–	–	–
COx-a_L	Bias	0.02 [-0.05 – 0.07]	0 [-0.07 – 0.07]	0 [-0.16 – 0.09]	0.02 [-0.21 – 0.11]	-0.03 [-0.07 – 0.01]	–	–	–	–
	Lower LoA	-0.66 [-0.88 – -0.5]	-0.74 [-0.93 – -0.6]	-0.73 [-1.02 – -0.64]	-0.72 [-1.07 – -0.48]	-0.74 [-0.8 – -0.64]	–	–	–	–
	Upper LoA	1.03 [-4.72 – 5.35]	-0.12 [-4.37 – 4.05]	0.2 [-11.75 – 5.61]	1.12 [-13.91 – 9.7]	-2.27 [-5.06 – 1.06]	–	–	–	–
	LoA Spread	1.33 [1.04 – 1.76]	1.41 [1.23 – 1.76]	1.47 [1.19 – 1.9]	1.45 [1.16 – 1.96]	1.41 [1.31 – 1.46]	–	–	–	–
	Relative Bias	0.67 [0.55 – 0.89]	0.73 [0.61 – 0.92]	0.7 [0.61 – 0.9]	0.7 [0.64 – 0.76]	0.65 [0.64 – 0.66]	–	–	–	–
	Regression Slope	-0.08 [-0.14 – -0.01]	-0.14 [-0.29 – 0.01]	-0.2 [-0.38 – -0.08]	-0.3 [-0.57 – -0.17]	-0.34 [-0.44 – -0.34]	–	–	–	–
	Regression Intercept	0.44 [0.19 – 0.81]	0.83 [0.32 – 1.2]	1.1 [0.76 – 1.4]	1.5 [1.3 – 1.9]	1.6 [1.5 – 1.7]	–	–	–	–
COx-a_R	Bias	0.03 [-0.07 – 0.07]	0 [-0.07 – 0.12]	-0.01 [-0.14 – 0.16]	-0.04 [-0.16 – 0.17]	0.05 [-0.03 – 0.08]	–	–	–	–
	Lower LoA	-0.76 [-0.93 – -0.54]	-0.76 [-0.98 – -0.57]	-0.79 [-1 – -0.57]	-0.68 [-1.02 – -0.52]	-0.66 [-0.8 – -0.55]	–	–	–	–
	Upper LoA	1.61 [-4.58 – 5.62]	0.23 [-4.65 – 8.12]	-0.43 [-8.03 – 8.75]	-1.65 [-11.23 – 12.38]	3.54 [-1.71 – 6.88]	–	–	–	–
	LoA Spread	1.48 [1.29 – 1.69]	1.57 [1.32 – 1.87]	1.53 [1.25 – 1.9]	1.45 [1.13 – 1.72]	1.41 [1.28 – 1.53]	–	–	–	–
	Relative Bias	0.72 [0.62 – 0.89]	0.74 [0.67 – 1.03]	0.74 [0.67 – 1.01]	0.74 [0.67 – 1.02]	0.71 [0.7 – 0.73]	–	–	–	–
	Regression Slope	-0.08 [-0.21 – 0.01]	-0.15 [-0.28 – -0.02]	-0.23 [-0.38 – -0.02]	-0.33 [-0.41 – -0.25]	-0.29 [-0.35 – -0.28]	–	–	–	–
	Regression Intercept	0.64 [0.3 – 0.86]	0.96 [0.52 – 1.2]	1.1 [0.9 – 1.45]	1.5 [1.3 – 1.9]	1.5 [1.45 – 1.75]	–	–	–	–
**TBI Population**										
rSO_2_L	Bias	-0.01 [-0.04 – 0.04]	0 [-0.09 – 0.08]	-0.01 [-0.15 – 0.06]	0.02 [-0.1 – 0.22]	-0.03 [-0.31 – 0.27]	-0.14 [-0.62 – 0.45]	-0.16 [-1.52 – 1.15]	-0.74 [-2.15 – 1.2]	-1.84 [-3.16 – 1.27]
	Lower LoA	-3.65 [-6.31 – -2.48]	-4.38 [-7.34 – -2.83]	-4.67 [-7.88 – -2.86]	-5.32 [-8.18 – -3.34]	-6.46 [-10.03 – -4.13]	-7.3 [-11.47 – -4.05]	-7.85 [-14.28 – -4.79]	-9.71 [-16.11 – -6.13]	-10.77 [-19.53 – -6.87]
	Upper LoA	3.89 [2.4 – 6.26]	4.39 [2.82 – 7.41]	5.05 [2.92 – 8.15]	5.49 [3.55 – 8.95]	6.47 [4.06 – 10.49]	7.43 [4.55 – 12.46]	8.32 [5.17 – 12.15]	7.32 [4.87 – 15.15]	10.39 [5.99 – 19.09]
	LoA Spread	7.53 [4.96 – 12.43]	8.71 [5.69 – 14.78]	9.69 [5.77 – 15.62]	10.78 [6.93 – 16.81]	13.3 [7.7 – 19.82]	14.61 [8.69 – 24.16]	16.36 [10.69 – 25.58]	16.7 [11.16 – 26.33]	18.75 [14.24 – 40.25]
	Relative Bias	-0.1 [-0.79 – 0.56]	0 [-1.1 – 0.63]	-0.1 [-0.98 – 0.95]	0.25 [-1.16 – 1.78]	-0.29 [-2.12 – 2.13]	-0.88 [-3.45 – 4.05]	-1.75 [-7.13 – 8.26]	-2.62 [-11.29 – 6.41]	-7.54 [-15.03 – 8.14]
	Regression Slope	0.07 [0.01 – 0.15]	0.1 [0.02 – 0.22]	0.1 [0.02 – 0.28]	0.14 [0.03 – 0.41]	0.31 [0.04 – 0.63]	0.47 [0.06 – 0.95]	0.88 [0.2 – 1.7]	1.3 [0.5 – 2]	1.8 [1.15 – 2]
	Regression Intercept	-4.45 [-10.75 – -0.72]	-6.3 [-17.75 – -1.53]	-6.9 [-19.75 – -1.55]	-9.45 [-30 – -1.33]	-17.5 [-43.75 – -2.58]	-30 [-56.5 – -4.28]	-54 [-110 – -14.25]	-81 [-120 – -32.5]	-97.5 [-132.5 – -77.5]

rSO_2_R	Bias	0 [-0.04 – 0.05]	-0.02 [-0.12 – 0.06]	-0.02 [-0.12 – 0.12]	-0.04 [-0.26 – 0.14]	-0.15 [-0.58 – 0.23]	-0.08 [-0.83 – 0.44]	-0.57 [-1.92 – 0.93]	-1.33 [-2.57 – 0.74]	-2.05 [-2.8 – 2.14]
	Lower LoA	-3.86 [-6.25 – -2.28]	-4.55 [-8.03 – -2.49]	-5.23 [-8.9 – -2.68]	-6.23 [-10.12 – -3.29]	-6.92 [-11.28 – -3.47]	-8.01 [-12.4 – -3.87]	-9.77 [-14.87 – -5.68]	-11.28 [-17.78 – -5.84]	-8.77 [-21.21 – -6.97]
	Upper LoA	3.99 [2.22 – 6.27]	4.7 [2.56 – 8.03]	5.24 [2.99 – 8.58]	6.17 [3.61 – 9.7]	6.98 [3.9 – 10.36]	7.74 [3.95 – 12.23]	8.33 [4.98 – 14.01]	8.67 [4.4 – 15.7]	10.3 [6.29 – 21.07]
	LoA Spread	7.71 [4.52 – 12.44]	9.25 [5.12 – 16.29]	10.42 [5.84 – 17.58]	12.38 [7.29 – 19.22]	13.9 [7.4 – 21.14]	15.35 [8.55 – 25.36]	20.9 [10.93 – 27.74]	15.98 [13.05 – 31.78]	20.16 [13.15 – 37.02]
	Relative Bias	0.06 [-0.66 – 0.77]	-0.34 [-1.08 – 0.68]	-0.3 [-1.26 – 1.16]	-0.32 [-2.92 – 1.38]	-0.97 [-3.51 – 1.76]	-0.63 [-5.09 – 2.46]	-3.88 [-9.89 – 4.07]	-5.6 [-17.67 – 3.49]	-2.48 [-16.98 – 12.39]
	Regression Slope	0.06 [0.02 – 0.17]	0.1 [0.03 – 0.22]	0.09 [0.02 – 0.24]	0.1 [0 – 0.36]	0.21 [0.04 – 0.54]	0.33 [0.15 – 0.69]	0.67 [0.16 – 1.7]	1.4 [0.46 – 2]	1.6 [1.2 – 1.93]
	Regression Intercept	-4.2 [-10 – -1]	-6.15 [-14 – -1.9]	-6.35 [-14.75 – -1.13]	-7.15 [-24 – 0.04]	-12 [-35 – -2.45]	-20 [-52.25 – -8.8]	-44 [-107.5 – -13]	-91 [-120 – -35.5]	-97 [-110 – -80.25]
COx_L	Bias	0 [-0.01 – 0.01]	0 [-0.01 – 0.02]	0 [-0.01 – 0.02]	0 [-0.01 – 0.02]	0 [-0.02 – 0.03]	0.01 [-0.02 – 0.03]	0.01 [-0.02 – 0.04]	0.02 [-0.03 – 0.08]	0.01 [-0.04 – 0.08]
	Lower LoA	-0.55 [-0.62 – -0.5]	-0.6 [-0.67 – -0.56]	-0.62 [-0.72 – -0.58]	-0.64 [-0.74 – -0.59]	-0.65 [-0.74 – -0.6]	-0.65 [-0.75 – -0.6]	-0.66 [-0.75 – -0.6]	-0.65 [-0.73 – -0.55]	-0.63 [-0.76 – -0.54]
	Upper LoA	0.56 [0.5 – 0.62]	0.62 [0.56 – 0.69]	0.63 [0.57 – 0.71]	0.64 [0.58 – 0.73]	0.66 [0.6 – 0.74]	0.66 [0.6 – 0.74]	0.68 [0.61 – 0.77]	0.69 [0.61 – 0.77]	0.7 [0.66 – 0.77]
	LoA Spread	1.1 [1.01 – 1.24]	1.21 [1.1 – 1.38]	1.25 [1.14 – 1.42]	1.27 [1.17 – 1.46]	1.3 [1.2 – 1.45]	1.3 [1.21 – 1.49]	1.35 [1.23 – 1.48]	1.33 [1.24 – 1.49]	1.35 [1.26 – 1.52]
	Relative Bias	0.21 [-0.99 – 1.32]	0.17 [-0.81 – 1.7]	0.07 [-0.73 – 1.47]	0.24 [-0.96 – 1.66]	0.26 [-1.38 – 2.04]	0.36 [-1.22 – 2.26]	0.61 [-1.43 – 3.13]	1.25 [-2.42 – 6.83]	0.92 [-2.64 – 7.12]
	Regression Slope	0.67 [0.59 – 0.77]	1.1 [0.92 – 1.2]	1.3 [1.1 – 1.4]	1.5 [1.3 – 1.7]	1.7 [1.5 – 1.8]	1.8 [1.6 – 1.9]	1.9 [1.7 – 2]	2 [1.9 – 2]	2 [1.7 – 2]
	Regression Intercept	-0.02 [-0.08 – 0.02]	-0.02 [-0.13 – 0.03]	-0.05 [-0.14 – 0.02]	-0.06 [-0.18 – 0.04]	-0.07 [-0.22 – 0.03]	-0.06 [-0.22 – 0.04]	-0.05 [-0.24 – 0.05]	-0.05 [-0.21 – 0.07]	-0.09 [-0.22 – 0.09]
COx_R	Bias	0 [-0.01 – 0.01]	0 [-0.01 – 0.01]	0 [-0.01 – 0.01]	0 [-0.02 – 0.01]	0 [-0.02 – 0.01]	0 [-0.03 – 0.02]	0 [-0.04 – 0.02]	0 [-0.05 – 0.03]	-0.02 [-0.1 – 0.04]
	Lower LoA	-0.54 [-0.62 – -0.5]	-0.6 [-0.69 – -0.54]	-0.61 [-0.7 – -0.56]	-0.62 [-0.73 – -0.58]	-0.64 [-0.73 – -0.58]	-0.65 [-0.76 – -0.58]	-0.66 [-0.77 – -0.6]	-0.65 [-0.76 – -0.55]	-0.69 [-0.84 – -0.63]
	Upper LoA	0.55 [0.49 – 0.62]	0.6 [0.54 – 0.67]	0.62 [0.54 – 0.69]	0.63 [0.56 – 0.71]	0.64 [0.56 – 0.72]	0.65 [0.56 – 0.74]	0.67 [0.56 – 0.74]	0.67 [0.56 – 0.72]	0.66 [0.58 – 0.74]
	LoA Spread	1.07 [0.99 – 1.25]	1.2 [1.08 – 1.36]	1.24 [1.12 – 1.4]	1.28 [1.14 – 1.42]	1.3 [1.15 – 1.41]	1.32 [1.17 – 1.46]	1.35 [1.18 – 1.46]	1.31 [1.16 – 1.46]	1.39 [1.26 – 1.53]
	Relative Bias	-0.16 [-1.11 – 0.86]	0.08 [-1.06 – 1.04]	0.06 [-1.01 – 0.72]	-0.02 [-1.32 – 1.15]	-0.17 [-1.4 – 0.95]	-0.28 [-1.99 – 1.41]	-0.18 [-2.55 – 1.47]	0.12 [-3.73 – 2.09]	-1.68 [-7.25 – 2.78]
	Regression Slope	0.69 [0.58 – 0.77]	1.1 [0.92 – 1.2]	1.25 [1.2 – 1.4]	1.5 [1.4 – 1.6]	1.7 [1.5 – 1.8]	1.8 [1.6 – 1.9]	1.9 [1.8 – 2]	2 [1.8 – 2]	2 [1.8 – 2]
	Regression Intercept	0 [-0.01 – 0.01]	0 [-0.01 – 0.01]	0 [-0.01 – 0.01]	0 [-0.01 – 0.02]	0 [-0.02 – 0.02]	0 [-0.02 – 0.02]	0.01 [-0.02 – 0.02]	0.01 [-0.01 – 0.03]	0.01 [-0.02 – 0.04]
COx-a_L	Bias	0.12 [-0.76 – 1.36]	0.08 [-1 – 1.3]	0.05 [-0.96 – 1.24]	0.13 [-1.01 – 1.47]	0.25 [-1.19 – 1.65]	0.23 [-1.72 – 1.44]	0.7 [-1.29 – 1.75]	1.16 [-1.25 – 2.65]	0.82 [-1.3 – 4.14]
	Lower LoA	1.09 [1 – 1.2]	1.17 [1.05 – 1.3]	1.23 [1.11 – 1.34]	1.26 [1.14 – 1.37]	1.28 [1.17 – 1.38]	1.27 [1.16 – 1.4]	1.3 [1.19 – 1.39]	1.27 [1.19 – 1.38]	1.27 [1.17 – 1.39]
	Upper LoA	-0.53 [-0.6 – -0.49]	-0.58 [-0.65 – -0.53]	-0.6 [-0.67 – -0.56]	-0.62 [-0.69 – -0.57]	-0.62 [-0.68 – -0.58]	-0.63 [-0.7 – -0.59]	-0.64 [-0.7 – -0.59]	-0.62 [-0.7 – -0.56]	-0.61 [-0.72 – -0.54]
	LoA Spread	0.55 [0.49 – 0.6]	0.6 [0.54 – 0.65]	0.63 [0.56 – 0.68]	0.64 [0.56 – 0.69]	0.64 [0.59 – 0.69]	0.65 [0.58 – 0.71]	0.66 [0.59 – 0.71]	0.66 [0.59 – 0.72]	0.65 [0.6 – 0.68]
	Relative Bias	-0.07 [-0.11 – -0.02]	-0.11 [-0.18 – -0.03]	-0.13 [-0.21 – -0.03]	-0.16 [-0.25 – -0.04]	-0.19 [-0.3 – -0.04]	-0.21 [-0.31 – -0.02]	-0.21 [-0.31 – -0.02]	-0.21 [-0.32 – -0.03]	-0.2 [-0.3 – -0.05]
	Regression Slope	0.69 [0.64 – 0.76]	1.1 [1 – 1.2]	1.3 [1.2 – 1.4]	1.5 [1.5 – 1.6]	1.7 [1.6 – 1.8]	1.8 [1.7 – 1.9]	1.9 [1.8 – 1.98]	2 [1.9 – 2]	2 [1.9 – 2]
	Regression Intercept	0 [-0.01 – 0.01]	0 [-0.01 – 0.01]	0 [-0.02 – 0.01]	0 [-0.02 – 0]	0 [-0.03 – 0.01]	-0.01 [-0.03 – 0.02]	-0.01 [-0.04 – 0.01]	-0.01 [-0.05 – 0.02]	-0.03 [-0.09 – 0.01]
COx-a_R	Bias	0.11 [-0.71 – 0.65]	0.05 [-0.97 – 0.72]	-0.16 [-1.51 – 0.53]	-0.22 [-1.58 – 0.45]	-0.22 [-2.37 – 0.99]	-0.57 [-2.14 – 1.18]	-0.81 [-2.95 – 1.2]	-0.59 [-3.94 – 1.61]	-2.33 [-6.8 – 0.9]
	Lower LoA	1.07 [0.95 – 1.19]	1.19 [1.04 – 1.28]	1.22 [1.09 – 1.31]	1.25 [1.1 – 1.35]	1.28 [1.13 – 1.36]	1.28 [1.13 – 1.36]	1.29 [1.15 – 1.37]	1.26 [1.13 – 1.37]	1.29 [1.22 – 1.35]
	Upper LoA	-0.53 [-0.59 – -0.48]	-0.59 [-0.64 – -0.52]	-0.61 [-0.66 – -0.54]	-0.63 [-0.68 – -0.56]	-0.64 [-0.7 – -0.56]	-0.65 [-0.71 – -0.57]	-0.66 [-0.72 – -0.58]	-0.64 [-0.72 – -0.56]	-0.65 [-0.75 – -0.63]
	LoA Spread	0.54 [0.49 – 0.6]	0.59 [0.53 – 0.65]	0.61 [0.55 – 0.66]	0.63 [0.56 – 0.67]	0.63 [0.55 – 0.69]	0.63 [0.56 – 0.69]	0.62 [0.56 – 0.67]	0.62 [0.53 – 0.68]	0.62 [0.56 – 0.68]
	Relative Bias	-0.05 [-0.11 – 0]	-0.09 [-0.18 – -0.01]	-0.12 [-0.22 – -0.01]	-0.15 [-0.26 – -0.02]	-0.16 [-0.29 – -0.01]	-0.18 [-0.32 – -0.02]	-0.19 [-0.32 – -0.03]	-0.23 [-0.33 – -0.05]	-0.24 [-0.39 – -0.13]
	Regression Slope	0.7 [0.64 – 0.77]	1.1 [1 – 1.2]	1.3 [1.2 – 1.4]	1.6 [1.4 – 1.7]	1.7 [1.6 – 1.8]	1.8 [1.7 – 1.9]	1.9 [1.8 – 2]	2 [1.9 – 2]	2 [1.85 – 2]
	Regression Intercept	-0.02 [-0.08 – 0.03]	-0.04 [-0.12 – 0.05]	-0.05 [-0.14 – 0.05]	-0.05 [-0.17 – 0.05]	-0.08 [-0.19 – 0.05]	-0.08 [-0.22 – 0.1]	-0.06 [-0.24 – 0.07]	-0.04 [-0.26 – 0.1]	-0.14 [-0.33 – 0.14]

COx, cerebral oximetry index with cerebral perfusion pressure; COx-a, cerebral oximetry index with arterial blood pressure; HC, healthy control volunteer group; IQR, interquartile range; r-value, Pearson correlation coefficient; rSO_2, regional cerebral oxygen saturation; SP, elective spinal surgery patient group; TBI, traumatic brain injury patient group.

File S8c: Windowed-Point – Bland-Altman Analysis of rSO_2 and COx/COx-a in All Populations using 10-Second Temporal Resolution

Physiologic Variable	Value	Median [IQR]								
		5-Minute Interval	10-Minute Interval	15-Minute Interval	30-Minute Interval	1-Hour Interval	2-Hour Interval	6-Hour Interval	12-Hour Interval	1-Day Interval
**HC Population**										
rSO_2_L	Bias	0 [-0.03 – 0.02]	0 [-0.03 – 0.03]	0.01 [-0.03 – 0.04]	0.03 [-0.08 – 0.18]	–	–	–	–	–
	Lower LoA	-1.7 [-2.04 – -1.24]	-1.52 [-1.86 – -1.18]	-1.44 [-1.82 – -1.13]	-1.3 [-1.9 – -0.9]	–	–	–	–	–
	Upper LoA	1.69 [1.27 – 2.05]	1.54 [1.19 – 1.9]	1.47 [1.08 – 1.82]	1.42 [1.04 – 2.08]	–	–	–	–	–
	LoA Spread	3.36 [2.48 – 4.15]	3.07 [2.34 – 3.71]	2.91 [2.23 – 3.66]	2.65 [1.9 – 3.84]	–	–	–	–	–
	Relative Bias	-0.08 [-0.92 – 0.79]	0.09 [-1 – 1.27]	0.13 [-1.01 – 1.63]	0.74 [-2.45 – 4.42]	–	–	–	–	–
	Regression Slope	0.01 [-0.03 – 0.06]	0.07 [0.03 – 0.16]	0.12 [0.03 – 0.21]	0.12 [0.02 – 0.34]	–	–	–	–	–
	Regression Intercept	-1.04 [-4.23 – 1.7]	-4.85 [-11 – -1.9]	-8.85 [-16 – -1.85]	-7.55 [-22.75 – 0.01]	–	–	–	–	–
rSO_2_R	Bias	-0.01 [-0.04 – 0.04]	0.01 [-0.03 – 0.04]	0 [-0.05 – 0.06]	0.02 [-0.12 – 0.18]	–	–	–	–	–
	Lower LoA	-1.82 [-2.4 – -1.5]	-1.64 [-2.17 – -1.31]	-1.59 [-2.09 – -1.31]	-1.42 [-2.02 – -1.1]	–	–	–	–	–
	Upper LoA	1.8 [1.48 – 2.3]	1.67 [1.36 – 2.15]	1.59 [1.28 – 2.05]	1.5 [1.17 – 2.18]	–	–	–	–	–
	LoA Spread	3.66 [3 – 4.75]	3.33 [2.65 – 4.24]	3.15 [2.66 – 4.18]	2.92 [2.3 – 4.07]	–	–	–	–	–
	Relative Bias	-0.21 [-1.13 – 0.89]	0.27 [-0.91 – 1.15]	0 [-1.44 – 1.78]	1 [-3.04 – 4.51]	–	–	–	–	–
	Regression Slope	0 [-0.03 – 0.06]	0.08 [0.01 – 0.17]	0.1 [0.03 – 0.24]	0.18 [0.02 – 0.47]	–	–	–	–	–
	Regression Intercept	-0.34 [-4.3 – 1.7]	-5 [-11.75 – -0.99]	-7.85 [-16.75 – -2.45]	-11 [-33 – -0.9]	–	–	–	–	–
COx_L	Bias	–	–	–	–	–	–	–	–	–
	Lower LoA	–	–	–	–	–	–	–	–	–
	Upper LoA	–	–	–	–	–	–	–	–	–
	LoA Spread	–	–	–	–	–	–	–	–	–
	Relative Bias	–	–	–	–	–	–	–	–	–
	Regression Slope	–	–	–	–	–	–	–	–	–
	Regression Intercept	–	–	–	–	–	–	–	–	–
COx_R	Bias	–	–	–	–	–	–	–	–	–
	Lower LoA	–	–	–	–	–	–	–	–	–
	Upper LoA	–	–	–	–	–	–	–	–	–
	LoA Spread	–	–	–	–	–	–	–	–	–
	Relative Bias	–	–	–	–	–	–	–	–	–
	Regression Slope	–	–	–	–	–	–	–	–	–
	Regression Intercept	–	–	–	–	–	–	–	–	–
COx-a_L	Bias	0 [0 – 0]	0 [0 – 0]	0 [0 – 0]	0 [-0.01 – 0.01]	–	–	–	–	–
	Lower LoA	-0.13 [-0.15 – -0.12]	-0.12 [-0.13 – -0.11]	-0.12 [-0.13 – -0.1]	-0.11 [-0.12 – -0.08]	–	–	–	–	–
	Upper LoA	-0.05 [-0.89 – 0.92]	-0.21 [-1.14 – 0.66]	0.04 [-1.31 – 1.12]	0.34 [-4.39 – 2.83]	–	–	–	–	–
	LoA Spread	0.26 [0.23 – 0.3]	0.23 [0.21 – 0.27]	0.23 [0.21 – 0.26]	0.21 [0.16 – 0.25]	–	–	–	–	–
	Relative Bias	0.13 [0.12 – 0.15]	0.12 [0.11 – 0.13]	0.12 [0.1 – 0.14]	0.11 [0.08 – 0.12]	–	–	–	–	–
	Regression Slope	0 [0 – 0.01]	0 [0 – 0.01]	0 [0 – 0.01]	0 [-0.01 – 0.02]	–	–	–	–	–
	Regression Intercept	-0.02 [-0.03 – -0.02]	-0.02 [-0.03 – -0.01]	-0.02 [-0.03 – -0.01]	-0.04 [-0.13 – -0.01]	–	–	–	–	–
COx-a_R	Bias	0 [0 – 0]	0 [0 – 0]	0 [0 – 0]	0 [0 – 0.01]	–	–	–	–	–
	Lower LoA	-0.13 [-0.16 – -0.12]	-0.12 [-0.14 – -0.11]	-0.12 [-0.13 – -0.1]	-0.11 [-0.13 – -0.08]	–	–	–	–	–
	Upper LoA	-0.02 [-0.75 – 0.67]	-0.07 [-1.07 – 0.83]	-0.1 [-0.8 – 1.14]	0.79 [-2.09 – 3.7]	–	–	–	–	–
	LoA Spread	0.27 [0.23 – 0.31]	0.24 [0.21 – 0.27]	0.23 [0.21 – 0.27]	0.21 [0.17 – 0.25]	–	–	–	–	–
	Relative Bias	0.13 [0.12 – 0.15]	0.12 [0.11 – 0.14]	0.11 [0.1 – 0.14]	0.1 [0.08 – 0.13]	–	–	–	–	–
	Regression Slope	0 [0 – 0.01]	0 [0 – 0]	0 [0 – 0.01]	0.01 [-0.01 – 0.02]	–	–	–	–	–
	Regression Intercept	-0.03 [-0.04 – -0.02]	-0.02 [-0.03 – -0.01]	-0.02 [-0.04 – 0]	-0.04 [-0.15 – 0]	–	–	–	–	–
**SP Population**										
rSO_2_L	Bias	0.02 [0.01 – 0.08]	0.01 [0 – 0.06]	0.02 [0 – 0.05]	0.02 [0 – 0.05]	0.02 [0 – 0.03]	0.03 [0 – 0.1]	145.35 [72.36 – 218.35]	–	–
	Lower LoA	-2.39 [-4.45 – -1.57]	-2.8 [-5.44 – -1.23]	-1.87 [-4.8 – -1.16]	-2.2 [-4.41 – -0.97]	-1.08 [-2.29 – -0.85]	-1.13 [-2.11 – -0.78]	-5307.18 [-7954.95 – -2659.4]	–	–
	Upper LoA	2.33 [1.59 – 4.55]	2.8 [1.26 – 5.62]	1.91 [1.16 – 4.83]	2.22 [1 – 4.59]	1.1 [0.87 – 2.41]	1.21 [0.94 – 2.14]	5597.88 [2804.11 – 8391.65]	–	–
	LoA Spread	4.72 [3.16 – 9]	5.6 [2.49 – 11.06]	3.78 [2.33 – 9.67]	4.42 [1.97 – 8.86]	2.18 [1.76 – 4.7]	2.35 [1.76 – 4.25]	10905.06 [5463.51 – 16346.6]	–	–

	Relative Bias	0.6 [0.13 – 1.06]	0.37 [-0.02 – 1.16]	0.5 [-0.03 – 1.11]	0.54 [0.21 – 0.99]	0.57 [-0.18 – 1.14]	1.24 [0.22 – 3.83]	-0.79 [-1.86 – 0.27]	–	–
	Regression Slope	-0.02 [-0.07 – -0.01]	-0.01 [-0.07 – 0]	-0.01 [-0.04 – 0]	0 [-0.02 – 0.01]	0 [-0.02 – 0.01]	0.02 [0 – 0.03]	-0.3 [-1.15 – 0.55]	–	–
	Regression Intercept	1.2 [0.37 – 2.65]	0.43 [-0.08 – 2.6]	0.32 [-0.11 – 2.55]	0.19 [-0.67 – 0.97]	-0.25 [-0.93 – 0.57]	-1.1 [-1.98 – -0.27]	15 [-42.5 – 72.5]	–	–
rSO$_2$_R	Bias	0.01 [-0.01 – 0.07]	0.01 [-0.01 – 0.07]	0.01 [-0.01 – 0.03]	0.02 [0.01 – 0.07]	0.02 [0 – 0.04]	0.03 [0 – 0.06]	-0.29 [-0.33 – -0.25]	–	–
	Lower LoA	-3.15 [-6.03 – -1.4]	-2.17 [-7.46 – -1.07]	-1.58 [-7.26 – -1]	-1.16 [-2.81 – -0.92]	-0.95 [-2.06 – -0.8]	-1.21 [-1.53 – -0.87]	-17.8 [-20.16 – -15.45]	–	–
	Upper LoA	3.32 [1.36 – 6.29]	2.29 [1.06 – 7.51]	1.62 [1.03 – 7.13]	1.14 [0.96 – 3.05]	0.97 [0.83 – 2.08]	1.28 [0.95 – 1.66]	17.22 [14.94 – 19.49]	–	–
	LoA Spread	6.47 [2.75 – 12.28]	4.45 [2.13 – 15.08]	3.2 [2.03 – 14.39]	2.3 [1.89 – 5.82]	1.92 [1.64 – 4.13]	2.43 [1.81 – 3.22]	35.02 [30.4 – 39.65]	–	–
	Relative Bias	0.18 [-0.38 – 0.77]	0.56 [-0.18 – 0.97]	0.4 [-0.48 – 0.78]	0.67 [0.3 – 1.25]	0.74 [0.34 – 1.5]	1.17 [0.02 – 2.75]	-0.84 [-0.84 – -0.84]	–	–
	Regression Slope	-0.02 [-0.14 – -0.01]	-0.01 [-0.08 – 0]	-0.01 [-0.18 – 0]	0 [-0.02 – 0]	0.01 [0 – 0.02]	0.01 [0 – 0.05]	0.44 [0.22 – 0.65]	–	–
	Regression Intercept	1.6 [0.45 – 9.2]	0.81 [0.14 – 5.8]	0.38 [0.1 – 13.5]	0.21 [-0.07 – 1.75]	-0.42 [-1.28 – 0.01]	-0.7 [-2.8 – -0.05]	-28.98 [-42.99 – -14.97]	–	–
COx_L	Bias	–	–	–	–	–	–	–	–	–
	Lower LoA	–	–	–	–	–	–	–	–	–
	Upper LoA	–	–	–	–	–	–	–	–	–
	LoA Spread	–	–	–	–	–	–	–	–	–
	Relative Bias	–	–	–	–	–	–	–	–	–
	Regression Slope	–	–	–	–	–	–	–	–	–
	Regression Intercept	–	–	–	–	–	–	–	–	–
COx_R	Bias	–	–	–	–	–	–	–	–	–
	Lower LoA	–	–	–	–	–	–	–	–	–
	Upper LoA	–	–	–	–	–	–	–	–	–
	LoA Spread	–	–	–	–	–	–	–	–	–
	Relative Bias	–	–	–	–	–	–	–	–	–
	Regression Slope	–	–	–	–	–	–	–	–	–
	Regression Intercept	–	–	–	–	–	–	–	–	–
COx-a_L	Bias	0 [0 – 0]	0 [0 – 0]	0 [0 – 0]	0 [0 – 0]	0 [0 – 0]	0 [0 – 0]	-0.01 [-0.01 – -0.01]	–	–
	Lower LoA	-0.21 [-0.24 – -0.17]	-0.16 [-0.19 – -0.15]	-0.15 [-0.18 – -0.14]	-0.14 [-0.15 – -0.13]	-0.14 [-0.15 – -0.12]	-0.14 [-0.16 – -0.13]	-0.15 [-0.15 – -0.15]	–	–
	Upper LoA	0.07 [-0.22 – 0.44]	-0.01 [-0.28 – 0.43]	0.11 [-0.09 – 0.44]	0.15 [-0.04 – 0.35]	0.15 [-0.3 – 0.43]	0.31 [-0.23 – 0.77]	-1.94 [-1.94 – -1.94]	–	–
	LoA Spread	0.4 [0.34 – 0.48]	0.32 [0.29 – 0.37]	0.3 [0.28 – 0.35]	0.28 [0.26 – 0.31]	0.27 [0.25 – 0.29]	0.28 [0.25 – 0.3]	0.28 [0.28 – 0.28]	–	–
	Relative Bias	0.2 [0.17 – 0.24]	0.16 [0.15 – 0.19]	0.15 [0.14 – 0.18]	0.14 [0.13 – 0.16]	0.14 [0.12 – 0.15]	0.14 [0.12 – 0.15]	0.14 [0.14 – 0.14]	–	–
	Regression Slope	0.01 [0 – 0.01]	0 [0 – 0.01]	0 [0 – 0.01]	0 [0 – 0]	0 [0 – 0]	0 [0 – 0]	0 [0 – 0]	–	–
	Regression Intercept	-0.03 [-0.04 – -0.02]	-0.02 [-0.02 – -0.02]	-0.01 [-0.02 – -0.01]	-0.01 [-0.01 – 0]	0 [-0.01 – 0]	0.01 [-0.01 – 0.01]	0.02 [0.02 – 0.02]	–	–
COx-a_R	Bias	0 [0 – 0]	0 [0 – 0]	0 [0 – 0]	0 [0 – 0]	0 [0 – 0]	0 [0 – 0]	0 [-0.01 – 0]	–	–
	Lower LoA	-0.19 [-0.24 – -0.18]	-0.16 [-0.19 – -0.15]	-0.16 [-0.19 – -0.14]	-0.14 [-0.17 – -0.13]	-0.14 [-0.15 – -0.12]	-0.13 [-0.15 – -0.11]	-0.16 [-0.17 – -0.15]	–	–
	Upper LoA	0.06 [-0.35 – 0.6]	0.19 [-0.16 – 0.51]	0.11 [-0.17 – 0.42]	0.11 [-0.19 – 0.27]	0.04 [-0.15 – 0.26]	0.13 [-0.52 – 0.72]	-0.88 [-1.64 – -0.12]	–	–
	LoA Spread	0.39 [0.35 – 0.49]	0.32 [0.3 – 0.38]	0.31 [0.28 – 0.38]	0.28 [0.26 – 0.34]	0.27 [0.24 – 0.3]	0.26 [0.23 – 0.31]	0.32 [0.31 – 0.33]	–	–
	Relative Bias	0.2 [0.18 – 0.24]	0.16 [0.15 – 0.19]	0.16 [0.14 – 0.19]	0.14 [0.13 – 0.17]	0.14 [0.12 – 0.15]	0.13 [0.11 – 0.15]	0.16 [0.15 – 0.16]	–	–
	Regression Slope	0.01 [0 – 0.01]	0 [0 – 0]	0 [0 – 0]	0 [0 – 0]	0 [0 – 0]	0 [0 – 0]	-0.01 [-0.01 – 0]	–	–
	Regression Intercept	-0.02 [-0.03 – -0.02]	-0.01 [-0.02 – -0.01]	-0.01 [-0.02 – -0.01]	-0.01 [-0.01 – 0]	0 [0 – 0.01]	0.01 [0 – 0.02]	0.02 [0.02 – 0.02]	–	–
**TBI Population**										
rSO$_2$_L	Bias	0.01 [0 – 0.02]	0 [-0.01 – 0.02]	0 [-0.69 – 0.03]	0 [-319.87 – 118.86]	0 [-4.07E+13 – 0.06]	0 [-2749.68 – 0.01]	0 [0 – 0.01]	0 [0 – 0]	-0.01 [-0.01 – 0]
	Lower LoA	-3.36 [-6.59 – -1.86]	-4.81 [-28.97 – -1.89]	-124.56 [-8.11E+06 – -1.83]	-71773.65 [-6.97E+20 – -1.74]	-2.36E+05 [-1.85E+36 – -1.36]	-9.98 [-2.06E+33 – -1.34]	-1.29 [-4859.86 – -1.11]	-1.47 [-3.64 – -1.12]	-2.57 [-3 – -1.98]
	Upper LoA	3.35 [1.84 – 6.67]	4.86 [1.91 – 29.27]	124.22 [1.83 – 8.06E+06]	71488.76 [1.75 – 6.91E+20]	2.38E+05 [1.36 – 1.84E+36]	10.1 [1.34 – 2.05E+33]	1.3 [1.12 – 4930.55]	1.48 [1.11 – 3.66]	2.55 [1.95 – 3]
	LoA Spread	6.72 [3.71 – 13.26]	9.67 [3.81 – 58.25]	248.78 [3.66 – 1.62E+07]	1.43E+05 [3.49 – 1.39E+21]	4.75E+05 [2.73 – 3.68E+36]	20.08 [2.68 – 4.12E+33]	2.6 [2.23 – 9790.41]	2.95 [2.23 – 7.3]	5.12 [3.93 – 6.01]
	Relative Bias	0.09 [-0.09 – 0.24]	0.05 [-0.09 – 0.17]	0.05 [-0.12 – 0.2]	0.09 [-0.12 – 0.17]	0.04 [-0.11 – 0.16]	0 [-0.11 – 0.13]	0.1 [-0.17 – 0.18]	-0.02 [-0.05 – 0.11]	-0.12 [-0.18 – -0.1]

	Regression Slope	-0.02 [-0.1 – -0.01]	-0.04 [-1.38 – 0]	-1.55 [-2 – 0]	-2 [-2 – 0]	-2 [-2 – 0]	-0.39 [-2 – 0]	0 [-2 – 0.01]	0 [0 – 0.06]	0.03 [0 – 0.06]
	Regression Intercept	1.2 [0.36 – 7.03]	2.7 [0.17 – 95.5]	40.5 [0.09 – 137.5]	1.53 [-0.16 – 147.5]	0.09 [-0.25 – 487750]	-0.17 [-3.65 – 35.93]	-0.1 [-0.67 – 0.03]	-0.15 [-4.2 – 0.18]	-2.1 [-3.7 – -0.04]
rSO_2_R	Bias	0 [0 – 0.02]	0.01 [-0.01 – 0.04]	0 [-0.02 – 0.26]	0 [-7279.62 – 4.43E+05]	0 [-1.03E+06 – 14023.44]	0 [-554.01 – 5.12E+12]	0 [0 – 0]	0 [0 – 0.01]	0 [0 – 0]
	Lower LoA	-3.08 [-5.61 – -1.82]	-6.3 [-29.22 – -2.49]	-55.85 [-3.36E+06 – -2.07]	-2.60E+06 [-6.11E+16 – -2.55]	-2.43E+08 [-9.13E+26 – -1.49]	-2.62E+11 [-5.23E+40 – -1.35]	-1.11 [-1.48 – -0.82]	-1.02 [-2.19 – -0.81]	-1.03 [-6.58 – -0.93]
	Upper LoA	3.1 [1.82 – 5.62]	6.28 [2.51 – 29.46]	55.71 [2.08 – 3.39E+06]	2.58E+06 [2.58 – 6.10E+16]	2.42E+08 [1.49 – 9.10E+26]	2.59E+11 [1.34 – 5.22E+40]	1.11 [0.83 – 1.5]	1.02 [0.82 – 2.2]	1.03 [0.93 – 6.58]
	LoA Spread	6.18 [3.63 – 11.22]	12.6 [5.01 – 58.68]	111.56 [4.16 – 6.75E+06]	5.19E+06 [5.13 – 1.22E+17]	4.85E+08 [2.98 – 1.82E+27]	5.21E+11 [2.69 – 1.05E+41]	2.22 [1.65 – 2.98]	2.04 [1.63 – 4.39]	2.06 [1.86 – 13.16]
	Relative Bias	0.08 [-0.07 – 0.19]	0.11 [-0.12 – 0.22]	0.1 [-0.12 – 0.18]	0.05 [-0.15 – 0.16]	0.04 [-0.13 – 0.14]	0.02 [-0.12 – 0.15]	0.08 [-0.03 – 0.15]	0 [-0.11 – 0.17]	-0.04 [-0.06 – -0.02]
	Regression Slope	-0.02 [-0.06 – -0.01]	-0.09 [-1.35 – -0.01]	-1.45 [-2 – 0]	-2 [-2 – 0]	-2 [-2 – 0]	-2 [-2 – 0]	0 [0 – 0.01]	0 [0 – 0.02]	0 [0 – 0.02]
	Regression Intercept	1.25 [0.46 – 4.53]	7.4 [0.52 – 92.25]	16.5 [0.07 – 140]	71.5 [-0.18 – 140]	0.03 [-0.73 – 160]	-0.03 [-1.1 – 167.5]	-0.16 [-0.55 – -0.04]	-0.33 [-1.33 – -0.09]	-0.26 [-0.92 – -0.18]
COx_L	Bias	0 [0 – 0]	0 [0 – 0]	0 [0 – 0]	0 [0 – 0]	0 [0 – 0]	0 [0 – 0]	0 [0 – 0]	0 [0 – 0]	0 [0 – 0]
	Lower LoA	-0.2 [-0.22 – -0.19]	-0.16 [-0.18 – -0.15]	-0.15 [-0.17 – -0.14]	-0.14 [-0.15 – -0.13]	-0.14 [-0.15 – -0.13]	-0.13 [-0.15 – -0.13]	-0.13 [-0.14 – -0.13]	-0.13 [-0.14 – -0.13]	-0.13 [-0.13 – -0.12]
	Upper LoA	0.2 [0.19 – 0.22]	0.17 [0.15 – 0.18]	0.15 [0.14 – 0.17]	0.14 [0.13 – 0.15]	0.14 [0.13 – 0.15]	0.13 [0.13 – 0.15]	0.13 [0.13 – 0.14]	0.13 [0.13 – 0.15]	0.13 [0.12 – 0.13]
	LoA Spread	0.4 [0.37 – 0.44]	0.33 [0.31 – 0.36]	0.31 [0.29 – 0.33]	0.29 [0.27 – 0.31]	0.28 [0.26 – 0.3]	0.27 [0.25 – 0.29]	0.26 [0.26 – 0.29]	0.26 [0.25 – 0.29]	0.25 [0.25 – 0.26]
	Relative Bias	0.1 [-0.11 – 0.31]	0.07 [-0.02 – 0.19]	0.04 [-0.03 – 0.15]	0.02 [-0.04 – 0.08]	-0.01 [-0.06 – 0.07]	0 [-0.05 – 0.05]	0.05 [-0.01 – 0.15]	0.06 [-0.09 – 0.2]	0.02 [-0.2 – 0.1]
	Regression Slope	-0.03 [-0.04 – -0.02]	-0.01 [-0.02 – -0.01]	-0.01 [-0.01 – -0.01]	0 [0 – 0]	0 [0 – 0]	0.01 [0 – 0.01]	0.02 [0.01 – 0.02]	0.02 [0.01 – 0.03]	0.02 [0.02 – 0.03]
	Regression Intercept	0 [0 – 0]	0 [0 – 0]	0 [0 – 0]	0 [0 – 0]	0 [0 – 0]	0 [0 – 0]	0 [0 – 0]	0 [0 – 0]	0 [0 – 0]
COx_R	Bias	0 [0 – 0]	0 [0 – 0]	0 [0 – 0]	0 [0 – 0]	0 [0 – 0]	0 [0 – 0]	0 [0 – 0]	0 [0 – 0]	0 [0 – 0]
	Lower LoA	-0.21 [-0.23 – -0.19]	-0.17 [-0.18 – -0.15]	-0.16 [-0.17 – -0.14]	-0.15 [-0.16 – -0.13]	-0.14 [-0.15 – -0.13]	-0.13 [-0.14 – -0.13]	-0.15 [-0.16 – -0.14]	-0.15 [-0.16 – -0.13]	-0.13 [-0.13 – -0.13]
	Upper LoA	0.21 [0.19 – 0.23]	0.17 [0.15 – 0.18]	0.16 [0.14 – 0.18]	0.15 [0.13 – 0.16]	0.14 [0.13 – 0.15]	0.13 [0.13 – 0.15]	0.15 [0.14 – 0.16]	0.15 [0.13 – 0.15]	0.13 [0.13 – 0.13]
	LoA Spread	0.42 [0.38 – 0.47]	0.33 [0.3 – 0.36]	0.32 [0.29 – 0.35]	0.3 [0.27 – 0.33]	0.28 [0.26 – 0.3]	0.27 [0.25 – 0.29]	0.29 [0.27 – 0.32]	0.3 [0.26 – 0.31]	0.26 [0.26 – 0.26]
	Relative Bias	0.13 [-0.06 – 0.27]	0.01 [-0.09 – 0.14]	0.03 [-0.05 – 0.12]	0.01 [-0.06 – 0.09]	0.01 [-0.04 – 0.06]	0 [-0.06 – 0.07]	0.04 [-0.03 – 0.12]	-0.21 [-0.38 – 0.14]	-0.22 [-0.3 – -0.14]
	Regression Slope	-0.03 [-0.04 – -0.02]	-0.01 [-0.02 – -0.01]	-0.01 [-0.01 – -0.01]	0 [0 – 0]	0 [0 – 0]	0.01 [0 – 0.01]	0.01 [0 – 0.02]	0.02 [0.01 – 0.02]	0.02 [0.02 – 0.02]
	Regression Intercept	0 [0 – 0]	0 [0 – 0]	0 [0 – 0]	0 [0 – 0]	0 [0 – 0]	0 [0 – 0]	0 [0 – 0]	0 [0 – 0]	0 [0 – 0]
COx-a_L	Bias	0.17 [-0.03 – 0.26]	0.09 [0 – 0.19]	0.08 [0.01 – 0.13]	0.05 [0 – 0.1]	0.01 [-0.03 – 0.06]	0.01 [-0.03 – 0.04]	-0.03 [-0.11 – 0.05]	-0.04 [-0.13 – 0.25]	-0.01 [-0.37 – 0.15]
	Lower LoA	0.4 [0.37 – 0.44]	0.32 [0.3 – 0.36]	0.31 [0.29 – 0.34]	0.29 [0.28 – 0.32]	0.28 [0.26 – 0.3]	0.27 [0.25 – 0.29]	0.27 [0.26 – 0.29]	0.26 [0.25 – 0.28]	0.26 [0.26 – 0.26]
	Upper LoA	-0.2 [-0.22 – -0.18]	-0.16 [-0.18 – -0.15]	-0.16 [-0.17 – -0.14]	-0.14 [-0.16 – -0.14]	-0.14 [-0.15 – -0.13]	-0.13 [-0.15 – -0.13]	-0.14 [-0.14 – -0.13]	-0.13 [-0.14 – -0.13]	-0.13 [-0.13 – -0.13]
	LoA Spread	0.2 [0.18 – 0.22]	0.16 [0.15 – 0.18]	0.16 [0.15 – 0.17]	0.15 [0.14 – 0.16]	0.14 [0.13 – 0.15]	0.13 [0.13 – 0.15]	0.14 [0.13 – 0.14]	0.13 [0.13 – 0.14]	0.13 [0.13 – 0.13]
	Relative Bias	0 [0 – 0.01]	0 [0 – 0]	0 [0 – 0]	0 [0 – 0]	0 [0 – 0]	0 [0 – 0]	0 [0 – 0]	0 [0 – 0]	0 [0 – 0]
	Regression Slope	-0.03 [-0.04 – -0.02]	-0.01 [-0.02 – -0.01]	-0.01 [-0.01 – -0.01]	0 [0 – 0]	0 [0 – 0.01]	0.01 [0 – 0.01]	0.01 [0.01 – 0.02]	0.01 [0.01 – 0.02]	0.02 [0.02 – 0.03]
	Regression Intercept	0 [0 – 0]	0 [0 – 0]	0 [0 – 0]	0 [0 – 0]	0 [0 – 0]	0 [0 – 0]	0 [0 – 0]	0 [0 – 0]	0 [0 – 0]
COx-a_R	Bias	0.14 [-0.03 – 0.3]	0.1 [-0.03 – 0.2]	0.06 [-0.04 – 0.14]	0.04 [-0.03 – 0.08]	0.01 [-0.02 – 0.07]	0 [-0.04 – 0.05]	0.01 [-0.11 – 0.25]	0.15 [-0.04 – 0.22]	-0.4 [-0.44 – -0.19]
	Lower LoA	0.4 [0.37 – 0.47]	0.33 [0.3 – 0.36]	0.31 [0.29 – 0.34]	0.29 [0.27 – 0.32]	0.28 [0.26 – 0.3]	0.27 [0.26 – 0.29]	0.29 [0.28 – 0.31]	0.29 [0.27 – 0.31]	0.29 [0.27 – 0.29]
	Upper LoA	-0.2 [-0.24 – -0.18]	-0.17 [-0.18 – -0.15]	-0.16 [-0.17 – -0.14]	-0.15 [-0.16 – -0.14]	-0.14 [-0.15 – -0.13]	-0.14 [-0.14 – -0.13]	-0.15 [-0.15 – -0.14]	-0.14 [-0.15 – -0.13]	-0.14 [-0.15 – -0.13]
	LoA Spread	0.2 [0.18 – 0.24]	0.17 [0.15 – 0.18]	0.16 [0.14 – 0.17]	0.15 [0.14 – 0.16]	0.14 [0.13 – 0.15]	0.14 [0.13 – 0.14]	0.15 [0.14 – 0.15]	0.15 [0.13 – 0.15]	0.14 [0.13 – 0.15]
	Relative Bias	0 [0 – 0.01]	0 [0 – 0]	0 [0 – 0]	0 [0 – 0]	0 [0 – 0]	0 [0 – 0]	0 [0 – 0]	0 [0 – 0]	0 [0 – 0]
	Regression Slope	-0.03 [-0.04 – -0.02]	-0.01 [-0.02 – -0.01]	-0.01 [-0.01 – -0.01]	0 [0 – 0]	0 [0 – 0.01]	0.01 [0 – 0.01]	0.01 [0.01 – 0.01]	0.01 [0.01 – 0.02]	0.02 [0.02 – 0.02]
	Regression Intercept	0 [0 – 0]	0 [0 – 0]	0 [0 – 0]	0 [0 – 0]	0 [0 – 0]	0 [0 – 0]	0 [0 – 0]	0 [0 – 0]	0 [0 – 0]

COx, cerebral oximetry index with cerebral perfusion pressure; COx-a, cerebral oximetry index with arterial blood pressure; HC, healthy control volunteer group; IQR, interquartile range; r-value, Pearson correlation coefficient; rSO_2, regional cerebral oxygen saturation; SP, elective spinal surgery patient group; TBI, traumatic brain injury patient group.

File S8d: Windowed-Interval – Bland-Altman Analysis of rSO_2 and COx/COx-a in All Populations using 10-Second Temporal Resolution

Physiologic Variable	Value	Median [IQR]								
		5-Minute Interval	10-Minute Interval	15-Minute Interval	30-Minute Interval	1-Hour Interval	2-Hour Interval	6-Hour Interval	12-Hour Interval	1-Day Interval
**HC Population**										
rSO_2_L	Bias	0.03 [-0.23 – 0.29]	0.09 [-0.41 – 0.5]	0.2 [-0.81 – 0.79]	–	–	–	–	–	–
	Lower LoA	-2.95 [-3.79 – -2.31]	-2.81 [-3.52 – -1.96]	-2.48 [-3.56 – -1.52]	–	–	–	–	–	–
	Upper LoA	2.95 [2.21 – 4.17]	3 [2.02 – 3.94]	2.44 [1.63 – 3.87]	–	–	–	–	–	–
	LoA Spread	5.91 [4.68 – 8.12]	5.52 [4.38 – 7.55]	4.97 [4.1 – 6.15]	–	–	–	–	–	–
	Relative Bias	0.54 [-3.92 – 4.99]	1.52 [-7.75 – 9.41]	2.66 [-15.69 – 15.12]	–	–	–	–	–	–
	Regression Slope	0.25 [-0.11 – 0.62]	0.99 [0.21 – 1.6]	1.5 [0.93 – 1.9]	–	–	–	–	–	–
	Regression Intercept	-17.5 [-44.25 – 7.88]	-69.5 [-120 – -14.75]	-105 [-147.5 – -66.5]	–	–	–	–	–	–
rSO_2_R	Bias	-0.06 [-0.41 – 0.27]	0.05 [-0.4 – 0.68]	-0.02 [-0.83 – 0.72]	–	–	–	–	–	–
	Lower LoA	-3.23 [-4.3 – -2.39]	-2.93 [-3.9 – -2.02]	-2.53 [-3.73 – -1.98]	–	–	–	–	–	–
	Upper LoA	3.25 [2.45 – 4.27]	3.12 [1.96 – 4.28]	2.64 [1.59 – 3.93]	–	–	–	–	–	–
	LoA Spread	6.41 [5.15 – 8.16]	5.81 [4.55 – 7.36]	5.52 [4.3 – 6.89]	–	–	–	–	–	–
	Relative Bias	-0.95 [-5.8 – 3.84]	0.75 [-9.25 – 10.16]	-0.21 [-18.81 – 11.77]	–	–	–	–	–	–
	Regression Slope	0.18 [-0.15 – 0.53]	0.94 [0.2 – 1.5]	1.4 [0.87 – 1.8]	–	–	–	–	–	–
	Regression Intercept	-11 [-38.5 – 10.75]	-67 [-110 – -14.5]	-100 [-130 – -61.75]	–	–	–	–	–	–
COx_L	Bias	–	–	–	–	–	–	–	–	–
	Lower LoA	–	–	–	–	–	–	–	–	–
	Upper LoA	–	–	–	–	–	–	–	–	–
	LoA Spread	–	–	–	–	–	–	–	–	–
	Relative Bias	–	–	–	–	–	–	–	–	–
	Regression Slope	–	–	–	–	–	–	–	–	–
	Regression Intercept	–	–	–	–	–	–	–	–	–
COx_R	Bias	–	–	–	–	–	–	–	–	–
	Lower LoA	–	–	–	–	–	–	–	–	–
	Upper LoA	–	–	–	–	–	–	–	–	–
	LoA Spread	–	–	–	–	–	–	–	–	–
	Relative Bias	–	–	–	–	–	–	–	–	–
	Regression Slope	–	–	–	–	–	–	–	–	–
	Regression Intercept	–	–	–	–	–	–	–	–	–
COx-a_L	Bias	0 [-0.07 – 0.07]	0.02 [-0.16 – 0.12]	-0.07 [-0.25 – 0.12]	–	–	–	–	–	–
	Lower LoA	-0.59 [-0.78 – -0.44]	-0.53 [-0.73 – -0.35]	-0.49 [-0.79 – -0.35]	–	–	–	–	–	–
	Upper LoA	-0.13 [-5.5 – 5.61]	2.19 [-16.59 – 11.23]	-8.41 [-22.91 – 16.07]	–	–	–	–	–	–
	LoA Spread	1.17 [0.91 – 1.46]	0.98 [0.79 – 1.35]	0.94 [0.76 – 1.19]	–	–	–	–	–	–
	Relative Bias	0.56 [0.44 – 0.76]	0.49 [0.33 – 0.7]	0.4 [0.22 – 0.63]	–	–	–	–	–	–
	Regression Slope	-0.01 [-0.08 – 0.1]	-0.11 [-0.38 – 0.12]	-0.18 [-0.66 – 0.24]	–	–	–	–	–	–
	Regression Intercept	-0.03 [-0.26 – 0.35]	1.1 [0.12 – 1.73]	2 [1.23 – 2]	–	–	–	–	–	–
COx-a_R	Bias	-0.01 [-0.06 – 0.06]	-0.03 [-0.16 – 0.13]	-0.03 [-0.26 – 0.1]	–	–	–	–	–	–
	Lower LoA	-0.63 [-0.83 – -0.49]	-0.56 [-0.74 – -0.38]	-0.58 [-0.73 – -0.4]	–	–	–	–	–	–
	Upper LoA	-0.73 [-5.66 – 4.41]	-3.09 [-15.16 – 10.62]	-2.53 [-27.99 – 7.6]	–	–	–	–	–	–
	LoA Spread	1.27 [0.95 – 1.66]	1.08 [0.82 – 1.31]	0.98 [0.75 – 1.2]	–	–	–	–	–	–
	Relative Bias	0.65 [0.44 – 0.86]	0.49 [0.33 – 0.7]	0.45 [0.19 – 0.68]	–	–	–	–	–	–
	Regression Slope	-0.01 [-0.09 – 0.09]	-0.07 [-0.33 – 0.11]	-0.14 [-0.68 – 0.11]	–	–	–	–	–	–
	Regression Intercept	-0.09 [-0.53 – 0.23]	0.73 [0.09 – 1.5]	2 [1.13 – 2]	–	–	–	–	–	–
**SP Population**										
rSO_2_L	Bias	-0.09 [-0.3 – 0.17]	0.08 [-0.67 – 0.45]	0.45 [-0.11 – 0.93]	0.18 [-0.41 – 1.57]	0.43 [-1.19 – 1.76]	-1.41 [-1.65 – -0.45]	–	–	–
	Lower LoA	-11.92 [-169.91 – -4.38]	-7.03 [-23.72 – -4.09]	-5.84 [-10.97 – -4.4]	-7.37 [-9.33 – -4.15]	-6.39 [-10.47 – -3.49]	-13.04 [-19.76 – -9.03]	–	–	–
	Upper LoA	11.25 [4.92 – 166.44]	7.55 [4.57 – 24.49]	7.78 [4.58 – 11.19]	7.44 [5.73 – 11.19]	7.34 [4.58 – 9.67]	9.25 [7.64 – 16.46]	–	–	–
	LoA Spread	23.25 [9.51 – 336.35]	14.58 [8.76 – 48.21]	13.49 [9.11 – 21.23]	14.48 [10.58 – 19.01]	12.58 [9.44 – 19.73]	22.29 [16.67 – 36.22]	–	–	–

	Relative Bias	-0.48 [-1.49 – 0.75]	0.47 [-2.08 – 2.59]	3.76 [-1.11 – 4.84]	1.79 [-2.85 – 7.88]	2.62 [-5.05 – 13.96]	-2.8 [-5.66 – 0.9]	–	–	–
	Regression Slope	-0.12 [-1.95 – 0.01]	-0.14 [-0.78 – 0]	0 [-0.31 – 0.19]	0.17 [-0.14 – 0.99]	1.55 [0.65 – 2]	1.1 [0.86 – 1.5]	–	–	–
	Regression Intercept	8.7 [-0.3 – 110]	9.1 [0.39 – 44.5]	-0.25 [-9.6 – 17.5]	-12 [-65.5 – 10.15]	-96.5 [-127.5 – -37.25]	-91 [-115.5 – -65]	–	–	–
rSO_2_R	Bias	0.02 [-0.2 – 0.33]	0.01 [-0.45 – 0.56]	0.05 [-0.28 – 0.67]	0.15 [-0.87 – 1.2]	0.2 [-0.74 – 1.17]	-1.22 [-3.27 – -0.19]	–	–	–
	Lower LoA	-8.35 [-38.71 – -3.92]	-6.28 [-10.43 – -4.34]	-6.03 [-9.54 – -4.46]	-7.42 [-9.41 – -5.01]	-5.92 [-8.89 – -3.23]	-21.44 [-24.7 – -12.16]	–	–	–
	Upper LoA	6.15 [4.18 – 38.49]	5.63 [4.32 – 8.73]	6.41 [5.19 – 9.66]	7.39 [6.27 – 11.26]	6.64 [4.81 – 10.16]	10.8 [7.67 – 18.16]	–	–	–
	LoA Spread	14.5 [8.14 – 77.2]	10.11 [9.05 – 20.8]	13.51 [9.71 – 19.5]	14.8 [11.88 – 19.68]	13.33 [8.76 – 18.08]	32.24 [19.83 – 42.86]	–	–	–
	Relative Bias	0.09 [-0.6 – 1.41]	0.12 [-3.88 – 4.43]	0.69 [-1.49 – 3.42]	0.87 [-3.18 – 9.49]	1.15 [-3.4 – 14.24]	-2.28 [-9.39 – 4.47]	–	–	–
	Regression Slope	-0.1 [-1.55 – 0.02]	-0.03 [-0.1 – 0.03]	-0.02 [-0.23 – 0.1]	-0.2 [-0.36 – 0.78]	1.7 [0.59 – 2]	2 [1.27 – 2]	–	–	–
	Regression Intercept	8.3 [-1.3 – 97]	2.2 [-2.1 – 6.95]	2.3 [-6.8 – 17]	8.6 [-38 – 23.5]	-105 [-137.5 – -36.25]	-150 [-150 – -98]	–	–	–
COx_L	Bias	–	–	–	–	–	–	–	–	–
	Lower LoA	–	–	–	–	–	–	–	–	–
	Upper LoA	–	–	–	–	–	–	–	–	–
	LoA Spread	–	–	–	–	–	–	–	–	–
	Relative Bias	–	–	–	–	–	–	–	–	–
	Regression Slope	–	–	–	–	–	–	–	–	–
	Regression Intercept	–	–	–	–	–	–	–	–	–
COx_R	Bias	–	–	–	–	–	–	–	–	–
	Lower LoA	–	–	–	–	–	–	–	–	–
	Upper LoA	–	–	–	–	–	–	–	–	–
	LoA Spread	–	–	–	–	–	–	–	–	–
	Relative Bias	–	–	–	–	–	–	–	–	–
	Regression Slope	–	–	–	–	–	–	–	–	–
	Regression Intercept	–	–	–	–	–	–	–	–	–
COx-a_L	Bias	0.01 [-0.03 – 0.05]	0 [-0.07 – 0.05]	0.01 [-0.1 – 0.09]	0.04 [-0.07 – 0.17]	0.03 [-0.04 – 0.19]	0.04 [-0.03 – 0.05]	–	–	–
	Lower LoA	-1.11 [-1.45 – -0.93]	-1.13 [-1.33 – -0.91]	-1.05 [-1.26 – -0.89]	-0.86 [-1.1 – -0.68]	-0.7 [-0.93 – -0.54]	-0.67 [-0.74 – -0.61]	–	–	–
	Upper LoA	0.23 [-1.37 – 1.55]	-0.05 [-2.43 – 2.61]	0.45 [-6.33 – 3.85]	2.44 [-3.75 – 8.24]	1.54 [-2.02 – 13]	3.51 [-1.98 – 3.53]	–	–	–
	LoA Spread	2.21 [1.85 – 2.87]	2.26 [1.8 – 2.77]	2.12 [1.71 – 2.55]	1.71 [1.43 – 2.12]	1.57 [1.38 – 1.82]	1.41 [1.3 – 1.43]	–	–	–
	Relative Bias	1.15 [0.95 – 1.43]	1.11 [0.88 – 1.52]	0.99 [0.87 – 1.32]	0.93 [0.78 – 1.11]	0.87 [0.67 – 0.99]	0.64 [0.62 – 0.71]	–	–	–
	Regression Slope	0.06 [-0.01 – 0.21]	0.05 [-0.04 – 0.15]	-0.02 [-0.19 – 0.08]	-0.08 [-0.29 – 0.22]	-0.15 [-0.47 – 0.08]	-0.34 [-0.52 – -0.29]	–	–	–
	Regression Intercept	-0.33 [-0.9 – -0.18]	-0.08 [-0.74 – 0.11]	0.16 [-0.12 – 0.5]	0.81 [0.1 – 1.2]	1.75 [1.4 – 1.9]	1.9 [1.6 – 1.95]	–	–	–
COx-a_R	Bias	0 [-0.05 – 0.08]	-0.01 [-0.13 – 0.04]	-0.03 [-0.09 – 0.06]	-0.05 [-0.1 – 0.04]	0.03 [-0.07 – 0.11]	0.09 [0.06 – 0.09]	–	–	–
	Lower LoA	-1.33 [-2.68 – -0.98]	-1.13 [-1.61 – -0.94]	-1.16 [-1.26 – -0.99]	-0.99 [-1.07 – -0.84]	-0.83 [-0.91 – -0.61]	-0.8 [-0.8 – -0.69]	–	–	–
	Upper LoA	0.16 [-1.8 – 1.64]	-0.62 [-4.24 – 1.85]	-1.56 [-4.8 – 2.3]	-3.16 [-6.58 – 1.96]	1.72 [-4.39 – 8.25]	5.23 [3.66 – 6.08]	–	–	–
	LoA Spread	2.54 [1.94 – 5.34]	2.29 [1.79 – 3.17]	2.23 [1.85 – 2.71]	1.87 [1.69 – 2.14]	1.63 [1.44 – 1.84]	1.68 [1.51 – 1.73]	–	–	–
	Relative Bias	1.24 [0.98 – 2.71]	1.25 [0.86 – 1.51]	1.07 [0.95 – 1.29]	0.89 [0.75 – 1.18]	0.86 [0.65 – 0.98]	0.88 [0.82 – 0.93]	–	–	–
	Regression Slope	0.07 [0 – 0.27]	0.02 [-0.01 – 0.1]	-0.06 [-0.13 – 0.07]	-0.12 [-0.26 – 0.01]	-0.17 [-0.5 – 0.09]	-0.22 [-0.26 – -0.07]	–	–	–
	Regression Intercept	-0.49 [-1.35 – -0.23]	-0.17 [-0.8 – 0.16]	0.02 [-0.31 – 0.44]	0.57 [0.04 – 1.1]	1.45 [0.78 – 1.9]	1.7 [0.91 – 1.75]	–	–	–
**TBI Population**										
rSO_2_L	Bias	0.02 [-3.23 – 20.27]	-0.05 [-8.31 – 0.12]	-0.05 [-0.41 – 0.18]	-0.06 [-0.41 – 0.14]	-0.05 [-0.34 – 0.15]	-0.06 [-0.45 – 0.32]	-0.49 [-1.12 – 0.46]	-0.73 [-1.72 – 0.4]	-0.95 [-3.06 – 1.17]
	Lower LoA	-3603.01 [-184501.48 – -82.12]	-250.94 [-536705.29 – -6.73]	-25.73 [-41157.56 – -5.77]	-11.15 [-125.99 – -6.4]	-7.85 [-12.38 – -5.59]	-8.6 [-13.78 – -5.86]	-9.89 [-14.78 – -6.87]	-10.44 [-16.07 – -7.74]	-11.4 [-20.16 – -8.11]
	Upper LoA	3641.36 [80.43 – 186677.81]	248.73 [6.62 – 535695.1]	25.34 [5.8 – 41001.89]	11.06 [6.48 – 121.36]	7.25 [5.56 – 12.59]	8.14 [6.09 – 13.8]	8.67 [6.74 – 16.03]	9.81 [6.31 – 17.61]	9.68 [6.35 – 15.96]
	LoA Spread	7244.37 [162.55 – 371179.29]	499.67 [13.29 – 1072400.39]	51.07 [11.54 – 82159.44]	22.21 [13 – 247.35]	15.35 [11.27 – 24.79]	16.31 [12.06 – 27.36]	18.3 [14.07 – 29.96]	20.79 [15.18 – 31.86]	21.1 [15.45 – 37.58]
	Relative Bias	0.01 [-0.1 – 0.23]	-0.05 [-0.27 – 0.14]	-0.04 [-0.69 – 0.13]	-0.1 [-1 – 0.47]	-0.19 [-1.38 – 0.81]	-0.41 [-2.15 – 1.74]	-2.25 [-5.59 – 1.31]	-2.6 [-7.32 – 2.56]	-6.86 [-13.25 – 6.55]
	Regression Slope	-2 [-2 – -1.83]	-2 [-2 – -0.09]	-0.95 [-2 – -0.03]	-0.1 [-1.9 – 0.01]	0 [-0.07 – 0.07]	0.03 [-0.03 – 0.16]	0.08 [-0.11 – 0.44]	0.18 [-0.03 – 0.75]	0.96 [0.18 – 1.85]

	Regression Intercept	130 [97.75 – 140]	110 [5.13 – 130]	56.5 [1.2 – 130]	4.3 [-0.99 – 100]	0.28 [-5.15 – 4.33]	-2 [-12 – 1.48]	-5.4 [-28.5 – 7.33]	-12 [-46.5 – 1.5]	-62 [-120 – -13.5]
rSO_2_R	Bias	0.07 [-3.17 – 8.69]	-0.02 [-1.78 – 0.27]	-0.04 [-1.04 – 0.26]	0.02 [-0.37 – 0.15]	0.03 [-0.27 – 0.21]	-0.05 [-0.46 – 0.44]	-0.46 [-1.17 – 0.15]	-0.46 [-1.66 – 0.7]	-1.97 [-4.64 – 0.26]
	Lower LoA	-2012.01 [-34147.33 – -102.56]	-157.65 [-90234.59 – -14.27]	-140.31 [-26507.2 – -7.49]	-10.51 [-79.21 – -4.59]	-7.73 [-18.44 – -4.9]	-8.25 [-15.86 – -5.69]	-10.16 [-16.69 – -6.49]	-10.39 [-19.78 – -7.58]	-12.2 [-21.16 – -8.72]
	Upper LoA	2029.08 [102.58 – 34061.78]	156.13 [14.76 – 88421.91]	145.59 [7.46 – 26425.9]	11.19 [4.99 – 79.72]	7.42 [5.28 – 17.27]	7.94 [6.33 – 15.44]	9.3 [7.17 – 17.11]	11.65 [7.18 – 18.52]	9.04 [6.79 – 17.3]
	LoA Spread	4041.09 [205.3 – 68209.11]	313.77 [29.02 – 178656.5]	285.9 [14.95 – 52933.1]	22.35 [9.67 – 158.93]	14.95 [10.17 – 35.29]	16.4 [12.6 – 31.44]	18.76 [13.5 – 32.1]	21.52 [16.47 – 40.03]	22.07 [16.59 – 35]
	Relative Bias	0.07 [-0.09 – 0.24]	-0.02 [-0.29 – 0.37]	-0.03 [-0.34 – 0.17]	0.02 [-0.47 – 0.7]	0.03 [-1.36 – 1.2]	-0.4 [-2.19 – 2.07]	-2.25 [-5.71 – 1.1]	-2.02 [-6.53 – 2.77]	-7.72 [-16.59 – 0.99]
	Regression Slope	-2 [-2 – -2]	-1.9 [-2 – -0.33]	-1.9 [-2 – -0.11]	-0.07 [-1.75 – 0.04]	0.01 [-0.14 – 0.08]	0.05 [-0.06 – 0.22]	0.13 [-0.04 – 0.44]	0.25 [-0.02 – 0.56]	0.85 [0.29 – 1.5]
	Regression Intercept	140 [120 – 150]	120 [15.75 – 140]	120 [6.65 – 140]	2.45 [-3.03 – 84.25]	-0.95 [-5.75 – 8.15]	-3.2 [-16 – 3.63]	-7.9 [-35.25 – 1.85]	-17.5 [-41.5 – 1]	-63 [-120 – -16]
COx_L	Bias	0 [-0.01 – 0.02]	0 [-0.01 – 0.02]	0 [-0.02 – 0.02]	0 [-0.02 – 0.02]	0 [-0.02 – 0.02]	0.01 [-0.03 – 0.04]	0 [-0.04 – 0.03]	0 [-0.02 – 0.06]	0.01 [-0.02 – 0.06]
	Lower LoA	-1.29 [-6.42 – -0.9]	-1.06 [-2.08 – -0.84]	-1.04 [-1.76 – -0.81]	-0.85 [-0.99 – -0.77]	-0.8 [-0.89 – -0.75]	-0.75 [-0.85 – -0.71]	-0.73 [-0.81 – -0.65]	-0.67 [-0.75 – -0.64]	-0.66 [-0.74 – -0.59]
	Upper LoA	1.3 [0.88 – 6.54]	1.08 [0.85 – 2.08]	1.04 [0.83 – 1.77]	0.88 [0.77 – 0.98]	0.81 [0.73 – 0.91]	0.76 [0.69 – 0.85]	0.71 [0.63 – 0.81]	0.71 [0.63 – 0.81]	0.72 [0.63 – 0.81]
	LoA Spread	2.57 [1.79 – 12.96]	2.14 [1.68 – 4.16]	2.09 [1.63 – 3.57]	1.74 [1.55 – 1.95]	1.63 [1.46 – 1.76]	1.53 [1.4 – 1.67]	1.45 [1.31 – 1.6]	1.39 [1.27 – 1.54]	1.39 [1.26 – 1.52]
	Relative Bias	0.11 [-0.29 – 0.45]	0.1 [-0.57 – 0.72]	-0.05 [-0.51 – 0.91]	0 [-1.03 – 1.07]	0.06 [-1.48 – 1.43]	0.65 [-1.94 – 2.34]	-0.37 [-2.92 – 1.75]	0.06 [-1.59 – 3.9]	0.73 [-1.34 – 4.7]
	Regression Slope	-0.89 [-1.98 – -0.53]	-0.57 [-1.48 – -0.17]	-0.32 [-1.4 – -0.05]	0.09 [-0.26 – 0.24]	0.35 [0.2 – 0.51]	0.66 [0.41 – 0.99]	1.35 [0.84 – 1.8]	1.55 [1.2 – 1.83]	1.8 [1.5 – 2]
	Regression Intercept	0.03 [-0.02 – 0.11]	0.01 [-0.02 – 0.05]	0.01 [-0.01 – 0.05]	0 [-0.02 – 0.03]	-0.01 [-0.04 – 0.03]	-0.02 [-0.06 – 0.04]	-0.04 [-0.12 – 0.05]	-0.02 [-0.11 – 0.05]	-0.02 [-0.18 – 0.12]
COx_R	Bias	0 [-0.01 – 0.03]	0 [-0.02 – 0.02]	0 [-0.01 – 0.02]	-0.01 [-0.03 – 0.01]	0 [-0.02 – 0.03]	0 [-0.02 – 0.03]	0 [-0.04 – 0.05]	0 [-0.04 – 0.03]	0.01 [-0.03 – 0.05]
	Lower LoA	-1.23 [-5.29 – -0.94]	-1.11 [-2.5 – -0.85]	-0.99 [-1.56 – -0.83]	-0.86 [-0.99 – -0.8]	-0.81 [-0.87 – -0.74]	-0.75 [-0.81 – -0.68]	-0.71 [-0.78 – -0.64]	-0.7 [-0.76 – -0.61]	-0.66 [-0.75 – -0.59]
	Upper LoA	1.23 [0.97 – 5.57]	1.11 [0.84 – 2.49]	0.98 [0.85 – 1.59]	0.86 [0.79 – 0.97]	0.81 [0.75 – 0.89]	0.77 [0.69 – 0.83]	0.71 [0.63 – 0.78]	0.67 [0.61 – 0.78]	0.71 [0.61 – 0.77]
	LoA Spread	2.47 [1.89 – 10.83]	2.22 [1.71 – 5.05]	1.96 [1.67 – 3.15]	1.73 [1.59 – 1.93]	1.59 [1.49 – 1.75]	1.48 [1.4 – 1.64]	1.41 [1.31 – 1.53]	1.38 [1.26 – 1.54]	1.38 [1.28 – 1.46]
	Relative Bias	0.07 [-0.37 – 0.75]	-0.06 [-0.74 – 0.53]	0.02 [-0.75 – 0.74]	-0.41 [-1.56 – 0.84]	-0.29 [-1.29 – 1.61]	0.19 [-1.56 – 2.08]	-0.31 [-2.74 – 3.08]	-0.02 [-2.78 – 2.32]	0.52 [-2.15 – 3.67]
	Regression Slope	-0.9 [-1.9 – -0.47]	-0.67 [-1.6 – -0.24]	-0.34 [-1.2 – -0.07]	0.09 [-0.14 – 0.25]	0.34 [0.18 – 0.53]	0.67 [0.44 – 1.03]	1.3 [0.92 – 1.7]	1.6 [1.1 – 1.8]	1.9 [1.6 – 2]
	Regression Intercept	0.01 [-0.01 – 0.04]	0 [-0.01 – 0.02]	0 [-0.01 – 0.02]	0 [-0.02 – 0.02]	0 [-0.02 – 0.03]	-0.01 [-0.03 – 0.02]	-0.01 [-0.03 – 0.03]	0 [-0.02 – 0.04]	0 [-0.02 – 0.05]
COx-a_L	Bias	0.19 [-0.12 – 0.81]	0.19 [-0.54 – 0.59]	0.13 [-0.48 – 0.83]	0.03 [-0.96 – 1.05]	-0.1 [-1.44 – 1.35]	-0.49 [-2.17 – 1.23]	-0.41 [-2.25 – 2.11]	0.3 [-1.42 – 2.3]	0.26 [-1.59 – 3.27]
	Lower LoA	3.72 [1.96 – 27.09]	2.05 [1.71 – 4.34]	1.86 [1.65 – 2.72]	1.67 [1.53 – 1.94]	1.55 [1.45 – 1.72]	1.5 [1.37 – 1.67]	1.37 [1.25 – 1.54]	1.33 [1.22 – 1.52]	1.3 [1.22 – 1.4]
	Upper LoA	-1.86 [-13.55 – -0.97]	-1.01 [-2.14 – -0.86]	-0.92 [-1.37 – -0.81]	-0.83 [-0.97 – -0.77]	-0.77 [-0.85 – -0.72]	-0.77 [-0.82 – -0.7]	-0.7 [-0.79 – -0.61]	-0.65 [-0.74 – -0.61]	-0.63 [-0.68 – -0.58]
	LoA Spread	1.87 [0.99 – 13.54]	1.03 [0.85 – 2.19]	0.93 [0.82 – 1.37]	0.84 [0.77 – 0.97]	0.78 [0.72 – 0.85]	0.73 [0.66 – 0.83]	0.68 [0.61 – 0.76]	0.66 [0.62 – 0.75]	0.66 [0.62 – 0.74]
	Relative Bias	0.08 [0.03 – 0.18]	0.05 [0.01 – 0.11]	0.03 [0 – 0.09]	0 [-0.03 – 0.04]	-0.03 [-0.07 – 0.01]	-0.07 [-0.12 – -0.02]	-0.11 [-0.19 – -0.02]	-0.13 [-0.21 – -0.04]	-0.17 [-0.25 – -0.06]
	Regression Slope	-1.4 [-2 – -0.53]	-0.51 [-1.58 – -0.24]	-0.27 [-1.18 – -0.05]	0.1 [-0.19 – 0.26]	0.41 [0.23 – 0.58]	0.74 [0.44 – 0.97]	1.4 [0.9 – 1.7]	1.7 [1.5 – 1.9]	1.9 [1.7 – 2]
	Regression Intercept	0.01 [0 – 0.04]	0 [-0.01 – 0.02]	0 [-0.02 – 0.02]	0 [-0.02 – 0.02]	0 [-0.02 – 0.02]	-0.01 [-0.03 – 0.02]	0 [-0.04 – 0.05]	0 [-0.02 – 0.03]	0.01 [-0.02 – 0.04]
COx-a_R	Bias	0.3 [-0.11 – 0.78]	0.1 [-0.43 – 0.71]	-0.01 [-0.61 – 0.83]	-0.02 [-1.13 – 1.14]	-0.22 [-1.55 – 1.19]	-0.35 [-2.41 – 1.22]	-0.12 [-2.56 – 3.5]	0.16 [-1.71 – 2.53]	0.68 [-1.99 – 2.89]
	Lower LoA	2.65 [1.99 – 11.44]	2.04 [1.7 – 4.34]	1.96 [1.75 – 3.5]	1.71 [1.55 – 1.9]	1.56 [1.43 – 1.71]	1.46 [1.32 – 1.64]	1.35 [1.23 – 1.5]	1.34 [1.2 – 1.47]	1.3 [1.18 – 1.43]
	Upper LoA	-1.34 [-5.64 – -0.97]	-1.02 [-2.16 – -0.86]	-0.98 [-1.75 – -0.86]	-0.86 [-0.94 – -0.78]	-0.77 [-0.86 – -0.7]	-0.74 [-0.81 – -0.67]	-0.66 [-0.75 – -0.59]	-0.65 [-0.74 – -0.59]	-0.62 [-0.73 – -0.57]
	LoA Spread	1.33 [1 – 5.79]	1.03 [0.85 – 2.18]	0.99 [0.89 – 1.72]	0.85 [0.77 – 0.95]	0.77 [0.71 – 0.86]	0.72 [0.64 – 0.82]	0.7 [0.61 – 0.77]	0.66 [0.6 – 0.73]	0.65 [0.59 – 0.75]
	Relative Bias	0.08 [0.01 – 0.14]	0.04 [0 – 0.1]	0.03 [0 – 0.1]	0 [-0.02 – 0.02]	-0.02 [-0.09 – 0.01]	-0.06 [-0.13 – 0.01]	-0.09 [-0.18 – -0.02]	-0.09 [-0.21 – -0.02]	-0.15 [-0.24 – -0.02]
	Regression Slope	-0.94 [-1.9 – -0.56]	-0.54 [-1.5 – -0.24]	-0.44 [-1.4 – -0.13]	0.08 [-0.13 – 0.22]	0.42 [0.24 – 0.59]	0.75 [0.45 – 0.94]	1.35 [0.79 – 1.7]	1.7 [1.3 – 1.9]	1.9 [1.7 – 2]
	Regression Intercept	0.03 [-0.03 – 0.09]	0.01 [-0.03 – 0.08]	0 [-0.02 – 0.06]	-0.02 [-0.04 – 0.01]	-0.01 [-0.05 – 0.03]	-0.01 [-0.07 – 0.04]	-0.02 [-0.12 – 0.08]	-0.02 [-0.11 – 0.1]	-0.02 [-0.18 – 0.13]

COx, cerebral oximetry index with cerebral perfusion pressure; COx-a, cerebral oximetry index with arterial blood pressure; HC, healthy control volunteer group; IQR, interquartile range; r-value, Pearson correlation coefficient; rSO_2, regional cerebral oxygen saturation; SP, elective spinal surgery patient group; TBI, traumatic brain injury patient group.

File S8e: Anchored-Point – Bland-Altman Analysis of rSO_2 and COx/COx-a in All Populations using 1-Minute and 5-Minute Temporal Resolutions

Physiologic Variable	Value	Median [IQR]		
		HC	SP	TBI
**1-Minute Temporal Resolution**				
rSO_2_L	Bias	0.14 [-0.57 – 0.65]	1.66 [-0.12 – 5.05]	-0.74 [-3.75 – 1.09]
	Lower LoA	-1.43 [-2.3 – -0.55]	-2.77 [-4.97 – -1.48]	-7.93 [-14.13 – -4.35]
	Upper LoA	1.54 [0.91 – 2.57]	7.95 [4.9 – 12.37]	7.23 [3.14 – 13.73]
	LoA Spread	2.85 [2.09 – 4.2]	11.93 [6.84 – 16.9]	15.51 [10.29 – 24.67]
	Relative Bias	7.26 [-16.98 – 24]	19.1 [-0.18 – 37.75]	-5.64 [-20.24 – 8.4]
	Regression Slope	1.8 [1.2 – 2]	2 [2 – 2]	2 [2 – 2]
	Regression Intercept	-130 [-150 – -81.5]	-130 [-150 – -110]	-130 [-150 – -115]
rSO_2_R	Bias	0.2 [-0.64 – 0.68]	2.27 [0.48 – 3.08]	-0.86 [-4.56 – 1.34]
	Lower LoA	-1.55 [-2.4 – -0.8]	-4.33 [-5.89 – -1.91]	-8.19 [-14.72 – -3.37]
	Upper LoA	1.74 [0.81 – 2.88]	8.17 [4.75 – 12.69]	7.1 [3.12 – 12.42]
	LoA Spread	3.27 [2.33 – 4.37]	12.63 [8.94 – 16.12]	16.17 [8.4 – 24.98]
	Relative Bias	6.09 [-17.32 – 20.13]	18.34 [4.61 – 29.18]	-9.74 [-22.65 – 8.2]
	Regression Slope	1.9 [0.97 – 2]	2 [2 – 2]	2 [2 – 2]
	Regression Intercept	-120 [-150 – -65.25]	-130 [-140 – -110]	-140 [-150 – -120]
COx_L	Bias	–	–	0.01 [-0.05 – 0.08]
	Lower LoA	–	–	-0.61 [-0.73 – -0.53]
	Upper LoA	–	–	0.65 [0.55 – 0.75]
	LoA Spread	–	–	1.27 [1.17 – 1.43]
	Relative Bias	–	–	0.44 [-4.15 – 7.01]
	Regression Slope	–	–	2 [2 – 2]
	Regression Intercept	–	–	-0.06 [-0.25 – 0.08]
COx_R	Bias	–	–	-0.01 [-0.08 – 0.07]
	Lower LoA	–	–	-0.61 [-0.72 – -0.54]
	Upper LoA	–	–	0.6 [0.53 – 0.7]
	LoA Spread	–	–	1.25 [1.11 – 1.4]
	Relative Bias	–	–	-1.11 [-5.85 – 5.59]
	Regression Slope	–	–	2 [2 – 2]
	Regression Intercept	–	–	-0.06 [-0.29 – 0.1]
COx-a_L	Bias	0.02 [-0.15 – 0.16]	0.02 [-0.08 – 0.06]	0 [-0.03 – 0.06]
	Lower LoA	-0.28 [-0.56 – -0.15]	-0.76 [-1.02 – -0.52]	-0.6 [-0.68 – -0.52]
	Upper LoA	0.34 [0.18 – 0.5]	0.72 [0.58 – 0.91]	0.61 [0.55 – 0.69]
	LoA Spread	0.68 [0.5 – 0.96]	1.51 [1.18 – 1.83]	1.23 [1.08 – 1.34]
	Relative Bias	3.5 [-20.64 – 23.84]	0.72 [-5.98 – 5]	0.34 [-2.85 – 4.57]
	Regression Slope	1.3 [0.54 – 2]	1.9 [1.7 – 1.95]	2 [2 – 2]
	Regression Intercept	-0.04 [-0.31 – 0.18]	-0.39 [-0.53 – -0.23]	-0.18 [-0.33 – -0.01]
COx-a_R	Bias	0.02 [-0.13 – 0.14]	-0.04 [-0.16 – 0.14]	0 [-0.05 – 0.05]
	Lower LoA	-0.33 [-0.51 – -0.15]	-0.82 [-1 – -0.53]	-0.62 [-0.71 – -0.51]
	Upper LoA	0.3 [0.16 – 0.55]	0.78 [0.57 – 0.88]	0.6 [0.53 – 0.69]
	LoA Spread	0.65 [0.45 – 0.93]	1.46 [1.26 – 1.7]	1.22 [1.08 – 1.32]
	Relative Bias	4.18 [-20.9 – 20.18]	-2.27 [-12.72 – 9.81]	-0.42 [-4.11 – 4.22]
	Regression Slope	1.6 [0.09 – 2]	1.8 [1.6 – 1.9]	2 [2 – 2]

	Regression Intercept	-0.07 [-0.32 – 0.18]	-0.35 [-0.42 – -0.16]	-0.14 [-0.3 – 0]
**5-Minute Temporal Resolution**				
rSO_2_L	Bias	0.01 [-0.9 – 0.95]	1.79 [0.59 – 4.51]	-0.37 [-2.9 – 1.43]
	Lower LoA	-1.83 [-2.87 – -0.24]	-2.6 [-4.76 – -1.46]	-7.54 [-13.06 – -3.28]
	Upper LoA	1.96 [0.68 – 4.03]	7.39 [5.07 – 13.66]	6.26 [3.11 – 14.12]
	LoA Spread	3.79 [2.34 – 5.9]	10.17 [7.97 – 16.47]	14.36 [9.5 – 26.18]
	Relative Bias	11.5 [-22.97 – 41.99]	20.8 [7.42 – 30.57]	-2.51 [-18.97 – 15.4]
	Regression Slope	-1 [-3.3 – 2.4]	2 [1.75 – 2]	2 [2 – 2]
	Regression Intercept	0.71 [-1.9 – 127.5]	-120 [-140 – -79.5]	-130 [-140 – -110]
rSO_2_R	Bias	0.06 [-1.16 – 1.35]	1.45 [-0.58 – 3.77]	-0.95 [-3.15 – 1.32]
	Lower LoA	-1.81 [-3.27 – -0.2]	-3.95 [-6.1 – -2.23]	-7.61 [-13.45 – -3.57]
	Upper LoA	1.72 [0.35 – 4.42]	6.19 [3.31 – 12.46]	7.32 [2.79 – 12.68]
	LoA Spread	3.25 [1.48 – 6.68]	9.85 [7.65 – 18.03]	15.7 [8.48 – 22.97]
	Relative Bias	4.23 [-36.02 – 35.96]	18.78 [-6.84 – 28.19]	-6.54 [-22.69 – 9.18]
	Regression Slope	-0.45 [-2.8 – 1.28]	2 [1.3 – 2]	2 [1.9 – 2]
	Regression Intercept	0.16 [-48.5 – 117.5]	-110 [-140 – -66.5]	-130 [-150 – -120]
COx_L	Bias	–	–	0.02 [-0.05 – 0.07]
	Lower LoA	–	–	-0.5 [-0.6 – -0.43]
	Upper LoA	–	–	0.55 [0.48 – 0.61]
	LoA Spread	–	–	1.05 [0.95 – 1.22]
	Relative Bias	–	–	1.63 [-4.38 – 6.84]
	Regression Slope	–	–	2 [2 – 2]
	Regression Intercept	–	–	-0.06 [-0.23 – 0.06]
COx_R	Bias	–	–	-0.01 [-0.08 – 0.07]
	Lower LoA	–	–	-0.52 [-0.63 – -0.43]
	Upper LoA	–	–	0.52 [0.44 – 0.63]
	LoA Spread	–	–	1.07 [0.93 – 1.18]
	Relative Bias	–	–	-0.97 [-7.27 – 6.58]
	Regression Slope	–	–	2 [2 – 2]
	Regression Intercept	–	–	-0.07 [-0.24 – 0.09]
COx-a_L	Bias	-0.03 [-0.24 – 0.26]	0.02 [-0.07 – 0.12]	0.01 [-0.04 – 0.05]
	Lower LoA	-0.51 [-0.85 – -0.24]	-0.62 [-1 – -0.36]	-0.5 [-0.56 – -0.44]
	Upper LoA	0.41 [0.07 – 0.8]	0.63 [0.49 – 0.94]	0.51 [0.45 – 0.57]
	LoA Spread	0.92 [0.42 – 1.69]	1.28 [0.91 – 1.8]	1.03 [0.9 – 1.12]
	Relative Bias	-7.64 [-42.21 – 23.23]	3.15 [-4.93 – 12.13]	0.9 [-4.22 – 5.04]
	Regression Slope	-0.78 [-2.6 – 2.2]	1.9 [1.7 – 2]	2 [2 – 2]
	Regression Intercept	-0.03 [-0.34 – 0.37]	-0.27 [-0.48 – -0.04]	-0.19 [-0.31 – -0.04]
COx-a_R	Bias	0 [-0.21 – 0.26]	-0.09 [-0.14 – 0.1]	0 [-0.05 – 0.06]
	Lower LoA	-0.4 [-0.83 – -0.1]	-0.77 [-0.97 – -0.52]	-0.5 [-0.58 – -0.43]
	Upper LoA	0.54 [0.11 – 0.75]	0.7 [0.52 – 0.79]	0.49 [0.43 – 0.58]
	LoA Spread	0.79 [0.36 – 1.31]	1.42 [1.2 – 1.71]	1.01 [0.88 – 1.13]
	Relative Bias	2.48 [-27.6 – 34.1]	-6.14 [-11.62 – 8.9]	-0.57 [-5.6 – 5.66]
	Regression Slope	0.12 [-1.7 – 1.8]	1.9 [0.97 – 2]	2 [2 – 2]
	Regression Intercept	0.08 [-0.23 – 0.51]	-0.38 [-0.46 – -0.09]	-0.13 [-0.35 – -0.02]

COx, cerebral oximetry index with cerebral perfusion pressure; COx-a, cerebral oximetry index with arterial blood pressure; HC, healthy control volunteer group; IQR, interquartile range; LoA, limit of agreement; rSO_2, regional cerebral oxygen saturation; SP, elective spinal surgery patient group; TBI, traumatic brain injury patient group.

File S8f: Anchored-Interval – Bland-Altman Analysis of rSO_2 and COx/COx-a in All Populations using 1-Minute Temporal Resolution

Physiologic Variable	Value	Median [IQR]								
		5-Minute Interval	10-Minute Interval	15-Minute Interval	30-Minute Interval	1-Hour Interval	2-Hour Interval	6-Hour Interval	12-Hour Interval	1-Day Interval
**HC Population**										
rSO_2_L	Bias	0.12 [-0.52 – 0.56]	–	–	–	–	–	–	–	–
	Lower LoA	-1.51 [-2.4 – -0.79]	–	–	–	–	–	–	–	–
	Upper LoA	1.56 [1.11 – 2.3]	–	–	–	–	–	–	–	–
	LoA Spread	3.02 [2.35 – 4.31]	–	–	–	–	–	–	–	–
	Relative Bias	5.22 [-13.56 – 21.95]	–	–	–	–	–	–	–	–
	Regression Slope	0.76 [0 – 1.58]	–	–	–	–	–	–	–	–
	Regression Intercept	-53 [-110 – 1.25]	–	–	–	–	–	–	–	–
rSO_2_R	Bias	0.1 [-0.42 – 0.55]	–	–	–	–	–	–	–	–
	Lower LoA	-1.77 [-2.62 – -1.01]	–	–	–	–	–	–	–	–
	Upper LoA	1.86 [0.99 – 2.77]	–	–	–	–	–	–	–	–
	LoA Spread	3.51 [2.52 – 5.05]	–	–	–	–	–	–	–	–
	Relative Bias	3.3 [-13.04 – 15.95]	–	–	–	–	–	–	–	–
	Regression Slope	0.83 [-0.12 – 1.7]	–	–	–	–	–	–	–	–
	Regression Intercept	-55.5 [-110 – 7.48]	–	–	–	–	–	–	–	–
COx_L	Bias	–	–	–	–	–	–	–	–	–
	Lower LoA	–	–	–	–	–	–	–	–	–
	Upper LoA	–	–	–	–	–	–	–	–	–
	LoA Spread	–	–	–	–	–	–	–	–	–
	Relative Bias	–	–	–	–	–	–	–	–	–
	Regression Slope	–	–	–	–	–	–	–	–	–
	Regression Intercept	–	–	–	–	–	–	–	–	–
COx_R	Bias	–	–	–	–	–	–	–	–	–
	Lower LoA	–	–	–	–	–	–	–	–	–
	Upper LoA	–	–	–	–	–	–	–	–	–
	LoA Spread	–	–	–	–	–	–	–	–	–
	Relative Bias	–	–	–	–	–	–	–	–	–
	Regression Slope	–	–	–	–	–	–	–	–	–
	Regression Intercept	–	–	–	–	–	–	–	–	–
COx-a_L	Bias	0.01 [-0.11 – 0.13]	–	–	–	–	–	–	–	–
	Lower LoA	-0.31 [-0.62 – -0.17]	–	–	–	–	–	–	–	–
	Upper LoA	0.85 [-14.44 – 21.33]	–	–	–	–	–	–	–	–
	LoA Spread	0.73 [0.51 – 1.03]	–	–	–	–	–	–	–	–
	Relative Bias	0.35 [0.23 – 0.5]	–	–	–	–	–	–	–	–
	Regression Slope	0 [-0.18 – 0.12]	–	–	–	–	–	–	–	–
	Regression Intercept	0.34 [-0.4 – 0.84]	–	–	–	–	–	–	–	–
COx-a_R	Bias	0.02 [-0.12 – 0.12]	–	–	–	–	–	–	–	–
	Lower LoA	-0.32 [-0.53 – -0.15]	–	–	–	–	–	–	–	–
	Upper LoA	4.06 [-17.62 – 16.56]	–	–	–	–	–	–	–	–
	LoA Spread	0.65 [0.47 – 0.98]	–	–	–	–	–	–	–	–
	Relative Bias	0.32 [0.19 – 0.58]	–	–	–	–	–	–	–	–
	Regression Slope	-0.01 [-0.2 – 0.2]	–	–	–	–	–	–	–	–
	Regression Intercept	0.21 [-0.42 – 1.3]	–	–	–	–	–	–	–	–
**SP Population**										
rSO_2_L	Bias	0.51 [0.05 – 0.92]	1.07 [0.12 – 1.43]	0.99 [0.06 – 3.52]	0.98 [-0.4 – 3.97]	1.47 [-1.52 – 1.51]	–	–	–	–
	Lower LoA	-2.67 [-4.61 – -1.74]	-2.72 [-6.08 – -1.75]	-3.43 [-5.69 – -1.61]	-3.74 [-6.35 – -1.61]	-5.31 [-28.64 – -3.39]	–	–	–	–
	Upper LoA	4.49 [2.92 – 6.18]	6.16 [4.21 – 9.01]	5.64 [3.57 – 10.49]	8.15 [4.65 – 12.01]	8.42 [6.41 – 25.7]	–	–	–	–
	LoA Spread	6.96 [4.6 – 10.89]	10.55 [6.09 – 14.12]	9.9 [5.87 – 13.55]	10.31 [9.43 – 18.63]	13.73 [9.8 – 54.34]	–	–	–	–
	Relative Bias	7.56 [1.14 – 13.69]	12.49 [2.68 – 18.87]	11.29 [0.58 – 24.89]	9.24 [-3.98 – 35.16]	11.32 [3.29 – 18.15]	–	–	–	–
	Regression Slope	0.16 [-0.01 – 0.39]	0.37 [0.15 – 0.91]	0.74 [0.25 – 1.7]	0.96 [0.39 – 1.9]	1.5 [1.35 – 1.8]	–	–	–	–

	Regression Intercept	-11 [-19 – 0.77]	-24 [-51 – -4.1]	-43 [-115 – -11.25]	-59 [-120 – -19]	-110 [-130 – -95]	–	–	–	–
rSO_2_R	Bias	0.38 [0.19 – 0.95]	0.85 [0.14 – 1.68]	1.04 [0.18 – 2.77]	1.11 [0.18 – 3.46]	-0.72 [-3.53 – 0.11]	–	–	–	–
	Lower LoA	-3.54 [-4.49 – -2]	-4.01 [-6.58 – -1.88]	-4.39 [-5.43 – -1.97]	-4.4 [-6.61 – -1.58]	-10.42 [-32.84 – -6.21]	–	–	–	–
	Upper LoA	5.14 [2.4 – 6.17]	6.16 [2.57 – 8.75]	6.78 [3.27 – 9.15]	8.17 [3.82 – 11.93]	8.98 [6.43 – 25.78]	–	–	–	–
	LoA Spread	8.73 [5.27 – 10.61]	10.62 [6.44 – 14.78]	10.85 [7.23 – 13.91]	11.14 [5.88 – 16.38]	19.4 [12.64 – 58.62]	–	–	–	–
	Relative Bias	8.36 [1.89 – 11.98]	10.38 [2.53 – 18.26]	13.23 [1.51 – 20.1]	15.05 [2.73 – 27.44]	-3.72 [-5.1 – 6.13]	–	–	–	–
	Regression Slope	0.16 [0.04 – 0.53]	0.36 [0.18 – 1.25]	0.61 [0.09 – 1.55]	0.92 [0.39 – 2]	2 [1.75 – 2]	–	–	–	–
	Regression Intercept	-10 [-28 – -1.96]	-24 [-74.5 – -7.55]	-29 [-82 – -6.8]	-55 [-97 – -24]	-130 [-140 – -120]	–	–	–	–
COx_L	Bias	–	–	–	–	–	–	–	–	–
	Lower LoA	–	–	–	–	–	–	–	–	–
	Upper LoA	–	–	–	–	–	–	–	–	–
	LoA Spread	–	–	–	–	–	–	–	–	–
	Relative Bias	–	–	–	–	–	–	–	–	–
	Regression Slope	–	–	–	–	–	–	–	–	–
	Regression Intercept	–	–	–	–	–	–	–	–	–
COx_R	Bias	–	–	–	–	–	–	–	–	–
	Lower LoA	–	–	–	–	–	–	–	–	–
	Upper LoA	–	–	–	–	–	–	–	–	–
	LoA Spread	–	–	–	–	–	–	–	–	–
	Relative Bias	–	–	–	–	–	–	–	–	–
	Regression Slope	–	–	–	–	–	–	–	–	–
	Regression Intercept	–	–	–	–	–	–	–	–	–
COx-a_L	Bias	0 [-0.06 – 0.06]	0.02 [-0.04 – 0.06]	0.02 [-0.06 – 0.05]	0.03 [-0.04 – 0.07]	0.07 [0.04 – 0.08]	–	–	–	–
	Lower LoA	-0.75 [-0.89 – -0.44]	-0.72 [-0.94 – -0.52]	-0.86 [-1.01 – -0.53]	-0.72 [-0.99 – -0.53]	-0.66 [-0.83 – -0.59]	–	–	–	–
	Upper LoA	0.09 [-3.17 – 4.9]	1.28 [-2.44 – 4.13]	1.86 [-4.97 – 4.46]	2.16 [-4.06 – 5.44]	3.22 [2.19 – 5.53]	–	–	–	–
	LoA Spread	1.44 [1.13 – 1.84]	1.52 [1.17 – 1.93]	1.56 [1.2 – 2.03]	1.38 [1.11 – 2.06]	1.34 [1.29 – 1.74]	–	–	–	–
	Relative Bias	0.71 [0.58 – 0.91]	0.75 [0.63 – 0.95]	0.78 [0.64 – 1.01]	0.72 [0.66 – 1.03]	0.72 [0.7 – 0.93]	–	–	–	–
	Regression Slope	-0.11 [-0.21 – -0.04]	-0.16 [-0.37 – -0.06]	-0.2 [-0.41 – -0.07]	-0.26 [-0.53 – -0.18]	-0.25 [-0.4 – -0.1]	–	–	–	–
	Regression Intercept	0.8 [0.56 – 0.96]	1.1 [0.87 – 1.45]	1.4 [1.04 – 1.6]	1.7 [1.6 – 1.9]	1.6 [1.15 – 1.6]	–	–	–	–
COx-a_R	Bias	0 [-0.07 – 0.07]	0.02 [-0.09 – 0.06]	-0.03 [-0.13 – 0.11]	-0.02 [-0.13 – 0.12]	0.04 [0 – 0.09]	–	–	–	–
	Lower LoA	-0.67 [-0.84 – -0.55]	-0.78 [-0.92 – -0.53]	-0.82 [-0.97 – -0.53]	-0.74 [-0.98 – -0.48]	-0.65 [-0.78 – -0.55]	–	–	–	–
	Upper LoA	-0.35 [-5.11 – 4.74]	0.97 [-5.77 – 4.18]	-1.41 [-7.57 – 7.26]	-1.08 [-8.9 – 9.98]	2.71 [0.15 – 7.41]	–	–	–	–
	LoA Spread	1.37 [1.16 – 1.64]	1.46 [1.3 – 1.71]	1.47 [1.29 – 1.83]	1.44 [1.2 – 1.74]	1.38 [1.28 – 1.56]	–	–	–	–
	Relative Bias	0.69 [0.58 – 0.88]	0.77 [0.66 – 0.9]	0.78 [0.66 – 0.9]	0.77 [0.59 – 0.93]	0.73 [0.73 – 0.78]	–	–	–	–
	Regression Slope	-0.13 [-0.28 – -0.03]	-0.21 [-0.33 – 0]	-0.25 [-0.4 – -0.01]	-0.32 [-0.42 – -0.14]	-0.31 [-0.32 – -0.25]	–	–	–	–
	Regression Intercept	0.72 [0.4 – 0.97]	1.1 [0.5 – 1.4]	1.4 [1.1 – 1.65]	1.7 [1.3 – 1.9]	1.6 [1.5 – 1.65]	–	–	–	–
**TBI Population**										
rSO_2_L	Bias	0 [-0.1 – 0.04]	0.01 [-0.14 – 0.07]	-0.03 [-0.16 – 0.1]	0.02 [-0.18 – 0.21]	-0.73 [-2.72 – 1.2]	-0.1 [-0.44 – 0.63]	-0.26 [-1.8 – 0.89]	-0.66 [-2.77 – 0.95]	-0.73 [-2.72 – 1.2]
	Lower LoA	-3.45 [-6.64 – -2.29]	-4.54 [-7.44 – -2.54]	-4.92 [-7.96 – -2.8]	-5.47 [-8.83 – -3.26]	-10.82 [-21.36 – -6.72]	-6.95 [-12.08 – -4.51]	-8.14 [-15.16 – -5.14]	-9.34 [-15.5 – -5.97]	-10.82 [-21.36 – -6.72]
	Upper LoA	3.62 [2.25 – 6.47]	4.25 [2.61 – 7.34]	4.72 [2.77 – 8.26]	5.54 [3.47 – 9.67]	10.56 [6.62 – 22.19]	7.24 [4.45 – 13.26]	7.99 [5.02 – 12.35]	7.98 [4.65 – 14.52]	10.56 [6.62 – 22.19]
	LoA Spread	7.09 [4.55 – 13.16]	8.93 [5.14 – 14.71]	9.8 [5.58 – 16.33]	11.01 [6.95 – 17.1]	20.23 [14.26 – 44.58]	13.87 [8.15 – 23.99]	16.35 [10.41 – 26.85]	16.16 [11.59 – 26.71]	20.23 [14.26 – 44.58]
	Relative Bias	-0.05 [-1.33 – 0.49]	0.11 [-1.42 – 0.91]	-0.28 [-1.51 – 1.27]	0.16 [-1.88 – 1.91]	-3.07 [-13.83 – 5.78]	-0.58 [-3.31 – 4.06]	-2.54 [-8.33 – 5.39]	-3.35 [-11.08 – 5.07]	-3.07 [-13.83 – 5.78]
	Regression Slope	0.05 [0.01 – 0.14]	0.07 [0.03 – 0.19]	0.08 [0.02 – 0.25]	0.12 [0.01 – 0.3]	1.8 [0.87 – 2]	0.38 [0 – 1.13]	0.98 [0.18 – 1.55]	1.4 [0.51 – 2]	1.8 [0.87 – 2]
	Regression Intercept	-3.65 [-9.88 – -0.64]	-5.1 [-13 – -1.63]	-4.8 [-16.75 – -1.01]	-7.75 [-21.75 – -0.58]	-97 [-130 – -62.25]	-25.5 [-65.5 – -0.69]	-66 [-107.25 – -13]	-82 [-120 – -33]	-97 [-130 – -62.25]
rSO_2_R	Bias	-0.02 [-0.07 – 0.05]	-0.02 [-0.1 – 0.07]	-0.01 [-0.19 – 0.1]	-0.04 [-0.21 – 0.15]	-0.42 [-3 – 2.5]	-0.08 [-0.73 – 0.57]	-0.56 [-2.11 – 1.15]	-0.7 [-3.11 – 1.61]	-0.42 [-3 – 2.5]
	Lower LoA	-3.54 [-7.06 – -1.97]	-4.25 [-8.23 – -2.32]	-4.66 [-9.18 – -2.57]	-5.51 [-9.44 – -3.08]	-9.83 [-21.99 – -5.27]	-6.88 [-12.71 – -3.51]	-9.44 [-15.52 – -5.62]	-9.48 [-17.07 – -5.64]	-9.83 [-21.99 – -5.27]

	Upper LoA	3.61 [1.94 – 6.83]	4.21 [2.32 – 7.94]	4.71 [2.68 – 8.62]	5.39 [3.37 – 9.85]	10.23 [6.54 – 22.58]	7.29 [3.72 – 12.53]	8.38 [4.9 – 14.46]	9.8 [4.06 – 15.02]	10.23 [6.54 – 22.58]
	LoA Spread	7.05 [3.98 – 13.89]	8.34 [4.71 – 16.28]	9.37 [5.31 – 18.17]	11.22 [6.51 – 18.8]	19.91 [14.5 – 39.08]	14.04 [8.46 – 25.15]	20.14 [11 – 26.05]	17.65 [12.04 – 30.78]	19.91 [14.5 – 39.08]
	Relative Bias	-0.24 [-0.96 – 0.75]	-0.23 [-1.18 – 1.12]	-0.2 [-1.49 – 1.42]	-0.71 [-1.71 – 1.47]	-1.89 [-12.15 – 14.73]	-0.63 [-5.01 – 2.48]	-3.36 [-12.31 – 5.27]	-4.04 [-18.79 – 4.9]	-1.89 [-12.15 – 14.73]
	Regression Slope	0.05 [0.02 – 0.12]	0.08 [0.02 – 0.2]	0.1 [0.02 – 0.21]	0.14 [0.01 – 0.34]	1.7 [1.15 – 2]	0.37 [0.04 – 0.67]	0.79 [0.15 – 1.58]	1.3 [0.45 – 1.95]	1.7 [1.15 – 2]
	Regression Intercept	-3.8 [-8.43 – -0.94]	-5.15 [-14 – -1.3]	-6.6 [-13 – -1.03]	-8.4 [-22 – -1]	-105 [-122.5 – -79.75]	-26.5 [-43 – -2.95]	-53.5 [-110 – -12]	-90 [-120 – -30.5]	-105 [-122.5 – -79.75]
COx_L	Bias	0 [-0.01 – 0.02]	0 [-0.01 – 0.02]	0 [-0.01 – 0.02]	0 [-0.01 – 0.02]	0.03 [-0.04 – 0.09]	0.01 [-0.02 – 0.03]	0.01 [-0.01 – 0.04]	0.01 [-0.03 – 0.09]	0.03 [-0.04 – 0.09]
	Lower LoA	-0.55 [-0.62 – -0.5]	-0.59 [-0.68 – -0.54]	-0.6 [-0.7 – -0.55]	-0.61 [-0.72 – -0.57]	-0.6 [-0.73 – -0.52]	-0.63 [-0.73 – -0.57]	-0.63 [-0.72 – -0.57]	-0.63 [-0.71 – -0.55]	-0.6 [-0.73 – -0.52]
	Upper LoA	0.56 [0.51 – 0.62]	0.6 [0.54 – 0.67]	0.61 [0.55 – 0.68]	0.63 [0.57 – 0.71]	0.67 [0.64 – 0.73]	0.65 [0.59 – 0.74]	0.67 [0.59 – 0.75]	0.68 [0.59 – 0.74]	0.67 [0.64 – 0.73]
	LoA Spread	1.11 [1.01 – 1.23]	1.19 [1.09 – 1.33]	1.22 [1.11 – 1.38]	1.23 [1.15 – 1.42]	1.3 [1.22 – 1.46]	1.27 [1.18 – 1.45]	1.3 [1.21 – 1.44]	1.28 [1.21 – 1.45]	1.3 [1.22 – 1.46]
	Relative Bias	0.12 [-0.69 – 1.51]	0.04 [-1.07 – 1.49]	-0.06 [-1.04 – 1.59]	0.37 [-1.21 – 1.82]	2.28 [-3.53 – 7.2]	0.5 [-1.31 – 2.99]	0.75 [-1.06 – 3.96]	0.81 [-2.15 – 6.81]	2.28 [-3.53 – 7.2]
	Regression Slope	0.77 [0.65 – 0.85]	1.1 [0.97 – 1.3]	1.3 [1.13 – 1.5]	1.6 [1.4 – 1.7]	2 [1.88 – 2]	1.8 [1.7 – 1.9]	1.9 [1.8 – 2]	2 [1.9 – 2]	2 [1.88 – 2]
	Regression Intercept	-0.02 [-0.1 – 0.03]	-0.03 [-0.14 – 0.02]	-0.03 [-0.16 – 0.03]	-0.04 [-0.18 – 0.04]	-0.1 [-0.21 – 0.08]	-0.03 [-0.21 – 0.06]	-0.05 [-0.2 – 0.05]	-0.03 [-0.2 – 0.06]	-0.1 [-0.21 – 0.08]
COx_R	Bias	0 [-0.01 – 0.01]	0 [-0.01 – 0.01]	0 [-0.01 – 0.01]	0 [-0.02 – 0.01]	-0.04 [-0.09 – 0.01]	0 [-0.03 – 0.01]	0 [-0.03 – 0.03]	0 [-0.03 – 0.04]	-0.04 [-0.09 – 0.01]
	Lower LoA	-0.54 [-0.6 – -0.49]	-0.59 [-0.67 – -0.53]	-0.59 [-0.68 – -0.54]	-0.61 [-0.7 – -0.56]	-0.71 [-0.78 – -0.61]	-0.63 [-0.71 – -0.57]	-0.65 [-0.73 – -0.57]	-0.62 [-0.74 – -0.56]	-0.71 [-0.78 – -0.61]
	Upper LoA	0.55 [0.48 – 0.61]	0.6 [0.53 – 0.66]	0.62 [0.54 – 0.67]	0.62 [0.54 – 0.7]	0.65 [0.59 – 0.72]	0.64 [0.54 – 0.7]	0.65 [0.56 – 0.72]	0.65 [0.57 – 0.7]	0.65 [0.59 – 0.72]
	LoA Spread	1.09 [0.98 – 1.21]	1.19 [1.04 – 1.32]	1.19 [1.08 – 1.35]	1.24 [1.1 – 1.38]	1.36 [1.22 – 1.5]	1.27 [1.12 – 1.39]	1.29 [1.15 – 1.41]	1.28 [1.12 – 1.42]	1.36 [1.22 – 1.5]
	Relative Bias	-0.03 [-1.04 – 0.74]	0.04 [-1.27 – 0.9]	-0.01 [-1.13 – 0.74]	-0.1 [-1.46 – 0.9]	-2.68 [-6.31 – 0.95]	-0.1 [-2.25 – 1.27]	-0.32 [-2.32 – 2.21]	-0.29 [-2.18 – 3.32]	-2.68 [-6.31 – 0.95]
	Regression Slope	0.8 [0.64 – 0.87]	1.15 [1 – 1.3]	1.3 [1.2 – 1.4]	1.6 [1.4 – 1.7]	2 [1.88 – 2]	1.85 [1.7 – 1.9]	1.9 [1.8 – 2]	1.9 [1.85 – 2]	2 [1.88 – 2]
	Regression Intercept	0 [-0.01 – 0.02]	0 [-0.01 – 0.02]	0 [-0.01 – 0.02]	0 [-0.01 – 0.02]	0.02 [-0.01 – 0.04]	0.01 [-0.02 – 0.02]	0.01 [-0.01 – 0.02]	0.01 [-0.02 – 0.03]	0.02 [-0.01 – 0.04]
COx-a_L	Bias	0.34 [-0.75 – 1.67]	0.01 [-1.02 – 1.43]	0.24 [-1.01 – 1.53]	0.31 [-0.89 – 1.71]	1.17 [-0.99 – 3.18]	0.62 [-1.27 – 1.93]	0.71 [-0.81 – 2.06]	0.87 [-1.13 – 2.84]	1.17 [-0.99 – 3.18]
	Lower LoA	1.08 [0.96 – 1.18]	1.16 [1.04 – 1.25]	1.18 [1.06 – 1.29]	1.21 [1.09 – 1.33]	1.24 [1.13 – 1.35]	1.24 [1.1 – 1.36]	1.26 [1.14 – 1.36]	1.24 [1.15 – 1.34]	1.24 [1.13 – 1.35]
	Upper LoA	-0.53 [-0.58 – -0.48]	-0.57 [-0.62 – -0.52]	-0.58 [-0.64 – -0.54]	-0.6 [-0.66 – -0.55]	-0.59 [-0.7 – -0.53]	-0.61 [-0.67 – -0.55]	-0.62 [-0.68 – -0.55]	-0.6 [-0.67 – -0.53]	-0.59 [-0.7 – -0.53]
	LoA Spread	0.54 [0.48 – 0.59]	0.59 [0.53 – 0.62]	0.6 [0.53 – 0.66]	0.61 [0.55 – 0.67]	0.62 [0.58 – 0.68]	0.63 [0.56 – 0.67]	0.64 [0.58 – 0.7]	0.64 [0.57 – 0.7]	0.62 [0.58 – 0.68]
	Relative Bias	-0.08 [-0.12 – -0.02]	-0.13 [-0.19 – -0.03]	-0.15 [-0.23 – -0.03]	-0.17 [-0.28 – -0.03]	-0.21 [-0.31 – -0.06]	-0.19 [-0.31 – -0.03]	-0.21 [-0.31 – -0.02]	-0.22 [-0.32 – -0.03]	-0.21 [-0.31 – -0.06]
	Regression Slope	0.79 [0.72 – 0.87]	1.2 [1.1 – 1.3]	1.35 [1.3 – 1.5]	1.6 [1.5 – 1.7]	2 [1.9 – 2]	1.9 [1.78 – 1.9]	1.9 [1.9 – 2]	2 [1.9 – 2]	2 [1.9 – 2]
	Regression Intercept	0 [-0.01 – 0.01]	0 [-0.01 – 0.01]	0 [-0.02 – 0.01]	0 [-0.01 – 0.01]	-0.03 [-0.07 – 0]	0 [-0.02 – 0.01]	-0.01 [-0.03 – 0.02]	-0.01 [-0.04 – 0.03]	-0.03 [-0.07 – 0]
COx-a_R	Bias	0.06 [-1.1 – 0.84]	0.02 [-1.12 – 0.96]	0 [-1.17 – 1.22]	-0.03 [-1.13 – 1.02]	-2.69 [-4.85 – 0.24]	-0.36 [-1.57 – 1.34]	-0.55 [-2.71 – 1.43]	-0.9 [-2.88 – 2.31]	-2.69 [-4.85 – 0.24]
	Lower LoA	1.06 [0.97 – 1.16]	1.14 [1.05 – 1.22]	1.17 [1.05 – 1.27]	1.19 [1.07 – 1.31]	1.25 [1.18 – 1.31]	1.24 [1.1 – 1.33]	1.25 [1.11 – 1.33]	1.22 [1.1 – 1.32]	1.25 [1.18 – 1.31]
	Upper LoA	-0.53 [-0.58 – -0.47]	-0.57 [-0.63 – -0.51]	-0.58 [-0.64 – -0.52]	-0.6 [-0.66 – -0.54]	-0.64 [-0.72 – -0.61]	-0.62 [-0.68 – -0.55]	-0.64 [-0.69 – -0.56]	-0.63 [-0.68 – -0.55]	-0.64 [-0.72 – -0.61]
	LoA Spread	0.53 [0.48 – 0.58]	0.57 [0.52 – 0.61]	0.58 [0.53 – 0.64]	0.6 [0.54 – 0.66]	0.58 [0.54 – 0.68]	0.62 [0.54 – 0.67]	0.61 [0.55 – 0.67]	0.61 [0.54 – 0.67]	0.58 [0.54 – 0.68]
	Relative Bias	-0.06 [-0.13 – 0]	-0.09 [-0.19 – -0.01]	-0.11 [-0.2 – -0.01]	-0.12 [-0.24 – -0.01]	-0.25 [-0.38 – -0.16]	-0.15 [-0.27 – -0.02]	-0.17 [-0.32 – -0.03]	-0.21 [-0.3 – -0.05]	-0.25 [-0.38 – -0.16]
	Regression Slope	0.81 [0.72 – 0.88]	1.2 [1.1 – 1.3]	1.4 [1.3 – 1.5]	1.6 [1.5 – 1.7]	2 [1.9 – 2]	1.9 [1.8 – 1.9]	1.9 [1.9 – 2]	2 [1.9 – 2]	2 [1.9 – 2]
	Regression Intercept	-0.02 [-0.09 – 0.03]	-0.02 [-0.13 – 0.04]	-0.03 [-0.17 – 0.05]	-0.03 [-0.18 – 0.05]	-0.11 [-0.3 – 0.02]	-0.05 [-0.2 – 0.07]	-0.05 [-0.22 – 0.1]	-0.09 [-0.2 – 0.1]	-0.11 [-0.3 – 0.02]

COx, cerebral oximetry index with cerebral perfusion pressure; COx-a, cerebral oximetry index with arterial blood pressure; HC, healthy control volunteer group; IQR, interquartile range; r-value, Pearson correlation coefficient; rSO_2, regional cerebral oxygen saturation; SP, elective spinal surgery patient group; TBI, traumatic brain injury patient group.

File S8g: Anchored-Interval – Bland-Altman Analysis of rSO_2 and COx/COx-a in All Populations using 5-Minute Temporal Resolution

Physiologic Variable	Value	Median [IQR]								
		5-Minute Interval	10-Minute Interval	15-Minute Interval	30-Minute Interval	1-Hour Interval	2-Hour Interval	6-Hour Interval	12-Hour Interval	1-Day Interval
**HC Population**										
rSO_2_L	Bias	-0.13 [-0.87 – 0.75]	–	–	–	–	–	–	–	–
	Lower LoA	-2.91 [-4.69 – -1.13]	–	–	–	–	–	–	–	–
	Upper LoA	3.32 [1.39 – 4.73]	–	–	–	–	–	–	–	–
	LoA Spread	5.72 [3.01 – 8.98]	–	–	–	–	–	–	–	–
	Relative Bias	-0.28 [-9.75 – 16.38]	–	–	–	–	–	–	–	–
	Regression Slope	-1.5 [-2.6 – 1.2]	–	–	–	–	–	–	–	–
	Regression Intercept	0.6 [-1.9 – 137.5]	–	–	–	–	–	–	–	–
rSO_2_R	Bias	-0.01 [-0.88 – 0.98]	–	–	–	–	–	–	–	–
	Lower LoA	-2.59 [-4.3 – -0.86]	–	–	–	–	–	–	–	–
	Upper LoA	3.1 [0.92 – 4.45]	–	–	–	–	–	–	–	–
	LoA Spread	5.38 [2.44 – 8.34]	–	–	–	–	–	–	–	–
	Relative Bias	-0.2 [-13.44 – 20.47]	–	–	–	–	–	–	–	–
	Regression Slope	-1.55 [-3.35 – 0.53]	–	–	–	–	–	–	–	–
	Regression Intercept	1.75 [-1.85 – 147.5]	–	–	–	–	–	–	–	–
COx_L	Bias	–	–	–	–	–	–	–	–	–
	Lower LoA	–	–	–	–	–	–	–	–	–
	Upper LoA	–	–	–	–	–	–	–	–	–
	LoA Spread	–	–	–	–	–	–	–	–	–
	Relative Bias	–	–	–	–	–	–	–	–	–
	Regression Slope	–	–	–	–	–	–	–	–	–
	Regression Intercept	–	–	–	–	–	–	–	–	–
COx_R	Bias	–	–	–	–	–	–	–	–	–
	Lower LoA	–	–	–	–	–	–	–	–	–
	Upper LoA	–	–	–	–	–	–	–	–	–
	LoA Spread	–	–	–	–	–	–	–	–	–
	Relative Bias	–	–	–	–	–	–	–	–	–
	Regression Slope	–	–	–	–	–	–	–	–	–
	Regression Intercept	–	–	–	–	–	–	–	–	–
COx-a_L	Bias	-0.01 [-0.17 – 0.22]	–	–	–	–	–	–	–	–
	Lower LoA	-0.56 [-0.99 – -0.31]	–	–	–	–	–	–	–	–
	Upper LoA	-2.14 [-18.98 – 15.69]	–	–	–	–	–	–	–	–
	LoA Spread	1.11 [0.54 – 1.79]	–	–	–	–	–	–	–	–
	Relative Bias	0.5 [0.22 – 0.96]	–	–	–	–	–	–	–	–
	Regression Slope	0.09 [-0.2 – 0.43]	–	–	–	–	–	–	–	–
	Regression Intercept	-1.4 [-3.35 – 0.08]	–	–	–	–	–	–	–	–
COx-a_R	Bias	-0.03 [-0.18 – 0.17]	–	–	–	–	–	–	–	–
	Lower LoA	-0.6 [-1.2 – -0.23]	–	–	–	–	–	–	–	–
	Upper LoA	-0.46 [-9.83 – 15.24]	–	–	–	–	–	–	–	–
	LoA Spread	1.08 [0.56 – 2.34]	–	–	–	–	–	–	–	–
	Relative Bias	0.58 [0.22 – 1.05]	–	–	–	–	–	–	–	–
	Regression Slope	-0.06 [-0.29 – 0.29]	–	–	–	–	–	–	–	–
	Regression Intercept	-1.4 [-3.25 – 0.17]	–	–	–	–	–	–	–	–
**SP Population**										
rSO_2_L	Bias	0.59 [0.26 – 1.24]	0.88 [0.3 – 2.34]	0.96 [0.17 – 2.69]	0.96 [0.3 – 4.02]	1.64 [1.25 – 23.88]	–	–	–	–
	Lower LoA	-2.64 [-5.04 – -1.82]	-2.53 [-5.71 – -1.87]	-3.33 [-6.54 – -1.83]	-3.33 [-6.02 – -1.72]	-5.37 [-63.16 – -3.57]	–	–	–	–
	Upper LoA	3.83 [3.11 – 6.93]	5.45 [3.66 – 7.59]	5.86 [3.95 – 10.92]	6.29 [3.93 – 12.93]	8.65 [6.06 – 110.92]	–	–	–	–
	LoA Spread	7.21 [4.79 – 11.74]	8.67 [6.07 – 13.16]	11.29 [6.1 – 15.74]	9.7 [6.08 – 16.26]	14.02 [9.63 – 174.09]	–	–	–	–
	Relative Bias	9.93 [3.81 – 12.54]	11.45 [5.42 – 22.11]	11.6 [2.83 – 21.17]	13.73 [5.82 – 23.74]	13.8 [12.74 – 15.11]	–	–	–	–

	Regression Slope	0.18 [-0.12 – 0.5]	0.22 [-0.2 – 0.76]	0.58 [0.04 – 1.05]	0.59 [0.06 – 1.3]	0.99 [-0.51 – 1.05]	–	–	–	–
	Regression Intercept	-8.7 [-34.5 – 7.4]	-13 [-47.5 – 11]	-39 [-63.5 – -0.13]	-36 [-78 – -2.4]	-72 [-72.5 – 34]	–	–	–	–
rSO_2_R	Bias	0.4 [0.19 – 1.06]	0.54 [0.01 – 1.31]	0.59 [-0.33 – 2.08]	1.09 [0.11 – 2.54]	1.6 [0.29 – 4.39]	–	–	–	–
	Lower LoA	-3.24 [-4.78 – -1.76]	-2.87 [-6.01 – -1.69]	-3.58 [-5.75 – -1.82]	-3.32 [-5.72 – -1.91]	-5.29 [-73.17 – -2.86]	–	–	–	–
	Upper LoA	4.77 [2.71 – 6.1]	5.5 [2.87 – 8.21]	6.24 [3.21 – 8.59]	6.96 [3.09 – 10.91]	3.63 [3.43 – 79.53]	–	–	–	–
	LoA Spread	7.44 [5.55 – 10.21]	8.39 [5.03 – 13.55]	9.63 [6.33 – 13.48]	10.26 [6.01 – 15.33]	8.52 [6.29 – 152.5]	–	–	–	–
	Relative Bias	9.46 [2.35 – 14.94]	9.93 [0.12 – 17.23]	13.96 [-3.01 – 19.52]	16.72 [2.47 – 23.01]	2.43 [-4.81 – 20.9]	–	–	–	–
	Regression Slope	0.19 [-0.06 – 0.53]	0.39 [-0.01 – 0.76]	0.42 [0.23 – 0.84]	0.74 [0.34 – 1.9]	0.6 [-0.6 – 1.2]	–	–	–	–
	Regression Intercept	-5.9 [-37 – 4.5]	-28 [-47.5 – 1.9]	-30 [-59.5 – -12]	-47 [-96 – -22]	-43 [-81.5 – 38.5]	–	–	–	–
COx_L	Bias	–	–	–	–	–	–	–	–	–
	Lower LoA	–	–	–	–	–	–	–	–	–
	Upper LoA	–	–	–	–	–	–	–	–	–
	LoA Spread	–	–	–	–	–	–	–	–	–
	Relative Bias	–	–	–	–	–	–	–	–	–
	Regression Slope	–	–	–	–	–	–	–	–	–
	Regression Intercept	–	–	–	–	–	–	–	–	–
COx_R	Bias	–	–	–	–	–	–	–	–	–
	Lower LoA	–	–	–	–	–	–	–	–	–
	Upper LoA	–	–	–	–	–	–	–	–	–
	LoA Spread	–	–	–	–	–	–	–	–	–
	Relative Bias	–	–	–	–	–	–	–	–	–
	Regression Slope	–	–	–	–	–	–	–	–	–
	Regression Intercept	–	–	–	–	–	–	–	–	–
COx-a_L	Bias	0.03 [-0.05 – 0.07]	0.04 [-0.06 – 0.09]	-0.01 [-0.08 – 0.07]	0.06 [-0.03 – 0.13]	-0.02 [-0.03 – 0.04]	–	–	–	–
	Lower LoA	-0.6 [-0.96 – -0.46]	-0.6 [-0.82 – -0.46]	-0.62 [-0.99 – -0.48]	-0.54 [-0.75 – -0.38]	-0.61 [-0.69 – -0.51]	–	–	–	–
	Upper LoA	1.73 [-4.84 – 6.19]	3.87 [-5.06 – 9]	-0.75 [-5.43 – 8.72]	4.12 [-1.97 – 12.74]	-1.43 [-2.2 – 4.31]	–	–	–	–
	LoA Spread	1.28 [0.97 – 1.78]	1.26 [0.92 – 1.62]	1.35 [1.05 – 1.89]	1.17 [0.98 – 1.63]	1.19 [1.11 – 1.32]	–	–	–	–
	Relative Bias	0.72 [0.49 – 0.92]	0.63 [0.45 – 0.89]	0.65 [0.54 – 0.9]	0.66 [0.5 – 0.89]	0.61 [0.6 – 0.65]	–	–	–	–
	Regression Slope	-0.16 [-0.32 – -0.07]	-0.2 [-0.42 – -0.08]	-0.25 [-0.51 – -0.12]	-0.21 [-0.52 – 0]	-0.29 [-0.46 – -0.2]	–	–	–	–
	Regression Intercept	1.2 [0.56 – 1.8]	1.5 [0.86 – 1.9]	1.7 [0.96 – 2.25]	1.9 [1.5 – 2.1]	1.7 [1.5 – 1.75]	–	–	–	–
COx-a_R	Bias	-0.04 [-0.11 – 0.09]	-0.07 [-0.11 – 0.09]	-0.07 [-0.16 – 0.09]	-0.01 [-0.11 – 0.09]	0.01 [-0.03 – 0.05]	–	–	–	–
	Lower LoA	-0.7 [-0.93 – -0.51]	-0.65 [-0.94 – -0.49]	-0.76 [-0.98 – -0.52]	-0.59 [-0.84 – -0.45]	-0.59 [-0.72 – -0.48]	–	–	–	–
	Upper LoA	-3.25 [-8.17 – 6.69]	-4.38 [-8.77 – 8.91]	-5.88 [-10.15 – 8.01]	-0.86 [-8.75 – 9.71]	0.48 [-1.73 – 5.11]	–	–	–	–
	LoA Spread	1.47 [1.06 – 1.7]	1.42 [1.03 – 1.62]	1.53 [1.23 – 1.76]	1.46 [1 – 1.61]	1.19 [1.07 – 1.38]	–	–	–	–
	Relative Bias	0.65 [0.59 – 0.86]	0.63 [0.51 – 0.83]	0.68 [0.59 – 0.88]	0.71 [0.57 – 0.81]	0.6 [0.58 – 0.66]	–	–	–	–
	Regression Slope	-0.23 [-0.3 – -0.06]	-0.28 [-0.37 – 0.03]	-0.28 [-0.38 – 0.05]	-0.3 [-0.4 – -0.03]	-0.37 [-0.41 – -0.26]	–	–	–	–
	Regression Intercept	1.1 [0.43 – 1.45]	1.2 [0.53 – 1.5]	1.1 [0.17 – 1.75]	1.1 [0.73 – 1.8]	1.8 [1.45 – 1.85]	–	–	–	–
**TBI Population**										
rSO_2_L	Bias	0.01 [-0.06 – 0.08]	0.03 [-0.09 – 0.16]	-0.01 [-0.17 – 0.18]	0 [-0.19 – 0.26]	-0.01 [-0.35 – 0.43]	-0.1 [-0.54 – 0.69]	-0.21 [-1.52 – 0.92]	-0.45 [-2.23 – 1.03]	-0.85 [-3.5 – 2.17]
	Lower LoA	-3.54 [-6.91 – -2.14]	-3.91 [-8.03 – -2.41]	-4.21 [-8.58 – -2.73]	-5.38 [-9.81 – -3.15]	-6.28 [-10.58 – -3.57]	-6.78 [-11.75 – -4.07]	-7.49 [-14 – -4.5]	-8.98 [-14.07 – -5.03]	-10.68 [-17.7 – -6.03]
	Upper LoA	3.48 [2.18 – 6.99]	4.02 [2.57 – 8.1]	4.31 [2.75 – 9.85]	4.95 [3.29 – 10.67]	5.91 [3.49 – 10.61]	6.81 [3.98 – 13.13]	7.7 [4.34 – 13.73]	8.31 [4.88 – 15.43]	11.18 [5.51 – 17.94]
	LoA Spread	7.15 [4.26 – 13.74]	7.95 [4.94 – 16.9]	8.51 [5.38 – 17.95]	10.4 [6.81 – 19.46]	12 [6.8 – 20.79]	13.46 [7.9 – 24.89]	14.94 [10.8 – 26.22]	16.07 [11.41 – 29.95]	23.09 [13.65 – 36.05]
	Relative Bias	0.09 [-0.91 – 1.13]	0.28 [-1.5 – 2.13]	-0.15 [-2.02 – 1.44]	-0.04 [-1.57 – 3.05]	-0.11 [-2.82 – 2.87]	-0.56 [-3.93 – 4.26]	-2 [-7.75 – 5.65]	-1.93 [-11.67 – 5.76]	-5.97 [-15.67 – 8.47]
	Regression Slope	0.03 [0 – 0.1]	0.07 [-0.01 – 0.2]	0.1 [0.01 – 0.23]	0.12 [0.01 – 0.37]	0.32 [0.05 – 0.63]	0.5 [0.12 – 0.99]	0.82 [0.49 – 1.58]	1.4 [0.67 – 2]	1.75 [1.03 – 1.93]
	Regression Intercept	-1.9 [-6.6 – 0.22]	-4.3 [-12.75 – 0.64]	-6.05 [-15 – -0.68]	-7.35 [-21.75 – -0.91]	-19.5 [-43.5 – -2.25]	-31.5 [-62.5 – -6.33]	-52.5 [-110 – -28.25]	-80 [-115 – -42]	-98.5 [-112.5 – -61.75]
rSO_2_R	Bias	-0.02 [-0.14 – 0.04]	-0.03 [-0.2 – 0.09]	-0.05 [-0.19 – 0.17]	-0.04 [-0.29 – 0.25]	-0.08 [-0.57 – 0.38]	-0.07 [-0.95 – 0.63]	-0.56 [-1.86 – 0.58]	-0.44 [-2.53 – 1.17]	-0.42 [-2.57 – 2.38]

	Lower LoA	-3.64 [-6.74 – -1.79]	-4.3 [-8.18 – -2.24]	-4.6 [-8.97 – -2.28]	-4.93 [-9.66 – -2.86]	-5.86 [-10.19 – -3.36]	-6.89 [-11.92 – -3.51]	-8.38 [-15.22 – -5.14]	-8.8 [-17.26 – -5.12]	-8.15 [-18.43 – -5.01]
	Upper LoA	3.62 [1.69 – 6.83]	4.44 [2.06 – 7.69]	4.63 [2.48 – 9.01]	5.27 [2.94 – 9.23]	6.72 [3.23 – 9.33]	7.72 [3.29 – 11.61]	8 [3.94 – 13.06]	9.47 [3.66 – 16.47]	9.82 [5.26 – 19.62]
	LoA Spread	7.3 [3.44 – 13.52]	8.74 [4.28 – 15.8]	9.33 [4.77 – 18.28]	10.29 [6.26 – 19]	11.78 [6.55 – 20.03]	14.22 [6.87 – 23.36]	18.83 [10.38 – 25.71]	16.64 [11.1 – 32.42]	18.95 [12.84 – 33.32]
	Relative Bias	-0.36 [-2.08 – 0.87]	-0.36 [-2.14 – 1.35]	-0.54 [-2.15 – 1.67]	-0.43 [-2.46 – 2.56]	-1.04 [-5.02 – 3.16]	-0.87 [-6.03 – 3.31]	-3.04 [-11.88 – 4.19]	-4.04 [-11.53 – 2.6]	-1.48 [-11.46 – 13.66]
	Regression Slope	0.04 [0.01 – 0.1]	0.07 [0.02 – 0.13]	0.07 [0.02 – 0.19]	0.12 [0.03 – 0.32]	0.29 [0.08 – 0.54]	0.42 [0.06 – 0.88]	1 [0.33 – 1.6]	1.4 [0.61 – 1.8]	1.45 [1.06 – 1.9]
	Regression Intercept	-2.9 [-6.48 – -0.4]	-4.7 [-9.18 – -1.23]	-5 [-11.75 – -1.13]	-8.3 [-21.25 – -1.8]	-17 [-37 – -5.83]	-26 [-63.5 – -3.25]	-68 [-110 – -22]	-89 [-115 – -41]	-92 [-112.5 – -72.5]
COx_L	Bias	0 [-0.01 – 0.02]	0 [-0.01 – 0.02]	0.01 [-0.01 – 0.02]	0.01 [-0.02 – 0.03]	0 [-0.02 – 0.03]	0.01 [-0.01 – 0.04]	0.01 [-0.01 – 0.05]	0.02 [-0.02 – 0.05]	0.02 [-0.05 – 0.06]
	Lower LoA	-0.5 [-0.56 – -0.46]	-0.51 [-0.58 – -0.47]	-0.52 [-0.59 – -0.47]	-0.52 [-0.6 – -0.47]	-0.52 [-0.6 – -0.47]	-0.52 [-0.61 – -0.46]	-0.52 [-0.6 – -0.47]	-0.53 [-0.6 – -0.44]	-0.52 [-0.64 – -0.45]
	Upper LoA	0.51 [0.47 – 0.57]	0.53 [0.48 – 0.6]	0.54 [0.48 – 0.61]	0.54 [0.49 – 0.62]	0.55 [0.49 – 0.62]	0.55 [0.49 – 0.61]	0.56 [0.5 – 0.65]	0.56 [0.51 – 0.62]	0.56 [0.52 – 0.63]
	LoA Spread	1.01 [0.94 – 1.14]	1.04 [0.96 – 1.19]	1.05 [0.97 – 1.19]	1.05 [0.99 – 1.22]	1.05 [0.98 – 1.23]	1.05 [0.97 – 1.23]	1.07 [1.01 – 1.24]	1.07 [1.01 – 1.25]	1.14 [1.02 – 1.25]
	Relative Bias	0.3 [-0.83 – 1.94]	0.42 [-0.84 – 1.96]	0.45 [-1.19 – 2.3]	0.69 [-1.52 – 2.65]	0.42 [-1.32 – 2.96]	0.66 [-0.97 – 3.41]	1.08 [-1.25 – 3.59]	1.78 [-1.49 – 5.67]	1.64 [-4.17 – 5.32]
	Regression Slope	1.2 [0.97 – 1.4]	1.4 [1.2 – 1.6]	1.6 [1.3 – 1.7]	1.7 [1.4 – 1.88]	1.7 [1.53 – 1.9]	1.8 [1.6 – 1.9]	1.9 [1.8 – 2]	2 [1.9 – 2]	2 [1.9 – 2]
	Regression Intercept	-0.03 [-0.14 – 0.03]	-0.03 [-0.16 – 0.03]	-0.03 [-0.17 – 0.03]	-0.03 [-0.19 – 0.03]	-0.04 [-0.19 – 0.03]	-0.04 [-0.2 – 0.05]	-0.04 [-0.19 – 0.05]	-0.06 [-0.21 – 0.06]	-0.1 [-0.21 – 0.05]
COx_R	Bias	0 [-0.02 – 0.01]	0 [-0.02 – 0.01]	0 [-0.02 – 0.01]	0 [-0.02 – 0.01]	0 [-0.03 – 0.02]	0 [-0.03 – 0.02]	0.01 [-0.04 – 0.03]	0 [-0.04 – 0.05]	-0.03 [-0.08 – 0.05]
	Lower LoA	-0.5 [-0.57 – -0.45]	-0.53 [-0.59 – -0.46]	-0.54 [-0.59 – -0.47]	-0.54 [-0.6 – -0.47]	-0.55 [-0.6 – -0.47]	-0.55 [-0.61 – -0.47]	-0.56 [-0.62 – -0.47]	-0.52 [-0.62 – -0.45]	-0.61 [-0.66 – -0.52]
	Upper LoA	0.51 [0.45 – 0.57]	0.53 [0.46 – 0.59]	0.53 [0.47 – 0.6]	0.54 [0.48 – 0.61]	0.54 [0.48 – 0.61]	0.54 [0.46 – 0.62]	0.55 [0.46 – 0.61]	0.54 [0.47 – 0.61]	0.55 [0.5 – 0.63]
	LoA Spread	1.02 [0.91 – 1.12]	1.04 [0.93 – 1.15]	1.06 [0.94 – 1.17]	1.08 [0.95 – 1.18]	1.09 [0.95 – 1.21]	1.08 [0.95 – 1.2]	1.08 [0.97 – 1.18]	1.08 [0.96 – 1.19]	1.14 [1.01 – 1.26]
	Relative Bias	-0.15 [-2.07 – 0.99]	-0.23 [-2.25 – 0.86]	-0.01 [-2.03 – 1.21]	-0.01 [-2.46 – 1.22]	-0.08 [-2.61 – 1.44]	-0.04 [-2.87 – 1.81]	0.62 [-2.86 – 3.03]	-0.41 [-3.33 – 5.11]	-2.15 [-8.33 – 4.41]
	Regression Slope	1.3 [1 – 1.4]	1.5 [1.2 – 1.6]	1.6 [1.3 – 1.7]	1.7 [1.5 – 1.8]	1.8 [1.63 – 1.9]	1.8 [1.7 – 1.9]	1.9 [1.8 – 2]	1.9 [1.8 – 2]	1.9 [1.9 – 2]
	Regression Intercept	0 [-0.01 – 0.01]	0 [-0.01 – 0.01]	0 [-0.01 – 0.02]	0 [-0.02 – 0.02]	0 [-0.02 – 0.02]	0 [-0.02 – 0.02]	0 [-0.01 – 0.03]	0.01 [-0.02 – 0.03]	0.02 [-0.04 – 0.04]
COx-a_L	Bias	0.22 [-0.95 – 1.4]	0.2 [-1.37 – 1.52]	0.22 [-1.02 – 1.73]	0.28 [-1.53 – 1.64]	0.32 [-2.07 – 2.08]	0.19 [-1.91 – 2.05]	0.33 [-1.21 – 2.82]	1.34 [-1.48 – 3.53]	2 [-3.03 – 3.58]
	Lower LoA	0.98 [0.89 – 1.07]	1.01 [0.91 – 1.11]	1.02 [0.91 – 1.1]	1.03 [0.92 – 1.1]	1.03 [0.91 – 1.1]	1.03 [0.92 – 1.11]	1.03 [0.95 – 1.13]	1.04 [0.94 – 1.12]	1.04 [0.91 – 1.13]
	Upper LoA	-0.49 [-0.54 – -0.44]	-0.5 [-0.55 – -0.45]	-0.51 [-0.55 – -0.45]	-0.51 [-0.56 – -0.46]	-0.51 [-0.55 – -0.46]	-0.51 [-0.56 – -0.45]	-0.51 [-0.56 – -0.46]	-0.51 [-0.57 – -0.45]	-0.49 [-0.59 – -0.45]
	LoA Spread	0.49 [0.44 – 0.53]	0.51 [0.46 – 0.56]	0.51 [0.45 – 0.56]	0.52 [0.46 – 0.56]	0.52 [0.46 – 0.56]	0.53 [0.46 – 0.56]	0.53 [0.49 – 0.58]	0.52 [0.49 – 0.58]	0.52 [0.48 – 0.57]
	Relative Bias	-0.13 [-0.2 – -0.04]	-0.15 [-0.24 – -0.04]	-0.16 [-0.25 – -0.03]	-0.18 [-0.27 – -0.03]	-0.17 [-0.28 – -0.05]	-0.19 [-0.29 – -0.03]	-0.21 [-0.3 – -0.03]	-0.22 [-0.31 – -0.06]	-0.24 [-0.29 – -0.12]
	Regression Slope	1.3 [1.1 – 1.48]	1.5 [1.3 – 1.68]	1.6 [1.4 – 1.7]	1.7 [1.6 – 1.8]	1.8 [1.6 – 1.9]	1.9 [1.7 – 1.9]	1.9 [1.8 – 2]	2 [1.9 – 2]	2 [1.9 – 2]
	Regression Intercept	0 [-0.01 – 0.01]	0 [-0.02 – 0.02]	0 [-0.02 – 0.01]	0 [-0.02 – 0.02]	0 [-0.02 – 0.02]	0 [-0.03 – 0.02]	0 [-0.04 – 0.03]	0 [-0.03 – 0.04]	-0.03 [-0.06 – 0.01]
COx-a_R	Bias	-0.14 [-1.4 – 1.31]	-0.17 [-1.63 – 1.55]	-0.11 [-1.88 – 1.38]	-0.1 [-1.86 – 1.66]	0.02 [-2.17 – 1.84]	-0.19 [-2.73 – 2.46]	0.08 [-3.46 – 2.56]	-0.04 [-3.19 – 3.94]	-2.62 [-5.69 – 1.09]
	Lower LoA	0.96 [0.87 – 1.05]	0.99 [0.9 – 1.09]	0.99 [0.9 – 1.11]	1 [0.9 – 1.1]	1.02 [0.91 – 1.11]	1.02 [0.91 – 1.12]	1.04 [0.93 – 1.13]	1.02 [0.94 – 1.12]	1.02 [0.99 – 1.1]
	Upper LoA	-0.48 [-0.54 – -0.43]	-0.5 [-0.56 – -0.44]	-0.5 [-0.56 – -0.45]	-0.51 [-0.57 – -0.45]	-0.51 [-0.57 – -0.45]	-0.52 [-0.57 – -0.45]	-0.52 [-0.57 – -0.47]	-0.51 [-0.54 – -0.45]	-0.55 [-0.59 – -0.49]
	LoA Spread	0.48 [0.44 – 0.53]	0.49 [0.44 – 0.55]	0.5 [0.45 – 0.55]	0.51 [0.45 – 0.56]	0.51 [0.46 – 0.56]	0.51 [0.46 – 0.56]	0.51 [0.44 – 0.57]	0.51 [0.45 – 0.58]	0.49 [0.44 – 0.57]
	Relative Bias	-0.09 [-0.19 – -0.02]	-0.1 [-0.23 – -0.02]	-0.1 [-0.23 – -0.02]	-0.13 [-0.25 – -0.03]	-0.13 [-0.27 – -0.03]	-0.13 [-0.29 – -0.03]	-0.15 [-0.29 – -0.04]	-0.19 [-0.29 – -0.04]	-0.28 [-0.37 – -0.13]
	Regression Slope	1.2 [1.1 – 1.3]	1.4 [1.3 – 1.5]	1.6 [1.4 – 1.7]	1.7 [1.5 – 1.8]	1.8 [1.6 – 1.9]	1.9 [1.7 – 1.9]	1.9 [1.9 – 2]	2 [1.9 – 2]	2 [1.9 – 2]
	Regression Intercept	-0.03 [-0.13 – 0.04]	-0.05 [-0.17 – 0.05]	-0.03 [-0.17 – 0.06]	-0.04 [-0.18 – 0.06]	-0.04 [-0.22 – 0.06]	-0.08 [-0.23 – 0.04]	-0.07 [-0.2 – 0.09]	-0.07 [-0.19 – 0.12]	-0.12 [-0.34 – -0.01]

COx, cerebral oximetry index with cerebral perfusion pressure; COx-a, cerebral oximetry index with arterial blood pressure; HC, healthy control volunteer group; IQR, interquartile range; r-value, Pearson correlation coefficient; rSO_2, regional cerebral oxygen saturation; SP, elective spinal surgery patient group; TBI, traumatic brain injury patient group.

File S8h: Windowed-Point – Bland-Altman Analysis of rSO_2 and COx/COx-a in All Populations using 1-Minute Temporal Resolution

Physiologic Variable	Value	Median [IQR]								
		5-Minute Interval	10-Minute Interval	15-Minute Interval	30-Minute Interval	1-Hour Interval	2-Hour Interval	6-Hour Interval	12-Hour Interval	1-Day Interval
**HC Population**										
rSO_2_L	Bias	-0.03 [-0.22 – 0.1]	0.01 [-0.13 – 0.11]	0.01 [-0.13 – 0.2]	0.11 [-0.2 – 0.49]	–	–	–	–	–
	Lower LoA	-2.47 [-3.48 – -1.75]	-2.15 [-2.85 – -1.64]	-1.86 [-2.43 – -1.48]	-1.68 [-2.14 – -1.02]	–	–	–	–	–
	Upper LoA	2.46 [1.8 – 3.27]	2.1 [1.69 – 2.98]	1.91 [1.41 – 2.61]	1.87 [1.31 – 2.45]	–	–	–	–	–
	LoA Spread	4.88 [3.57 – 6.61]	4.33 [3.21 – 5.74]	3.84 [2.94 – 5.03]	3.32 [2.63 – 4.58]	–	–	–	–	–
	Relative Bias	-0.78 [-3.48 – 2.01]	0.15 [-3.33 – 2.55]	0.19 [-3.7 – 5.9]	2.84 [-4.69 – 11.36]	–	–	–	–	–
	Regression Slope	-0.33 [-0.55 – -0.15]	-0.09 [-0.33 – 0.06]	0.05 [-0.15 – 0.31]	0.37 [-0.13 – 0.63]	–	–	–	–	–
	Regression Intercept	22 [7.95 – 42]	6.65 [-3.98 – 23.5]	-4.05 [-21.5 – 9.3]	-15 [-39.75 – 1.53]	–	–	–	–	–
rSO_2_R	Bias	-0.01 [-0.18 – 0.13]	0 [-0.11 – 0.15]	0.03 [-0.13 – 0.21]	0.07 [-0.25 – 0.43]	–	–	–	–	–
	Lower LoA	-2.78 [-3.74 – -2.16]	-2.37 [-3.13 – -1.87]	-2.1 [-2.84 – -1.66]	-1.64 [-2.01 – -1.09]	–	–	–	–	–
	Upper LoA	2.85 [2.12 – 3.41]	2.48 [1.92 – 3.26]	2.26 [1.64 – 2.95]	1.91 [1.38 – 2.34]	–	–	–	–	–
	LoA Spread	5.7 [4.25 – 7.18]	4.79 [3.87 – 6.32]	4.39 [3.55 – 5.78]	3.39 [2.55 – 4.58]	–	–	–	–	–
	Relative Bias	-0.22 [-2.92 – 2.41]	-0.02 [-2.53 – 3.22]	0.85 [-3.49 – 4.45]	1.16 [-5.31 – 10.63]	–	–	–	–	–
	Regression Slope	-0.29 [-0.51 – -0.1]	-0.11 [-0.38 – 0.08]	-0.05 [-0.25 – 0.21]	0.34 [-0.09 – 0.96]	–	–	–	–	–
	Regression Intercept	22 [6.4 – 39]	7.5 [-6.08 – 27.75]	2.85 [-14.5 – 17.75]	-7.4 [-52.75 – 1.4]	–	–	–	–	–
COx_L	Bias	–	–	–	–	–	–	–	–	–
	Lower LoA	–	–	–	–	–	–	–	–	–
	Upper LoA	–	–	–	–	–	–	–	–	–
	LoA Spread	–	–	–	–	–	–	–	–	–
	Relative Bias	–	–	–	–	–	–	–	–	–
	Regression Slope	–	–	–	–	–	–	–	–	–
	Regression Intercept	–	–	–	–	–	–	–	–	–
COx_R	Bias	–	–	–	–	–	–	–	–	–
	Lower LoA	–	–	–	–	–	–	–	–	–
	Upper LoA	–	–	–	–	–	–	–	–	–
	LoA Spread	–	–	–	–	–	–	–	–	–
	Relative Bias	–	–	–	–	–	–	–	–	–
	Regression Slope	–	–	–	–	–	–	–	–	–
	Regression Intercept	–	–	–	–	–	–	–	–	–
COx-a_L	Bias	0 [-0.03 – 0.03]	0 [-0.02 – 0.03]	0 [-0.03 – 0.03]	-0.02 [-0.08 – 0.02]	–	–	–	–	–
	Lower LoA	-0.53 [-0.64 – -0.41]	-0.42 [-0.52 – -0.33]	-0.36 [-0.44 – -0.29]	-0.35 [-0.46 – -0.29]	–	–	–	–	–
	Upper LoA	0.08 [-2.35 – 2.88]	-0.44 [-3.08 – 3]	-0.36 [-4.34 – 4.57]	-2.94 [-10.29 – 2.42]	–	–	–	–	–
	LoA Spread	1.04 [0.82 – 1.31]	0.82 [0.65 – 1.05]	0.71 [0.56 – 0.91]	0.63 [0.51 – 0.81]	–	–	–	–	–
	Relative Bias	0.53 [0.41 – 0.68]	0.42 [0.32 – 0.53]	0.36 [0.27 – 0.44]	0.3 [0.24 – 0.39]	–	–	–	–	–
	Regression Slope	0.04 [-0.01 – 0.14]	0.01 [-0.03 – 0.04]	0.01 [-0.04 – 0.05]	0 [-0.1 – 0.07]	–	–	–	–	–
	Regression Intercept	-0.28 [-0.5 – -0.14]	-0.1 [-0.31 – -0.03]	-0.07 [-0.19 – 0.03]	-0.18 [-0.57 – 0.19]	–	–	–	–	–
COx-a_R	Bias	0 [-0.04 – 0.03]	0 [-0.03 – 0.03]	0 [-0.02 – 0.03]	-0.02 [-0.09 – 0.03]	–	–	–	–	–
	Lower LoA	-0.48 [-0.63 – -0.39]	-0.41 [-0.51 – -0.33]	-0.37 [-0.46 – -0.3]	-0.37 [-0.46 – -0.31]	–	–	–	–	–
	Upper LoA	-0.17 [-3.32 – 3.1]	-0.07 [-3.29 – 3]	0.13 [-3.68 – 2.96]	-3.62 [-9.96 – 3.76]	–	–	–	–	–
	LoA Spread	0.99 [0.79 – 1.26]	0.82 [0.66 – 1.03]	0.75 [0.58 – 0.94]	0.66 [0.48 – 0.83]	–	–	–	–	–
	Relative Bias	0.49 [0.39 – 0.61]	0.42 [0.32 – 0.51]	0.38 [0.28 – 0.45]	0.3 [0.22 – 0.39]	–	–	–	–	–
	Regression Slope	0.01 [-0.02 – 0.07]	0.01 [-0.03 – 0.05]	0 [-0.03 – 0.05]	-0.02 [-0.22 – 0.03]	–	–	–	–	–
	Regression Intercept	-0.31 [-0.5 – -0.15]	-0.16 [-0.34 – -0.01]	-0.1 [-0.24 – 0.09]	-0.08 [-0.61 – 0.47]	–	–	–	–	–
**SP Population**										
rSO_2_L	Bias	0.06 [-0.09 – 0.12]	0.03 [-0.06 – 0.13]	0.02 [-0.1 – 0.09]	0.05 [-0.04 – 0.07]	0.02 [-0.01 – 0.08]	0.12 [0.04 – 0.2]	-0.66 [-0.73 – -0.58]	–	–
	Lower LoA	-3.53 [-7.31 – -2.17]	-3.19 [-7.62 – -2.35]	-2.75 [-5.34 – -2.18]	-2.13 [-3.96 – -1.71]	-1.68 [-3.46 – -1.12]	-1.7 [-3.72 – -1.24]	-16.55 [-20.87 – -12.23]	–	–
	Upper LoA	3.66 [2.45 – 7.22]	3.12 [2.41 – 7.6]	2.58 [2.24 – 6.18]	2.19 [1.76 – 4.06]	1.89 [1.19 – 3.55]	2.31 [1.42 – 3.95]	15.23 [10.76 – 19.7]	–	–
	LoA Spread	6.86 [4.61 – 14.67]	6.31 [4.72 – 15.23]	5.33 [4.45 – 11.5]	4.31 [3.49 – 7.87]	3.56 [2.27 – 7.01]	4.03 [2.7 – 7.7]	31.78 [22.99 – 40.57]	–	–
	Relative Bias	1.07 [-1.05 – 2.2]	0.6 [-0.73 – 2.42]	0.31 [-1.48 – 1.87]	0.9 [-0.59 – 1.95]	0.76 [-0.18 – 2.43]	2.18 [1.19 – 5.53]	-3.37 [-4.54 – -2.2]	–	–

	Regression Slope	-0.05 [-0.17 – -0.02]	-0.07 [-0.18 – -0.03]	-0.07 [-0.16 – -0.04]	-0.04 [-0.14 – -0.01]	-0.02 [-0.11 – 0.01]	0.02 [-0.02 – 0.04]	0.67 [0.31 – 1.04]	–	–
	Regression Intercept	3.6 [1.07 – 9.15]	4.3 [2.45 – 9.55]	4.5 [2.15 – 11]	2.5 [0.93 – 7.1]	1.05 [-1.01 – 7.58]	-1.1 [-2.4 – 1.46]	-53.35 [-81.68 – -25.03]	–	–
rSO_2_R	Bias	0 [-0.06 – 0.11]	0.05 [-0.01 – 0.21]	-0.02 [-0.16 – 0.04]	0.04 [0 – 0.07]	0.04 [0 – 0.06]	0.09 [0.04 – 0.18]	-0.24 [-0.45 – -0.02]	–	–
	Lower LoA	-2.96 [-5.24 – -2.22]	-2.8 [-4.25 – -2.12]	-2.68 [-7.09 – -1.91]	-2.34 [-4.01 – -1.68]	-1.69 [-2.43 – -1.14]	-2.03 [-2.83 – -1]	-22.26 [-25.15 – -19.36]	–	–
	Upper LoA	3.14 [2.24 – 5.21]	2.89 [2.29 – 4.55]	2.61 [1.95 – 6.85]	2.52 [1.74 – 4.54]	1.62 [1.22 – 2.39]	2.23 [1.11 – 2.74]	21.78 [19.32 – 24.25]	–	–
	LoA Spread	6.07 [4.46 – 10.45]	5.65 [4.4 – 8.72]	5.29 [3.77 – 13.95]	4.77 [3.41 – 8.62]	3.27 [2.34 – 4.72]	4.26 [2.24 – 5.5]	44.04 [38.69 – 49.4]	–	–
	Relative Bias	0.2 [-0.66 – 1.69]	1.21 [-0.09 – 2.21]	-0.37 [-1.84 – 0.79]	0.52 [-0.14 – 2.12]	1.39 [0.12 – 2.1]	3.02 [0.69 – 5.45]	-0.32 [-0.77 – 0.13]	–	–
	Regression Slope	-0.05 [-0.13 – 0]	-0.04 [-0.12 – -0.01]	-0.06 [-0.16 – -0.03]	-0.05 [-0.09 – -0.01]	-0.01 [-0.07 – 0.02]	0.01 [-0.04 – 0.06]	0.03 [0.02 – 0.04]	–	–
	Regression Intercept	3.1 [0.03 – 9.15]	3.2 [0.87 – 8.2]	3.9 [1.9 – 10.9]	3.7 [0.86 – 5.55]	0.64 [-1.1 – 4.13]	-0.4 [-3.35 – 2.55]	-2.15 [-2.48 – -1.83]	–	–
COx_L	Bias	–	–	–	–	–	–	–	–	–
	Lower LoA	–	–	–	–	–	–	–	–	–
	Upper LoA	–	–	–	–	–	–	–	–	–
	LoA Spread	–	–	–	–	–	–	–	–	–
	Relative Bias	–	–	–	–	–	–	–	–	–
	Regression Slope	–	–	–	–	–	–	–	–	–
	Regression Intercept	–	–	–	–	–	–	–	–	–
COx_R	Bias	–	–	–	–	–	–	–	–	–
	Lower LoA	–	–	–	–	–	–	–	–	–
	Upper LoA	–	–	–	–	–	–	–	–	–
	LoA Spread	–	–	–	–	–	–	–	–	–
	Relative Bias	–	–	–	–	–	–	–	–	–
	Regression Slope	–	–	–	–	–	–	–	–	–
	Regression Intercept	–	–	–	–	–	–	–	–	–
COx-a_L	Bias	0.02 [-0.01 – 0.03]	0.01 [0 – 0.01]	0.01 [0 – 0.02]	0 [0 – 0.01]	0 [0 – 0.01]	0.01 [-0.01 – 0.01]	-0.02 [-0.02 – -0.02]	–	–
	Lower LoA	-0.67 [-0.81 – -0.58]	-0.61 [-0.78 – -0.55]	-0.58 [-0.68 – -0.49]	-0.48 [-0.56 – -0.41]	-0.44 [-0.5 – -0.37]	-0.43 [-0.55 – -0.37]	-0.45 [-0.45 – -0.45]	–	–
	Upper LoA	1.29 [-0.71 – 2.42]	0.64 [-0.29 – 1.11]	0.48 [-0.04 – 1.23]	0.08 [-0.49 – 0.9]	-0.03 [-0.4 – 0.62]	0.62 [-0.98 – 1.52]	-2.82 [-2.82 – -2.82]	–	–
	LoA Spread	1.36 [1.2 – 1.67]	1.24 [1.1 – 1.56]	1.17 [1 – 1.4]	0.94 [0.83 – 1.13]	0.89 [0.74 – 1.05]	0.88 [0.74 – 1.11]	0.84 [0.84 – 0.84]	–	–
	Relative Bias	0.68 [0.61 – 0.86]	0.64 [0.55 – 0.78]	0.59 [0.51 – 0.72]	0.47 [0.42 – 0.57]	0.45 [0.37 – 0.52]	0.46 [0.37 – 0.55]	0.4 [0.4 – 0.4]	–	–
	Regression Slope	0.06 [0.02 – 0.07]	0.02 [0.01 – 0.04]	0.02 [0.01 – 0.03]	0 [-0.01 – 0.01]	-0.02 [-0.02 – 0]	-0.03 [-0.04 – -0.01]	-0.02 [-0.02 – -0.02]	–	–
	Regression Intercept	-0.22 [-0.29 – -0.15]	-0.13 [-0.22 – -0.09]	-0.09 [-0.12 – -0.05]	0.01 [-0.03 – 0.07]	0.08 [0.05 – 0.13]	0.14 [0.09 – 0.19]	0.12 [0.12 – 0.12]	–	–
COx-a_R	Bias	0.01 [-0.01 – 0.03]	0.01 [0 – 0.02]	0 [0 – 0.01]	0 [0 – 0.01]	0 [0 – 0.01]	-0.01 [-0.02 – 0.01]	0.01 [0 – 0.01]	–	–
	Lower LoA	-0.69 [-0.81 – -0.62]	-0.63 [-0.71 – -0.56]	-0.6 [-0.62 – -0.52]	-0.52 [-0.58 – -0.45]	-0.43 [-0.49 – -0.38]	-0.39 [-0.51 – -0.36]	-0.4 [-0.42 – -0.38]	–	–
	Upper LoA	0.45 [-0.76 – 2.3]	0.73 [-0.2 – 1.2]	0.39 [-0.33 – 0.79]	0.33 [-0.26 – 1.08]	0.28 [-0.36 – 0.99]	-0.68 [-2.99 – 0.9]	0.77 [0.38 – 1.17]	–	–
	LoA Spread	1.39 [1.26 – 1.66]	1.27 [1.15 – 1.44]	1.19 [1.06 – 1.26]	1.04 [0.89 – 1.18]	0.88 [0.77 – 0.99]	0.83 [0.72 – 1.01]	0.81 [0.77 – 0.85]	–	–
	Relative Bias	0.71 [0.63 – 0.84]	0.64 [0.59 – 0.72]	0.61 [0.54 – 0.64]	0.53 [0.44 – 0.59]	0.44 [0.39 – 0.5]	0.44 [0.34 – 0.5]	0.41 [0.39 – 0.44]	–	–
	Regression Slope	0.06 [0 – 0.08]	0.02 [0 – 0.06]	0.01 [0 – 0.03]	0 [-0.01 – 0.01]	-0.01 [-0.03 – 0]	-0.03 [-0.06 – -0.01]	-0.02 [-0.04 – 0]	–	–
	Regression Intercept	-0.22 [-0.31 – -0.16]	-0.14 [-0.19 – -0.09]	-0.06 [-0.12 – -0.02]	0.02 [-0.03 – 0.05]	0.08 [0.04 – 0.12]	0.14 [0.08 – 0.2]	0.18 [0.17 – 0.18]	–	–
**TBI Population**										
rSO_2_L	Bias	0.01 [-0.03 – 0.05]	-0.01 [-0.04 – 0.04]	0 [-0.03 – 0.03]	0 [-0.02 – 0.03]	0 [-0.03 – 0.04]	0 [-0.04 – 0.04]	0 [-0.01 – 0.08]	0 [-0.01 – 0.03]	0 [-0.01 – 0.01]
	Lower LoA	-3.37 [-6.65 – -2.33]	-4.24 [-7.78 – -2.64]	-3.79 [-8.11 – -2.43]	-3.76 [-8.97 – -2.38]	-4.07 [-13.39 – -2.24]	-3.85 [-44516.96 – -2.05]	-2.83 [-257736761.29 – -1.79]	-2.63 [-19.47 – -1.72]	-2.66 [-5.34 – -1.78]
	Upper LoA	3.35 [2.28 – 6.77]	4.1 [2.72 – 7.77]	3.86 [2.45 – 8.19]	3.63 [2.37 – 9]	4 [2.22 – 13.38]	3.83 [2.07 – 44897.32]	2.85 [1.83 – 244555748.62]	2.66 [1.71 – 20.05]	2.67 [1.77 – 5.33]
	LoA Spread	6.72 [4.63 – 13.42]	8.37 [5.4 – 15.54]	7.61 [4.88 – 16.31]	7.37 [4.75 – 17.97]	8.07 [4.46 – 26.77]	7.71 [4.08 – 89414.28]	5.68 [3.62 – 502292509.91]	5.29 [3.44 – 39.52]	5.33 [3.54 – 10.67]

	Relative Bias	0.05 [-0.32 – 0.62]	-0.06 [-0.56 – 0.37]	0.01 [-0.4 – 0.48]	0.01 [-0.35 – 0.32]	0.02 [-0.35 – 0.35]	-0.07 [-0.28 – 0.33]	0.02 [-0.29 – 0.3]	-0.06 [-0.3 – 0.28]	-0.01 [-0.24 – 0.12]
	Regression Slope	-0.02 [-0.07 – -0.01]	-0.04 [-0.14 – -0.02]	-0.04 [-0.12 – -0.02]	-0.03 [-0.08 – -0.01]	-0.02 [-0.39 – 0]	-0.01 [-2 – 0]	0 [-2 – 0.01]	0 [-0.91 – 0.01]	0.01 [0 – 0.02]
	Regression Intercept	1.65 [0.55 – 4.38]	2.9 [1.2 – 9.5]	2.4 [0.94 – 8.1]	2.1 [0.61 – 5.53]	1.6 [0.27 – 23]	0.83 [0.03 – 98.5]	-0.16 [-0.77 – 4.7]	-0.18 [-0.96 – 3.65]	-0.48 [-1.2 – 0.05]
rSO_2_R	Bias	0.01 [-0.02 – 0.06]	-0.01 [-0.05 – 0.05]	0 [-0.04 – 0.05]	0 [-0.02 – 0.03]	0 [-0.04 – 0.05]	0 [-0.03 – 0.02]	0 [-0.04 – 0.02]	0 [-0.01 – 0.03]	0 [-0.03 – 0.01]
	Lower LoA	-3.34 [-6.48 – -2.04]	-4.56 [-7.81 – -2.4]	-4.62 [-9.31 – -2.49]	-4.3 [-8.42 – -2.29]	-4.76 [-22.85 – -2.18]	-4.41 [-1336.93 – -1.84]	-3.59 [-1208353229.57 – -1.66]	-3.78 [-9.25 – -1.77]	-3.8 [-7.5 – -1.65]
	Upper LoA	3.24 [2.06 – 6.65]	4.57 [2.47 – 7.89]	4.65 [2.45 – 9.73]	4.31 [2.23 – 8.5]	4.79 [2.14 – 23.09]	4.42 [1.89 – 1314.62]	3.58 [1.55 – 1194404861.08]	3.91 [1.78 – 9.41]	3.85 [1.64 – 7.57]
	LoA Spread	6.66 [4.11 – 13.13]	9.14 [4.87 – 15.7]	9.24 [4.96 – 19.08]	8.61 [4.53 – 16.91]	9.55 [4.31 – 45.95]	8.83 [3.73 – 2651.55]	7.17 [3.26 – 2402758090.65]	7.76 [3.55 – 18.57]	7.65 [3.29 – 15.07]
	Relative Bias	0.24 [-0.3 – 0.72]	-0.15 [-0.63 – 0.48]	0.03 [-0.53 – 0.52]	0 [-0.35 – 0.36]	-0.03 [-0.4 – 0.38]	-0.03 [-0.38 – 0.35]	-0.07 [-0.36 – 0.31]	0.07 [-0.26 – 0.33]	0.01 [-0.32 – 0.22]
	Regression Slope	-0.03 [-0.07 – -0.01]	-0.06 [-0.1 – -0.02]	-0.04 [-0.12 – -0.02]	-0.04 [-0.12 – -0.01]	-0.02 [-1.48 – 0]	-0.01 [-2 – 0]	0 [-2 – 0.01]	0 [-0.02 – 0.02]	0.01 [-0.02 – 0.01]
	Regression Intercept	2 [0.76 – 4.88]	3.6 [1.2 – 7.08]	3.15 [1.23 – 8.83]	2.8 [0.49 – 8.28]	0.98 [0.14 – 110]	0.51 [-0.08 – 76.75]	-0.13 [-1.05 – 0.99]	-0.35 [-1.5 – 0.23]	-0.51 [-2.03 – 0.05]
COx_L	Bias	0 [0 – 0.01]	0 [0 – 0]	0 [0 – 0]	0 [0 – 0]	0 [0 – 0]	0 [0 – 0]	0 [0 – 0]	0 [0 – 0]	0 [0 – 0]
	Lower LoA	-0.56 [-0.61 – -0.49]	-0.56 [-0.65 – -0.47]	-0.5 [-0.59 – -0.43]	-0.42 [-0.47 – -0.39]	-0.38 [-0.41 – -0.35]	-0.35 [-0.38 – -0.33]	-0.34 [-0.36 – -0.32]	-0.33 [-0.36 – -0.31]	-0.33 [-0.35 – -0.3]
	Upper LoA	0.57 [0.49 – 0.61]	0.56 [0.46 – 0.65]	0.49 [0.44 – 0.59]	0.42 [0.39 – 0.47]	0.37 [0.35 – 0.41]	0.35 [0.33 – 0.38]	0.34 [0.32 – 0.36]	0.33 [0.31 – 0.36]	0.33 [0.3 – 0.36]
	LoA Spread	1.13 [0.98 – 1.23]	1.13 [0.93 – 1.3]	0.99 [0.87 – 1.18]	0.84 [0.78 – 0.93]	0.75 [0.7 – 0.83]	0.7 [0.66 – 0.76]	0.68 [0.64 – 0.72]	0.67 [0.62 – 0.72]	0.66 [0.6 – 0.71]
	Relative Bias	0.23 [-0.29 – 0.68]	0.11 [-0.29 – 0.32]	0.04 [-0.15 – 0.24]	0.02 [-0.12 – 0.15]	-0.06 [-0.2 – 0.06]	0 [-0.13 – 0.11]	-0.01 [-0.13 – 0.1]	0.05 [-0.11 – 0.21]	0.09 [-0.06 – 0.2]
	Regression Slope	-0.22 [-0.25 – -0.17]	-0.17 [-0.25 – -0.12]	-0.1 [-0.16 – -0.07]	-0.01 [-0.03 – 0.01]	0.06 [0.04 – 0.07]	0.11 [0.08 – 0.12]	0.14 [0.12 – 0.16]	0.15 [0.13 – 0.17]	0.15 [0.13 – 0.17]
	Regression Intercept	0.01 [0 – 0.02]	0.01 [0 – 0.02]	0 [0 – 0.01]	0 [0 – 0]	0 [0 – 0]	0 [-0.01 – 0]	0 [-0.01 – 0]	0 [-0.01 – 0]	-0.01 [-0.02 – 0]
COx_R	Bias	0 [0 – 0.01]	0 [0 – 0]	0 [0 – 0]	0 [0 – 0]	0 [0 – 0]	0 [0 – 0]	0 [0 – 0]	0 [0 – 0]	0 [0 – 0]
	Lower LoA	-0.54 [-0.6 – -0.48]	-0.54 [-0.64 – -0.48]	-0.5 [-0.59 – -0.44]	-0.42 [-0.49 – -0.39]	-0.38 [-0.42 – -0.36]	-0.35 [-0.38 – -0.33]	-0.33 [-0.36 – -0.31]	-0.33 [-0.36 – -0.3]	-0.32 [-0.36 – -0.3]
	Upper LoA	0.55 [0.48 – 0.61]	0.54 [0.48 – 0.62]	0.5 [0.44 – 0.58]	0.42 [0.39 – 0.49]	0.38 [0.36 – 0.43]	0.35 [0.33 – 0.38]	0.34 [0.31 – 0.36]	0.33 [0.3 – 0.36]	0.32 [0.3 – 0.36]
	LoA Spread	1.1 [0.96 – 1.22]	1.09 [0.97 – 1.26]	1 [0.87 – 1.18]	0.84 [0.78 – 0.97]	0.76 [0.71 – 0.85]	0.7 [0.66 – 0.76]	0.67 [0.62 – 0.73]	0.66 [0.61 – 0.73]	0.65 [0.6 – 0.72]
	Relative Bias	0.37 [-0.24 – 0.96]	0.08 [-0.21 – 0.38]	-0.01 [-0.23 – 0.27]	0.01 [-0.14 – 0.15]	0 [-0.1 – 0.19]	0 [-0.1 – 0.11]	-0.01 [-0.15 – 0.2]	-0.03 [-0.2 – 0.2]	0.04 [-0.18 – 0.24]
	Regression Slope	-0.2 [-0.24 – -0.12]	-0.19 [-0.28 – -0.1]	-0.1 [-0.17 – -0.07]	-0.01 [-0.04 – 0.01]	0.05 [0.04 – 0.08]	0.1 [0.08 – 0.12]	0.14 [0.12 – 0.15]	0.15 [0.13 – 0.16]	0.15 [0.13 – 0.17]
	Regression Intercept	0 [0 – 0.01]	0 [0 – 0]	0 [0 – 0]	0 [0 – 0]	0 [0 – 0]	0 [0 – 0]	0 [0 – 0]	0 [0 – 0]	0 [0 – 0]
COx-a_L	Bias	0.5 [-0.1 – 0.98]	0.22 [-0.02 – 0.44]	0.12 [-0.01 – 0.35]	0.05 [-0.12 – 0.2]	0.04 [-0.06 – 0.16]	0.03 [-0.11 – 0.14]	-0.01 [-0.12 – 0.13]	0.04 [-0.11 – 0.19]	0.09 [-0.08 – 0.25]
	Lower LoA	1.07 [0.98 – 1.2]	1.09 [0.92 – 1.22]	0.98 [0.87 – 1.1]	0.81 [0.77 – 0.91]	0.74 [0.69 – 0.8]	0.69 [0.65 – 0.77]	0.66 [0.62 – 0.72]	0.65 [0.6 – 0.71]	0.64 [0.6 – 0.7]
	Upper LoA	-0.54 [-0.59 – -0.48]	-0.54 [-0.61 – -0.46]	-0.49 [-0.55 – -0.43]	-0.4 [-0.45 – -0.38]	-0.37 [-0.4 – -0.35]	-0.35 [-0.38 – -0.33]	-0.33 [-0.36 – -0.31]	-0.33 [-0.35 – -0.3]	-0.32 [-0.35 – -0.3]
	LoA Spread	0.54 [0.5 – 0.61]	0.55 [0.46 – 0.61]	0.49 [0.43 – 0.55]	0.4 [0.38 – 0.46]	0.37 [0.35 – 0.4]	0.35 [0.33 – 0.38]	0.33 [0.31 – 0.36]	0.33 [0.3 – 0.36]	0.33 [0.3 – 0.35]
	Relative Bias	0.02 [0.01 – 0.03]	0.02 [0.01 – 0.03]	0.01 [0 – 0.02]	0 [0 – 0]	0 [-0.01 – 0]	-0.01 [-0.01 – 0]	-0.01 [-0.02 – 0]	-0.01 [-0.02 – 0]	-0.02 [-0.02 – -0.01]
	Regression Slope	-0.21 [-0.24 – -0.15]	-0.18 [-0.28 – -0.11]	-0.1 [-0.16 – -0.07]	-0.01 [-0.03 – 0.01]	0.05 [0.04 – 0.07]	0.1 [0.08 – 0.12]	0.15 [0.12 – 0.16]	0.15 [0.14 – 0.17]	0.16 [0.14 – 0.17]
	Regression Intercept	0 [0 – 0.01]	0 [0 – 0]	0 [0 – 0]	0 [0 – 0]	0 [0 – 0]	0 [0 – 0]	0 [0 – 0]	0 [0 – 0]	0 [0 – 0]
COx-a_R	Bias	0.37 [-0.06 – 1.04]	0.15 [-0.14 – 0.46]	0.07 [-0.15 – 0.26]	0.07 [-0.15 – 0.26]	-0.01 [-0.17 – 0.09]	0.03 [-0.11 – 0.15]	0 [-0.18 – 0.16]	0 [-0.2 – 0.19]	0.02 [-0.1 – 0.24]
	Lower LoA	1.08 [0.97 – 1.19]	1.12 [0.95 – 1.29]	1.01 [0.9 – 1.14]	0.85 [0.76 – 0.9]	0.76 [0.71 – 0.81]	0.7 [0.65 – 0.75]	0.66 [0.62 – 0.72]	0.66 [0.59 – 0.72]	0.64 [0.59 – 0.71]
	Upper LoA	-0.53 [-0.59 – -0.48]	-0.56 [-0.63 – -0.48]	-0.5 [-0.58 – -0.45]	-0.42 [-0.45 – -0.38]	-0.38 [-0.4 – -0.36]	-0.35 [-0.37 – -0.33]	-0.33 [-0.36 – -0.31]	-0.33 [-0.36 – -0.3]	-0.32 [-0.36 – -0.29]
	LoA Spread	0.54 [0.49 – 0.6]	0.56 [0.47 – 0.65]	0.51 [0.45 – 0.56]	0.42 [0.38 – 0.45]	0.38 [0.35 – 0.41]	0.35 [0.32 – 0.38]	0.33 [0.31 – 0.36]	0.33 [0.3 – 0.36]	0.32 [0.29 – 0.35]
	Relative Bias	0.02 [0 – 0.04]	0.01 [0 – 0.03]	0.01 [0 – 0.02]	0 [0 – 0]	0 [-0.01 – 0]	-0.01 [-0.01 – 0]	-0.01 [-0.02 – 0]	-0.01 [-0.02 – 0]	-0.01 [-0.02 – 0]
	Regression Slope	-0.22 [-0.25 – -0.14]	-0.2 [-0.31 – -0.12]	-0.14 [-0.21 – -0.07]	-0.01 [-0.03 – 0.01]	0.05 [0.04 – 0.06]	0.1 [0.09 – 0.11]	0.14 [0.13 – 0.16]	0.16 [0.14 – 0.16]	0.16 [0.14 – 0.17]
	Regression Intercept	0.01 [-0.01 – 0.02]	0 [-0.01 – 0.01]	0 [-0.01 – 0.01]	0 [0 – 0]	0 [-0.01 – 0]	0 [-0.01 – 0]	0 [-0.02 – 0.01]	0 [-0.02 – 0.01]	0 [-0.02 – 0.01]

COx, cerebral oximetry index with cerebral perfusion pressure; COx-a, cerebral oximetry index with arterial blood pressure; HC, healthy control volunteer group; IQR, interquartile range; r-value, Pearson correlation coefficient; rSO_2, regional cerebral oxygen saturation; SP, elective spinal surgery patient group; TBI, traumatic brain injury patient group.

File S8i: Windowed-Point – Bland-Altman Analysis of rSO$_2$ and COx/COx-a in All Populations using 5-Minute Temporal Resolution

Physiologic Variable	Value	Median [IQR]								
		5-Minute Interval	10-Minute Interval	15-Minute Interval	30-Minute Interval	1-Hour Interval	2-Hour Interval	6-Hour Interval	12-Hour Interval	1-Day Interval
**HC Population**										
rSO$_2$_L	Bias	0.65 [0.65 – 0.65]	0 [-0.42 – 0.27]	-0.01 [-0.49 – 0.47]	-0.22 [-1.27 – 1.4]	–	–	–	–	–
	Lower LoA	0.15 [0.15 – 0.15]	-1.81 [-2.68 – -1.23]	-2.2 [-3.38 – -1.09]	-4.35 [-7.92 – -3.27]	–	–	–	–	–
	Upper LoA	1.15 [1.15 – 1.15]	1.94 [1.22 – 2.62]	2.22 [1.2 – 3.38]	4.09 [1.36 – 6.67]	–	–	–	–	–
	LoA Spread	1 [1 – 1]	3.74 [2.42 – 5.1]	4.2 [2.91 – 6.44]	8.42 [4.9 – 16.67]	–	–	–	–	–
	Relative Bias	64.61 [64.61 – 64.61]	0.3 [-8.12 – 7.73]	-0.17 [-7.39 – 12.42]	-5.94 [-18.12 – 5.7]	–	–	–	–	–
	Regression Slope	24 [24 – 24]	-0.26 [-0.92 – 0.1]	-0.73 [-1.88 – 1.04]	-2 [-4.2 – -1.6]	–	–	–	–	–
	Regression Intercept	-1.2 [-1.2 – -1.2]	17.5 [-7 – 67]	17 [-59.5 – 120]	1.45 [-0.76 – 30.05]	–	–	–	–	–
rSO$_2$_R	Bias	0.65 [0.65 – 0.65]	-0.16 [-0.53 – 0.25]	0.01 [-0.58 – 0.43]	0.44 [-0.17 – 1.65]	–	–	–	–	–
	Lower LoA	0.15 [0.15 – 0.15]	-2.05 [-3.14 – -1.42]	-2.24 [-3.82 – -1.42]	-3.93 [-4.45 – -3.42]	–	–	–	–	–
	Upper LoA	1.15 [1.15 – 1.15]	1.8 [1.13 – 2.65]	2.38 [1.1 – 3.51]	4.75 [3.92 – 4.94]	–	–	–	–	–
	LoA Spread	1 [1 – 1]	3.82 [2.67 – 5.59]	4.5 [2.33 – 7.23]	8.69 [5.81 – 9.48]	–	–	–	–	–
	Relative Bias	64.61 [64.61 – 64.61]	-5.42 [-13.8 – 4.6]	0.26 [-9.24 – 6.89]	4.72 [2.2 – 6.29]	–	–	–	–	–
	Regression Slope	24 [24 – 24]	-0.15 [-0.66 – 0.67]	-0.37 [-1.65 – 1.4]	-2.6 [-4.9 – -0.05]	–	–	–	–	–
	Regression Intercept	-1.2 [-1.2 – -1.2]	6.35 [-47.75 – 47.5]	2.35 [-80.75 – 95]	1.4 [-0.36 – 4.9]	–	–	–	–	–
COx_L	Bias	–	–	–	–	–	–	–	–	–
	Lower LoA	–	–	–	–	–	–	–	–	–
	Upper LoA	–	–	–	–	–	–	–	–	–
	LoA Spread	–	–	–	–	–	–	–	–	–
	Relative Bias	–	–	–	–	–	–	–	–	–
	Regression Slope	–	–	–	–	–	–	–	–	–
	Regression Intercept	–	–	–	–	–	–	–	–	–
COx_R	Bias	–	–	–	–	–	–	–	–	–
	Lower LoA	–	–	–	–	–	–	–	–	–
	Upper LoA	–	–	–	–	–	–	–	–	–
	LoA Spread	–	–	–	–	–	–	–	–	–
	Relative Bias	–	–	–	–	–	–	–	–	–
	Regression Slope	–	–	–	–	–	–	–	–	–
	Regression Intercept	–	–	–	–	–	–	–	–	–
COx-a_L	Bias	0.65 [0.65 – 0.65]	-0.03 [-0.11 – 0.07]	-0.01 [-0.13 – 0.11]	0.04 [-0.27 – 0.28]	–	–	–	–	–
	Lower LoA	0.15 [0.15 – 0.15]	-0.58 [-0.82 – -0.34]	-0.52 [-0.79 – -0.29]	-0.46 [-0.69 – -0.06]	–	–	–	–	–
	Upper LoA	64.61 [64.61 – 64.61]	-2.3 [-7.96 – 4.32]	-0.8 [-10.95 – 9.16]	-7.91 [-66.22 – 44.68]	–	–	–	–	–
	LoA Spread	1 [1 – 1]	1.16 [0.64 – 1.55]	1 [0.52 – 1.36]	0.53 [0.23 – 0.96]	–	–	–	–	–
	Relative Bias	1.15 [1.15 – 1.15]	0.57 [0.26 – 0.78]	0.5 [0.25 – 0.73]	0.29 [-0.02 – 0.58]	–	–	–	–	–
	Regression Slope	-1.2 [-1.2 – -1.2]	-0.02 [-0.17 – 0.16]	-0.06 [-0.31 – 0.19]	0.11 [-0.36 – 0.29]	–	–	–	–	–
	Regression Intercept	24 [24 – 24]	-0.18 [-1 – 0.52]	-0.44 [-1.3 – 0.82]	0.21 [-1.07 – 0.45]	–	–	–	–	–
COx-a_R	Bias	0.65 [0.65 – 0.65]	0 [-0.07 – 0.07]	-0.04 [-0.14 – 0.08]	-0.02 [-0.5 – 0.2]	–	–	–	–	–
	Lower LoA	0.15 [0.15 – 0.15]	-0.54 [-0.73 – -0.33]	-0.57 [-0.84 – -0.32]	-0.78 [-1.19 – -0.06]	–	–	–	–	–
	Upper LoA	64.61 [64.61 – 64.61]	-0.54 [-7.67 – 4.8]	-3.72 [-16.15 – 7.99]	-12.49 [-23.8 – 45.66]	–	–	–	–	–
	LoA Spread	1 [1 – 1]	1.02 [0.66 – 1.41]	1.07 [0.53 – 1.73]	0.93 [0.75 – 1.83]	–	–	–	–	–
	Relative Bias	1.15 [1.15 – 1.15]	0.5 [0.33 – 0.71]	0.52 [0.24 – 0.81]	0.51 [0.2 – 1.06]	–	–	–	–	–
	Regression Slope	-1.2 [-1.2 – -1.2]	0 [-0.13 – 0.23]	-0.03 [-0.27 – 0.15]	-0.11 [-0.44 – 0.18]	–	–	–	–	–
	Regression Intercept	24 [24 – 24]	-0.07 [-1.3 – 0.59]	-0.34 [-1.4 – 0.79]	-0.59 [-3.5 – -0.15]	–	–	–	–	–
**SP Population**										
rSO$_2$_L	Bias	0.08 [0.08 – 0.08]	0 [-0.15 – 0.3]	0.22 [-0.09 – 0.36]	0.1 [-0.1 – 0.62]	0.12 [-0.12 – 0.31]	0.35 [0.11 – 0.63]	-0.24 [-0.66 – 0.18]	–	–
	Lower LoA	-1.04 [-1.04 – -1.04]	-4.09 [-7.23 – -2.54]	-5.32 [-9.3 – -3.09]	-3.89 [-6.47 – -2.81]	-3.87 [-7.75 – -2.36]	-2.91 [-6.17 – -2]	-30.14 [-41.99 – -18.3]	–	–
	Upper LoA	1.19 [1.19 – 1.19]	4.14 [3.2 – 6.21]	5.16 [3.03 – 9.4]	4.72 [3.79 – 8.01]	3.7 [2.68 – 7.87]	3.9 [2.82 – 6.9]	29.67 [16.98 – 42.36]	–	–
	LoA Spread	2.23 [2.23 – 2.23]	8.22 [6.14 – 13.31]	10.35 [5.79 – 18.82]	9.45 [6.63 – 14.76]	7.94 [5.01 – 15.49]	6.75 [4.84 – 13.84]	59.81 [35.28 – 84.34]	–	–
	Relative Bias	3.46 [3.46 – 3.46]	0.09 [-1.44 – 1.7]	1.93 [-0.73 – 4.3]	0.86 [-1.56 – 5.29]	1.23 [-2.45 – 3.5]	5.58 [0.85 – 8.78]	-4.74 [-7.38 – -2.09]	–	–

	Regression Slope	-0.44 [-0.44 – -0.44]	0 [-0.03 – 0.02]	-0.21 [-0.44 – -0.08]	-0.2 [-0.36 – -0.07]	-0.06 [-0.39 – 0.08]	0.05 [-0.12 – 0.21]	0.39 [0.03 – 0.74]	–	–
	Regression Intercept	0.17 [0.17 – 0.17]	0.01 [-1.75 – 1.3]	12 [4.85 – 29.5]	14 [4.65 – 23]	4.7 [-5.23 – 23.75]	-2.24 [-17.25 – 7.38]	-31 [-57.5 – -4.5]	–	–
rSO_2_R	Bias	0.08 [0.08 – 0.08]	-0.06 [-0.35 – 0.19]	0.05 [-0.33 – 0.3]	0.17 [-0.2 – 0.65]	0.14 [-0.16 – 0.29]	0.41 [0.06 – 0.8]	-983.87 [-1475.34 – -492.41]	–	–
	Lower LoA	-1.04 [-1.04 – -1.04]	-3.79 [-6.15 – -2.73]	-6.37 [-9.85 – -3.48]	-5.69 [-7.02 – -3.55]	-4.14 [-6.3 – -2.35]	-2.89 [-5.52 – -1.84]	-7695.31 [-11538.87 – -3851.74]	–	–
	Upper LoA	1.19 [1.19 – 1.19]	3.78 [2.93 – 5.65]	5.72 [3.63 – 9.25]	5.18 [3.88 – 7.85]	4.52 [2.75 – 6.5]	4.39 [2.36 – 6.13]	5727.56 [2866.93 – 8588.2]	–	–
	LoA Spread	2.23 [2.23 – 2.23]	8.16 [5.45 – 11.81]	12.07 [7.14 – 19.67]	10.81 [7.39 – 14.79]	8.74 [5.17 – 12.28]	6.92 [4.63 – 11.7]	13422.87 [6718.68 – 20127.07]	–	–
	Relative Bias	3.46 [3.46 – 3.46]	-1.11 [-4.1 – 2.06]	0.92 [-2.49 – 1.8]	1.61 [-2.46 – 4.13]	1.79 [-1.03 – 3.77]	5.06 [1.44 – 11.49]	-6.91 [-7.12 – -6.7]	–	–
	Regression Slope	-0.44 [-0.44 – -0.44]	0 [-0.04 – 0.06]	-0.23 [-0.5 – -0.09]	-0.25 [-0.5 – -0.12]	-0.19 [-0.4 – 0.04]	0.1 [-0.04 – 0.29]	0.1 [-0.95 – 1.15]	–	–
	Regression Intercept	0.17 [0.17 – 0.17]	-0.33 [-3.95 – 2.15]	14 [5.85 – 28]	16 [8.9 – 35]	12.1 [-2.08 – 24.5]	-6.7 [-18.5 – 4.3]	-10 [-75 – 55]	–	–
COx_L	Bias	–	–	–	–	–	–	–	–	–
	Lower LoA	–	–	–	–	–	–	–	–	–
	Upper LoA	–	–	–	–	–	–	–	–	–
	LoA Spread	–	–	–	–	–	–	–	–	–
	Relative Bias	–	–	–	–	–	–	–	–	–
	Regression Slope	–	–	–	–	–	–	–	–	–
	Regression Intercept	–	–	–	–	–	–	–	–	–
COx_R	Bias	–	–	–	–	–	–	–	–	–
	Lower LoA	–	–	–	–	–	–	–	–	–
	Upper LoA	–	–	–	–	–	–	–	–	–
	LoA Spread	–	–	–	–	–	–	–	–	–
	Relative Bias	–	–	–	–	–	–	–	–	–
	Regression Slope	–	–	–	–	–	–	–	–	–
	Regression Intercept	–	–	–	–	–	–	–	–	–
COx-a_L	Bias	0.08 [0.08 – 0.08]	-0.01 [-0.02 – 0.02]	0.01 [-0.03 – 0.03]	0.02 [-0.02 – 0.08]	0.01 [-0.02 – 0.05]	0.01 [-0.08 – 0.1]	-0.04 [-0.08 – -0.01]	–	–
	Lower LoA	-1.04 [-1.04 – -1.04]	-0.79 [-0.97 – -0.64]	-0.95 [-1.21 – -0.75]	-0.93 [-1.16 – -0.81]	-0.76 [-0.92 – -0.64]	-0.63 [-0.87 – -0.56]	-0.63 [-0.64 – -0.63]	–	–
	Upper LoA	3.46 [3.46 – 3.46]	-0.27 [-1.27 – 1.4]	0.37 [-1.61 – 1.72]	0.86 [-1 – 3.15]	0.84 [-1.44 – 3.34]	0.43 [-5.86 – 3.35]	-4.25 [-7.42 – -1.08]	–	–
	LoA Spread	2.23 [2.23 – 2.23]	1.51 [1.31 – 1.95]	1.98 [1.5 – 2.37]	1.83 [1.63 – 2.48]	1.51 [1.24 – 1.8]	1.42 [1.09 – 1.67]	1.18 [1.12 – 1.24]	–	–
	Relative Bias	1.19 [1.19 – 1.19]	0.79 [0.67 – 0.94]	0.94 [0.81 – 1.18]	0.94 [0.8 – 1.38]	0.75 [0.66 – 0.96]	0.66 [0.57 – 0.87]	0.55 [0.48 – 0.61]	–	–
	Regression Slope	0.17 [0.17 – 0.17]	0 [-0.02 – 0.04]	0.07 [0.03 – 0.15]	0.09 [0.03 – 0.3]	-0.03 [-0.13 – 0.11]	-0.15 [-0.31 – -0.03]	-0.38 [-0.51 – -0.24]	–	–
	Regression Intercept	-0.44 [-0.44 – -0.44]	-0.03 [-0.18 – 0.06]	-0.47 [-0.65 – -0.33]	-0.29 [-0.63 – -0.17]	0.3 [-0.17 – 0.75]	1.35 [0.59 – 1.88]	1.17 [0.86 – 1.49]	–	–
COx-a_R	Bias	0.08 [0.08 – 0.08]	0 [-0.02 – 0.01]	0 [-0.03 – 0.04]	0.01 [-0.02 – 0.09]	-0.01 [-0.04 – 0.02]	-0.03 [-0.06 – 0.04]	-0.01 [-0.06 – 0.03]	–	–
	Lower LoA	-1.04 [-1.04 – -1.04]	-0.79 [-0.98 – -0.7]	-0.97 [-1.14 – -0.79]	-0.97 [-1.23 – -0.78]	-0.83 [-1.06 – -0.68]	-0.71 [-0.88 – -0.62]	-0.42 [-0.5 – -0.33]	–	–
	Upper LoA	3.46 [3.46 – 3.46]	-0.16 [-1.34 – 0.86]	0.18 [-1.48 – 2.16]	1.1 [-1.42 – 5.29]	-0.22 [-2.76 – 1.64]	-1.36 [-3.65 – 3.86]	0.84 [-4.68 – 6.35]	–	–
	LoA Spread	2.23 [2.23 – 2.23]	1.57 [1.41 – 1.94]	1.95 [1.71 – 2.3]	2.11 [1.59 – 2.39]	1.69 [1.41 – 2]	1.41 [1.08 – 1.71]	0.81 [0.72 – 0.89]	–	–
	Relative Bias	1.19 [1.19 – 1.19]	0.8 [0.71 – 0.97]	0.95 [0.86 – 1.15]	1.09 [0.82 – 1.26]	0.84 [0.72 – 1.02]	0.69 [0.56 – 0.86]	0.39 [0.39 – 0.39]	–	–
	Regression Slope	0.17 [0.17 – 0.17]	0 [-0.02 – 0.01]	0.06 [0.01 – 0.1]	0.1 [0.04 – 0.27]	-0.03 [-0.13 – 0.06]	-0.18 [-0.36 – -0.06]	-0.33 [-0.46 – -0.21]	–	–
	Regression Intercept	-0.44 [-0.44 – -0.44]	-0.02 [-0.09 – 0.07]	-0.42 [-0.6 – -0.3]	-0.35 [-0.66 – -0.09]	0.08 [-0.44 – 0.64]	0.87 [0.4 – 1.53]	1.28 [0.87 – 1.69]	–	–
**TBI Population**										
rSO_2_L	Bias	-0.01 [-0.01 – -0.01]	-0.07 [-0.16 – -0.03]	-0.01 [-0.05 – 0.05]	-0.03 [-0.14 – 0.06]	-0.03 [-0.2 – 0.12]	0.01 [-0.09 – 0.12]	0.01 [-0.02 – 0.11]	0 [-0.04 – 0.07]	-0.01 [-0.05 – 0.03]
	Lower LoA	-0.91 [-0.91 – -0.91]	-3.89 [-7.29 – -2.65]	-4.52 [-8.6 – -3.04]	-6.27 [-11.39 – -3.98]	-6.85 [-13.19 – -4.33]	-6.36 [-12.28 – -3.49]	-4.36 [-11.19 – -2.6]	-3.71 [-9.95 – -2.6]	-3.78 [-8.85 – -2.71]
	Upper LoA	0.9 [0.9 – 0.9]	3.79 [2.54 – 7.25]	4.46 [3.04 – 8.62]	5.81 [3.81 – 11.4]	6.65 [4.24 – 13.04]	6.08 [3.34 – 12.82]	4.31 [2.64 – 11.28]	3.72 [2.46 – 10.29]	3.79 [2.7 – 8.78]
	LoA Spread	1.81 [1.81 – 1.81]	7.65 [5.12 – 14.54]	9.04 [6.13 – 17.19]	12.06 [7.82 – 22.81]	13.61 [8.44 – 26.25]	12.44 [6.82 – 25.1]	8.89 [5.21 – 22.41]	7.4 [4.99 – 20.14]	7.53 [5.41 – 17.85]
	Relative Bias	-0.3 [-0.3 – -0.3]	-0.81 [-1.6 – -0.35]	-0.12 [-0.66 – 0.58]	-0.38 [-1.3 – 0.58]	-0.22 [-1.51 – 0.64]	0.14 [-0.75 – 0.88]	0.1 [-0.37 – 0.75]	0.01 [-0.63 – 0.71]	-0.15 [-0.53 – 0.48]
	Regression Slope	-0.67 [-0.67 – -0.67]	0 [-0.01 – 0.01]	-0.04 [-0.07 – -0.01]	-0.08 [-0.15 – -0.04]	-0.11 [-0.2 – -0.05]	-0.05 [-0.22 – -0.02]	-0.01 [-0.07 – 0]	0 [-0.08 – 0.01]	0.02 [-0.01 – 0.04]

	Regression Intercept	0.08 [0.08 – 0.08]	-0.09 [-0.72 – 0.43]	2.4 [0.75 – 4.55]	5.15 [2.23 – 10.75]	7.3 [3.03 – 15]	3.25 [1.4 – 15]	0.48 [-0.32 – 5.3]	0.08 [-1.14 – 4.4]	-0.99 [-2.45 – 0.71]
rSO_2_R	Bias	-0.01 [-0.01 – -0.01]	-0.07 [-0.17 – -0.03]	0.01 [-0.03 – 0.08]	-0.03 [-0.17 – 0.1]	-0.02 [-0.13 – 0.11]	-0.02 [-0.08 – 0.12]	0 [-0.06 – 0.12]	0 [-0.05 – 0.12]	0 [-0.05 – 0.03]
	Lower LoA	-0.91 [-0.91 – -0.91]	-4.19 [-7.3 – -2.36]	-4.86 [-9.04 – -2.5]	-6.64 [-10.64 – -3.88]	-6.91 [-12.51 – -3.72]	-5.66 [-12.48 – -3.27]	-4.55 [-9.75 – -2.42]	-4.59 [-11.25 – -2.67]	-4.88 [-10.08 – -2.25]
	Upper LoA	0.9 [0.9 – 0.9]	3.89 [2.17 – 7.14]	4.83 [2.61 – 8.96]	6.11 [3.63 – 10.57]	6.63 [3.72 – 12.88]	5.7 [3.06 – 12.47]	4.51 [2.44 – 9.5]	4.43 [2.7 – 11.14]	4.83 [2.33 – 9.79]
	LoA Spread	1.81 [1.81 – 1.81]	8.05 [4.49 – 14.4]	9.54 [5.07 – 17.95]	12.91 [7.57 – 21.04]	13.54 [7.4 – 25.39]	11.28 [6.27 – 24.89]	8.91 [4.86 – 19.35]	8.93 [5.37 – 22.77]	9.68 [4.58 – 19.89]
	Relative Bias	-0.3 [-0.3 – -0.3]	-0.84 [-1.65 – -0.45]	0.05 [-0.34 – 0.85]	-0.31 [-1.37 – 0.66]	-0.14 [-1.3 – 0.66]	-0.13 [-0.77 – 0.64]	-0.01 [-0.87 – 0.98]	0.05 [-0.7 – 0.75]	0.03 [-0.76 – 0.37]
	Regression Slope	-0.67 [-0.67 – -0.67]	0 [0 – 0.01]	-0.03 [-0.07 – -0.01]	-0.07 [-0.2 – -0.02]	-0.08 [-0.24 – -0.03]	-0.05 [-0.14 – -0.01]	-0.01 [-0.06 – 0.01]	0 [-0.03 – 0.02]	0.01 [0 – 0.04]
	Regression Intercept	0.08 [0.08 – 0.08]	-0.2 [-1.1 – 0.17]	2.45 [0.84 – 4.95]	4.8 [1.23 – 13.75]	4.85 [1.75 – 16.5]	3.45 [0.76 – 9.4]	0.39 [-0.58 – 4.15]	-0.23 [-1.5 – 1.7]	-0.56 [-2.4 – 0.06]
COx_L	Bias	-0.01 [-0.01 – -0.01]	0 [0 – 0]	0 [-0.01 – 0.01]	0 [-0.01 – 0.01]	0 [-0.01 – 0]	0 [-0.01 – 0]	0 [0 – 0]	0 [0 – 0]	0 [0 – 0.01]
	Lower LoA	-0.91 [-0.91 – -0.91]	-0.61 [-0.65 – -0.57]	-0.73 [-0.8 – -0.69]	-0.81 [-0.94 – -0.73]	-0.71 [-0.83 – -0.64]	-0.61 [-0.68 – -0.56]	-0.53 [-0.59 – -0.5]	-0.51 [-0.57 – -0.48]	-0.51 [-0.55 – -0.48]
	Upper LoA	0.9 [0.9 – 0.9]	0.61 [0.57 – 0.65]	0.75 [0.69 – 0.81]	0.82 [0.73 – 0.95]	0.7 [0.64 – 0.85]	0.61 [0.55 – 0.7]	0.53 [0.5 – 0.59]	0.51 [0.48 – 0.57]	0.52 [0.48 – 0.55]
	LoA Spread	1.81 [1.81 – 1.81]	1.22 [1.14 – 1.3]	1.47 [1.39 – 1.61]	1.64 [1.45 – 1.89]	1.42 [1.28 – 1.69]	1.22 [1.12 – 1.38]	1.06 [1 – 1.19]	1.02 [0.96 – 1.13]	1.04 [0.96 – 1.09]
	Relative Bias	-0.3 [-0.3 – -0.3]	-0.03 [-0.27 – 0.1]	0.01 [-0.42 – 0.47]	-0.11 [-0.5 – 0.54]	-0.02 [-0.42 – 0.23]	-0.01 [-0.37 – 0.26]	0.09 [-0.3 – 0.39]	0.15 [-0.3 – 0.45]	0.23 [-0.07 – 0.54]
	Regression Slope	-0.67 [-0.67 – -0.67]	0 [-0.03 – 0]	-0.35 [-0.42 – -0.29]	-0.51 [-0.66 – -0.28]	-0.12 [-0.41 – 0.17]	0.28 [0.1 – 0.49]	0.75 [0.53 – 1]	0.96 [0.75 – 1.2]	1 [0.78 – 1.2]
	Regression Intercept	0.08 [0.08 – 0.08]	0 [0 – 0]	0.01 [0 – 0.03]	0.01 [-0.01 – 0.04]	0 [-0.01 – 0.02]	0 [-0.02 – 0.01]	-0.01 [-0.05 – 0.01]	-0.02 [-0.06 – 0.03]	-0.03 [-0.1 – 0.01]
COx_R	Bias	-0.01 [-0.01 – -0.01]	0 [0 – 0]	0 [-0.01 – 0]	0 [-0.01 – 0.01]	0 [-0.01 – 0.01]	0 [-0.01 – 0.01]	0 [0 – 0]	0 [0 – 0]	0 [-0.01 – 0.01]
	Lower LoA	-0.91 [-0.91 – -0.91]	-0.6 [-0.66 – -0.56]	-0.74 [-0.8 – -0.69]	-0.81 [-0.95 – -0.72]	-0.68 [-0.79 – -0.63]	-0.6 [-0.68 – -0.55]	-0.52 [-0.57 – -0.49]	-0.51 [-0.55 – -0.48]	-0.5 [-0.55 – -0.47]
	Upper LoA	0.9 [0.9 – 0.9]	0.61 [0.56 – 0.65]	0.73 [0.69 – 0.8]	0.8 [0.71 – 0.95]	0.7 [0.63 – 0.83]	0.6 [0.55 – 0.69]	0.52 [0.5 – 0.58]	0.51 [0.48 – 0.56]	0.5 [0.46 – 0.55]
	LoA Spread	1.81 [1.81 – 1.81]	1.21 [1.11 – 1.31]	1.47 [1.39 – 1.6]	1.61 [1.42 – 1.92]	1.35 [1.28 – 1.67]	1.19 [1.09 – 1.36]	1.04 [1 – 1.15]	1.02 [0.97 – 1.11]	0.99 [0.94 – 1.09]
	Relative Bias	-0.3 [-0.3 – -0.3]	-0.07 [-0.31 – 0.12]	-0.09 [-0.45 – 0.26]	-0.11 [-0.58 – 0.42]	0.02 [-0.42 – 0.38]	0.01 [-0.39 – 0.35]	-0.02 [-0.42 – 0.28]	-0.09 [-0.38 – 0.35]	0.05 [-0.46 – 0.62]
	Regression Slope	-0.67 [-0.67 – -0.67]	-0.01 [-0.03 – 0]	-0.34 [-0.39 – -0.29]	-0.42 [-0.71 – -0.19]	-0.09 [-0.51 – 0.21]	0.23 [0.02 – 0.53]	0.72 [0.54 – 0.92]	0.92 [0.66 – 1.1]	0.98 [0.72 – 1.2]
	Regression Intercept	-0.01 [-0.01 – -0.01]	0 [0 – 0]	0 [0 – 0.01]	0 [-0.01 – 0.01]	0 [0 – 0]	0 [-0.01 – 0]	0 [0 – 0]	0 [0 – 0.01]	0 [0 – 0.01]
COx-a_L	Bias	-0.3 [-0.3 – -0.3]	-0.06 [-0.32 – 0.04]	0.2 [-0.14 – 0.52]	0.11 [-0.35 – 0.61]	-0.06 [-0.32 – 0.29]	0 [-0.4 – 0.33]	-0.04 [-0.3 – 0.37]	0.11 [-0.28 – 0.55]	0.23 [-0.19 – 0.81]
	Lower LoA	1.81 [1.81 – 1.81]	1.2 [1.1 – 1.3]	1.45 [1.34 – 1.58]	1.63 [1.45 – 1.8]	1.4 [1.23 – 1.6]	1.22 [1.08 – 1.31]	1.05 [0.97 – 1.15]	1 [0.93 – 1.1]	0.99 [0.91 – 1.06]
	Upper LoA	-0.91 [-0.91 – -0.91]	-0.6 [-0.66 – -0.56]	-0.72 [-0.78 – -0.67]	-0.82 [-0.9 – -0.71]	-0.7 [-0.8 – -0.61]	-0.61 [-0.65 – -0.54]	-0.53 [-0.57 – -0.48]	-0.5 [-0.55 – -0.46]	-0.49 [-0.52 – -0.46]
	LoA Spread	0.9 [0.9 – 0.9]	0.6 [0.55 – 0.65]	0.73 [0.67 – 0.79]	0.81 [0.73 – 0.9]	0.7 [0.61 – 0.81]	0.61 [0.54 – 0.66]	0.53 [0.49 – 0.58]	0.5 [0.46 – 0.54]	0.49 [0.46 – 0.53]
	Relative Bias	0.08 [0.08 – 0.08]	0 [0 – 0]	0.03 [0.02 – 0.05]	0.03 [0.01 – 0.07]	0 [-0.01 – 0.03]	-0.01 [-0.05 – 0]	-0.05 [-0.1 – -0.02]	-0.07 [-0.12 – -0.03]	-0.1 [-0.15 – -0.05]
	Regression Slope	-0.67 [-0.67 – -0.67]	-0.01 [-0.02 – 0]	-0.36 [-0.39 – -0.31]	-0.51 [-0.69 – -0.27]	-0.06 [-0.54 – 0.15]	0.33 [0.06 – 0.56]	0.75 [0.59 – 1]	0.97 [0.77 – 1.2]	1 [0.86 – 1.3]
	Regression Intercept	-0.01 [-0.01 – -0.01]	0 [0 – 0]	0 [0 – 0.01]	0 [-0.01 – 0.01]	0 [0 – 0.01]	0 [0 – 0]	0 [0 – 0]	0 [0 – 0.01]	0 [0 – 0.01]
COx-a_R	Bias	-0.3 [-0.3 – -0.3]	-0.08 [-0.3 – 0.07]	0 [-0.27 – 0.4]	0.04 [-0.42 – 0.57]	-0.04 [-0.34 – 0.41]	-0.05 [-0.34 – 0.34]	0.06 [-0.32 – 0.43]	0.06 [-0.4 – 0.5]	0.05 [-0.28 – 0.56]
	Lower LoA	1.81 [1.81 – 1.81]	1.19 [1.09 – 1.32]	1.43 [1.35 – 1.58]	1.6 [1.48 – 1.79]	1.44 [1.24 – 1.67]	1.23 [1.08 – 1.36]	1.06 [0.96 – 1.13]	1.02 [0.9 – 1.1]	0.99 [0.89 – 1.08]
	Upper LoA	-0.91 [-0.91 – -0.91]	-0.59 [-0.66 – -0.55]	-0.72 [-0.78 – -0.67]	-0.79 [-0.9 – -0.74]	-0.72 [-0.83 – -0.62]	-0.61 [-0.68 – -0.54]	-0.53 [-0.57 – -0.47]	-0.5 [-0.55 – -0.45]	-0.49 [-0.54 – -0.44]
	LoA Spread	0.9 [0.9 – 0.9]	0.59 [0.54 – 0.65]	0.71 [0.67 – 0.8]	0.81 [0.74 – 0.91]	0.71 [0.62 – 0.83]	0.61 [0.54 – 0.68]	0.53 [0.49 – 0.57]	0.51 [0.45 – 0.55]	0.49 [0.44 – 0.55]
	Relative Bias	0.08 [0.08 – 0.08]	0 [0 – 0]	0.02 [0.01 – 0.05]	0.03 [0.01 – 0.07]	0.01 [-0.01 – 0.02]	-0.01 [-0.04 – 0]	-0.05 [-0.1 – -0.01]	-0.08 [-0.12 – -0.01]	-0.09 [-0.14 – -0.02]
	Regression Slope	-0.67 [-0.67 – -0.67]	-0.01 [-0.02 – 0]	-0.35 [-0.39 – -0.3]	-0.58 [-0.74 – -0.34]	-0.16 [-0.56 – 0.02]	0.22 [0 – 0.44]	0.69 [0.57 – 0.97]	0.94 [0.79 – 1.1]	1.1 [0.92 – 1.18]
	Regression Intercept	0.08 [0.08 – 0.08]	0 [0 – 0]	0.01 [-0.01 – 0.04]	0.01 [-0.03 – 0.03]	-0.01 [-0.02 – 0.01]	0 [-0.02 – 0.02]	-0.02 [-0.06 – 0.03]	-0.03 [-0.1 – 0.03]	-0.02 [-0.08 – 0.07]

COx, cerebral oximetry index with cerebral perfusion pressure; COx-a, cerebral oximetry index with arterial blood pressure; HC, healthy control volunteer group; IQR, interquartile range; r-value, Pearson correlation coefficient; rSO_2, regional cerebral oxygen saturation; SP, elective spinal surgery patient group; TBI, traumatic brain injury patient group.

File S8j: Windowed-Interval – Bland-Altman Analysis of rSO_2 and COx/COx-a in All Populations using 1-Minute Temporal Resolution

Physiologic Variable	Value	Median [IQR]								
		5-Minute Interval	10-Minute Interval	15-Minute Interval	30-Minute Interval	1-Hour Interval	2-Hour Interval	6-Hour Interval	12-Hour Interval	1-Day Interval
**HC Population**										
rSO₂_L	Bias	-0.01 [-0.4 – 0.38]	0.1 [-0.47 – 0.71]	-0.14 [-0.73 – 0.95]	–	–	–	–	–	–
	Lower LoA	-2.95 [-4.13 – -2.04]	-2.39 [-3.74 – -1.55]	-2.02 [-3.29 – -0.81]	–	–	–	–	–	–
	Upper LoA	3.02 [2.02 – 4.59]	2.72 [1.84 – 4.14]	2.32 [1.28 – 3.54]	–	–	–	–	–	–
	LoA Spread	6.07 [4.15 – 8]	5.35 [3.66 – 7.12]	4.28 [3.11 – 5.55]	–	–	–	–	–	–
	Relative Bias	-0.1 [-7.33 – 6.93]	1.95 [-8.14 – 13.98]	-3.62 [-17.17 – 28.56]	–	–	–	–	–	–
	Regression Slope	-0.35 [-0.92 – 0.05]	0.31 [-0.59 – 1.03]	0.94 [0.32 – 1.7]	–	–	–	–	–	–
	Regression Intercept	19 [-3.85 – 69]	-23 [-78 – 42.5]	-74 [-120 – -24.5]	–	–	–	–	–	–
rSO₂_R	Bias	-0.06 [-0.4 – 0.39]	0.07 [-0.42 – 0.86]	0.09 [-0.84 – 1.22]	–	–	–	–	–	–
	Lower LoA	-3.38 [-4.66 – -2.25]	-2.62 [-3.84 – -1.79]	-2.25 [-2.99 – -1.35]	–	–	–	–	–	–
	Upper LoA	3.27 [2.2 – 5]	3.2 [2.02 – 4.35]	2.51 [1.34 – 4.06]	–	–	–	–	–	–
	LoA Spread	6.66 [4.5 – 9.32]	5.75 [4.46 – 7.49]	4.64 [3.72 – 6.67]	–	–	–	–	–	–
	Relative Bias	-0.79 [-6.83 – 5.49]	1.05 [-6.43 – 10.01]	1.87 [-20.82 – 24.63]	–	–	–	–	–	–
	Regression Slope	-0.35 [-0.92 – 0.09]	0.27 [-0.47 – 1.13]	0.86 [0.4 – 1.8]	–	–	–	–	–	–
	Regression Intercept	22.5 [-6.2 – 63.75]	-19 [-80.5 – 35.25]	-64.5 [-117.5 – -29.5]	–	–	–	–	–	–
COx_L	Bias	–	–	–	–	–	–	–	–	–
	Lower LoA	–	–	–	–	–	–	–	–	–
	Upper LoA	–	–	–	–	–	–	–	–	–
	LoA Spread	–	–	–	–	–	–	–	–	–
	Relative Bias	–	–	–	–	–	–	–	–	–
	Regression Slope	–	–	–	–	–	–	–	–	–
	Regression Intercept	–	–	–	–	–	–	–	–	–
COx_R	Bias	–	–	–	–	–	–	–	–	–
	Lower LoA	–	–	–	–	–	–	–	–	–
	Upper LoA	–	–	–	–	–	–	–	–	–
	LoA Spread	–	–	–	–	–	–	–	–	–
	Relative Bias	–	–	–	–	–	–	–	–	–
	Regression Slope	–	–	–	–	–	–	–	–	–
	Regression Intercept	–	–	–	–	–	–	–	–	–
COx-a_L	Bias	-0.01 [-0.17 – 0.1]	0 [-0.13 – 0.09]	-0.05 [-0.21 – 0.11]	–	–	–	–	–	–
	Lower LoA	-0.93 [-1.52 – -0.6]	-0.64 [-0.86 – -0.41]	-0.52 [-0.74 – -0.34]	–	–	–	–	–	–
	Upper LoA	-1.24 [-6.59 – 4.27]	-0.16 [-10.17 – 7.72]	-6.42 [-22.03 – 11.11]	–	–	–	–	–	–
	LoA Spread	1.93 [1.29 – 2.73]	1.3 [0.89 – 1.69]	0.97 [0.71 – 1.19]	–	–	–	–	–	–
	Relative Bias	0.88 [0.6 – 1.53]	0.62 [0.41 – 0.82]	0.42 [0.21 – 0.61]	–	–	–	–	–	–
	Regression Slope	0.08 [-0.07 – 0.32]	-0.04 [-0.22 – 0.1]	-0.16 [-0.43 – 0.17]	–	–	–	–	–	–
	Regression Intercept	-0.74 [-1.2 – -0.19]	0.32 [-0.43 – 1.1]	1.35 [0.32 – 1.9]	–	–	–	–	–	–
COx-a_R	Bias	0 [-0.08 – 0.08]	0.01 [-0.13 – 0.14]	-0.07 [-0.23 – 0.07]	–	–	–	–	–	–
	Lower LoA	-0.74 [-1.19 – -0.57]	-0.61 [-0.84 – -0.38]	-0.53 [-0.71 – -0.36]	–	–	–	–	–	–
	Upper LoA	-0.11 [-6.58 – 5.38]	0.47 [-14.28 – 10.92]	-8.26 [-21.72 – 7.79]	–	–	–	–	–	–
	LoA Spread	1.54 [1.16 – 2.31]	1.24 [0.87 – 1.62]	0.93 [0.72 – 1.18]	–	–	–	–	–	–
	Relative Bias	0.77 [0.62 – 1.1]	0.66 [0.38 – 0.91]	0.39 [0.25 – 0.61]	–	–	–	–	–	–
	Regression Slope	0.06 [-0.06 – 0.19]	-0.03 [-0.27 – 0.15]	-0.15 [-0.43 – 0.08]	–	–	–	–	–	–
	Regression Intercept	-0.66 [-1.1 – -0.25]	0.29 [-0.51 – 1.2]	1.6 [0.73 – 2]	–	–	–	–	–	–
**SP Population**										
rSO₂_L	Bias	-0.15 [-0.55 – 0.18]	0.21 [-0.16 – 0.49]	0.05 [-0.96 – 0.86]	0.28 [-0.78 – 1.54]	0 [-1.75 – 1.45]	-2.43 [-2.77 – -0.71]	–	–	–
	Lower LoA	-7.24 [-17.84 – -4.37]	-7.32 [-14.97 – -3.69]	-7.79 [-15.26 – -3.88]	-8.31 [-14.2 – -4.55]	-8.14 [-12.64 – -3.9]	-17.47 [-22.36 – -10.26]	–	–	–
	Upper LoA	7.32 [4.28 – 17]	7.75 [4.41 – 14.69]	8.07 [5.28 – 14.72]	8.65 [6.83 – 12.97]	7.4 [5.82 – 12.27]	11.26 [8.17 – 16.82]	–	–	–
	LoA Spread	14.55 [8.35 – 35.75]	14.67 [7.78 – 29.19]	15.1 [9.72 – 30.8]	17.99 [11.62 – 25.09]	16.73 [9.93 – 22.02]	28.73 [18.43 – 39.18]	–	–	–
	Relative Bias	-1.1 [-3.18 – 1.57]	0.79 [-1.87 – 4.2]	0.51 [-3.67 – 6.14]	0.91 [-3.56 – 10.81]	0.29 [-12.63 – 10.03]	-4.9 [-7.85 – 3.79]	–	–	–
	Regression Slope	-0.21 [-0.82 – -0.03]	-0.17 [-0.52 – -0.01]	-0.16 [-0.72 – 0.04]	-0.07 [-0.66 – 0.34]	0.65 [-0.02 – 1.4]	1.2 [1.1 – 1.5]	–	–	–

	Regression Intercept	13 [2.15 – 53.5]	11 [0.93 – 32]	11 [-2.4 – 46]	6.1 [-24.5 – 44.5]	-39.5 [-88.25 – -0.47]	-98 [-119 – -80.5]	–	–	–
rSO_2_R	Bias	-0.11 [-0.44 – 0.1]	-0.03 [-0.68 – 0.76]	0.02 [-0.9 – 0.83]	-0.08 [-1.2 – 0.87]	-0.2 [-1.31 – 0.84]	-1.67 [-3.52 – -0.32]	–	–	–
	Lower LoA	-6.68 [-12.89 – -4.82]	-9.49 [-12.83 – -6.02]	-7.8 [-12.35 – -4.5]	-8.87 [-13 – -4.37]	-6.34 [-11.88 – -3.88]	-19.73 [-23.81 – -11.02]	–	–	–
	Upper LoA	6.09 [4.73 – 11.24]	8.89 [6.19 – 12.85]	6.44 [4.74 – 13.94]	7.81 [6.28 – 10.78]	6.63 [4.42 – 9.68]	9 [6.67 – 16.78]	–	–	–
	LoA Spread	12.77 [9.74 – 23.71]	18.27 [11.62 – 25.14]	13.18 [9.79 – 26.29]	15.18 [10.81 – 24.12]	14.27 [8.93 – 20.34]	28.73 [17.69 – 40.58]	–	–	–
	Relative Bias	-0.89 [-4.02 – 0.5]	-0.21 [-3.63 – 2.94]	0.3 [-5.1 – 3.71]	-0.83 [-6.02 – 6.55]	-2.31 [-7.66 – 5.34]	-3.18 [-10.93 – 6.08]	–	–	–
	Regression Slope	-0.27 [-0.51 – -0.12]	-0.22 [-0.81 – -0.07]	-0.31 [-0.53 – 0.01]	-0.03 [-0.44 – 0.8]	1.15 [-0.14 – 1.9]	2 [1.18 – 2]	–	–	–
	Regression Intercept	18 [6.8 – 32]	17 [4.35 – 49.5]	22 [-0.46 – 39.5]	-0.38 [-44 – 26]	-78.5 [-117.5 – 7.75]	-150 [-150 – -91.5]	–	–	–
COx_L	Bias	–	–	–	–	–	–	–	–	–
	Lower LoA	–	–	–	–	–	–	–	–	–
	Upper LoA	–	–	–	–	–	–	–	–	–
	LoA Spread	–	–	–	–	–	–	–	–	–
	Relative Bias	–	–	–	–	–	–	–	–	–
	Regression Slope	–	–	–	–	–	–	–	–	–
	Regression Intercept	–	–	–	–	–	–	–	–	–
COx_R	Bias	–	–	–	–	–	–	–	–	–
	Lower LoA	–	–	–	–	–	–	–	–	–
	Upper LoA	–	–	–	–	–	–	–	–	–
	LoA Spread	–	–	–	–	–	–	–	–	–
	Relative Bias	–	–	–	–	–	–	–	–	–
	Regression Slope	–	–	–	–	–	–	–	–	–
	Regression Intercept	–	–	–	–	–	–	–	–	–
COx-a_L	Bias	0.05 [-0.01 – 0.15]	0 [-0.05 – 0.09]	0.02 [-0.1 – 0.11]	-0.01 [-0.07 – 0.04]	0.01 [-0.03 – 0.13]	0.02 [0.01 – 0.07]	–	–	–
	Lower LoA	-1.26 [-1.87 – -1.03]	-1.35 [-1.78 – -1]	-1.01 [-1.43 – -0.88]	-0.97 [-1.05 – -0.73]	-0.77 [-0.9 – -0.59]	-0.59 [-0.69 – -0.56]	–	–	–
	Upper LoA	2.16 [-0.83 – 3.86]	-0.18 [-2.5 – 2.52]	0.71 [-4.43 – 5]	-0.42 [-3.9 – 3.64]	0.76 [-2.27 – 8.31]	1.67 [1.11 – 5.19]	–	–	–
	LoA Spread	2.73 [2.13 – 4.17]	2.66 [2.05 – 3.75]	2.24 [1.82 – 2.65]	1.85 [1.48 – 2.11]	1.56 [1.27 – 1.76]	1.43 [1.27 – 1.52]	–	–	–
	Relative Bias	1.49 [1.1 – 2.24]	1.3 [1 – 1.91]	1.18 [0.97 – 1.35]	0.92 [0.69 – 1.05]	0.8 [0.65 – 0.93]	0.81 [0.69 – 0.82]	–	–	–
	Regression Slope	0.18 [0.07 – 0.32]	0.04 [-0.02 – 0.15]	0.05 [-0.08 – 0.16]	-0.22 [-0.32 – 0]	-0.2 [-0.48 – -0.02]	-0.28 [-0.41 – -0.24]	–	–	–
	Regression Intercept	-0.78 [-1.3 – -0.54]	-0.42 [-1.3 – -0.03]	-0.15 [-0.37 – 0.36]	0.91 [0.51 – 1.4]	1.7 [1.43 – 1.9]	1.9 [1.8 – 1.95]	–	–	–
COx-a_R	Bias	0.02 [-0.04 – 0.11]	0.03 [-0.01 – 0.08]	0.04 [-0.05 – 0.1]	-0.02 [-0.13 – 0.04]	0.07 [-0.03 – 0.13]	0.12 [0.1 – 0.16]	–	–	–
	Lower LoA	-1.37 [-1.8 – -1.15]	-1.17 [-1.56 – -0.99]	-1.04 [-1.35 – -0.85]	-0.94 [-1.09 – -0.78]	-0.74 [-0.83 – -0.6]	-0.56 [-0.65 – -0.56]	–	–	–
	Upper LoA	0.55 [-1.44 – 3.24]	1.02 [-0.5 – 3.17]	1 [-3.28 – 4.47]	-0.85 [-6.97 – 2.04]	4.4 [-2.44 – 6.96]	7.09 [6.8 – 10.25]	–	–	–
	LoA Spread	2.99 [2.37 – 3.61]	2.35 [2.1 – 3.06]	2.22 [1.8 – 2.76]	1.79 [1.57 – 2.08]	1.53 [1.32 – 1.84]	1.52 [1.4 – 1.62]	–	–	–
	Relative Bias	1.53 [1.16 – 1.84]	1.23 [1.1 – 1.55]	1.22 [0.96 – 1.43]	0.86 [0.74 – 1.02]	0.84 [0.65 – 0.98]	0.96 [0.84 – 0.97]	–	–	–
	Regression Slope	0.18 [0.05 – 0.32]	0.09 [-0.01 – 0.22]	0.05 [-0.02 – 0.15]	-0.21 [-0.32 – 0.03]	-0.15 [-0.44 – 0.03]	-0.1 [-0.17 – 0.02]	–	–	–
	Regression Intercept	-0.93 [-1.1 – -0.55]	-0.39 [-0.92 – -0.11]	-0.17 [-0.47 – 0.29]	0.65 [0.41 – 1.2]	1.6 [1.2 – 1.9]	1.4 [1.35 – 1.6]	–	–	–
**TBI Population**										
rSO_2_L	Bias	-0.03 [-0.12 – 0.06]	-0.08 [-0.33 – 0.11]	-0.03 [-0.35 – 0.12]	-0.05 [-0.44 – 0.21]	0 [-0.45 – 0.28]	-0.18 [-0.86 – 0.35]	-0.55 [-1.21 – 0.44]	-0.81 [-1.7 – 0.33]	-1.48 [-3.15 – 1.01]
	Lower LoA	-5.39 [-10.38 – -3.86]	-13.04 [-32.84 – -6.1]	-13.81 [-31.77 – -7.29]	-15.14 [-52.03 – -6.88]	-9.73 [-38.25 – -6.24]	-9.77 [-24.65 – -6.5]	-9 [-15.58 – -6.89]	-10.76 [-17.02 – -7.59]	-10.8 [-20.1 – -8.39]
	Upper LoA	5.46 [3.99 – 10.01]	12.51 [5.99 – 32.3]	13.61 [7.5 – 31.1]	13.54 [6.65 – 51.67]	9.99 [6.07 – 39]	10.19 [6.34 – 26.19]	9 [6.32 – 16.27]	9.43 [6.24 – 17.55]	10.03 [6.07 – 16.21]
	LoA Spread	10.97 [7.81 – 20.39]	25.44 [12.12 – 64.8]	27.16 [14.86 – 62.87]	28.72 [13.5 – 103.7]	19.5 [11.99 – 77.25]	19.54 [12.82 – 51.07]	19.22 [13.87 – 29.8]	21.09 [15.41 – 30.84]	19.73 [15.36 – 38.07]
	Relative Bias	-0.2 [-0.95 – 0.54]	-0.39 [-1.13 – 0.44]	-0.13 [-1.07 – 0.61]	-0.2 [-0.97 – 0.77]	-0.01 [-1.3 – 1.14]	-0.58 [-2.25 – 1.37]	-2.92 [-6.05 – 1.3]	-3.83 [-8.34 – 1.55]	-6.99 [-12.2 – 5.62]
	Regression Slope	-0.08 [-0.18 – -0.02]	-0.46 [-0.99 – -0.1]	-0.3 [-0.75 – -0.09]	-0.24 [-1.6 – -0.06]	-0.12 [-0.97 – 0.01]	-0.03 [-0.34 – 0.1]	0.08 [-0.07 – 0.39]	0.11 [-0.07 – 1]	0.97 [0.16 – 1.85]
	Regression Intercept	5.15 [1.55 – 10.75]	30 [6.75 – 69.5]	21 [6.43 – 52]	17.5 [4.23 – 98.5]	7.4 [-0.75 – 61]	1.9 [-6.48 – 23]	-5.6 [-27 – 5.1]	-8.9 [-58.5 – 4.5]	-47 [-120 – -13.5]
rSO_2_R	Bias	-0.02 [-0.12 – 0.09]	-0.07 [-0.3 – 0.07]	-0.12 [-0.43 – 0.1]	-0.07 [-0.79 – 0.43]	-0.1 [-0.71 – 0.19]	-0.1 [-0.58 – 0.33]	-0.61 [-1.43 – 0.44]	-0.66 [-2.09 – 1.07]	-2.01 [-4.3 – 0.19]

	Lower LoA	-5.57 [-12.51 – -3.1]	-10.31 [-26.52 – -6.3]	-14.15 [-83.69 – -6.76]	-34.04 [-237.7 – -9.15]	-11.64 [-37.83 – -6.09]	-9.86 [-27.86 – -6.61]	-10.15 [-18.69 – -6.87]	-10.86 [-17.95 – -7.62]	-12.21 [-19.09 – -8.5]
	Upper LoA	5.77 [3.11 – 12.17]	9.64 [6.07 – 27.38]	14.6 [6.99 – 83.92]	32.11 [9.5 – 230.33]	12.19 [6.08 – 37.78]	10.77 [6.51 – 27.01]	9.55 [6.91 – 17.67]	10.9 [7.16 – 17.71]	8.95 [6.29 – 16.65]
	LoA Spread	11.47 [6.05 – 24.68]	19.74 [12.25 – 55.02]	28.84 [13.32 – 167.61]	66.15 [18.73 – 468.03]	23.95 [12.12 – 75.61]	20.84 [13.18 – 55.54]	19.48 [14.49 – 37.6]	21.22 [15.57 – 38.72]	21.75 [16.72 – 35.17]
	Relative Bias	-0.25 [-0.91 – 1.01]	-0.25 [-1.09 – 0.45]	-0.58 [-1.05 – 0.27]	-0.2 [-0.79 – 0.62]	-0.44 [-1.77 – 0.73]	-0.3 [-2.17 – 1.8]	-2.49 [-6.18 – 1.84]	-3.12 [-7.04 – 3.06]	-9.14 [-17.72 – 1.16]
	Regression Slope	-0.08 [-0.19 – -0.03]	-0.24 [-0.82 – -0.08]	-0.32 [-1.8 – -0.08]	-1.35 [-2 – -0.07]	-0.06 [-1.28 – 0.05]	-0.07 [-0.6 – 0.08]	0.09 [-0.06 – 0.3]	0.27 [0 – 0.73]	0.9 [0.19 – 1.6]
	Regression Intercept	5.55 [2.15 – 12.75]	16 [4.3 – 64]	21 [5.85 – 110]	103.5 [5.05 – 140]	2.85 [-3.88 – 79.75]	4.4 [-6.38 – 47]	-7.1 [-18.75 – 2.98]	-19 [-58.5 – -0.67]	-63 [-120 – -12]
COx_L	Bias	0 [-0.01 – 0.02]	0.01 [-0.03 – 0.06]	0 [-0.03 – 0.02]	0 [-0.02 – 0.02]	0 [-0.02 – 0.01]	0 [-0.02 – 0.02]	0 [-0.02 – 0.02]	0.01 [-0.01 – 0.04]	0.01 [-0.02 – 0.05]
	Lower LoA	-1.19 [-1.4 – -1]	-1.82 [-5.27 – -1.09]	-1.33 [-4.36 – -0.94]	-0.91 [-1.16 – -0.79]	-0.74 [-0.86 – -0.66]	-0.69 [-0.77 – -0.63]	-0.67 [-0.74 – -0.61]	-0.65 [-0.72 – -0.6]	-0.64 [-0.7 – -0.57]
	Upper LoA	1.21 [1.01 – 1.45]	1.75 [1.04 – 5.31]	1.36 [0.91 – 4.4]	0.91 [0.78 – 1.19]	0.75 [0.68 – 0.88]	0.68 [0.62 – 0.77]	0.66 [0.6 – 0.74]	0.68 [0.61 – 0.76]	0.69 [0.61 – 0.76]
	LoA Spread	2.41 [2.02 – 2.86]	3.58 [2.08 – 10.65]	2.69 [1.85 – 8.75]	1.85 [1.57 – 2.4]	1.51 [1.36 – 1.71]	1.37 [1.28 – 1.52]	1.33 [1.24 – 1.49]	1.34 [1.23 – 1.46]	1.33 [1.23 – 1.45]
	Relative Bias	0.16 [-0.54 – 0.84]	0.33 [-0.54 – 0.93]	-0.06 [-0.77 – 0.6]	-0.1 [-1.15 – 1.31]	-0.27 [-0.93 – 0.91]	0.15 [-1.5 – 1.45]	-0.1 [-1.44 – 1.2]	0.94 [-0.71 – 3.05]	0.91 [-1.13 – 3.21]
	Regression Slope	-0.9 [-1.1 – -0.77]	-1.4 [-1.9 – -0.65]	-0.94 [-1.88 – -0.37]	-0.02 [-0.61 – 0.25]	0.57 [0.37 – 0.79]	0.97 [0.78 – 1.23]	1.5 [1.2 – 1.8]	1.7 [1.48 – 1.9]	1.9 [1.7 – 2]
	Regression Intercept	0.03 [-0.02 – 0.06]	0.03 [-0.02 – 0.1]	0.02 [-0.01 – 0.08]	-0.01 [-0.04 – 0.03]	-0.01 [-0.05 – 0.03]	-0.03 [-0.09 – 0.02]	-0.04 [-0.15 – 0.05]	-0.02 [-0.13 – 0.06]	-0.05 [-0.17 – 0.06]
COx_R	Bias	0 [-0.02 – 0.02]	0.02 [-0.01 – 0.08]	0.01 [-0.04 – 0.05]	0 [-0.01 – 0.04]	0 [-0.02 – 0.02]	0.01 [-0.02 – 0.02]	0 [-0.02 – 0.03]	-0.01 [-0.03 – 0.02]	0.01 [-0.02 – 0.06]
	Lower LoA	-1.13 [-1.36 – -0.96]	-1.88 [-5.11 – -1.01]	-1.33 [-4.66 – -1]	-0.87 [-1.31 – -0.78]	-0.76 [-0.81 – -0.68]	-0.69 [-0.75 – -0.64]	-0.66 [-0.72 – -0.61]	-0.66 [-0.72 – -0.61]	-0.65 [-0.71 – -0.59]
	Upper LoA	1.11 [0.95 – 1.35]	1.96 [1.07 – 4.99]	1.3 [1.02 – 5]	0.89 [0.78 – 1.29]	0.74 [0.7 – 0.82]	0.7 [0.64 – 0.76]	0.67 [0.6 – 0.73]	0.65 [0.6 – 0.74]	0.69 [0.61 – 0.76]
	LoA Spread	2.29 [1.91 – 2.68]	3.84 [2.09 – 10.24]	2.63 [1.98 – 9.58]	1.76 [1.6 – 2.61]	1.48 [1.38 – 1.62]	1.4 [1.3 – 1.5]	1.32 [1.24 – 1.44]	1.32 [1.21 – 1.46]	1.35 [1.24 – 1.42]
	Relative Bias	-0.07 [-0.74 – 0.87]	0.44 [-0.43 – 0.92]	0.23 [-0.79 – 0.99]	0.26 [-0.77 – 0.99]	0.12 [-1.04 – 1.34]	0.39 [-1.48 – 1.44]	-0.27 [-1.8 – 2.5]	-0.91 [-2.36 – 1.23]	0.53 [-1.69 – 3.7]
	Regression Slope	-0.81 [-1 – -0.5]	-1.5 [-1.9 – -0.59]	-0.98 [-1.9 – -0.54]	-0.03 [-1 – 0.27]	0.58 [0.39 – 0.8]	0.99 [0.75 – 1.2]	1.5 [1.25 – 1.7]	1.7 [1.4 – 1.8]	1.8 [1.6 – 2]
	Regression Intercept	0.01 [-0.01 – 0.02]	0.02 [-0.01 – 0.05]	0 [-0.04 – 0.03]	0 [-0.02 – 0.03]	0 [-0.01 – 0.02]	0 [-0.01 – 0.01]	0 [-0.02 – 0.02]	0.01 [-0.01 – 0.03]	0.02 [-0.02 – 0.05]
COx-a_L	Bias	0.3 [-0.31 – 0.82]	0.45 [-0.22 – 0.97]	0.15 [-0.61 – 0.82]	0.08 [-0.83 – 1.27]	0.09 [-0.65 – 1.45]	0.1 [-1 – 1.06]	0.25 [-1.62 – 1.49]	0.66 [-0.92 – 2.33]	1.42 [-1.29 – 4.23]
	Lower LoA	2.28 [1.93 – 2.74]	3.66 [2.18 – 9.64]	2.58 [2.02 – 10.71]	1.84 [1.57 – 2.4]	1.46 [1.32 – 1.66]	1.36 [1.25 – 1.51]	1.26 [1.18 – 1.37]	1.26 [1.18 – 1.38]	1.26 [1.17 – 1.37]
	Upper LoA	-1.15 [-1.36 – -0.95]	-1.77 [-4.79 – -1.08]	-1.28 [-5.31 – -0.99]	-0.92 [-1.2 – -0.77]	-0.73 [-0.81 – -0.65]	-0.68 [-0.75 – -0.62]	-0.64 [-0.69 – -0.59]	-0.63 [-0.69 – -0.58]	-0.61 [-0.66 – -0.55]
	LoA Spread	1.14 [0.97 – 1.37]	1.87 [1.11 – 4.85]	1.29 [0.99 – 5.4]	0.91 [0.8 – 1.2]	0.74 [0.66 – 0.82]	0.69 [0.62 – 0.76]	0.62 [0.59 – 0.7]	0.64 [0.61 – 0.71]	0.66 [0.6 – 0.72]
	Relative Bias	0.07 [0.03 – 0.12]	0.09 [0.04 – 0.18]	0.08 [0.01 – 0.16]	0.01 [-0.01 – 0.07]	-0.03 [-0.07 – 0]	-0.07 [-0.14 – -0.02]	-0.13 [-0.23 – -0.04]	-0.13 [-0.23 – -0.05]	-0.15 [-0.24 – -0.04]
	Regression Slope	-0.91 [-1.1 – -0.67]	-1.5 [-1.9 – -0.78]	-0.98 [-1.98 – -0.47]	-0.05 [-0.9 – 0.2]	0.61 [0.34 – 0.88]	1.1 [0.86 – 1.3]	1.6 [1.4 – 1.8]	1.8 [1.53 – 1.9]	1.9 [1.8 – 2]
	Regression Intercept	0 [-0.01 – 0.02]	0 [-0.02 – 0.04]	0 [-0.02 – 0.02]	0 [-0.02 – 0.02]	0 [-0.02 – 0.02]	0 [-0.02 – 0.02]	0.01 [-0.02 – 0.03]	0 [-0.02 – 0.02]	0.01 [-0.01 – 0.06]
COx-a_R	Bias	0.12 [-0.57 – 0.8]	0.22 [-0.5 – 0.87]	0.02 [-0.62 – 0.65]	0.11 [-0.91 – 0.98]	0.04 [-1.37 – 0.86]	-0.04 [-1.13 – 1.27]	0.54 [-1.37 – 2.34]	0.25 [-1.47 – 1.68]	1.23 [-1.3 – 4.09]
	Lower LoA	2.24 [1.87 – 2.62]	3.41 [2.18 – 6.37]	2.53 [1.96 – 7.52]	1.72 [1.51 – 2.46]	1.46 [1.34 – 1.61]	1.37 [1.22 – 1.48]	1.25 [1.16 – 1.38]	1.28 [1.15 – 1.42]	1.25 [1.15 – 1.39]
	Upper LoA	-1.12 [-1.31 – -0.94]	-1.69 [-3.2 – -1.11]	-1.28 [-3.74 – -0.98]	-0.85 [-1.21 – -0.75]	-0.74 [-0.82 – -0.67]	-0.67 [-0.74 – -0.61]	-0.63 [-0.69 – -0.56]	-0.65 [-0.71 – -0.56]	-0.59 [-0.68 – -0.55]
	LoA Spread	1.11 [0.94 – 1.32]	1.73 [1.08 – 3.24]	1.28 [0.98 – 3.74]	0.87 [0.76 – 1.22]	0.74 [0.66 – 0.8]	0.68 [0.61 – 0.75]	0.64 [0.59 – 0.7]	0.62 [0.59 – 0.71]	0.65 [0.58 – 0.71]
	Relative Bias	0.05 [0.01 – 0.12]	0.08 [0.01 – 0.19]	0.06 [0 – 0.13]	0.01 [-0.03 – 0.04]	-0.04 [-0.11 – 0]	-0.08 [-0.14 – -0.01]	-0.09 [-0.2 – -0.01]	-0.11 [-0.21 – -0.03]	-0.13 [-0.23 – -0.03]
	Regression Slope	-0.92 [-1 – -0.59]	-1.4 [-1.8 – -0.81]	-1 [-1.88 – -0.44]	0 [-1.13 – 0.26]	0.62 [0.35 – 0.83]	1.1 [0.72 – 1.38]	1.6 [1.3 – 1.8]	1.7 [1.5 – 1.9]	1.9 [1.8 – 2]
	Regression Intercept	0.02 [-0.04 – 0.08]	0.02 [-0.05 – 0.14]	0.02 [-0.04 – 0.12]	0 [-0.03 – 0.04]	-0.02 [-0.07 – 0.05]	-0.03 [-0.09 – 0.06]	-0.04 [-0.13 – 0.05]	-0.05 [-0.13 – 0.08]	-0.04 [-0.16 – 0.08]

COx, cerebral oximetry index with cerebral perfusion pressure; COx-a, cerebral oximetry index with arterial blood pressure; HC, healthy control volunteer group; IQR, interquartile range; r-value, Pearson correlation coefficient; rSO_2, regional cerebral oxygen saturation; SP, elective spinal surgery patient group; TBI, traumatic brain injury patient group.

File S8k: Windowed-Interval – Bland-Altman Analysis of rSO_2 and COx/COx-a in All Populations using 5-Minute Temporal Resolution

Physiologic Variable	Value	Median [IQR]								
		5-Minute Interval	10-Minute Interval	15-Minute Interval	30-Minute Interval	1-Hour Interval	2-Hour Interval	6-Hour Interval	12-Hour Interval	1-Day Interval
**HC Population**										
rSO_2_L	Bias	-0.18 [-0.18 – -0.18]	-0.01 [-0.56 – 0.51]	0.27 [-0.56 – 1.01]	–	–	–	–	–	–
	Lower LoA	-0.5 [-0.5 – -0.5]	-1.71 [-3.07 – -0.8]	-0.95 [-2.38 – -0.23]	–	–	–	–	–	–
	Upper LoA	0.14 [0.14 – 0.14]	1.88 [0.96 – 2.8]	1.66 [0.38 – 2.81]	–	–	–	–	–	–
	LoA Spread	0.64 [0.64 – 0.64]	3.39 [2.11 – 5.59]	2.47 [1.52 – 4.19]	–	–	–	–	–	–
	Relative Bias	-28.04 [-28.04 – -28.04]	-0.7 [-13.64 – 17.6]	9.84 [-19.87 – 36.84]	–	–	–	–	–	–
	Regression Slope	2 [2 – 2]	0.03 [-0.93 – 1.3]	1.3 [-0.23 – 2]	–	–	–	–	–	–
	Regression Intercept	-0.44 [-0.44 – -0.44]	-0.28 [-74.5 – 64.5]	-53 [-130 – 1.09]	–	–	–	–	–	–
rSO_2_R	Bias	-0.18 [-0.18 – -0.18]	-0.13 [-0.7 – 0.65]	0.03 [-1.1 – 0.95]	–	–	–	–	–	–
	Lower LoA	-0.5 [-0.5 – -0.5]	-1.99 [-3.3 – -1.19]	-1.71 [-3.06 – -0.76]	–	–	–	–	–	–
	Upper LoA	0.14 [0.14 – 0.14]	1.7 [0.89 – 3.38]	1.79 [0.8 – 2.96]	–	–	–	–	–	–
	LoA Spread	0.64 [0.64 – 0.64]	3.77 [2.33 – 6.03]	3.23 [2.41 – 5.15]	–	–	–	–	–	–
	Relative Bias	-28.04 [-28.04 – -28.04]	-4.71 [-21.05 – 11.62]	1.06 [-29.17 – 28.13]	–	–	–	–	–	–
	Regression Slope	2 [2 – 2]	0.35 [-0.67 – 2]	0.59 [-0.65 – 2]	–	–	–	–	–	–
	Regression Intercept	-0.44 [-0.44 – -0.44]	-17 [-132.5 – 43]	-41.5 [-140 – 43.5]	–	–	–	–	–	–
COx_L	Bias	–	–	–	–	–	–	–	–	–
	Lower LoA	–	–	–	–	–	–	–	–	–
	Upper LoA	–	–	–	–	–	–	–	–	–
	LoA Spread	–	–	–	–	–	–	–	–	–
	Relative Bias	–	–	–	–	–	–	–	–	–
	Regression Slope	–	–	–	–	–	–	–	–	–
	Regression Intercept	–	–	–	–	–	–	–	–	–
COx_R	Bias	–	–	–	–	–	–	–	–	–
	Lower LoA	–	–	–	–	–	–	–	–	–
	Upper LoA	–	–	–	–	–	–	–	–	–
	LoA Spread	–	–	–	–	–	–	–	–	–
	Relative Bias	–	–	–	–	–	–	–	–	–
	Regression Slope	–	–	–	–	–	–	–	–	–
	Regression Intercept	–	–	–	–	–	–	–	–	–
COx-a_L	Bias	-0.18 [-0.18 – -0.18]	-0.08 [-0.21 – 0.1]	-0.12 [-0.3 – 0.08]	–	–	–	–	–	–
	Lower LoA	-0.5 [-0.5 – -0.5]	-0.63 [-0.91 – -0.39]	-0.5 [-0.77 – -0.22]	–	–	–	–	–	–
	Upper LoA	-28.04 [-28.04 – -28.04]	-5.45 [-20.92 – 5.97]	-13.65 [-40.79 – 11.8]	–	–	–	–	–	–
	LoA Spread	0.64 [0.64 – 0.64]	1 [0.64 – 1.66]	0.83 [0.56 – 1.13]	–	–	–	–	–	–
	Relative Bias	0.14 [0.14 – 0.14]	0.51 [0.27 – 0.88]	0.28 [0.07 – 0.58]	–	–	–	–	–	–
	Regression Slope	-0.44 [-0.44 – -0.44]	-0.08 [-0.41 – 0.19]	-0.31 [-0.59 – 0.1]	–	–	–	–	–	–
	Regression Intercept	2 [2 – 2]	1 [-0.4 – 2]	2 [0.82 – 2.1]	–	–	–	–	–	–
COx-a_R	Bias	-0.18 [-0.18 – -0.18]	-0.01 [-0.13 – 0.13]	-0.02 [-0.2 – 0.14]	–	–	–	–	–	–
	Lower LoA	-0.5 [-0.5 – -0.5]	-0.47 [-0.79 – -0.26]	-0.49 [-0.68 – -0.28]	–	–	–	–	–	–
	Upper LoA	-28.04 [-28.04 – -28.04]	-1.21 [-16.98 – 13.09]	-4.69 [-26.88 – 13.04]	–	–	–	–	–	–
	LoA Spread	0.64 [0.64 – 0.64]	0.87 [0.54 – 1.32]	0.83 [0.61 – 1.23]	–	–	–	–	–	–
	Relative Bias	0.14 [0.14 – 0.14]	0.47 [0.16 – 0.65]	0.4 [0.13 – 0.66]	–	–	–	–	–	–
	Regression Slope	-0.44 [-0.44 – -0.44]	-0.04 [-0.33 – 0.16]	-0.09 [-0.44 – 0.12]	–	–	–	–	–	–
	Regression Intercept	2 [2 – 2]	1.4 [-0.07 – 2]	1.5 [0.08 – 2.18]	–	–	–	–	–	–
**SP Population**										
rSO_2_L	Bias	0.21 [0.21 – 0.21]	0.01 [-0.28 – 0.29]	0.72 [0.08 – 1.18]	0.17 [-0.84 – 1.39]	-0.57 [-2.03 – 0.77]	-1.51 [-2.04 – -0.36]	–	–	–
	Lower LoA	-0.89 [-0.89 – -0.89]	-5.75 [-7.25 – -3.02]	-7.33 [-14.21 – -3.91]	-7.48 [-12.8 – -3.97]	-8.76 [-15.13 – -4.39]	-11.52 [-17.45 – -7]	–	–	–
	Upper LoA	1.31 [1.31 – 1.31]	5.49 [3.54 – 7.83]	7.77 [3.97 – 17.04]	8.09 [5.68 – 13.36]	7.58 [3.95 – 16.36]	6.4 [5.23 – 13.37]	–	–	–
	LoA Spread	2.2 [2.2 – 2.2]	11.52 [6.37 – 15.73]	15.28 [8.32 – 31.24]	16.71 [10.86 – 25.11]	15.58 [9.24 – 29.42]	17.92 [12.24 – 30.82]	–	–	–

	Relative Bias	9.45 [9.45 – 9.45]	0.03 [-3.43 – 2.73]	3.96 [0.77 – 5.79]	2.92 [-4.75 – 12.42]	-2.85 [-9.44 – 7.35]	-3.47 [-8.88 – 4.35]	–	–	–
	Regression Slope	-0.33 [-0.33 – -0.33]	0.01 [-0.06 – 0.06]	-0.44 [-0.9 – -0.12]	-0.51 [-0.58 – -0.03]	0.01 [-1.02 – 0.97]	0.65 [0.42 – 1.33]	–	–	–
	Regression Intercept	0.26 [0.26 – 0.26]	-0.41 [-2.95 – 4.2]	26 [4.65 – 49.5]	25 [-1.27 – 45]	-1.71 [-67 – 64]	-55 [-102.5 – -33]	–	–	–
rSO_2_R	Bias	0.21 [0.21 – 0.21]	-0.13 [-0.43 – 0.53]	-0.01 [-0.85 – 0.98]	0.13 [-1.66 – 1.77]	0.17 [-1.56 – 1.17]	-1.86 [-3.03 – -0.32]	–	–	–
	Lower LoA	-0.89 [-0.89 – -0.89]	-5.72 [-8.32 – -3.31]	-9.63 [-21.82 – -5.38]	-9.77 [-13.91 – -4.54]	-7.26 [-11.39 – -2.96]	-16.56 [-20.78 – -9.59]	–	–	–
	Upper LoA	1.31 [1.31 – 1.31]	6 [4.07 – 7.36]	9.62 [6.8 – 20.21]	9.36 [7.25 – 19.04]	6.53 [4.44 – 10.09]	8.16 [6.61 – 14.71]	–	–	–
	LoA Spread	2.2 [2.2 – 2.2]	11.61 [7.54 – 15.63]	19.25 [12.75 – 42.02]	19 [12.03 – 36.16]	12.71 [8.48 – 25.04]	24.71 [16.2 – 35.49]	–	–	–
	Relative Bias	9.45 [9.45 – 9.45]	-1.12 [-3.47 – 4.65]	-0.05 [-3.5 – 6.11]	1.13 [-6.05 – 9.15]	2.42 [-10.93 – 12.92]	-4.03 [-10.51 – 5.94]	–	–	–
	Regression Slope	-0.33 [-0.33 – -0.33]	0 [-0.06 – 0.14]	-0.34 [-1.3 – -0.04]	-0.43 [-1.4 – 0.22]	0.38 [-0.85 – 1.4]	1.9 [1.13 – 1.95]	–	–	–
	Regression Intercept	0.26 [0.26 – 0.26]	0.12 [-8.6 – 4.5]	21 [2.81 – 71.5]	24 [-14.25 – 82]	-21 [-96.25 – 56.5]	-140 [-145 – -86]	–	–	–
COx_L	Bias	–	–	–	–	–	–	–	–	–
	Lower LoA	–	–	–	–	–	–	–	–	–
	Upper LoA	–	–	–	–	–	–	–	–	–
	LoA Spread	–	–	–	–	–	–	–	–	–
	Relative Bias	–	–	–	–	–	–	–	–	–
	Regression Slope	–	–	–	–	–	–	–	–	–
	Regression Intercept	–	–	–	–	–	–	–	–	–
COx_R	Bias	–	–	–	–	–	–	–	–	–
	Lower LoA	–	–	–	–	–	–	–	–	–
	Upper LoA	–	–	–	–	–	–	–	–	–
	LoA Spread	–	–	–	–	–	–	–	–	–
	Relative Bias	–	–	–	–	–	–	–	–	–
	Regression Slope	–	–	–	–	–	–	–	–	–
	Regression Intercept	–	–	–	–	–	–	–	–	–
COx-a_L	Bias	0.21 [0.21 – 0.21]	0 [-0.05 – 0.04]	0.09 [0 – 0.13]	-0.01 [-0.12 – 0.15]	0.04 [-0.06 – 0.14]	-0.1 [-0.1 – -0.03]	–	–	–
	Lower LoA	-0.89 [-0.89 – -0.89]	-0.85 [-0.98 – -0.73]	-0.95 [-1.16 – -0.8]	-1.06 [-4.02 – -0.73]	-0.74 [-0.95 – -0.5]	-0.71 [-0.89 – -0.56]	–	–	–
	Upper LoA	9.45 [9.45 – 9.45]	-0.23 [-3.01 – 2.54]	4.21 [0.01 – 5.85]	-0.51 [-4.87 – 7.57]	2.69 [-3.75 – 5.01]	-5.41 [-6.75 – -0.7]	–	–	–
	LoA Spread	2.2 [2.2 – 2.2]	1.68 [1.49 – 1.96]	2.06 [1.72 – 2.5]	1.99 [1.47 – 10.45]	1.47 [1.11 – 1.9]	1.23 [1.05 – 1.57]	–	–	–
	Relative Bias	1.31 [1.31 – 1.31]	0.83 [0.74 – 0.98]	1.02 [0.9 – 1.39]	1.01 [0.75 – 6.19]	0.69 [0.58 – 1.01]	0.51 [0.5 – 0.68]	–	–	–
	Regression Slope	0.26 [0.26 – 0.26]	0.02 [-0.04 – 0.07]	0.14 [0.06 – 0.23]	0.11 [-0.04 – 0.32]	-0.1 [-0.41 – 0.15]	-0.27 [-0.52 – -0.26]	–	–	–
	Regression Intercept	-0.33 [-0.33 – -0.33]	-0.14 [-0.2 – 0.08]	-0.53 [-0.91 – -0.1]	-0.41 [-1.55 – 0.31]	1.15 [0.04 – 1.98]	1.5 [1.25 – 1.7]	–	–	–
COx-a_R	Bias	0.21 [0.21 – 0.21]	0.02 [-0.04 – 0.07]	0.04 [-0.05 – 0.07]	0.01 [-0.09 – 0.19]	0.03 [-0.02 – 0.21]	0.09 [0.01 – 0.1]	–	–	–
	Lower LoA	-0.89 [-0.89 – -0.89]	-0.77 [-0.91 – -0.72]	-1.03 [-1.21 – -0.89]	-0.86 [-1.09 – -0.69]	-0.66 [-0.95 – -0.55]	-0.51 [-0.66 – -0.45]	–	–	–
	Upper LoA	9.45 [9.45 – 9.45]	1.36 [-1.87 – 4.33]	1.59 [-1.91 – 3.58]	0.64 [-3.46 – 11.77]	2.86 [-1.58 – 11.56]	7.21 [1.47 – 9.12]	–	–	–
	LoA Spread	2.2 [2.2 – 2.2]	1.61 [1.48 – 1.92]	2.16 [1.88 – 2.35]	1.71 [1.46 – 2.66]	1.64 [1.28 – 2.01]	1.19 [1.09 – 1.35]	–	–	–
	Relative Bias	1.31 [1.31 – 1.31]	0.9 [0.74 – 0.99]	1.12 [0.92 – 1.28]	0.94 [0.74 – 1.62]	0.73 [0.59 – 1.11]	0.68 [0.64 – 0.68]	–	–	–
	Regression Slope	0.26 [0.26 – 0.26]	0.02 [-0.03 – 0.08]	0.06 [0.02 – 0.19]	0.04 [-0.1 – 0.4]	-0.05 [-0.25 – 0.16]	-0.23 [-0.28 – -0.2]	–	–	–
	Regression Intercept	-0.33 [-0.33 – -0.33]	0.03 [-0.24 – 0.2]	-0.45 [-0.78 – -0.02]	-0.11 [-0.56 – 0.28]	0.33 [-0.37 – 1.78]	1.6 [1.6 – 1.6]	–	–	–
**TBI Population**										
rSO_2_L	Bias	-0.04 [-0.04 – -0.04]	-0.07 [-0.22 – 0]	0.01 [-0.11 – 0.11]	-0.13 [-0.48 – 0.27]	-0.08 [-0.62 – 0.46]	-0.14 [-0.81 – 0.48]	-0.28 [-1.34 – 0.49]	-0.82 [-1.78 – 0.5]	-1.24 [-3.48 – 0.52]
	Lower LoA	-1.19 [-1.19 – -1.19]	-4.44 [-7.73 – -3.22]	-5.81 [-9.89 – -4.03]	-11.63 [-22.17 – -6.11]	-15.97 [-74.71 – -9.58]	-12.33 [-34.49 – -8.1]	-10.04 [-22.92 – -6.82]	-10.99 [-19.69 – -7.77]	-11.4 [-20.03 – -8.75]
	Upper LoA	1.1 [1.1 – 1.1]	4.42 [3.15 – 7.82]	5.76 [4.11 – 10.23]	11.85 [5.89 – 21.25]	15.46 [9.62 – 79.43]	11.78 [8.09 – 34.6]	10.27 [6.74 – 21.24]	9.64 [6.84 – 16.19]	9.77 [6.4 – 14.53]
	LoA Spread	2.29 [2.29 – 2.29]	8.84 [6.39 – 15.25]	11.49 [8.02 – 20]	23.74 [11.9 – 43.96]	31.53 [19.09 – 154.14]	23.77 [16.24 – 70.92]	20.9 [14.03 – 42.19]	21.06 [15.93 – 30.43]	19.53 [16.32 – 33.76]
	Relative Bias	-1.95 [-1.95 – -1.95]	-0.82 [-1.77 – -0.05]	0.09 [-0.79 – 0.64]	-0.74 [-2.51 – 0.65]	-0.27 [-1.64 – 1.4]	-0.47 [-2.6 – 1.64]	-1.41 [-4.97 – 1.62]	-3.62 [-7.61 – 1.02]	-6.19 [-13.22 – 3.55]
	Regression Slope	-1.1 [-1.1 – -1.1]	0 [-0.02 – 0.02]	-0.05 [-0.12 – -0.01]	-0.33 [-0.76 – -0.06]	-0.52 [-1.65 – -0.12]	-0.21 [-0.74 – -0.02]	0.06 [-0.25 – 0.3]	0.1 [-0.04 – 0.6]	0.87 [0.17 – 1.8]
	Regression Intercept	0.11 [0.11 – 0.11]	-0.13 [-1.58 – 1.07]	3.25 [0.93 – 8.75]	19.5 [3.5 – 43.75]	34 [7.65 – 100]	13.5 [1.95 – 47.75]	-3.35 [-19 – 18.25]	-7.2 [-40 – 3.85]	-36 [-120 – -12]

rSO_2_R	Bias	-0.04 [-0.04 – -0.04]	-0.07 [-0.25 – -0.02]	0.02 [-0.05 – 0.13]	-0.06 [-0.65 – 0.41]	-0.01 [-0.78 – 0.36]	-0.13 [-1.18 – 0.95]	-0.27 [-1.14 – 0.78]	-0.7 [-2.03 – 0.25]	-1.91 [-3.77 – -0.01]
	Lower LoA	-1.19 [-1.19 – -1.19]	-4.86 [-8.09 – -2.92]	-6 [-10.64 – -3.64]	-10.55 [-24.9 – -6.47]	-13.31 [-48.36 – -6.85]	-13.12 [-37.55 – -7.01]	-10.21 [-18.19 – -6.92]	-12.3 [-20.17 – -8.01]	-12.53 [-19.91 – -8.84]
	Upper LoA	1.1 [1.1 – 1.1]	4.6 [2.86 – 7.55]	5.83 [3.68 – 10.84]	10.08 [6.79 – 26.15]	12.75 [6.63 – 46.46]	12 [6.79 – 37.01]	8.94 [6.49 – 18.55]	12 [7.75 – 19.7]	9.61 [6.38 – 17.62]
	LoA Spread	2.29 [2.29 – 2.29]	9.51 [5.83 – 15.64]	11.77 [7.26 – 21.59]	20.91 [12.92 – 49.39]	26.41 [13.58 – 92.11]	24.65 [14.24 – 73.95]	18.67 [13.48 – 35.74]	24.02 [14.97 – 39.4]	22.72 [17.3 – 33.23]
	Relative Bias	-1.95 [-1.95 – -1.95]	-0.99 [-2 – -0.19]	0.19 [-0.52 – 0.89]	-0.59 [-1.77 – 1.37]	-0.08 [-2.1 – 1.4]	-0.42 [-3.05 – 1.87]	-0.98 [-6.09 – 2.02]	-2.9 [-7.96 – 0.48]	-7.36 [-15.78 – -0.06]
	Regression Slope	-1.1 [-1.1 – -1.1]	0 [-0.02 – 0.02]	-0.04 [-0.09 – -0.01]	-0.28 [-0.77 – -0.09]	-0.26 [-1.35 – -0.08]	-0.2 [-1.18 – -0.01]	0.03 [-0.11 – 0.41]	0.25 [-0.15 – 0.63]	0.9 [0.21 – 1.6]
	Regression Intercept	0.11 [0.11 – 0.11]	-0.12 [-2.2 – 1.03]	2.95 [0.44 – 7.3]	18.5 [5.5 – 59.75]	18.5 [5.95 – 88.75]	12 [0.51 – 81.25]	-2.7 [-30.25 – 6.98]	-15 [-49.5 – 11]	-57 [-120 – -14]
COx_L	Bias	-0.04 [-0.04 – -0.04]	0 [-0.01 – 0]	0 [-0.01 – 0.01]	0 [-0.04 – 0.05]	0.01 [-0.02 – 0.03]	0 [-0.02 – 0.01]	-0.01 [-0.03 – 0.01]	0.01 [-0.02 – 0.02]	0.01 [-0.02 – 0.06]
	Lower LoA	-1.19 [-1.19 – -1.19]	-0.66 [-0.72 – -0.62]	-0.87 [-0.96 – -0.79]	-1.2 [-1.72 – -0.89]	-0.8 [-1.56 – -0.66]	-0.67 [-0.76 – -0.57]	-0.6 [-0.64 – -0.52]	-0.57 [-0.63 – -0.51]	-0.54 [-0.6 – -0.49]
	Upper LoA	1.1 [1.1 – 1.1]	0.65 [0.62 – 0.72]	0.88 [0.8 – 0.96]	1.21 [0.87 – 1.65]	0.81 [0.67 – 1.59]	0.66 [0.56 – 0.77]	0.55 [0.51 – 0.62]	0.57 [0.52 – 0.63]	0.6 [0.53 – 0.65]
	LoA Spread	2.29 [2.29 – 2.29]	1.31 [1.24 – 1.44]	1.74 [1.59 – 1.93]	2.45 [1.76 – 3.4]	1.59 [1.31 – 3.15]	1.32 [1.16 – 1.51]	1.15 [1.03 – 1.25]	1.13 [1.04 – 1.25]	1.14 [1.04 – 1.22]
	Relative Bias	-1.95 [-1.95 – -1.95]	-0.1 [-0.54 – 0.23]	0.06 [-0.54 – 0.7]	-0.15 [-1.47 – 1.47]	0.52 [-1.32 – 1.68]	-0.04 [-1.28 – 1.27]	-0.8 [-2.38 – 1.09]	0.87 [-1.97 – 2.3]	1.09 [-1.37 – 5.13]
	Regression Slope	-1.1 [-1.1 – -1.1]	-0.01 [-0.05 – 0.02]	-0.62 [-0.7 – -0.5]	-1.2 [-1.6 – -0.55]	-0.17 [-1.3 – 0.27]	0.46 [0.03 – 0.9]	1.35 [1.1 – 1.7]	1.65 [1.2 – 1.83]	1.8 [1.6 – 2]
	Regression Intercept	0.11 [0.11 – 0.11]	0 [-0.01 – 0]	0.02 [-0.01 – 0.06]	0.04 [-0.03 – 0.09]	0.01 [-0.02 – 0.08]	-0.01 [-0.05 – 0.03]	-0.03 [-0.11 – 0.02]	-0.03 [-0.11 – 0.01]	-0.04 [-0.15 – 0.07]
COx_R	Bias	-0.04 [-0.04 – -0.04]	0 [-0.01 – 0.01]	0 [-0.01 – 0.01]	0 [-0.06 – 0.04]	0 [-0.03 – 0.04]	0 [-0.02 – 0.02]	0 [-0.01 – 0.03]	0.01 [-0.02 – 0.03]	0.01 [-0.01 – 0.05]
	Lower LoA	-1.19 [-1.19 – -1.19]	-0.66 [-0.72 – -0.6]	-0.86 [-0.94 – -0.81]	-1.25 [-2.12 – -0.82]	-0.89 [-1.4 – -0.71]	-0.65 [-0.76 – -0.57]	-0.57 [-0.63 – -0.52]	-0.56 [-0.59 – -0.51]	-0.54 [-0.59 – -0.5]
	Upper LoA	1.1 [1.1 – 1.1]	0.67 [0.61 – 0.72]	0.86 [0.81 – 0.95]	1.22 [0.8 – 2.65]	0.86 [0.69 – 1.44]	0.66 [0.57 – 0.75]	0.59 [0.54 – 0.65]	0.57 [0.5 – 0.62]	0.58 [0.51 – 0.63]
	LoA Spread	2.29 [2.29 – 2.29]	1.33 [1.21 – 1.44]	1.72 [1.63 – 1.88]	2.38 [1.64 – 4.77]	1.72 [1.42 – 2.69]	1.28 [1.14 – 1.47]	1.16 [1.07 – 1.27]	1.12 [1.04 – 1.21]	1.14 [1.06 – 1.21]
	Relative Bias	-1.95 [-1.95 – -1.95]	-0.11 [-0.58 – 0.47]	0 [-0.7 – 0.68]	-0.05 [-1.12 – 1.65]	0.2 [-2.02 – 1.4]	0.22 [-1.5 – 1.43]	0.37 [-1.02 – 2.87]	0.51 [-2.17 – 2.92]	0.98 [-1.29 – 3.83]
	Regression Slope	-1.1 [-1.1 – -1.1]	-0.02 [-0.05 – 0.02]	-0.58 [-0.73 – -0.46]	-1.1 [-1.8 – -0.4]	-0.55 [-1.3 – 0.32]	0.58 [0.1 – 0.93]	1.2 [0.91 – 1.6]	1.6 [1.23 – 1.8]	1.8 [1.6 – 1.9]
	Regression Intercept	-0.04 [-0.04 – -0.04]	0 [-0.01 – 0.01]	0 [-0.01 – 0.02]	0 [-0.05 – 0.04]	0 [-0.03 – 0.03]	0 [-0.02 – 0.02]	0 [-0.02 – 0.02]	0 [-0.02 – 0.02]	0.01 [-0.01 – 0.05]
COx-a_L	Bias	-1.95 [-1.95 – -1.95]	-0.22 [-0.59 – 0.51]	-0.01 [-0.57 – 0.97]	-0.21 [-1.16 – 1.4]	0.04 [-1.27 – 1.28]	0.09 [-1.26 – 1.4]	-0.36 [-1.27 – 1.68]	0.29 [-1.87 – 1.81]	1.38 [-1.18 – 4.79]
	Lower LoA	2.29 [2.29 – 2.29]	1.29 [1.2 – 1.41]	1.69 [1.56 – 1.83]	2.38 [1.75 – 3.37]	1.55 [1.35 – 2.24]	1.24 [1.13 – 1.39]	1.09 [0.99 – 1.19]	1.07 [1 – 1.17]	1.05 [0.97 – 1.16]
	Upper LoA	-1.19 [-1.19 – -1.19]	-0.65 [-0.7 – -0.6]	-0.84 [-0.92 – -0.79]	-1.17 [-1.7 – -0.91]	-0.78 [-1.09 – -0.67]	-0.63 [-0.7 – -0.56]	-0.54 [-0.6 – -0.5]	-0.55 [-0.6 – -0.48]	-0.5 [-0.55 – -0.46]
	LoA Spread	1.1 [1.1 – 1.1]	0.64 [0.6 – 0.71]	0.85 [0.79 – 0.92]	1.21 [0.87 – 1.67]	0.78 [0.67 – 1.13]	0.63 [0.56 – 0.69]	0.54 [0.49 – 0.59]	0.54 [0.5 – 0.58]	0.56 [0.5 – 0.61]
	Relative Bias	0.11 [0.11 – 0.11]	0 [-0.01 – 0.01]	0.04 [0.02 – 0.08]	0.07 [0.01 – 0.16]	0.02 [-0.02 – 0.07]	-0.04 [-0.09 – 0]	-0.1 [-0.19 – -0.03]	-0.14 [-0.21 – -0.05]	-0.13 [-0.24 – -0.04]
	Regression Slope	-1.1 [-1.1 – -1.1]	-0.02 [-0.05 – 0.02]	-0.57 [-0.7 – -0.49]	-1.2 [-1.58 – -0.66]	-0.28 [-1.08 – 0.39]	0.65 [0.13 – 0.97]	1.4 [1.1 – 1.7]	1.65 [1.3 – 1.8]	1.8 [1.6 – 1.95]
	Regression Intercept	-0.04 [-0.04 – -0.04]	0 [-0.01 – 0.01]	0.01 [-0.01 – 0.02]	0 [-0.03 – 0.04]	0.01 [-0.03 – 0.03]	0 [-0.02 – 0.02]	0.01 [-0.01 – 0.03]	0.01 [-0.01 – 0.03]	0.02 [-0.01 – 0.05]
COx-a_R	Bias	-1.95 [-1.95 – -1.95]	-0.05 [-0.49 – 0.42]	0.44 [-0.93 – 1.17]	-0.04 [-1.47 – 1.6]	0.24 [-1.34 – 1.84]	-0.1 [-1.47 – 1.7]	0.75 [-0.9 – 2.75]	0.89 [-0.69 – 2.94]	1.76 [-1.12 – 4.94]
	Lower LoA	2.29 [2.29 – 2.29]	1.28 [1.18 – 1.42]	1.67 [1.54 – 1.83]	2.24 [1.82 – 3]	1.59 [1.34 – 2.33]	1.26 [1.13 – 1.42]	1.07 [1.01 – 1.19]	1.07 [0.98 – 1.17]	1.05 [0.96 – 1.16]
	Upper LoA	-1.19 [-1.19 – -1.19]	-0.63 [-0.7 – -0.6]	-0.86 [-0.92 – -0.76]	-1.11 [-1.56 – -0.93]	-0.79 [-1.14 – -0.66]	-0.63 [-0.73 – -0.56]	-0.53 [-0.58 – -0.48]	-0.52 [-0.57 – -0.48]	-0.5 [-0.57 – -0.46]
	LoA Spread	1.1 [1.1 – 1.1]	0.64 [0.58 – 0.71]	0.84 [0.77 – 0.93]	1.1 [0.9 – 1.43]	0.8 [0.65 – 1.18]	0.63 [0.57 – 0.71]	0.55 [0.51 – 0.6]	0.53 [0.49 – 0.59]	0.54 [0.48 – 0.61]
	Relative Bias	0.11 [0.11 – 0.11]	0 [-0.01 – 0.01]	0.03 [0.02 – 0.08]	0.07 [0.01 – 0.17]	0.03 [-0.02 – 0.07]	-0.02 [-0.06 – 0.03]	-0.08 [-0.16 – 0.02]	-0.11 [-0.2 – -0.02]	-0.13 [-0.25 – -0.01]
	Regression Slope	-1.1 [-1.1 – -1.1]	-0.01 [-0.05 – 0.02]	-0.59 [-0.68 – -0.49]	-1.1 [-1.5 – -0.66]	-0.3 [-1.18 – 0.12]	0.54 [0.1 – 0.9]	1.3 [0.99 – 1.6]	1.6 [1.4 – 1.9]	1.8 [1.6 – 2]
	Regression Intercept	0.11 [0.11 – 0.11]	0 [-0.01 – 0.01]	0.02 [-0.03 – 0.07]	0.03 [-0.06 – 0.09]	-0.01 [-0.06 – 0.04]	0 [-0.04 – 0.04]	-0.02 [-0.09 – 0.05]	-0.02 [-0.13 – 0.09]	-0.04 [-0.13 – 0.08]

COx, cerebral oximetry index with cerebral perfusion pressure; COx-a, cerebral oximetry index with arterial blood pressure; HC, healthy control volunteer group; IQR, interquartile range; r-value, Pearson correlation coefficient; rSO_2, regional cerebral oxygen saturation; SP, elective spinal surgery patient group; TBI, traumatic brain injury patient group.
